# Broadening the Applicability of a Custom Multi-Platform Panel of Microhaplotypes: Bio-Geographical Ancestry Inference and Expanded Reference Data

**DOI:** 10.3389/fgene.2020.581041

**Published:** 2020-10-20

**Authors:** María de la Puente, Jorge Ruiz-Ramírez, Adrián Ambroa-Conde, Catarina Xavier, Jorge Amigo, María Ángeles Casares de Cal, Antonio Gómez-Tato, Ángel Carracedo, Walther Parson, Christopher Phillips, María Victoria Lareu

**Affiliations:** ^1^Forensic Genetics Unit, Institute of Forensic Sciences, University of Santiago de Compostela, Santiago de Compostela, Spain; ^2^Institute of Legal Medicine, Medical University of Innsbruck, Innsbruck, Austria; ^3^Fundación Pública Galega de Medicina Xenómica (FPGMX), Santiago de Compostela, Spain; ^4^Faculty of Mathematics, University of Santiago de Compostela, Santiago de Compostela, Spain; ^5^Forensic Science Program, The Pennsylvania State University, University Park, PA, United States

**Keywords:** microhaplotypes, massively parallel sequencing, bio-geographical ancestry, mixed DNA, human identification

## Abstract

The development of microhaplotype (MH) panels for massively parallel sequencing (MPS) platforms is gaining increasing relevance for forensic analysis. Here, we expand the applicability of a 102 autosomal and 11 X-chromosome panel of MHs, previously validated with both MiSeq and Ion S5 MPS platforms and designed for identification purposes. We have broadened reference population data for identification purposes, including data from 240 HGDP-CEPH individuals of native populations from North Africa, the Middle East, Oceania and America. Using the enhanced population data, the panel was evaluated as a marker set for bio-geographical ancestry (BGA) inference, providing a clear differentiation of the five main continental groups of Africa, Europe, East Asia, Native America, and Oceania. An informative degree of differentiation was also achieved for the population variation encompassing North Africa, Middle East, Europe, South Asia, and East Asia. In addition, we explored the potential for individual BGA inference from simple mixed DNA, by simulation of mixed profiles followed by deconvolution of mixture components.

## Introduction

Microhaplotypes (MHs), defined as sets of SNPs in sequence segments of less than 200 base-pairs (bp), which define multi-allelic haplotypes, have been proposed as forensic markers in concert with the forensic adoption of massively parallel sequencing (MPS) technologies (Kidd et al., 2014; Oldoni et al., 2018). MPS platforms allow the detection of the phase of the SNP alleles in MH loci from the generated monoclonal (single strand) sequences, in contrast to other SNP genotyping methods used in forensics (Sobrino et al., 2005) or Sanger sequencing. The favorable characteristics of MH loci has prompted the search and characterization of new MH markers for forensic use and their genotyping using MPS-based panels (Kidd and Speed, 2015; Kidd et al., 2017; Chen et al., 2018, 2019a,b; van der Gaag et al., 2018; Voskoboinik et al., 2018; Bennett et al., 2019; De La Puente et al., 2019; Phillips et al., 2019; Turchi et al., 2019; Gandotra et al., 2020; Sun et al., 2020).

Three notable advantages of MHs are: a higher degree of polymorphism compared to single-site SNPs; the absence of stutter artifacts; and short amplicon lengths compared to STRs. Therefore, possible applications of MHs include a wide range of forensic scenarios: individual identification from degraded DNA (van der Gaag et al., 2018), kinship testing (Sun et al., 2020), mixture analysis (Voskoboinik et al., 2018; Bennett et al., 2019; Chen et al., 2019a) and bio-geographical ancestry (BGA) prediction (Chen et al., 2019b; Phillips et al., 2019). Moreover, the same markers have been proposed for multiple forensic applications examined simultaneously, constituting a multi-purpose set of panels (Oldoni et al., 2017; Turchi et al., 2019; Gandotra et al., 2020).

Here, we have made new evaluations of a previously published multi-platform (MiSeq and Ion S5) panel of 102 autosomal and 11 X-chromosome MHs validated for forensic identification (De La Puente et al., 2019) (herein MHs-panel), in order to: (i) expand the available reference dataset with native populations from major groups not covered by the 1,000 Genomes Project; (ii) provide a comprehensive description of the BGA prediction capabilities of the panel; and (iii) test the possibility of obtaining individual BGA predictions from the deconvoluted contributors detected in simple mixed profiles.

## Materials and Methods

### DNA Samples, Library Construction and Sequencing

A total of 246 DNAs were analyzed from the HGDP-CEPH Human Genome Diversity Panel (Cann et al., 2002) (herein CEPH), comprising: (i) 28 Oceanians–17 Papuan from New Guinea and 11 Melanesian from Bougainville; (ii) 62 Native Americans–14 Karitiana, 8 Surui from Brazil; 20 Maya, 13 Pima from Mexico; and 7 Piapoco from Colombia; (iii) 127 Middle East–40 Druze from Israel (Carmel), 42 Palestinian from Israel (Central), 45 Bedouin from Israel (Negev); and (iv) North-Africans–29 Mozabite from Algeria (Mzab).

Library preparation was performed with AmpliSeq Precision ID Library Kit [Thermo Fisher Scientific (TFS)] and Ion Xpress Barcode Adapters (TFS) optimizing the manufacturer’s recommendations to half-volumes. A total of 1 ng of input DNA was used, quantified with Qubit 3.0 Fluorometer (TFS) and Qubit dsDNA HS Assay Kit (TFS) following the manufacturer’s recommendations. The primer pool was described in De La Puente et al. (2019). Briefly, a total of 107 (10 Mb-spaced) autosomal and 11 (5 Mb-spaced) X-chromosome short highly polymorphic MHs were identified from 1,000 Genomes public data as optimal forensic MH markers and incorporated in a single-pool Hotspot AmpliSeq design targeting Formalin-Fixed Paraffin-Embedded (FFPE) DNA (i.e., with amplicons of 125–175 nucleotide lengths highly suitable for degraded DNA). Individual libraries were quantified with the Ion Library TaqMan Quantitation Kit (TFS), following manufacturer’s protocols. Equimolar pools of 39 to 46 libraries at 20–30 pM were prepared for sequencing. Template preparation was performed using the Ion 510, Ion 520, Ion 530 Kit-Chef (TFS), Ion 530 chips (TFS) and the Ion Chef Instrument. Sequencing was performed on the Ion S5 instrument with a read length of 200 (500 flows).

### Data Curation and Concordance With Databases

Sequencing quality parameters including sequence coverage, strand bias, allele balance and misincorporation rates were evaluated using single SNP data produced with the HID Genotyper plugin v.5.2.2 (TFS) of Torrent Suite v. 5.6.0 (TFS) using default parameters of minimum coverage of six reads and minimum allele read frequency of 0.1.

Microhaplotype calling was performed using the pipeline described in De La Puente et al. (2019), that allows inferring the phase of the SNPs on the same amplicon from the sequence reads obtained. Briefly, FASTQ reads were aligned using Burrows-Wheeler aligner (BWA) (Li and Durbin, 2009) to a customized reference genome comprising each MH amplicon joined. Alignments were processed with SAMtools (Li et al., 2009) to create the input files for the microhaplot R package (Thomas, 2019), which outputs a raw table of allele strings and depth per MH. Minor allele read frequency and minimum coverage filtering parameters were set to the default values of 0.1 and 15, respectively. A total of five MHs: 3pC, 5qD, 10qC, 12qA, and 19qB, were included in the primer set but previously identified as unreliable and therefore excluded from analysis; and genotypes were manually corrected, when necessary, according to the guidelines in De La Puente et al. (2019).

Genotyping and phase concordance with publicly available data from Simons Genome Diversity Project (SGDP) (Mallick et al., 2016) and recent whole genome sequencing of the HGDP panel (Almarri et al., 2020; Bergstrom et al., 2020) (herein HGDP WGS) was evaluated. SGDP dataset is phased using the probabilistic software IMPUTE2 (Howie et al., 2009) with 1,000 Genomes data as reference. SGDP lists whole-genome variant data for 280 worldwide samples, but 21 are overlaps with 1,000 genomes sample sets, and 133 are samples from the CEPH panel. In total, 35 CEPH samples overlapped between SGDP and those we genotyped from Middle East, Oceanian and American populations. The HGDP WGS dataset infers the phase of heterozygous SNPs with GATK HaplotypeCaller (McKenna et al., 2010; Poplin et al., 2018) for a total of 929 HGDP-CEPH panel samples of which 234 overlap with those we genotyped for the MH loci. GATK HaplotypeCaller reassembles active regions with significant variation in order to identify all the possible haplotypes, then for each haplotype a likelihood is calculated given the sequence read data by aligning each read against each haplotype and based on those likelihoods the genotypes are assigned.

### Population Metrics and Bio-Geographical Ancestry Analysis

Population data for haplotype frequency estimation and BGA analysis was obtained from 1,000 Genomes project phase III public releases (The Genomes Project Consortium, 2015) (herein 1 KG) and the genotyping of HGDP-CEPH populations. Additionally, data for 679 HGDP-CEPH individuals from 42 Sub-Saharan African, European, Central and South Asian and East Asian populations was collected from HGDP WGS. These populations comprise a limited number of individuals and descriptive analyses such as frequencies or F_ST_ were not conducted.

Population haplotype frequencies, expected Heterozygosity values (as 1 minus the sum of the squares of the haplotype frequencies) and cumulative match probabilities (as the product of the sum of the squares of the genotype probabilities of each locus) were calculated and plotted using R v. 3.6.1 (R Core Team, 2019) or Excel spreadsheets. F_ST_ and average number of pairwise differences within and between population were calculated using Arlequin version 3.5.1.2 (Excoffier and Lischer, 2010).

Bio-geographical ancestry analyses were conducted considering the autosomal MHs as independent markers and their haplotypes as alleles. Analyses with STRUCTURE v. 2.3.4 (Pritchard et al., 2000) were performed following guidelines in Porras-Hurtado et al. (2013), including the following parameters: five iterations for each K, one million burnin steps and one million MCMC steps, correlated allele frequencies under the Admixture model. When combining both reference and non-reference populations, the option “Update allele frequencies using only individuals with POPFLAG = 1” was selected and reference populations were set to 1. The optimum K was estimated considering the output graphs generated with Structure Harvester (Earl and Von Holdt, 2012). Ancestry membership was plotted using the CLUMPAK portal (Kopelman et al., 2015). Multidimensional scaling (MDS) analyses and Neighbor-joining (NJ) trees were constructed with R v. 3.6.1 (R Core Team, 2019) over an allele-distance matrix computed using the R package *pegas* (Paradis, 2010).

Population-specific Divergence (PSD) and simple pairwise Divergence values were calculated using infocalc v. 1.1 for obtaining Rosenberg’s informativeness-for-assignment metric (I*n*) (Rosenberg et al., 2003). For PSD, individual profiles were marked as AFR and non-AFR, etc.; and for pairwise comparisons, each pair of populations was grouped. In values for each autosomal MH were summed to obtain cumulative values. As explained in Cheung et al. (2019), I*n* is the most convenient metric for assessment of BGA informativeness in different types of genomic markers.

### Mixture Simulation, Profile Deconvolution and BGA Inference From Components

Three mixed profiles including 102 autosomal MHs were simulated from single source profiles of known ancestry, comprising: (i) a 1:3 mixture of HG02922 unadmixed ESN (AFR) and NA18939 unadmixed JPT (EAS)–herein, mixture 1; (ii) a 1:5 mixture of HG00097 unadmixed GBR (EUR) and HG00096 unadmixed GBR (EUR)–herein, mixture 2; and (iii) a 1:7 mixture of HG01565 admixed PEL (AMR) and HG00096 unadmixed GBR (EUR)–herein, mixture 3.

Two analysts conducted a blind deconvolution of each of the mixed profiles, instructed to separate two components (minor and major) assigning only the haplotypes that were unequivocally from one of the components when taking into account stochastic phenomena (allele drop-out, heterozygous imbalance). Results from both analysts were merged maintaining the most conservative profile when interpretations differed, and BGA inference analysis comprising STRUCTURE and MDS were performed as described in section “Population Differentiation and BGA Inference Performance.”

## Results

### Assay Performance and Genotyping Data Curation

Details of the overall performance of the seven sequencing runs are collected in Supplementary Table S1. All chips reached a satisfactory loading performance, with percentages ranging from 72 to 90%. In order to reduce the high proportion of polyclonal reads observed initially (38%), the molar concentration of the library pool was progressively lowered to 20 pM. Even when the number of chips is not statistically sufficient to test this effect, a tendency toward lower polyclonality was generally observed, except for chip 4.

Supplementary Table S2 and Supplementary Figure S1 outline the target coverage per sample. Samples reached comparable levels of overall mean coverage value across MHs of 3,572.33 ± 2,601.39 reads. Uniformity was maintained both within and among sequencing runs, with few samples giving values beyond the overall mean coverage. Most samples from chip 4 showed lower median coverage values, probably due to the fact that sample HGDP00693 had mean coverage values nearly eight times higher than the overall mean (28,313.96 ± 9,743). This excessive sequence coverage was most likely caused by erroneous quantification of the sample library (i.e., the library concentration was underestimated and pooled at a much higher concentration than 20 pM) and explains the high polyclonality of chip 4.

Supplementary Figure S2 shows normalized coverage values per marker, calculated as MH coverage per sample/total sample coverage. As expected, from previous analyses using the same primer pools, results closely match those found from the initial panel validation (De La Puente et al., 2019), with 6pB, 17qC, XpB, and 16pB having the lowest normalized coverage values. Coverage values per marker in each sample are shown in Supplementary Figure S3. For the problem MHs mentioned above, a high proportion of samples did not reach a minimum of 15 reads, affecting the calling process and genotype completeness of the typed samples. This was anticipated before sequencing but the loss of data from these discounted MH loci did not unduly affect the panel’s informativeness, taking into account the fact that most BGA panels can accommodate some degree of missing values.

Regarding strand bias, represented in Supplementary Figure S4, most MHs ranged between the 40–60% of forward coverage/total coverage. When compared to the initial evaluation, MHs XqA and 12pA presented a slight degree of reverse strand bias, which had not been previously observed. In contrast, 11qC and 19qA presented some forward strand bias uniquely in this study.

Allele read frequency balance is described in Supplementary Figure S5 as the percentage of reference allele sequence reads. For single source DNA samples, these frequencies would ideally cluster closely around 50% for heterozygous genotypes and 0 or 100% in homozygotes for the alternative or reference allele, respectively. Most MHs values are close to the expected values, with few outliers. MHs 6pB, 17qC, XpB, and 16pB display highly scattered plots that can be explained by stochastic PCR effects due to low coverage, as is often observed. In contrast with the initial evaluation, MHs 1qC, 7pC, 14qA, and 19qA showed adequate balance in this study, possibly due to the effect of a higher sample size.

Supplementary Figure S6 outlines the mean percentage misincorporation (as non-allelic bases detected at the SNP site/total coverage). Overall misincorporation rates reached levels of 0.29 ± 0.71%, a value closely matching that previously observed for these loci (0.25 ± 0.73%). Outlier misincorporation rates between the 5 and 1% thresholds were observed in MHs 15qB (4.69%), 1pC (3.21%), 4qB (2.32%), 6qD (1.60%), 13qD (1.47%), 9qA (1.52%), XqA (1.25%), 21qA (1.22%) 13qB (1.11%), and 7qC (1.03%). Some of these MHs were previously reported as sited within repetitive regions. However, these values did not come close to the 10% minimum allele read frequency used for MH-allele calling, and therefore, genotyping accuracy was not unduly affected.

After MH component SNP genotype calling, six samples: HGDP00588, HGDP00627, HGDP00634, HGDP00637, HGDP00640, and HGDP00642 showed highly imbalanced profiles with more than two haplotypes for several markers, and were excluded from further analysis, as this was most probably due to reference DNA contamination.

### Concordance With Online Variant Databases

Concordance with SGDP phased data comprised a total of 3,220 comparisons for 92 markers–note that all X-chromosome loci plus 10 autosomal MHs are not listed by SGDP. Comparisons were made in 35 samples (17 OCE, 10 AMR, 6 ME and 2 NAF). In addition, 82 genotypes could not be compared due to the lack of results from genotyping, most of these in MHs that showed the lowest coverage values: 6pB, 17qC, and 16pB. Concordance rates reached levels of 99.01%, with 31 discordances in 3,138 genotypes confined to 10 MHs, as listed in Supplementary Table S3.

All the discordancies were explored further in IGV in order to clarify possible causes. Most discordancies (21/31) we presume to be caused by the use of probabilistic software to phase the SGDP SNP genotype data (i.e., with IMPUTE2 software) in the following two ways: (i) erroneous phasing of heterozygous alleles in MHs 13qD, 20pA, and 22qA; or (ii) the software does not account for multi-allelic SNPs (i.e., more than two common alleles at the SNP site)–affecting MHs 1qC, 5qB, and 11qA. This supports the idea that more accurate phasing is obtained through applying MPS to short MHs sequenced as single strands, rather than inferring phase from individually genotyped SNPs.

For MH 16qB, previously identified as underperforming, a total of seven discordancies were found, due to allele drop-out (i.e., one of the alleles did not reach the minimum coverage threshold of 15 reads) during genotype calling. These genotypes were corrected for further analysis. Also, single discordancies were found for MHs 1qD, 7pC, and 11pA. In 11pA the discordancy was due to high allele imbalance of the sequence reads and was corrected; while the cause of the others remained unclear.

For concordance with HGDP WGS, a total of 234 out of the 240 analyzed samples–i.e., all excluding HGDP01003, HGDP01006, HGDP01042, HGDP01051, HGDP01273, and HGDP01278–were compared in 113 loci, adding up to a total of 26,442 comparisons. A total of 1,321 comparisons were inconclusive due to: (i) lack of genotypes in either dataset; (ii) HGDP WGS does not list the first SNP of MH XqD, located in position 93531382 (GRCh37/hg19) or 94276383 (GRCh38/hg38) and (iii) HGDP WGS does not provide phase information for the loci located on the X, thus haplotype reconstruction was not possible for MH loci comprising two or more heterozygous SNPs.

The concordance rate reached 99.75%, with only 62 discrepancies observed (details can be found in Supplementary Table S4). Similar to the comparisons with SGDP, the majority of these discordances (43/62) were observed in MHs 16pB, XpB, 17qC, and 6pB (with 18, 13, 4, and 4 discordances each, respectively), previously defined as misperforming markers in terms of coverage which caused high allele imbalance and allelic drop-out.

Low coverage and high allelic imbalance were also the causes for three discordances in MH 7pC; three in MH 12qC and one in MH 11pA. In addition, eight discrepancies were related to the phasing of MHs 1qD, 3qC, 13qD, and 17qA; these were analyzed thoroughly in IGV in order to confirm the phase obtained through the MHs panel. Moreover, raw data from the HGDP WGS sequencing project was inspected in IGV, resulting in confirmation of the phase obtained for the MHs panel. Therefore, the phasing algorithm performed errouneously in a very limited number of cases, which could be due to the fact that WGS reads do not neccesarily reach all the SNPs in the amplicon.

### Population Metrics

Details of the thirty populations included in this study are listed in Supplementary Table S5. Eight major populations were considered: AFR, sub-Saharan Africa; EUR, Europe; NAF, North Africa; ME, Middle East; SAS, South Asia; EAS, East Asia; OCE, Oceania; and AMR, America. For each major population, all individuals from different CEPH populations were gathered into a single population group, in order to achieve high sampling scales, although this was still relatively small for Oceanians.

Allele frequency estimates for 30 populations are given in Supplementary File S1 and genotypes/haplotypes listed in Supplementary Table S6. The latter contains information on the total number of chromosomes typed and data completeness per MH; and total number of counts per SNP allele. This information is intended to emphasize the need for caution with the frequency estimates derived from populations with few sampled individuals, especially NAF and OCE; as well as highlighting the underperforming MHs such as 6pB, 17qC, XpB, and 16pB.

**FIGURE 1:**
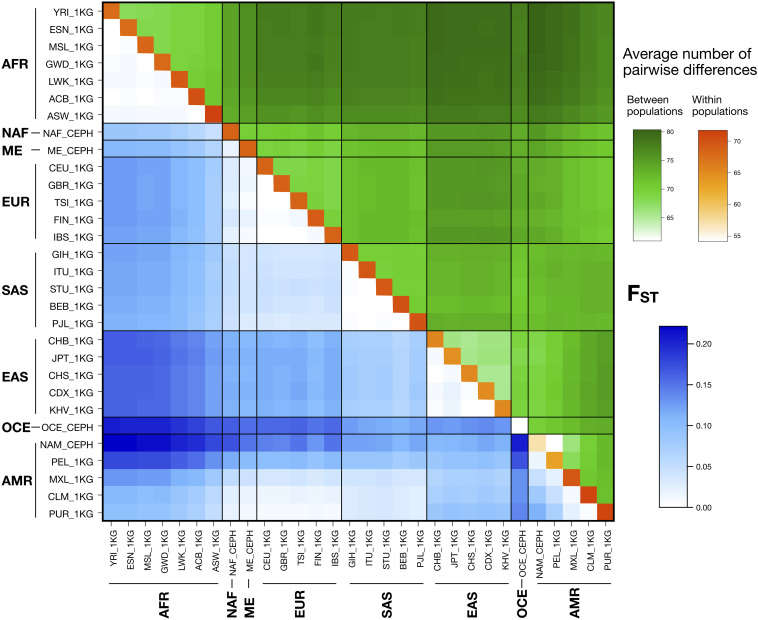
Pairwise F_ST_ (blue) and number of pairwise genotype differences between (green) and within (orange) populations for the autosomal MHs. Populations are named and grouped into eight major populations according to Supplementary Table S5.

Figure 1 represents pairwise F_ST_ values and average numbers of pairwise differences within and between populations, considering data from the 102 autosomal MHs. Pairwise F_ST_ values ranged from 7.00E-5 to 2.21E-1. As expected, low values were found when comparing populations within the same major population group and for comparisons including those between admixed AMR populations with higher proportions of European contributions (CLM, PUR) and the EUR populations. Higher values were found in comparisons between the AFR populations and EAS, OCE and AMR populations with a low degree of admixture, following the known demographic histories of continental populations. Likewise, the average number of pairwise differences between populations ranged from 60.94 to 80.35 and showed similar patterns to F_ST_–with the low values corresponding to comparisons inside the same major population group and high values in the comparisons involving an AFR population. The lowest value was recorded for the comparison of Native Americans (NAM) with the least admixed 1 KG AMR population of Peruvians from Lima (PEL). Average number of pairwise differences within populations ranged from 54.13 to 71.70 with the lowest values in NAM and OCE populations.

Heterozygosity values for the autosomal MHs are listed Supplementary Table S7 and represented graphically in Supplementary Figure S7. Heterozygosity showed variance both among markers (Supplementary Figure S7A) and populations (Supplementary Figure S7B). Overall mean Heterozygosity values were 0.67 ± 0.09 for autosomal MHs, close to the 0.667 level of a perfectly balanced tri-allelic marker. A single MH, 20pC, gave values lower than 0.5 and the rest had values ranging from 0.49 to 0.81, approaching the 0.5 and 0.75 theoretical limits of bi- and tetra-allelic single-site SNPs. Consistent with their inheritance patterns, X-chromosome MHs showed a lower overall mean Heterozygosity of 0.564 ± 0.118. In terms of populations, all showed comparable levels, but NAM and OCE populations had the lowest values, matching patterns of increasing homozygosity with distance from East Africa.

**FIGURE 2:**
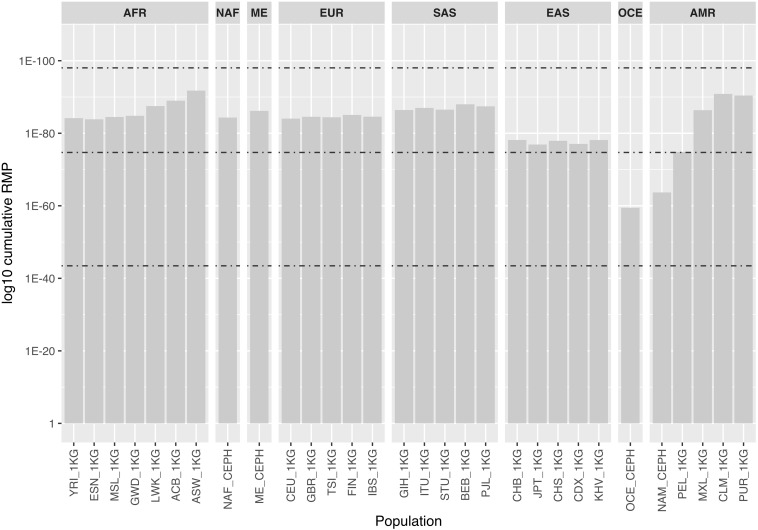
Bar chart represents log10 cumulative random match probability values (i.e., the probability that two individuals share the same profile) for the 30 populations considered, based on the autosomal MH data only. Populations are named and grouped into eight major populations according to Supplementary Table S5. Dashed lines represent, from bottom to top, the theoretical values for a panel composed of 102 perfectly balanced bi, tri and tetra-allelic SNPs for comparison: 3.56E-44, 1.98E-75, and 9.32E-99, respectively.

Figure 2 shows cumulative random match probability (RMP) for the 30 populations considering the autosomal MHs. Values for most populations ranged between 1.98E-75 and 9.32E-99, the maximum theoretical values for a panel of 102 tri- and tetra-allelic markers. As a consequence of their lower level of variability, NAM and OCE showed the lowest values. This decrease in discrimination power in such populations should be taken into account when assessing the use of the panel for analyzing distant pedigrees.

### Population Differentiation and BGA Inference Performance

Bio-geographical ancestry inference analyses were performed considering genetic information from the 102 autosomal MHs in the panel. In order to minimize possible sample size effects (Onogi et al., 2011), a reference set was constructed by selecting from each major population a single unadmixed population from the total of 30 previously described, as recorded in Supplementary Table S5. Additionally, classification was performed at two levels: (i) five major populations–AFR, EUR, EAS, OCE and AMR–for a first approach at a continental level (herein continental), followed by a second approach when appropriate (ii) with the five main Eurasian populations of NAF, ME, EUR, SAS, EAS to achieve a more detailed analysis of the variability continuously distributed North of the Sahara Desert, forming a natural barrier, and extending across Eurasia from NW to SE of this region (herein NAF-Eurasia). These hierarchical levels are devised so the substructure within NAF-Eurasia can be efficiently detected after a major continental comparison, as suggested in Rosenberg et al. (2002) and Evanno et al. (2005).

**FIGURE 3:**
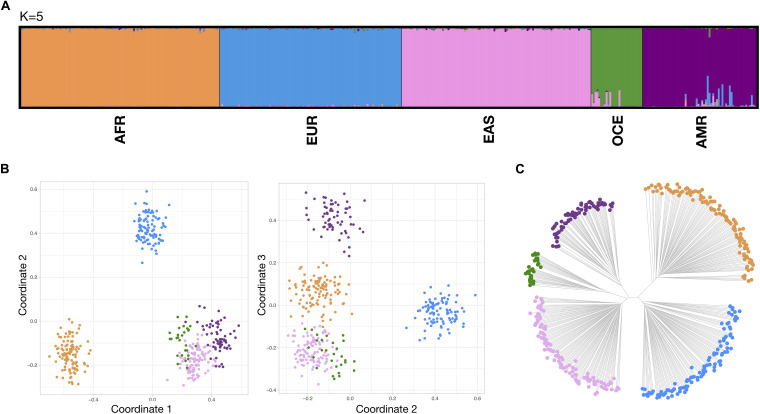
Bio-geographical ancestry analysis of the five continental reference populations. **(A)** STRUCTURE results of ancestry proportions at *K* = 5. Each bar represents an individual and is colored in segments whose lengths correspond to their genetic cluster membership coefficients in up to five inferred population groups. **(B)** Three dimensional MDS analysis showing coordinates 1 and 2 (left) and 2 and 3 (right). **(C)** Neighbor Joining (NJ) tree analysis. For the MDS and NJ-tree plots, populations are colored according to the five different clusters which correspond to the five major populations identified in the STRUCTURE plot.

Figure 3 compiles results from STRUCTURE, three dimensional MDS and neighbor-joining tree (NJ tree) for the reference populations at the continental level. In STRUCTURE, exploratory runs from *K* = 1 to *K* = 8 (detailed in Supplementary Figure S8–left) showed the most consistent cluster patterns at *K* = 5, supported both by the plateau at the mean of estimated Ln probability of data and the peak at Delta K. This five-group differentiation was also observed in the NJ tree, splitting into a 3–2 branch pattern, while some overlap between the OCE and EAS clusters persists in the MDS analysis. Both PSD and pairwise Divergence cumulative values, presented in Supplementary Figure S9–top, provided a relatively good balance between major population groups. Supplementary Figure S10 includes non-reference populations for the continental level. Unadmixed populations were predominantly assigned to their reference populations in all analysis systems, while admixed populations exhibited the expected patterns, showing mixed co-ancestry membership proportions in STRUCTURE and showing a spread distribution of points between the component clusters in the MDS and NJ tree plots.

**FIGURE 4:**
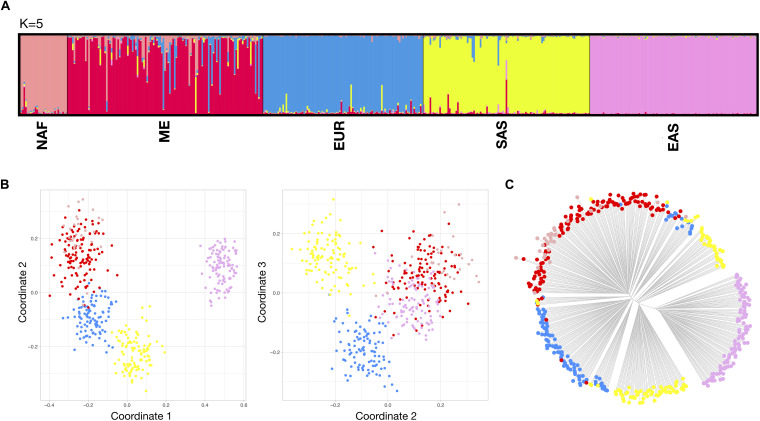
Bio-geographical ancestry analysis of the five NAF-Eurasia reference population sets. **(A)** STRUCTURE results of ancestry proportions at *K* = 5. Each bar represents an individual and is colored in segments whose lengths correspond to their genetic cluster membership coefficients in up to five inferred population groups. **(B)** Three dimensional MDS analysis showing coordinates 1 and 2 (left) and 2 and 3 (right). **(C)** Neighbor Joining (NJ) tree analysis. For the MDS and NJ-tree, populations are colored according to the five different clusters which correspond to the five major populations identified in the STRUCTURE plot.

For differentiations at the NAF-Eurasia level, results are compiled in Figure 4. Exploratory STRUCTURE runs (Supplementary Figure S8–right) showed a higher degree of irregular cluster membership patterns for SAS and ME. Optimal K was selected at 5, taking into account the plateau at the mean of estimated Ln probability of data. However, the Delta K graph showed a peak at *K* = 4, that arguably points to a slightly lower degree of differentiation between NAF and ME, as might be expected given their almost continuous regional distribution in the southern Mediterranean. These two population groups are often considered together for BGA analysis, but further expansion of the reference data, especially for NAF, could enhance the somewhat low levels of contrast found in our analyses. For the MDS analyses, a higher dispersion of the clusters was observed in comparison with the analysis at continental level, with some overlap between NAF and ME. The NJ tree plot shows a distinct EAS branch and a complex hierarchical pattern for SAS, EUR, ME and NAF branches. As expected, cumulative PSD and pairwise Divergence (Supplementary Figure S9–bottom) showed lower values and higher imbalance in these sets of populations in comparison to the more balanced continental differentiation. Pairwise Divergence increased accordingly to geographic distance, with comparisons including EAS reaching the highest values and the lowest values recorded for the closest pairs of NAF-ME, ME-EUR, and EUR-SAS. Supplementary Figure S11 assembles analysis including non-reference populations at the NAF-Eurasia level. All the tested unadmixed populations showed similar behavior to their reference populations.

Supplementary Figure S12 shows the population assignment analysis of the 42 Sub-Saharan African, European, Central and South Asian and East Asian populations from HGDP WGS against the continental and NAF-Eurasian reference populations, indicating the expected patterns. Central and South Asian populations show a clear frequency cline of admixture between European and East Asian ancestries at the continental level that can also be observed in both the MDS and NJ graphical summaries. At the Eurasian level, these populations show in STRUCTURE a complex mixture of ancestries with a predominant SAS component, despite the fact that none of these populations are located in the Indian sub-continent (unlike the reference populations), and this is reflected in the MDS plot showing a widely distributed set of points centered in the SAS cluster and extending to the NAF, ME, EUR, and EAS clusters.

### BGA Inference From Mixtures

Simulated profiles from mixtures 1, 2, and 3 are shown in Supplementary File S2, while Supplementary Table S8 contains information on both the individual profiles forming the mixtures and the deconvoluted major and minor components. All the haplotypes were assigned correctly to the previously known mixture contributors. Discrepancies between analysts were observed only for the more balanced ratio of 1:3 and were consistent with differences on the degree of risk assumed when assigning the alleles. For example, for MH 2pA analyst 1 assigned haplotypes TAAT/TAAT for the major component and TAGT/− for the minor, considering a possible drop-out of a second allele of the minor component; while analyst two assigned TAAT/− for the major and no haplotypes to the minor −/−; taking into account that it cannot be completely discounted that the TAGT haplotype was from the major component that was showing a high heterozygote imbalance. The most conservative approach–the one from analyst 2 in the example–was used for mixture component BGA inference analysis.

For mixture 1, with the most balanced ratio of 1:3, both the major and minor components resulted in partial profiles after deconvolution, reaching profile completeness percentages of 42.16 and 63.23% respectively. For mixtures 2 and 3, the higher imbalance of the components at ratios 1:5 and 1:7 allowed a full differentiation of the major component. The minor components of mixtures 2 and 3 reached a similar completeness level to that observed in mixture 1 of 42.16 and 43.63%, respectively, despite the fact that ancestry of the individuals contributing to these two mixtures are totally (mixture 2), or partially shared (EUR component in mixture 3). This is not unexpected as the panel was designed for identification purposes.

**FIGURE 5:**
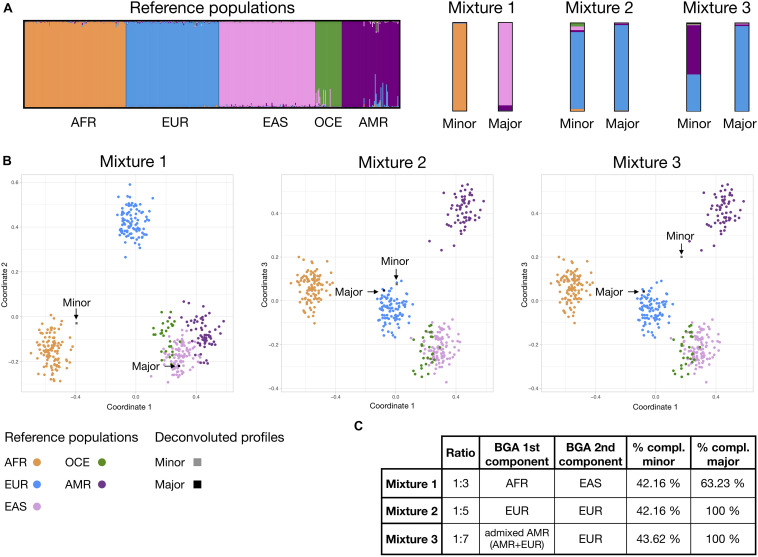
Bio-geographical ancestry inference for the major and minor mixture components in mixtures 1, 2, and 3; classified using the continental reference set presented on Figure 3. **(A)** STRUCTURE results of ancestry proportions at *K* = 5. Each bar represents an individual and is colored in segments whose lengths correspond to their genetic cluster membership coefficients in up to five inferred population groups. **(B)** Three dimensional MDS analysis showing coordinates 1 and 2 (for mixture 1) or 1 and 3 (for mixtures 2 and 3). Populations and major and minor components are colored according to the legend. **(C)** Table showing, for each mixture ratio, the expected ancestry of the known components and % of completeness (compl.) of the minor and major deconvoluted MH profiles. Details of the simulated profiles and deconvolution results can be found in Supplementary File S2 and Supplementary Table S8.

Figure 5 shows BGA results for the deconvoluted minor and major components of the mixtures. STRUCTURE analysis revealed the expected ancestry for all deconvoluted profiles. Moreover, estimated co-ancestry proportions of the EUR and AMR for the minor component reached similar levels to the complete profile of the admixed PEL component sample, with a 56.8 and a 55.7% of AMR component, respectively. For MDS, partial profiles from unadmixed samples tended to be spread more away from the reference population cluster, but consistently pointed to the expected ancestry. Admixed partial profile from minor component of mixture 3 appeared almost equidistant from the EUR and AMR clusters, inconsonance with expected.

## Discussion

In this study, designed to evaluate extended functionality of MH loci for mixed DNA analysis and compile the necessary population reference data for this purpose, a total of 240 reference HGDP-CEPH individuals of native populations from NAF, ME, OCE, and AMR were analyzed with the panel of 102 autosomal and 11 X-chromosome MHs. Most MHs (109/113) performed well in MPS tests, even when chips were loaded with ∼40 sample libraries. Moreover, 99% concordance was achieved between the MH alleles obtained through MPS and the SGDP phased data used for direct comparisons, while reaching 99.75% concordance with HGDP WGS data. The concordance study revealed some inconsistencies due to the probabilistic phasing algorithm used by both datasets, emphasizing the idea that the phase of the SNPs forming the haplotypes is more accurately derived when detected directly from sequence reads of individual strands, which will encompass all the SNPs in the MH in the same amplicon and using the pipeline developed for the forensic use of the panel. This pipeline outputs the depth coverage of each haplotype and produces profiles similar to those from STRs. Moreover, the pipeline allows for costumization of minimum allele frequency and minimum coverage parameters, analogous to the analytical and interpretation thresholds used in capillary electrophoresis analysis. These characteristics aid the interpretation of MH results by forensic experts, especially for mixture analysis, and enhances the utility of the MHs panel we have developed.

Despite the fact that some populations had limited numbers of samples, MHs showed similar degrees of polymorphism to those encountered in the extensive 1 KG dataset. This endorses the use of the panel for individual identification or kinship testing in the additional worldwide populations analyzed. For this purpose, one of the major advantages of the panel is the small size of the amplicons, that previously outperformed standard STR analysis when dealing with degraded DNA (De La Puente et al., 2019). Compared to SNaPshot (Sánchez et al., 2006; Freire-Aradas et al., 2012; Wang et al., 2016) or commercial MPS SNP panels (Precision ID Identity Panel from TFS, ForenSeq DNA Signature Prep Kit from Verogen) commonly used as supplementary kinship markers, or for degraded DNA analysis, the MHs panel offers a much higher discrimination power due to the increased levels of polymorphism of the markers, while maintaining sentitivity to low level DNA.

At the same time, the new population data we report is a valuable addition to BGA analyses using the panel. The results demonstrate the ability of the panel to differentiate the five major continental groups (AFR, EUR, EAS, OCE, and AMR) and, to a lesser extent, the main sets of populations within Eurasia (NAF, ME, EUR, SAS, EAS). Populations NAF, ME, and SAS are sited in the middle of variation clines and therefore their differentiation is challenging, especially for NAF and ME regions. To address such challenges, MPS capabilities support much bigger multiplex scales than a typical SNaPshot multiplex assay for SNP genotyping while mantaining forensic sensitivity, allowing a more fine scale geographic resolution in BGA analyses. The MHs panel takes advantage of the higher multiplex capabilities while of MPS using highly polymorphic markers giving high heterozygosity values within populations (allowing individual identification) and high between population differentation (allowing BGA inference). For these reasons, although not an original criterion for the selection of the component MHs of the panel, the degree of BGA information is similar or superior to that achieved with other custom (Eduardoff et al., 2016; Pereira et al., 2019; Phillips et al., 2019) or commercial MPS panels (Precision ID Ancestry Panel from TFS, ForenSeq DNA Signature Prep Kit from Verogen). The MHs panel considerably exceeds the capabilities of dedicated forensic SNaPshot assays for BGA in use before the advent of MPS (Phillips et al., 2007; Daca-Roszak et al., 2016; De La Puente et al., 2016).

Finally, in this study we began to explore the scope for BGA inference from deconvoluted mixed DNA contributors. Preliminary studies by Oldoni et al. (2017), based on likelihood ratios of profile likelihoods from each population indicated that it is feasible to deconvolute simple two-donor mixtures with skewed mixture ratios, by assigning haplotypes to a major and a minor component and then to infer their ancestry. Here, we confirmed this form of analysis is effective, because it can take advantage of the fact that both the MDS and STRUCTURE methodologies can handle partial profiles. However, extra caution must be used when inferring ancestry for investigative leads when the inferences are made from profiles with high levels of incompleteness. Despite profile deconvolution being both laborious and error-prone, in the near future it is likely that probabilistic genotyping software will be adapted for BGA inference purposes.

Deconvolution of mixed MH profiles is simplified by the abscense of stutter artifacts and probabilistic genotyping software can be readily adapted and used for individual identification of the mixture contributors. The ability of the panel to identify the contributors is supported by the fact that, assuming a similar level of informativeness for all MHs [and as shown by the consistent gradient of the RMP slope from Figure 4 in De La Puente et al. (2019)], a ∼60% locus completeness of the panel (comparable to the completeness levels shown for mixture 1 deconvolution of the major component) reaches a mean cumulative power of discrimination value across all populations (data from Figure 2) of ∼E-39 while a ∼40% completeness of the panel (comparable to the minor component) reaches levels of ∼E-30 (i.e., comparable to 21 autosomal STRs using GlobalFiler).

## Conclusion

The MHs panel we have previously developed is found to be even more of a multi-purpose tool for forensic applications than originally proposed. It is applicable in those forensic cases in which regular STR analysis by itself does not provide an answer or supplementary information is needed. The same component loci of the MHs panel prove to be highly informative for: individual identification with a focus on highly degraded DNA, especially since all amplicon sizes are less than 175 bp; kinship testing; mixed DNA analysis and BGA inference–with indications from our studies that the latter two functions can be combined in simple mixtures. With this in mind, the panel could help to improve identifications in disaster victim identification programs that involve multiple nationalities, where BGA can assist in the first triage of the victims and the selection of the correct allele frequencies for identification through comparisons to surviving relatives. The panel has been fully validated for forensic purposes and can be implemented with both the two main MPS platforms in common use in forensic laboratories: MiSeq and Ion S5, with the latter allowing automated library construction.

## Data Availability Statement

The data generated in this manuscript has been deposited at the following public repositories:

– raw reads as fastq were submitted to the European Nucleotide Archive (ENA) under accession number PRJEB39413
– vcf files from Torrent Suite were submitted to the European Variation Archive (EVA) under accession number PRJEB39574.

## Author Contributions

All authors listed made substantial and direct contributions to the work and approved it for publication. MVL, WP, CP, ÁC, and MdlP designed the study, developed the ideas and obtained funding for the project. JR-R and AA-C conducted the DNA analysis. MdlP, CP, CX, JA, MÁCdC, and AG-T analyzed the results. MdlP and CP wrote the manuscript. All authors discussed the results and contributed to the revision of the manuscript.

## Conflict of Interest

The authors declare that the research was conducted in the absence of any commercial or financial relationships that could be construed as a potential conflict of interest.
