# Supplementary material for: Broadening the Applicability of a Custom Multi-Platform Panel of Microhaplotypes: Bio-Geographical Ancestry Inference and Expanded Reference Data
Source: Front Genet. 2020 Oct 20;11:581041. doi: 10.3389/fgene.2020.581041 (PMC7606911; doi:10.3389/fgene.2020.581041)
Supplement: Supplementary file 1 [file Data_Sheet_1.PDF]

**Supplementary File S1.** Bar charts showing haplotype frequency data for the 30 populations considered in this study.

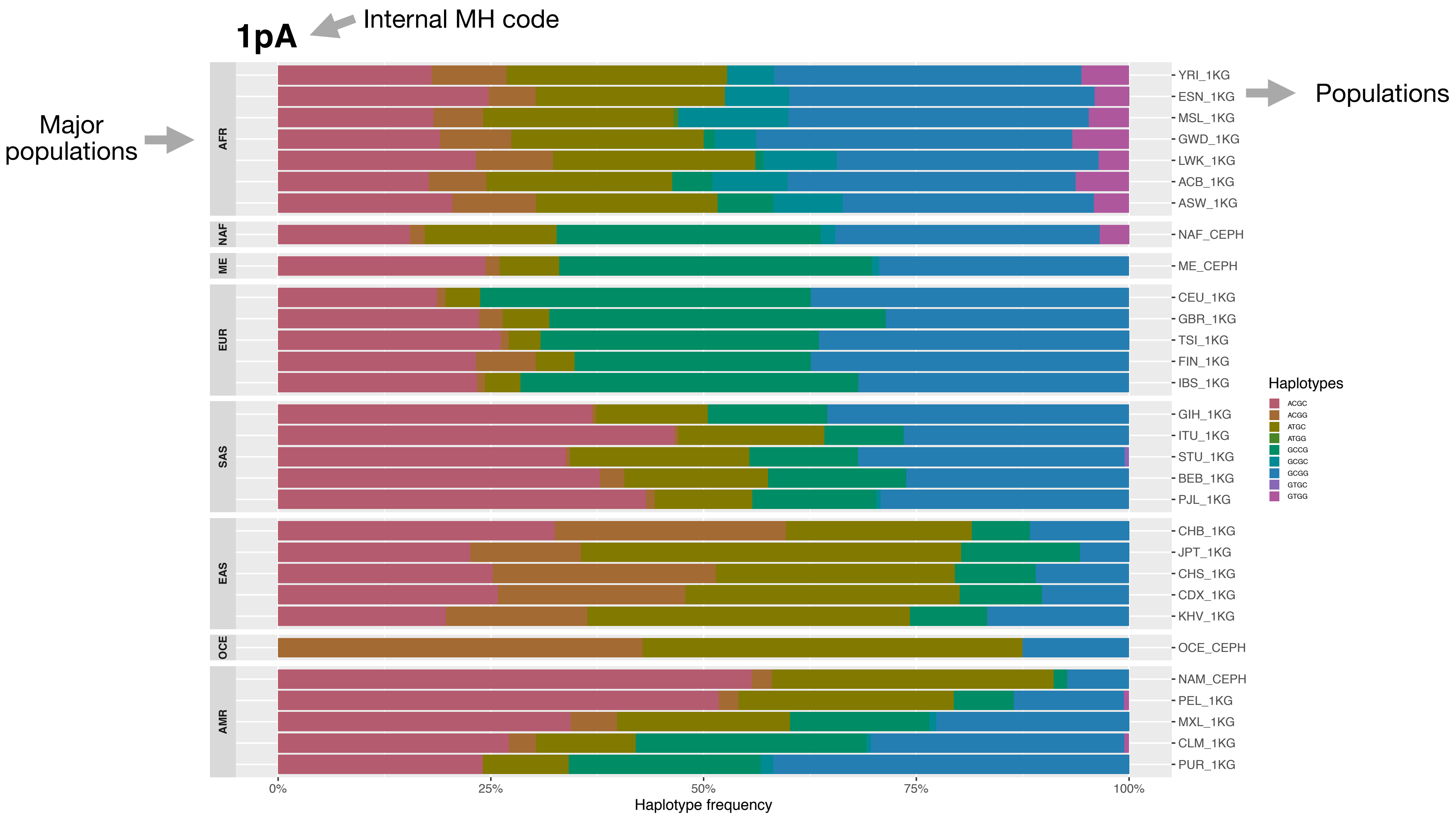

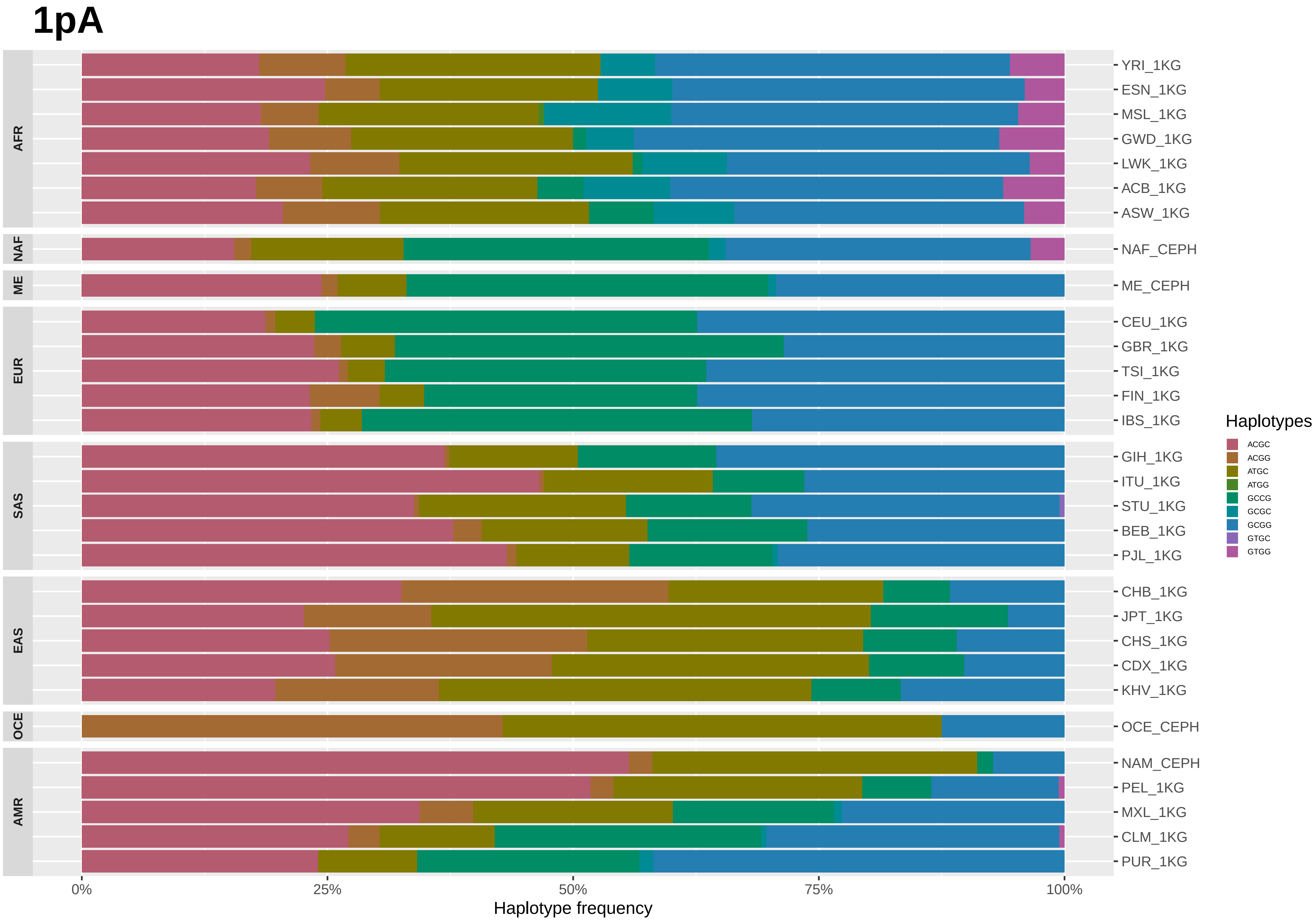
1pA
AFR
NAF
ME
EUR
SAS
EAS
OCE
AMR
YRI_1KG
ESN_1KG
MSL_1KG
GWD_1KG
LWK_1KG
ACB_1KG
ASW_1KG
NAF_CEPH
ME_CEPH
CEU_1KG
GBR_1KG
TSI_1KG
FIN_1KG
IBS_1KG
GIH_1KG
ITU_1KG
STU_1KG
BEB_1KG
PJL_1KG
CHB_1KG
JPT_1KG
CHS_1KG
CDX_1KG
KHV_1KG
OCE_CEPH
NAM_CEPH
PEL_1KG
MXL_1KG
CLM_1KG
PUR_1KG
0%
25%
50%
75%
100%
Haplotype frequency
Haplotypes
ACGC
ACGG
ATGC
ATGG
GCCG
GCGC
GCGG
GTGC
GTGG

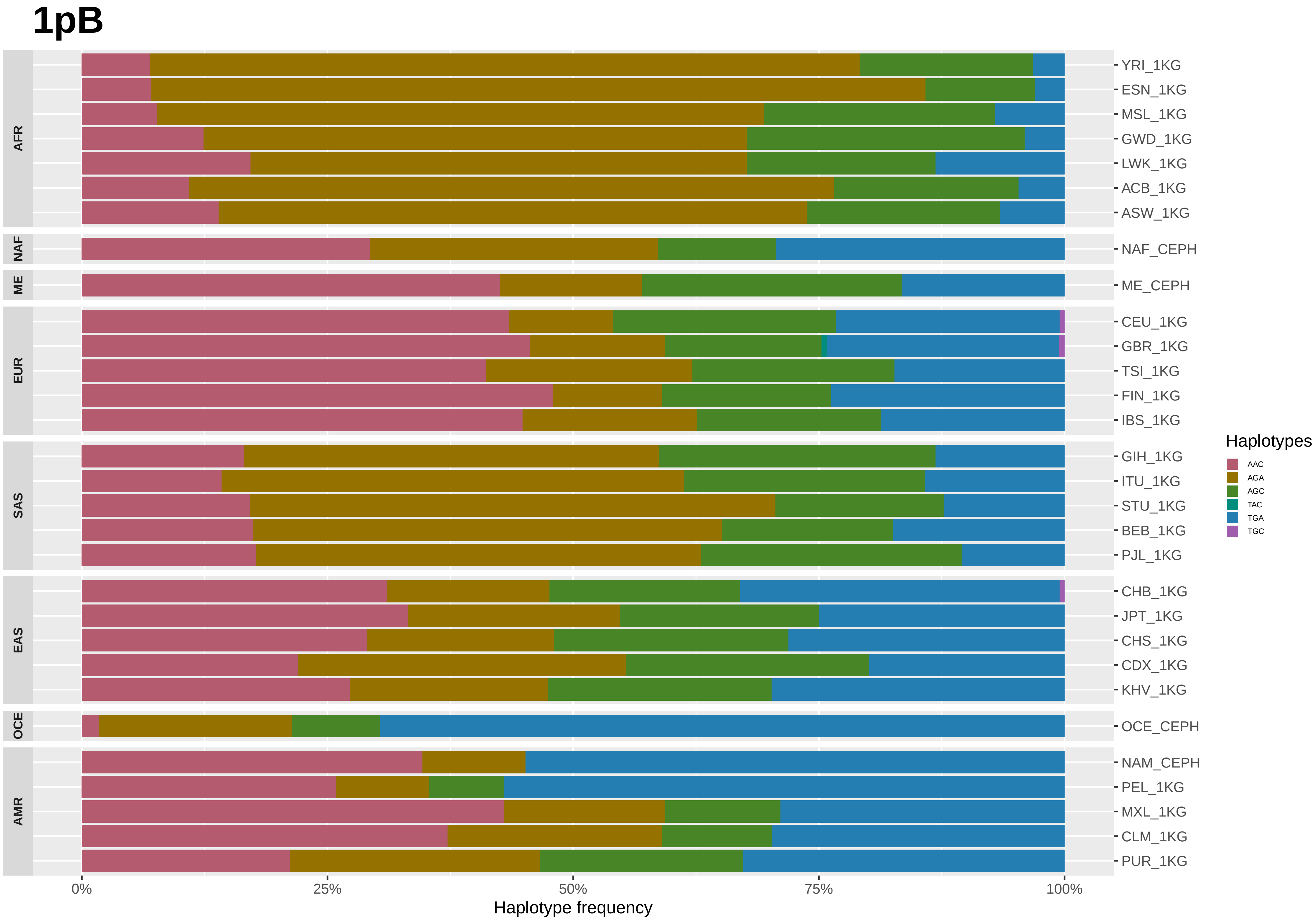
1pB
AFR
NAF
ME
EUR
SAS
EAS
OCE
AMR
YRI_1KG
ESN_1KG
MSL_1KG
GWD_1KG
LWK_1KG
ACB_1KG
ASW_1KG
NAF_CEPH
ME_CEPH
CEU_1KG
GBR_1KG
TSI_1KG
FIN_1KG
IBS_1KG
GIH_1KG
ITU_1KG
STU_1KG
BEB_1KG
PJL_1KG
CHB_1KG
JPT_1KG
CHS_1KG
CDX_1KG
KHV_1KG
OCE_CEPH
NAM_CEPH
PEL_1KG
MXL_1KG
CLM_1KG
PUR_1KG
0%
25%
50%
75%
100%
Haplotype frequency
Haplotypes
AAC
AGA
AGC
TAC
TGA
TGC

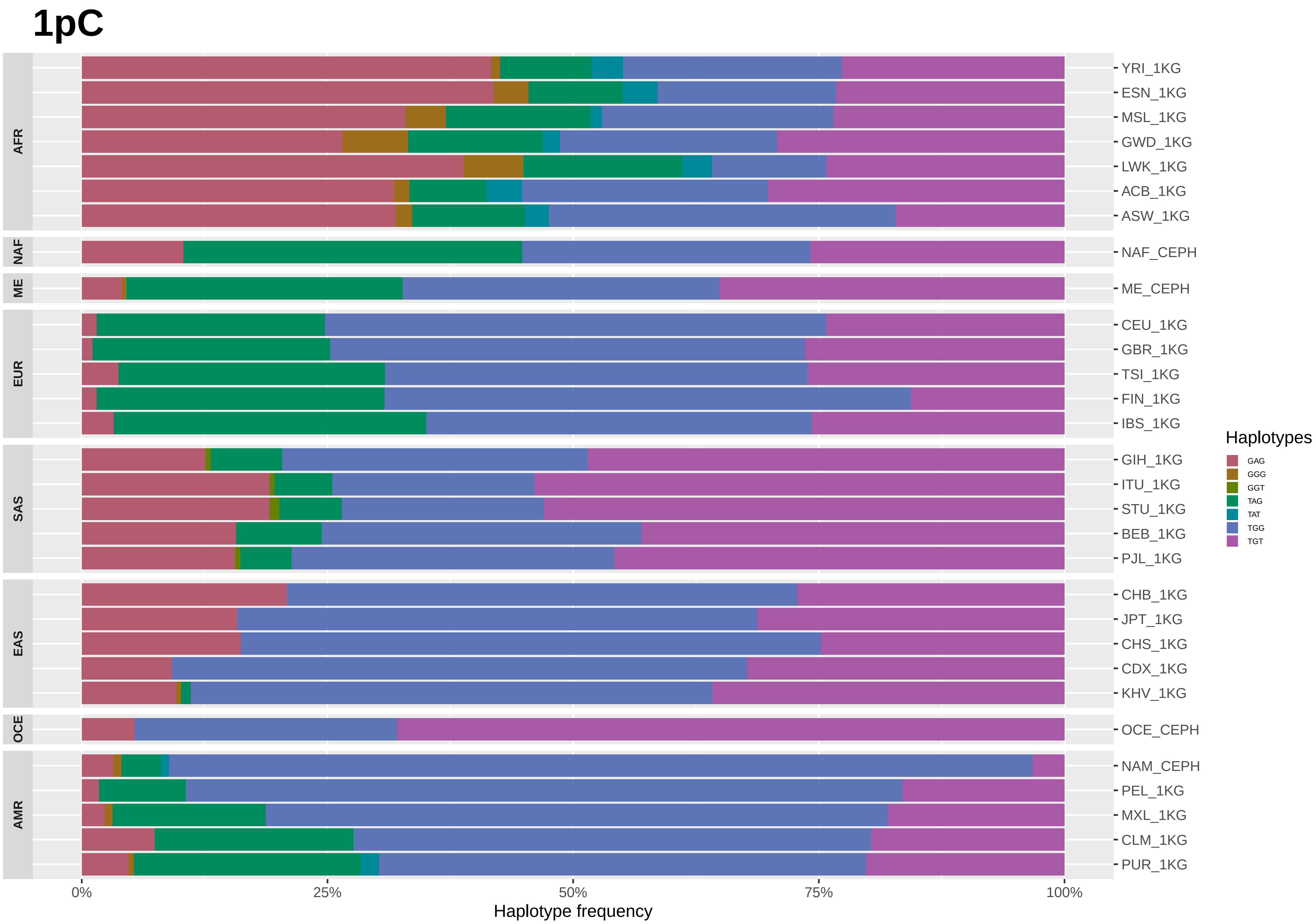

1pC
AFR
NAF
ME
EUR
SAS
EAS
OCE
AMR
YRI_1KG
ESN_1KG
MSL_1KG
GWD_1KG
LWK_1KG
ACB_1KG
ASW_1KG
NAF_CEPH
ME_CEPH
CEU_1KG
GBR_1KG
TSI_1KG
FIN_1KG
IBS_1KG
GIH_1KG
ITU_1KG
STU_1KG
BEB_1KG
PJL_1KG
CHB_1KG
JPT_1KG
CHS_1KG
CDX_1KG
KHV_1KG
OCE_CEPH
NAM_CEPH
PEL_1KG
MXL_1KG
CLM_1KG
PUR_1KG
0%
25%
50%
75%
100%
Haplotype frequency
Haplotypes
GAG
GGG
GGT
TAG
TAT
TGG
TGT

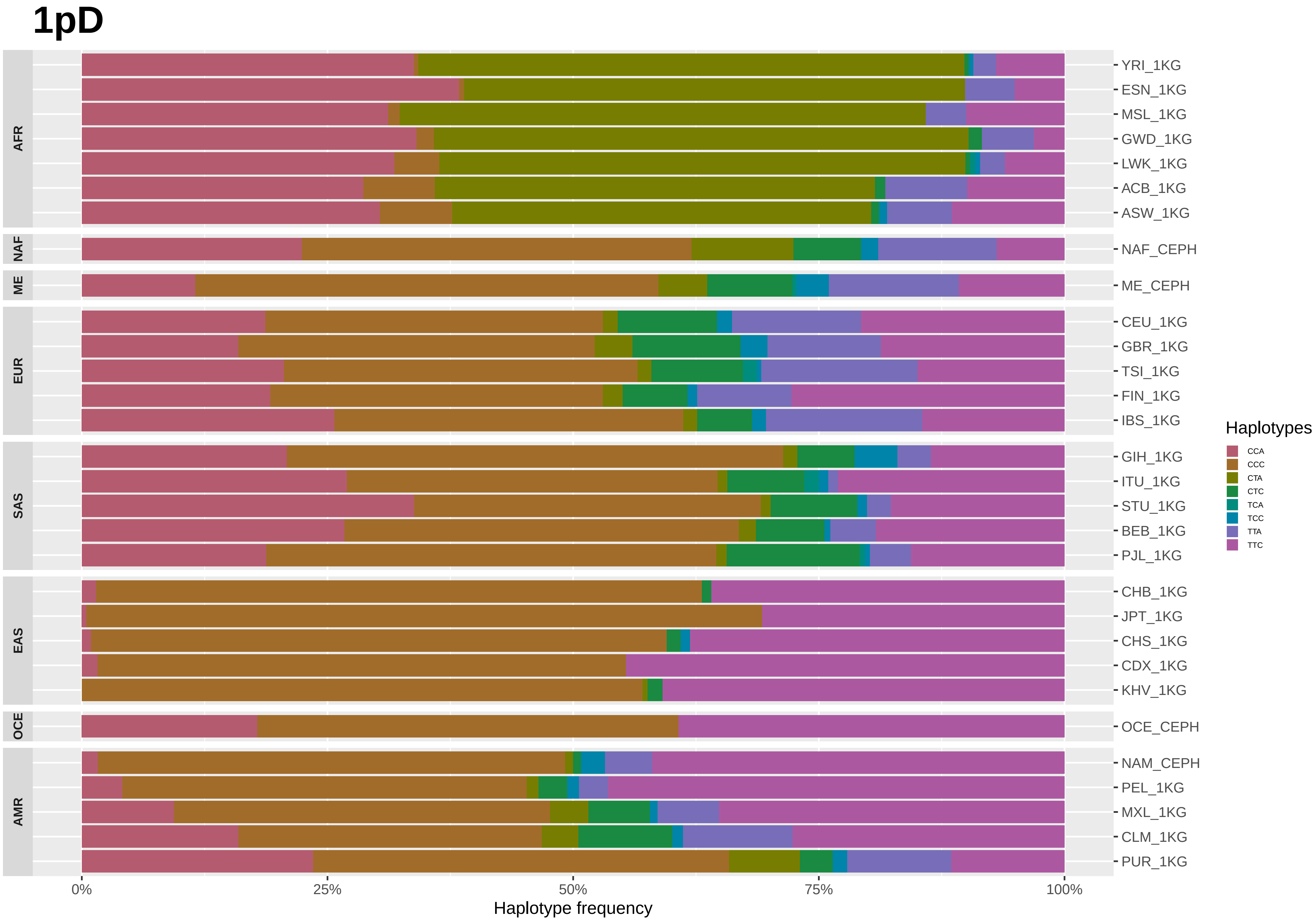

1pD
AFR
NAF
ME
EUR
SAS
EAS
OCE
AMR
YRI_1KG
ESN_1KG
MSL_1KG
GWD_1KG
LWK_1KG
ACB_1KG
ASW_1KG
NAF_CEPH
ME_CEPH
CEU_1KG
GBR_1KG
TSI_1KG
FIN_1KG
IBS_1KG
GIH_1KG
ITU_1KG
STU_1KG
BEB_1KG
PJL_1KG
CHB_1KG
JPT_1KG
CHS_1KG
CDX_1KG
KHV_1KG
OCE_CEPH
NAM_CEPH
PEL_1KG
MXL_1KG
CLM_1KG
PUR_1KG
0%
25%
50%
75%
100%
Haplotype frequency
Haplotypes
CCA
CCC
CTA
CTC
TCA
TCC
TTA
TTC

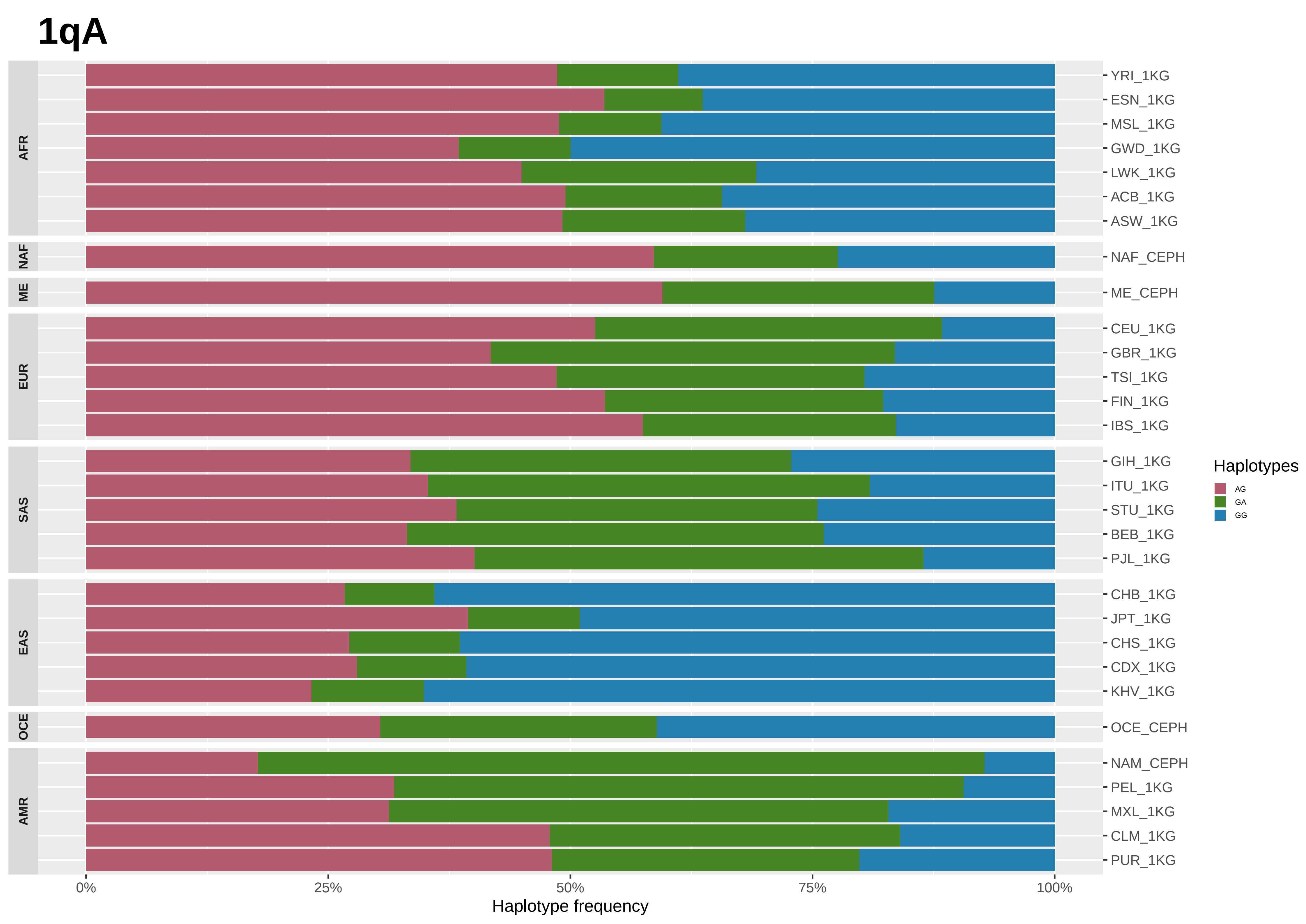

1qA
AFR
NAF
ME
EUR
SAS
EAS
OCE
AMR
YRI_1KG
ESN_1KG
MSL_1KG
GWD_1KG
LWK_1KG
ACB_1KG
ASW_1KG
NAF_CEPH
ME_CEPH
CEU_1KG
GBR_1KG
TSI_1KG
FIN_1KG
IBS_1KG
GIH_1KG
ITU_1KG
STU_1KG
BEB_1KG
PJL_1KG
CHB_1KG
JPT_1KG
CHS_1KG
CDX_1KG
KHV_1KG
OCE_CEPH
NAM_CEPH
PEL_1KG
MXL_1KG
CLM_1KG
PUR_1KG
0%
25%
50%
75%
100%
Haplotype frequency
Haplotypes
AG
GA
GG

# 1qB

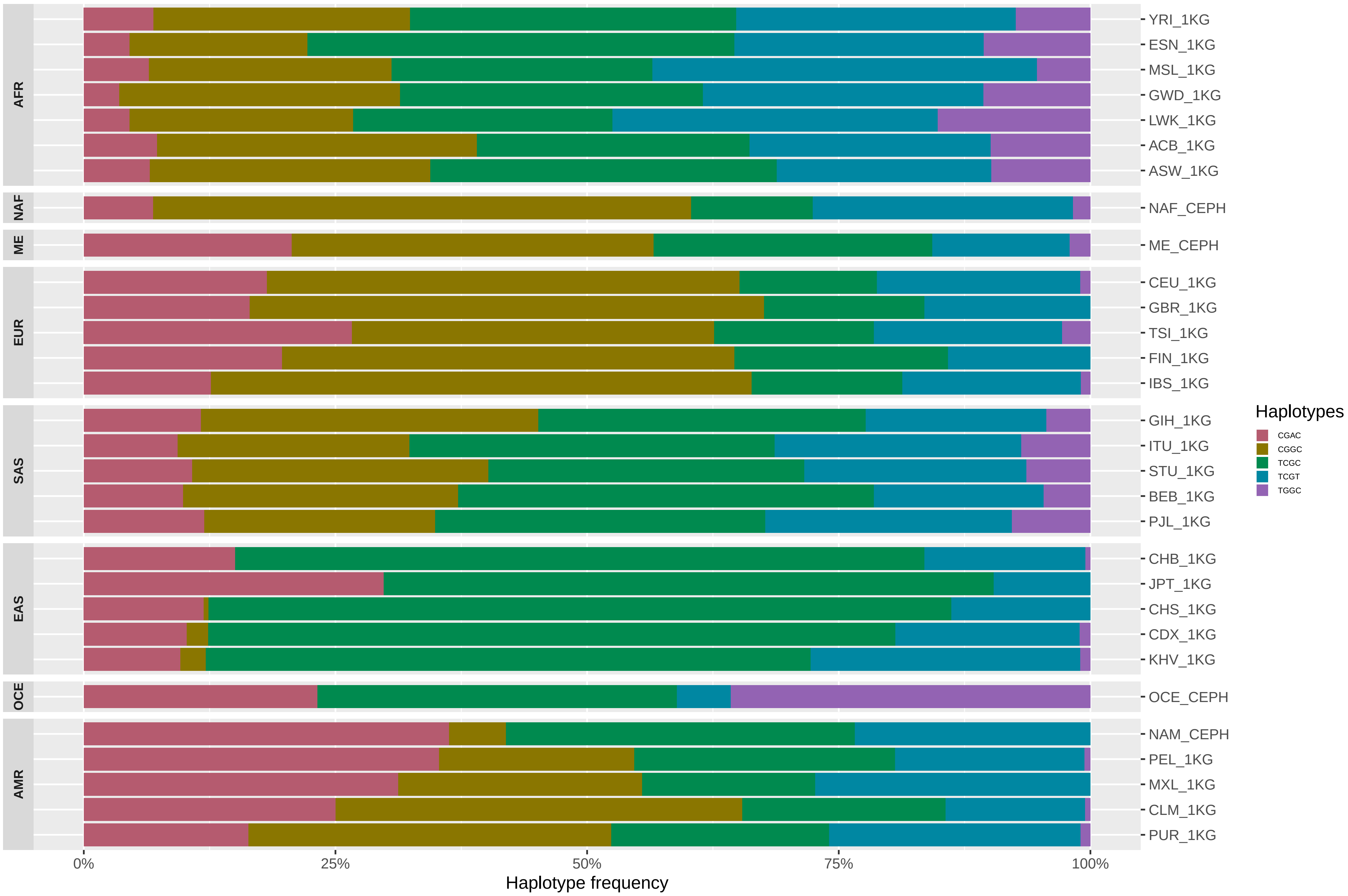

AFR
NAF
ME
EUR
SAS
EAS
OCE
AMR
YRI_1KG
ESN_1KG
MSL_1KG
GWD_1KG
LWK_1KG
ACB_1KG
ASW_1KG
NAF_CEPH
ME_CEPH
CEU_1KG
GBR_1KG
TSI_1KG
FIN_1KG
IBS_1KG
GIH_1KG
ITU_1KG
STU_1KG
BEB_1KG
PJL_1KG
CHB_1KG
JPT_1KG
CHS_1KG
CDX_1KG
KHV_1KG
OCE_CEPH
NAM_CEPH
PEL_1KG
MXL_1KG
CLM_1KG
PUR_1KG
0%
25%
50%
75%
100%
Haplotype frequency
Haplotypes
CGAC
CGGC
TCGC
TCGT
TGGC

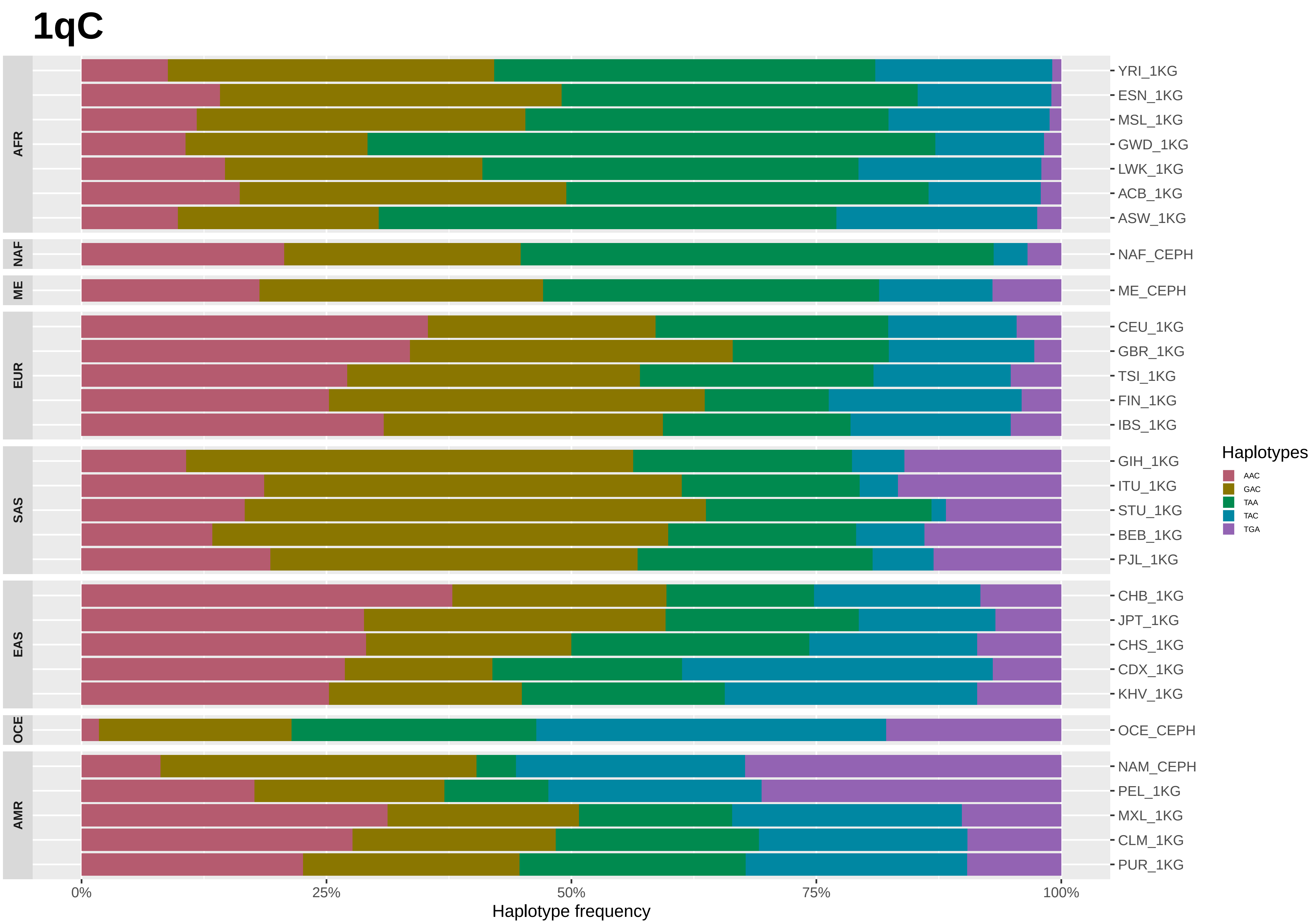

1qC
AFR
NAF
ME
EUR
SAS
EAS
OCE
AMR
YRI_1KG
ESN_1KG
MSL_1KG
GWD_1KG
LWK_1KG
ACB_1KG
ASW_1KG
NAF_CEPH
ME_CEPH
CEU_1KG
GBR_1KG
TSI_1KG
FIN_1KG
IBS_1KG
GIH_1KG
ITU_1KG
STU_1KG
BEB_1KG
PJL_1KG
CHB_1KG
JPT_1KG
CHS_1KG
CDX_1KG
KHV_1KG
OCE_CEPH
NAM_CEPH
PEL_1KG
MXL_1KG
CLM_1KG
PUR_1KG
0%
25%
50%
75%
100%
Haplotype frequency
Haplotypes
AAC
GAC
TAA
TAC
TGA

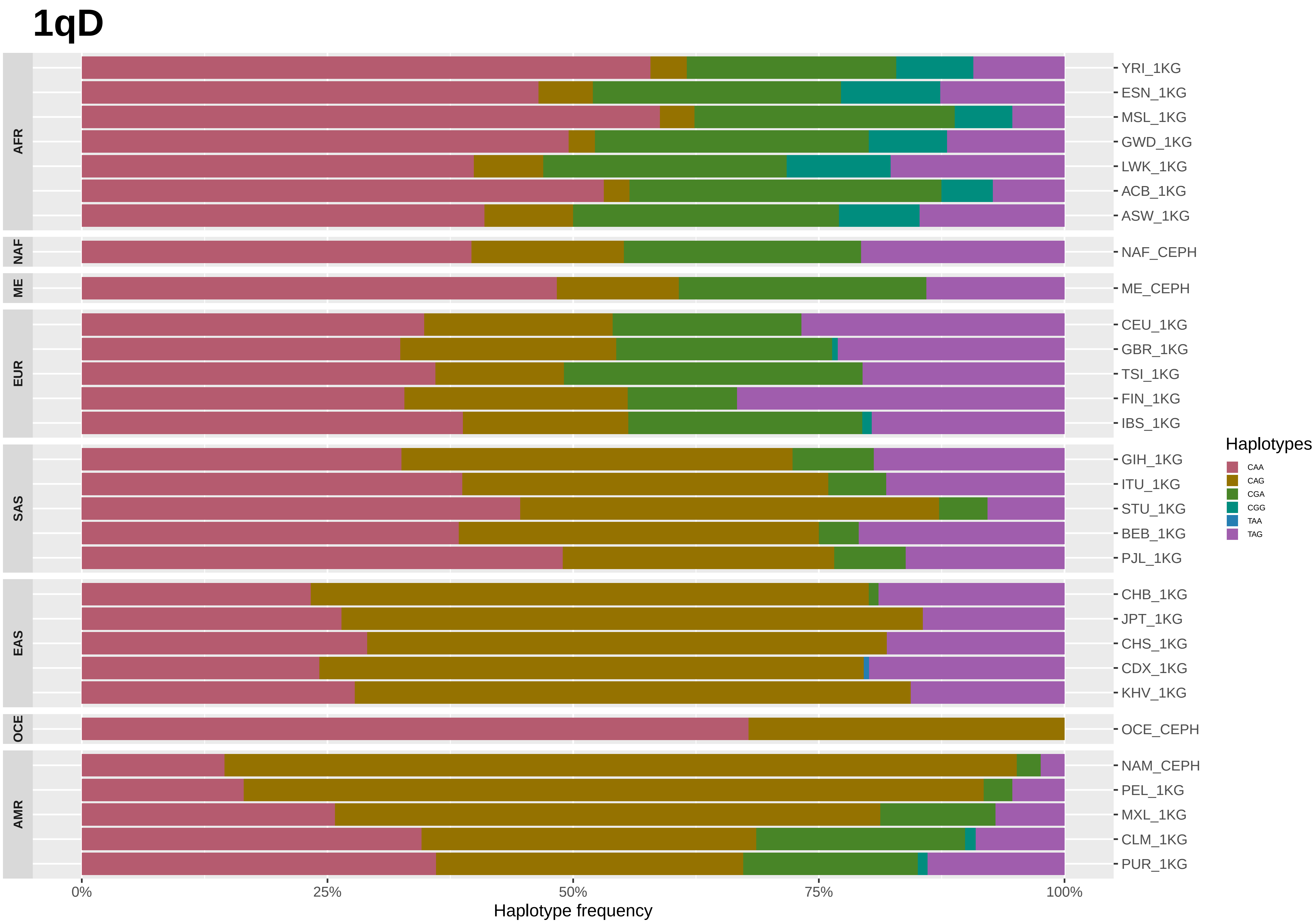
1qD
AFR
NAF
ME
EUR
SAS
EAS
OCE
AMR
YRI_1KG
ESN_1KG
MSL_1KG
GWD_1KG
LWK_1KG
ACB_1KG
ASW_1KG
NAF_CEPH
ME_CEPH
CEU_1KG
GBR_1KG
TSI_1KG
FIN_1KG
IBS_1KG
GIH_1KG
ITU_1KG
STU_1KG
BEB_1KG
PJL_1KG
CHB_1KG
JPT_1KG
CHS_1KG
CDX_1KG
KHV_1KG
OCE_CEPH
NAM_CEPH
PEL_1KG
MXL_1KG
CLM_1KG
PUR_1KG
0%
25%
50%
75%
100%
Haplotype frequency
Haplotypes
CAA
CAG
CGA
CGG
TAA
TAG

# 2pA

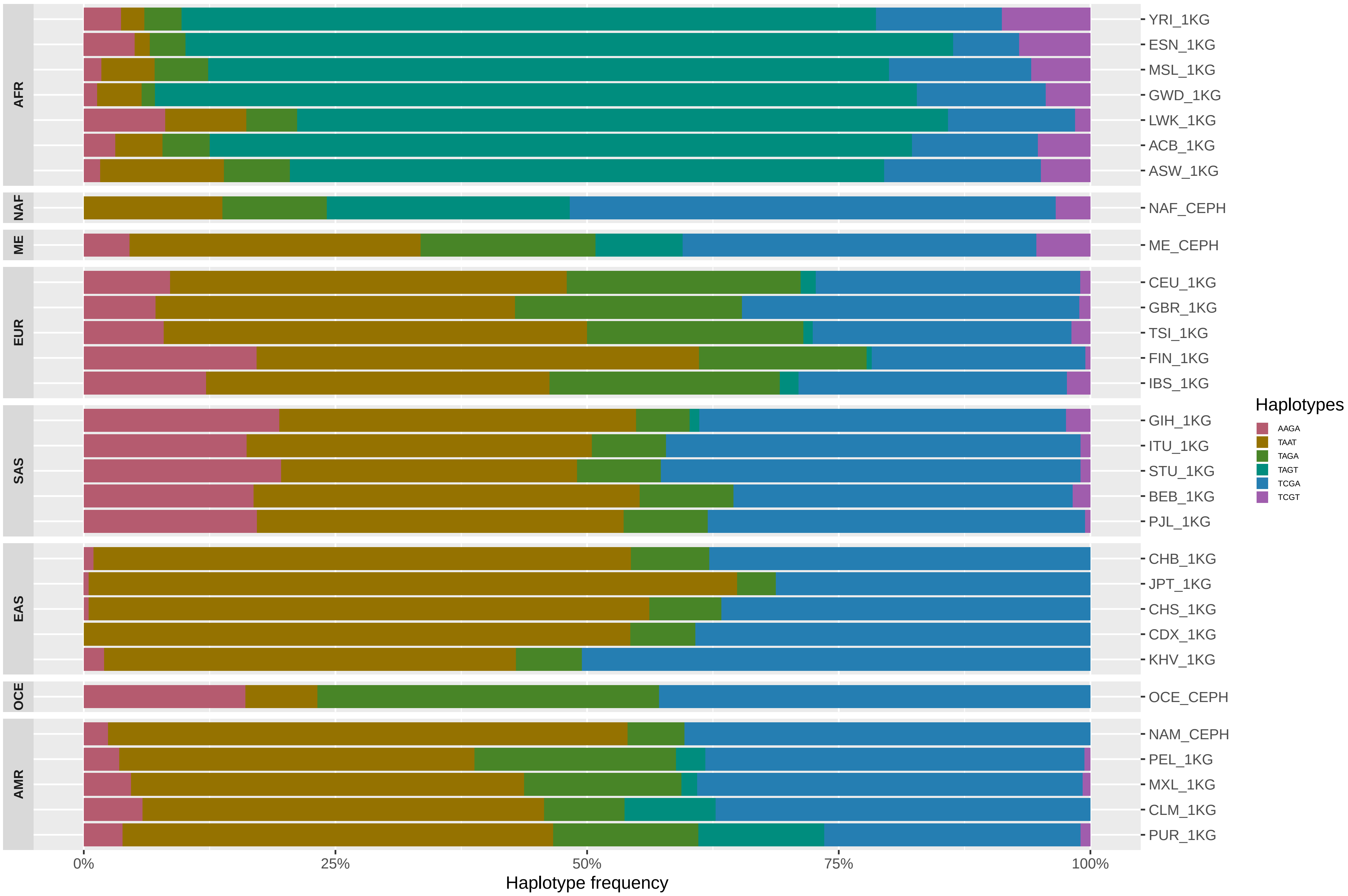

AFR
NAF
ME
EUR
SAS
EAS
OCE
AMR
YRI_1KG
ESN_1KG
MSL_1KG
GWD_1KG
LWK_1KG
ACB_1KG
ASW_1KG
NAF_CEPH
ME_CEPH
CEU_1KG
GBR_1KG
TSI_1KG
FIN_1KG
IBS_1KG
GIH_1KG
ITU_1KG
STU_1KG
BEB_1KG
PJL_1KG
CHB_1KG
JPT_1KG
CHS_1KG
CDX_1KG
KHV_1KG
OCE_CEPH
NAM_CEPH
PEL_1KG
MXL_1KG
CLM_1KG
PUR_1KG
0%
25%
50%
75%
100%
Haplotype frequency
Haplotypes
AAGA
TAAT
TAGA
TAGT
TCGA
TCGT

# 2pB

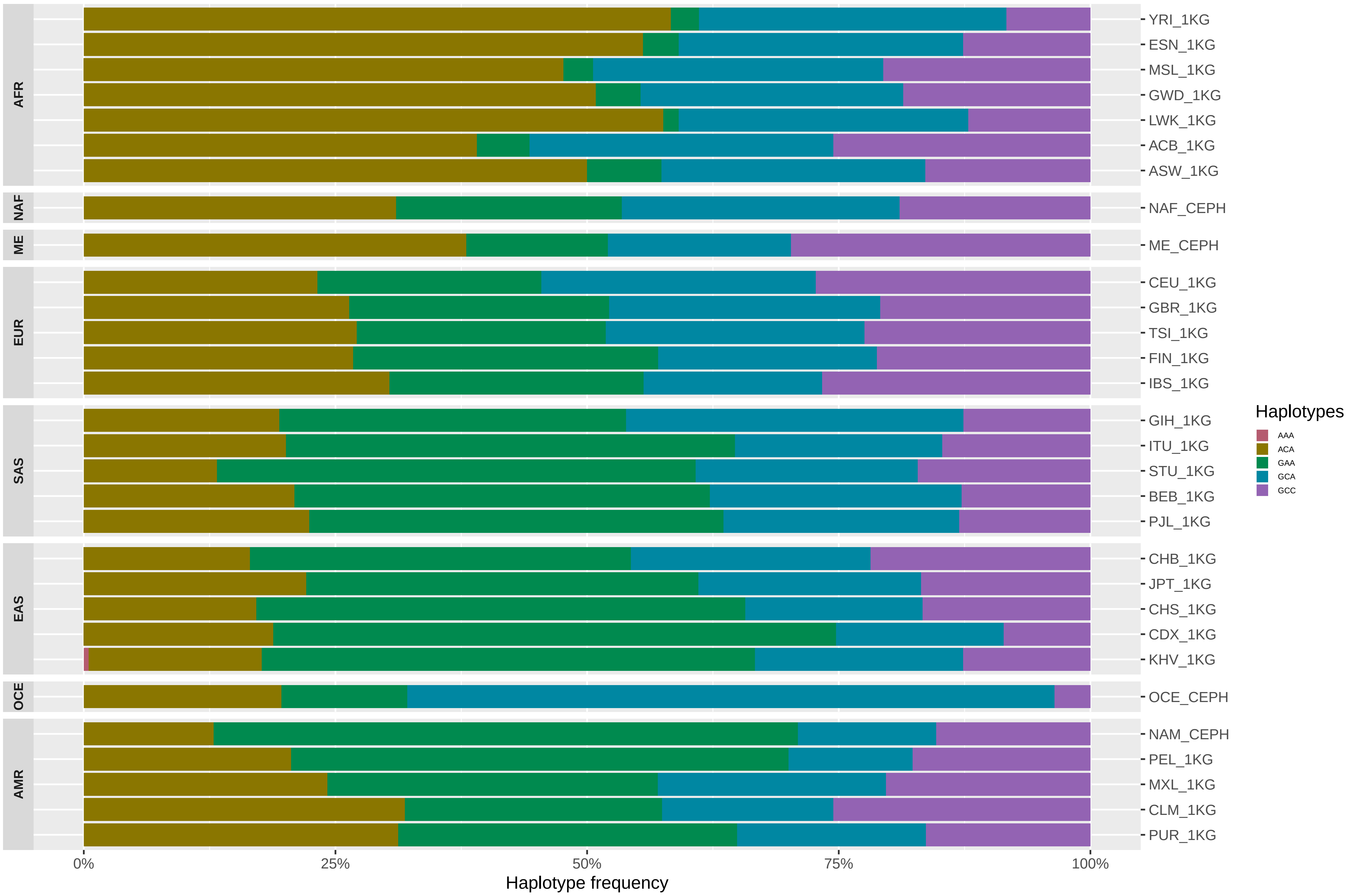
AFR
NAF
ME
EUR
SAS
EAS
OCE
AMR
YRI_1KG
ESN_1KG
MSL_1KG
GWD_1KG
LWK_1KG
ACB_1KG
ASW_1KG
NAF_CEPH
ME_CEPH
CEU_1KG
GBR_1KG
TSI_1KG
FIN_1KG
IBS_1KG
GIH_1KG
ITU_1KG
STU_1KG
BEB_1KG
PJL_1KG
CHB_1KG
JPT_1KG
CHS_1KG
CDX_1KG
KHV_1KG
OCE_CEPH
NAM_CEPH
PEL_1KG
MXL_1KG
CLM_1KG
PUR_1KG
0%
25%
50%
75%
100%
Haplotype frequency
Haplotypes
AAA
ACA
GAA
GCA
GCC

# 2pC

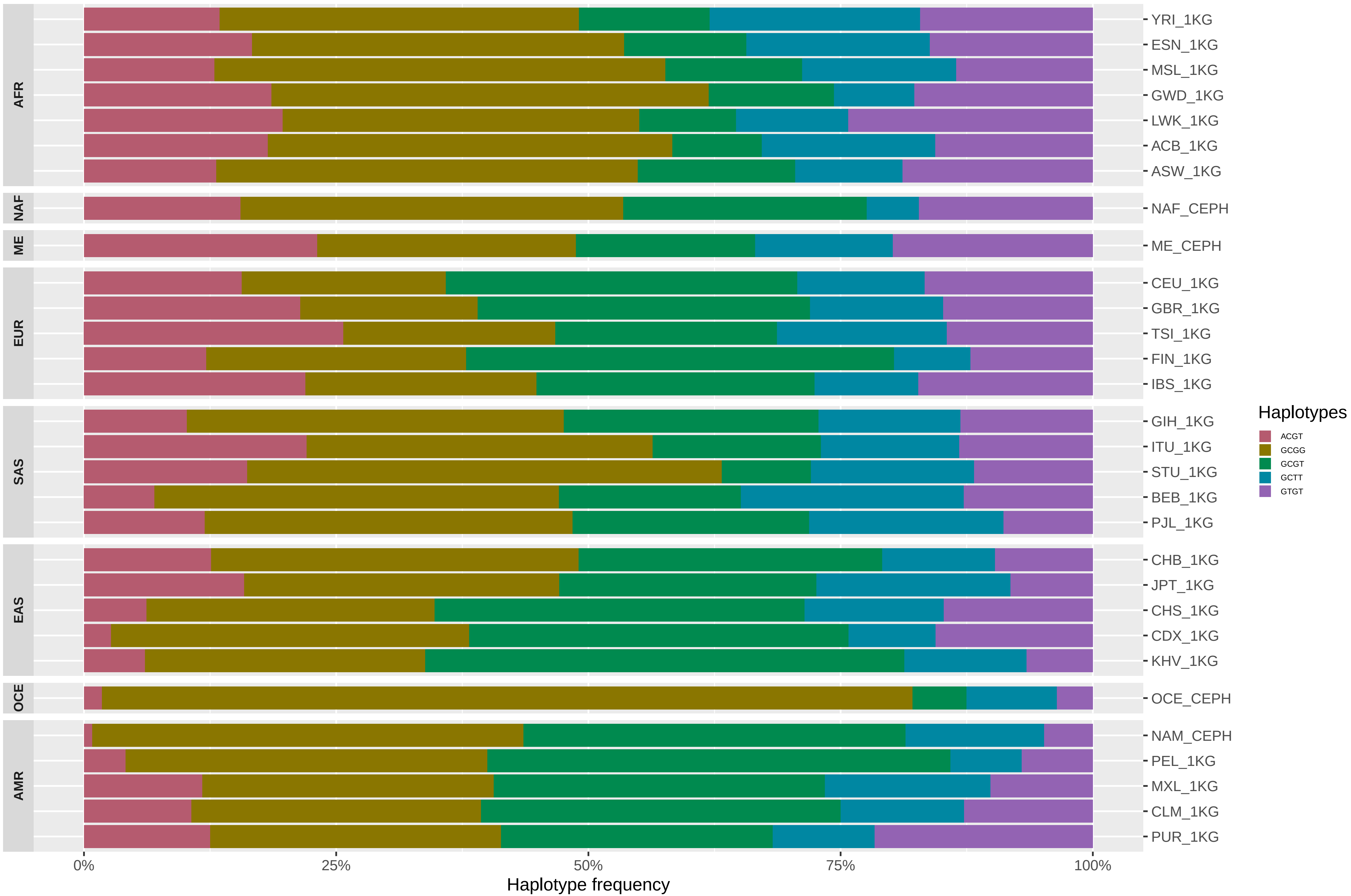
AFR
NAF
ME
EUR
SAS
EAS
OCE
AMR
YRI_1KG
ESN_1KG
MSL_1KG
GWD_1KG
LWK_1KG
ACB_1KG
ASW_1KG
NAF_CEPH
ME_CEPH
CEU_1KG
GBR_1KG
TSI_1KG
FIN_1KG
IBS_1KG
GIH_1KG
ITU_1KG
STU_1KG
BEB_1KG
PJL_1KG
CHB_1KG
JPT_1KG
CHS_1KG
CDX_1KG
KHV_1KG
OCE_CEPH
NAM_CEPH
PEL_1KG
MXL_1KG
CLM_1KG
PUR_1KG
0%
25%
50%
75%
100%
Haplotype frequency
Haplotypes
ACGT
GCGG
GCGT
GCTT
GTGT

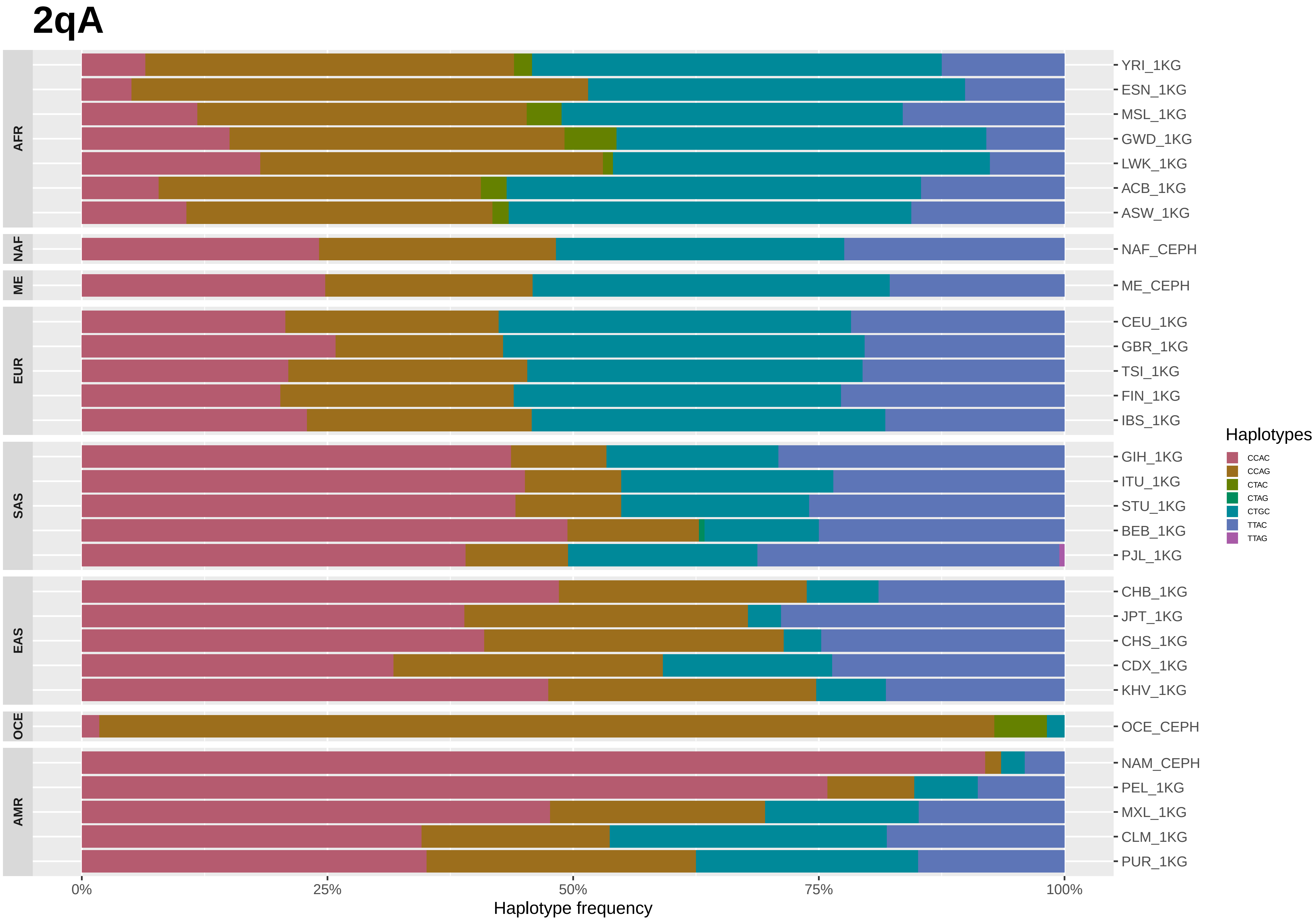

2qA
AFR
NAF
ME
EUR
SAS
EAS
OCE
AMR
YRI_1KG
ESN_1KG
MSL_1KG
GWD_1KG
LWK_1KG
ACB_1KG
ASW_1KG
NAF_CEPH
ME_CEPH
CEU_1KG
GBR_1KG
TSI_1KG
FIN_1KG
IBS_1KG
GIH_1KG
ITU_1KG
STU_1KG
BEB_1KG
PJL_1KG
CHB_1KG
JPT_1KG
CHS_1KG
CDX_1KG
KHV_1KG
OCE_CEPH
NAM_CEPH
PEL_1KG
MXL_1KG
CLM_1KG
PUR_1KG
0%
25%
50%
75%
100%
Haplotype frequency
Haplotypes
CCAC
CCAG
CTAC
CTAG
CTGC
TTAC
TTAG

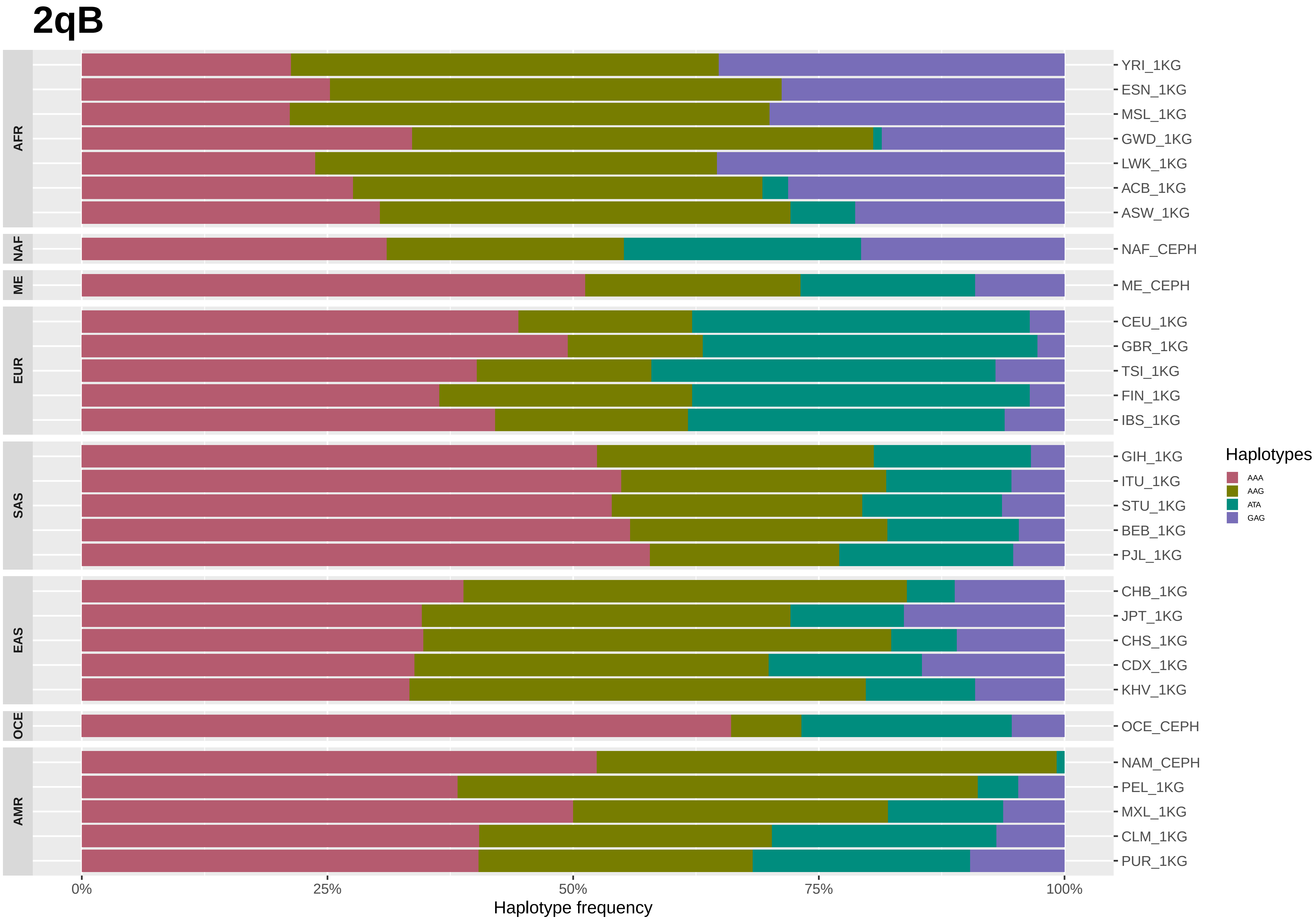

2qB
AFR
NAF
ME
EUR
SAS
EAS
OCE
AMR
YRI_1KG
ESN_1KG
MSL_1KG
GWD_1KG
LWK_1KG
ACB_1KG
ASW_1KG
NAF_CEPH
ME_CEPH
CEU_1KG
GBR_1KG
TSI_1KG
FIN_1KG
IBS_1KG
GIH_1KG
ITU_1KG
STU_1KG
BEB_1KG
PJL_1KG
CHB_1KG
JPT_1KG
CHS_1KG
CDX_1KG
KHV_1KG
OCE_CEPH
NAM_CEPH
PEL_1KG
MXL_1KG
CLM_1KG
PUR_1KG
0%
25%
50%
75%
100%
Haplotype frequency
Haplotypes
AAA
AAG
ATA
GAG

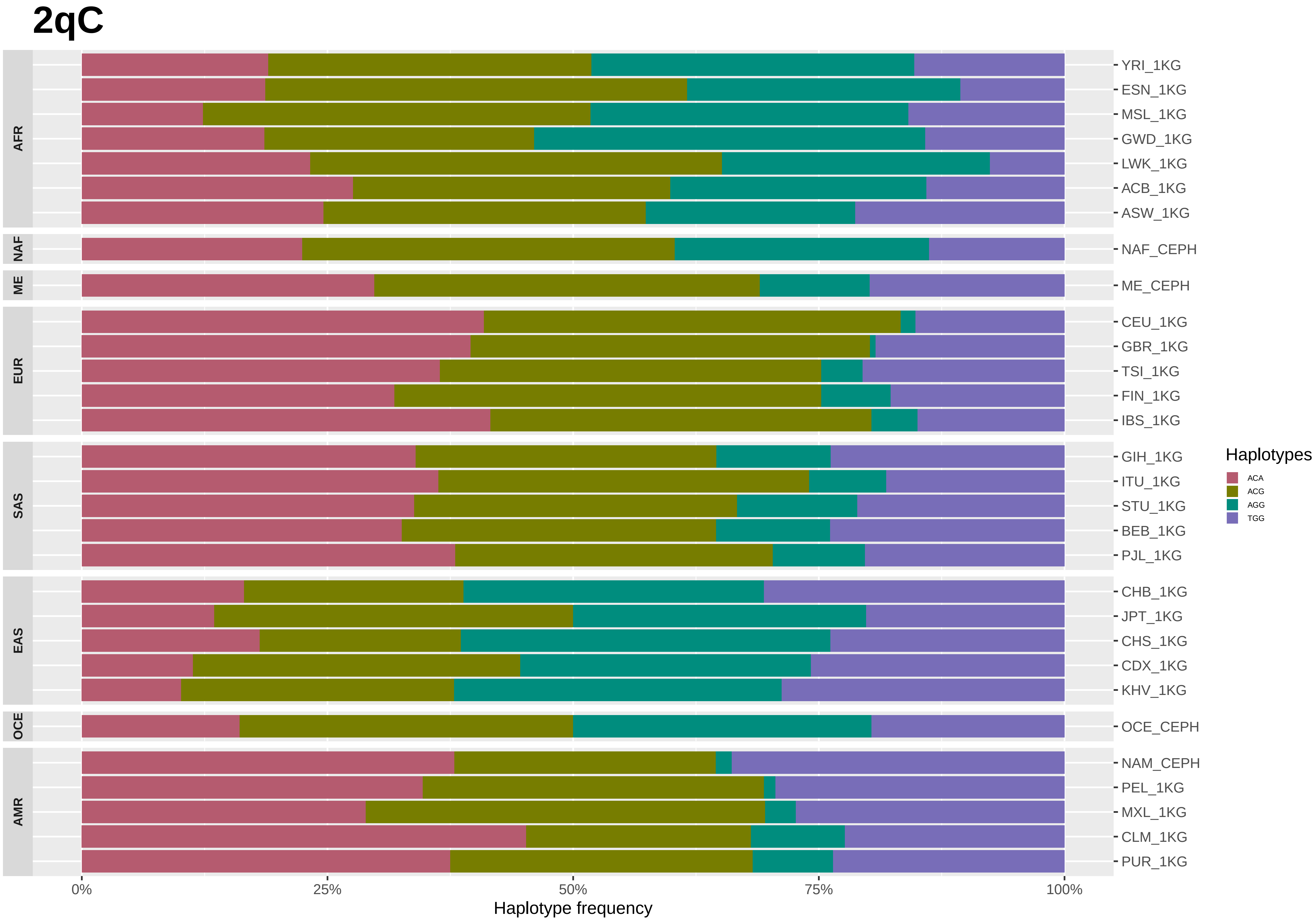
2qC
AFR
NAF
ME
EUR
SAS
EAS
OCE
AMR
YRI_1KG
ESN_1KG
MSL_1KG
GWD_1KG
LWK_1KG
ACB_1KG
ASW_1KG
NAF_CEPH
ME_CEPH
CEU_1KG
GBR_1KG
TSI_1KG
FIN_1KG
IBS_1KG
GIH_1KG
ITU_1KG
STU_1KG
BEB_1KG
PJL_1KG
CHB_1KG
JPT_1KG
CHS_1KG
CDX_1KG
KHV_1KG
OCE_CEPH
NAM_CEPH
PEL_1KG
MXL_1KG
CLM_1KG
PUR_1KG
0%
25%
50%
75%
100%
Haplotype frequency
Haplotypes
ACA
ACG
AGG
TGG

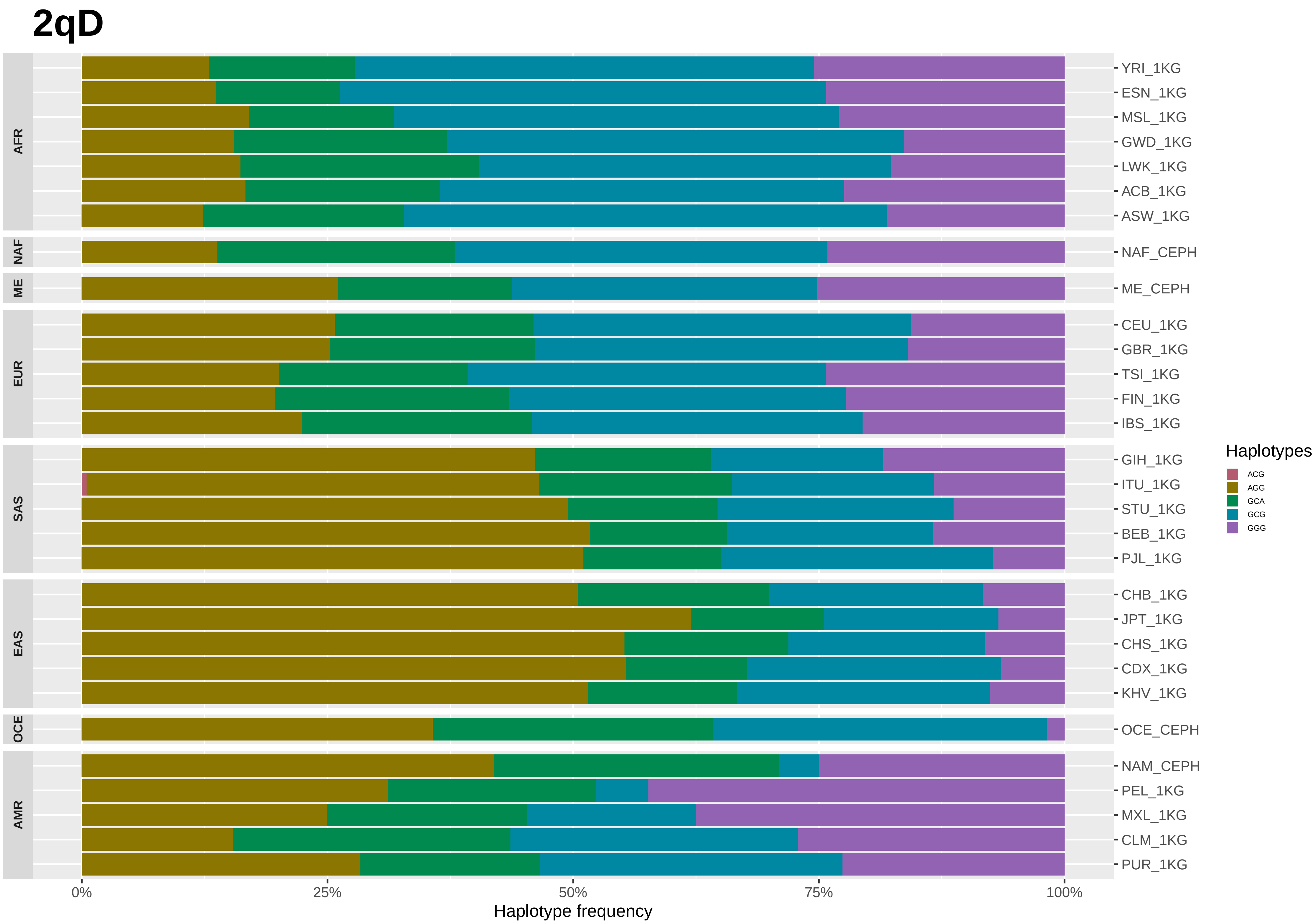
2qD
AFR
NAF
ME
EUR
SAS
EAS
OCE
AMR
YRI_1KG
ESN_1KG
MSL_1KG
GWD_1KG
LWK_1KG
ACB_1KG
ASW_1KG
NAF_CEPH
ME_CEPH
CEU_1KG
GBR_1KG
TSI_1KG
FIN_1KG
IBS_1KG
GIH_1KG
ITU_1KG
STU_1KG
BEB_1KG
PJL_1KG
CHB_1KG
JPT_1KG
CHS_1KG
CDX_1KG
KHV_1KG
OCE_CEPH
NAM_CEPH
PEL_1KG
MXL_1KG
CLM_1KG
PUR_1KG
0%
25%
50%
75%
100%
Haplotype frequency
Haplotypes
ACG
AGG
GCA
GCG
GGG

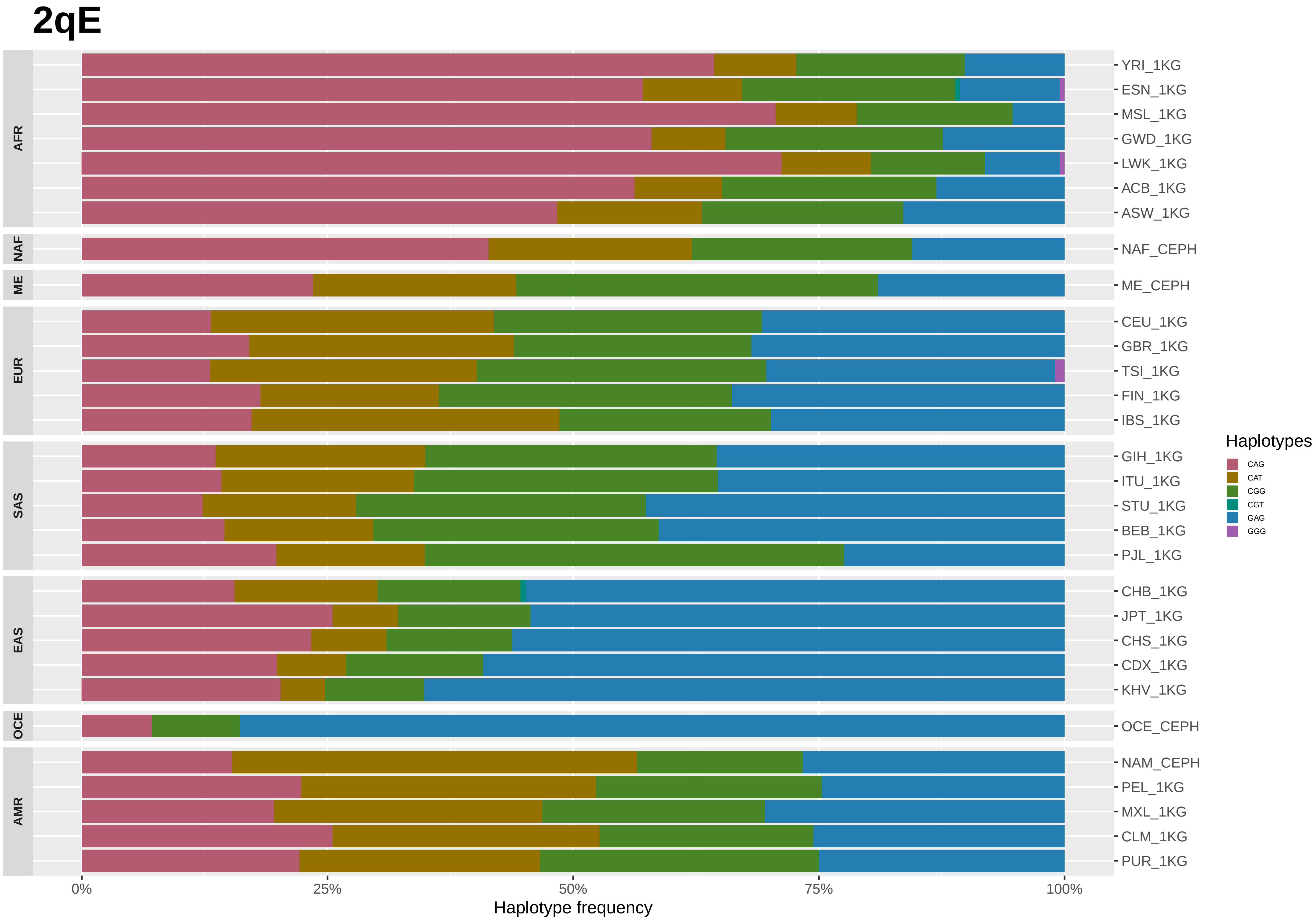
2qE
AFR
NAF
ME
EUR
SAS
EAS
OCE
AMR
YRI_1KG
ESN_1KG
MSL_1KG
GWD_1KG
LWK_1KG
ACB_1KG
ASW_1KG
NAF_CEPH
ME_CEPH
CEU_1KG
GBR_1KG
TSI_1KG
FIN_1KG
IBS_1KG
GIH_1KG
ITU_1KG
STU_1KG
BEB_1KG
PJL_1KG
CHB_1KG
JPT_1KG
CHS_1KG
CDX_1KG
KHV_1KG
OCE_CEPH
NAM_CEPH
PEL_1KG
MXL_1KG
CLM_1KG
PUR_1KG
0%
25%
50%
75%
100%
Haplotype frequency
Haplotypes
CAG
CAT
CGG
CGT
GAG
GGG

# 3pA

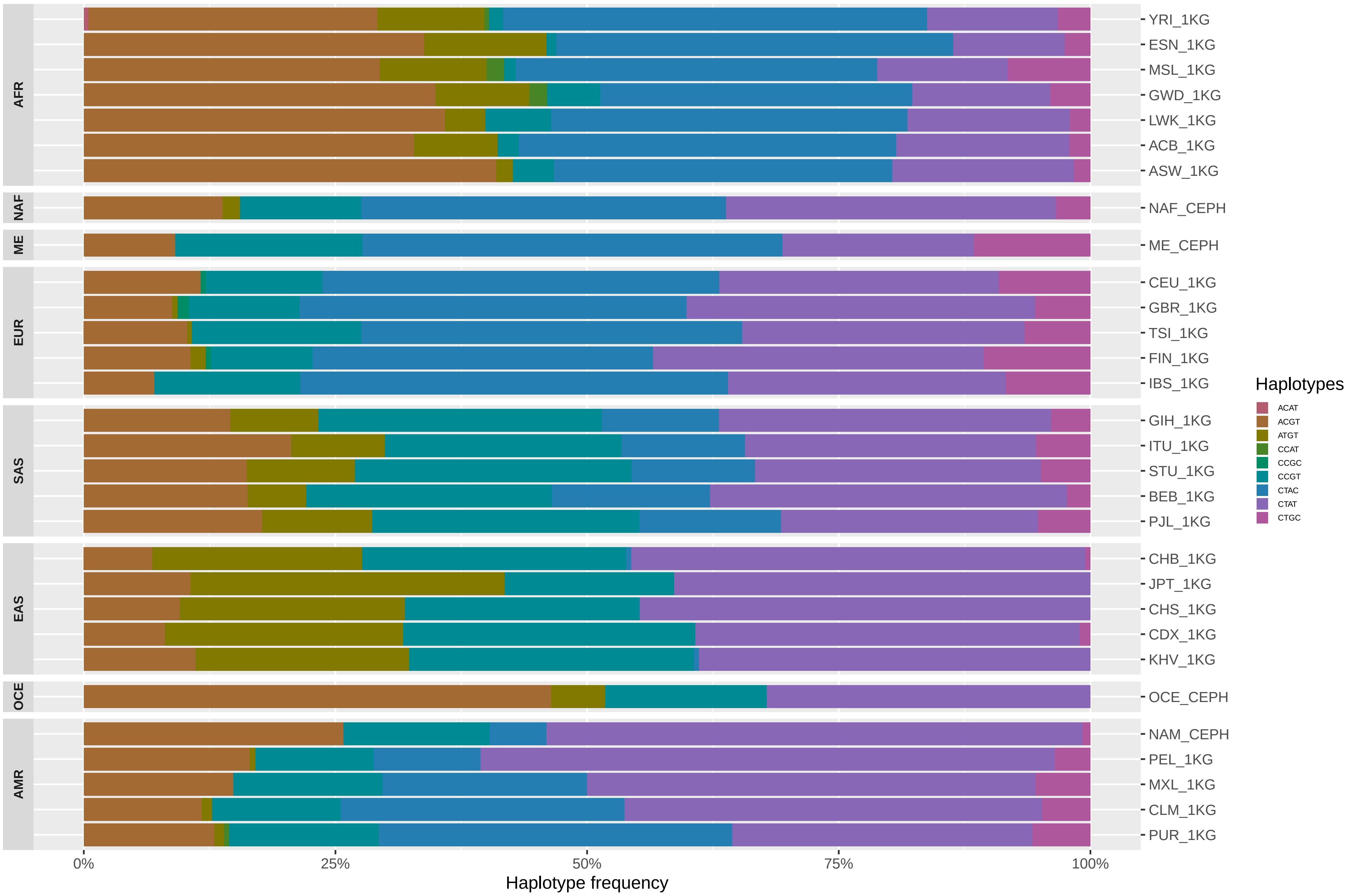
AFR
NAF
ME
EUR
SAS
EAS
OCE
AMR
YRI_1KG
ESN_1KG
MSL_1KG
GWD_1KG
LWK_1KG
ACB_1KG
ASW_1KG
NAF_CEPH
ME_CEPH
CEU_1KG
GBR_1KG
TSI_1KG
FIN_1KG
IBS_1KG
GIH_1KG
ITU_1KG
STU_1KG
BEB_1KG
PJL_1KG
CHB_1KG
JPT_1KG
CHS_1KG
CDX_1KG
KHV_1KG
OCE_CEPH
NAM_CEPH
PEL_1KG
MXL_1KG
CLM_1KG
PUR_1KG
0%
25%
50%
75%
100%
Haplotype frequency
Haplotypes
ACAT
ACGT
ATGT
CCAT
CCGC
CCGT
CTAC
CTAT
CTGC

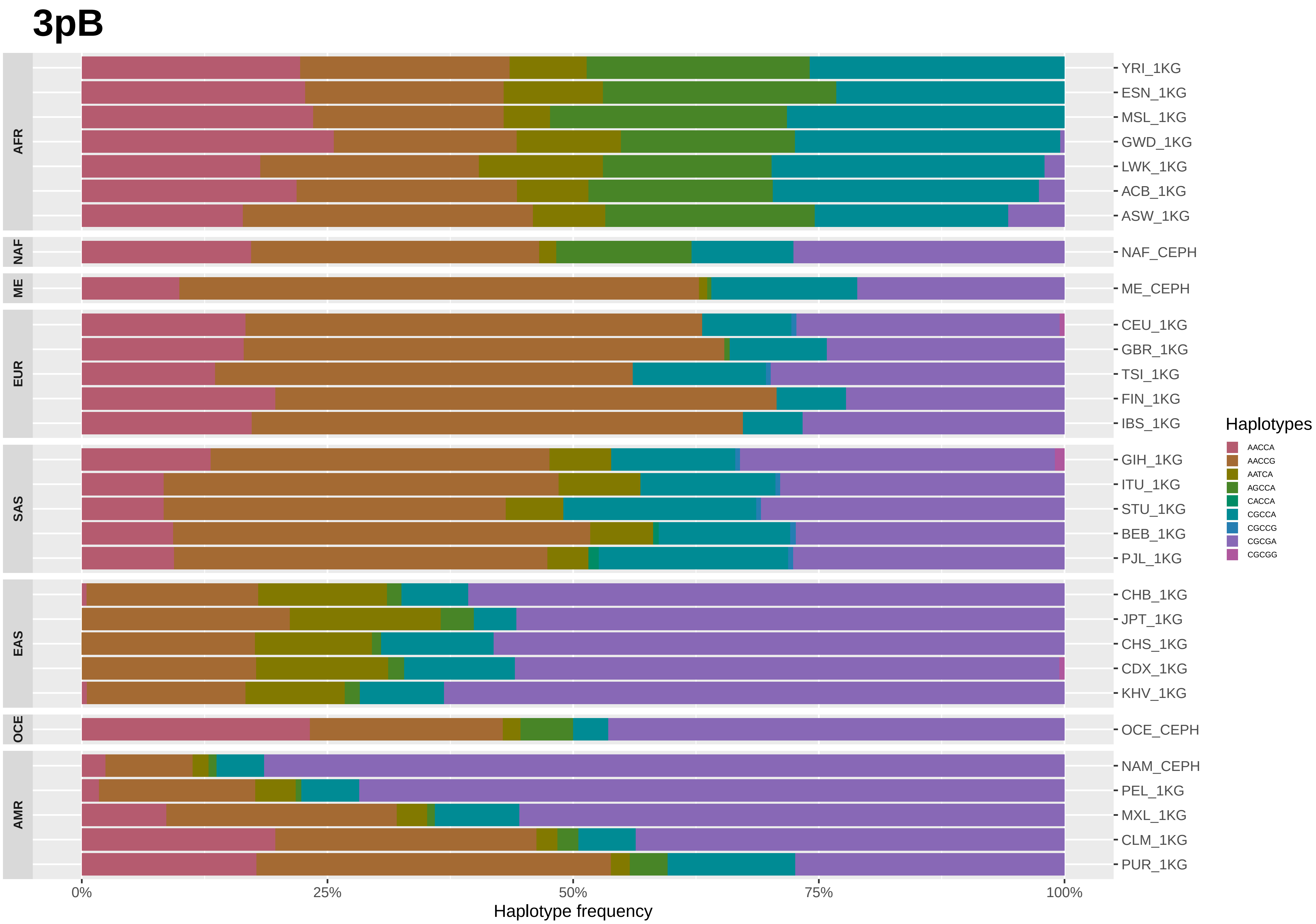
3pB
AFR
NAF
ME
EUR
SAS
EAS
OCE
AMR
YRI_1KG
ESN_1KG
MSL_1KG
GWD_1KG
LWK_1KG
ACB_1KG
ASW_1KG
NAF_CEPH
ME_CEPH
CEU_1KG
GBR_1KG
TSI_1KG
FIN_1KG
IBS_1KG
GIH_1KG
ITU_1KG
STU_1KG
BEB_1KG
PJL_1KG
CHB_1KG
JPT_1KG
CHS_1KG
CDX_1KG
KHV_1KG
OCE_CEPH
NAM_CEPH
PEL_1KG
MXL_1KG
CLM_1KG
PUR_1KG
0%
25%
50%
75%
100%
Haplotype frequency
Haplotypes
AACCA
AACCG
AATCA
AGCCA
CACCA
CGCCA
CGCCG
CGCGA
CGCGG

# 3qA

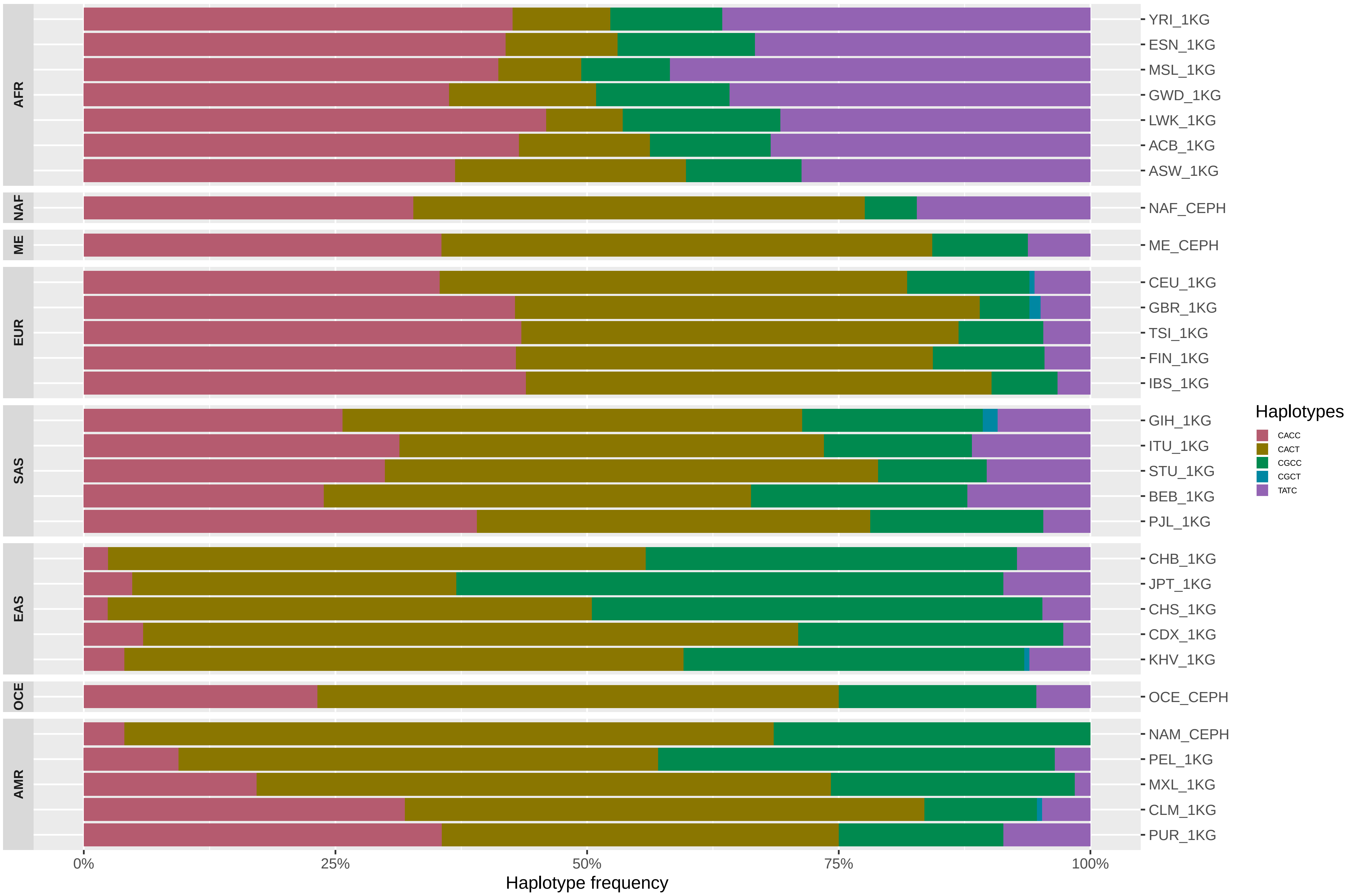

AFR
NAF
ME
EUR
SAS
EAS
OCE
AMR
YRI_1KG
ESN_1KG
MSL_1KG
GWD_1KG
LWK_1KG
ACB_1KG
ASW_1KG
NAF_CEPH
ME_CEPH
CEU_1KG
GBR_1KG
TSI_1KG
FIN_1KG
IBS_1KG
GIH_1KG
ITU_1KG
STU_1KG
BEB_1KG
PJL_1KG
CHB_1KG
JPT_1KG
CHS_1KG
CDX_1KG
KHV_1KG
OCE_CEPH
NAM_CEPH
PEL_1KG
MXL_1KG
CLM_1KG
PUR_1KG
0%
25%
50%
75%
100%
Haplotype frequency
Haplotypes
CACC
CACT
CGCC
CGCT
TATC

# 3qB

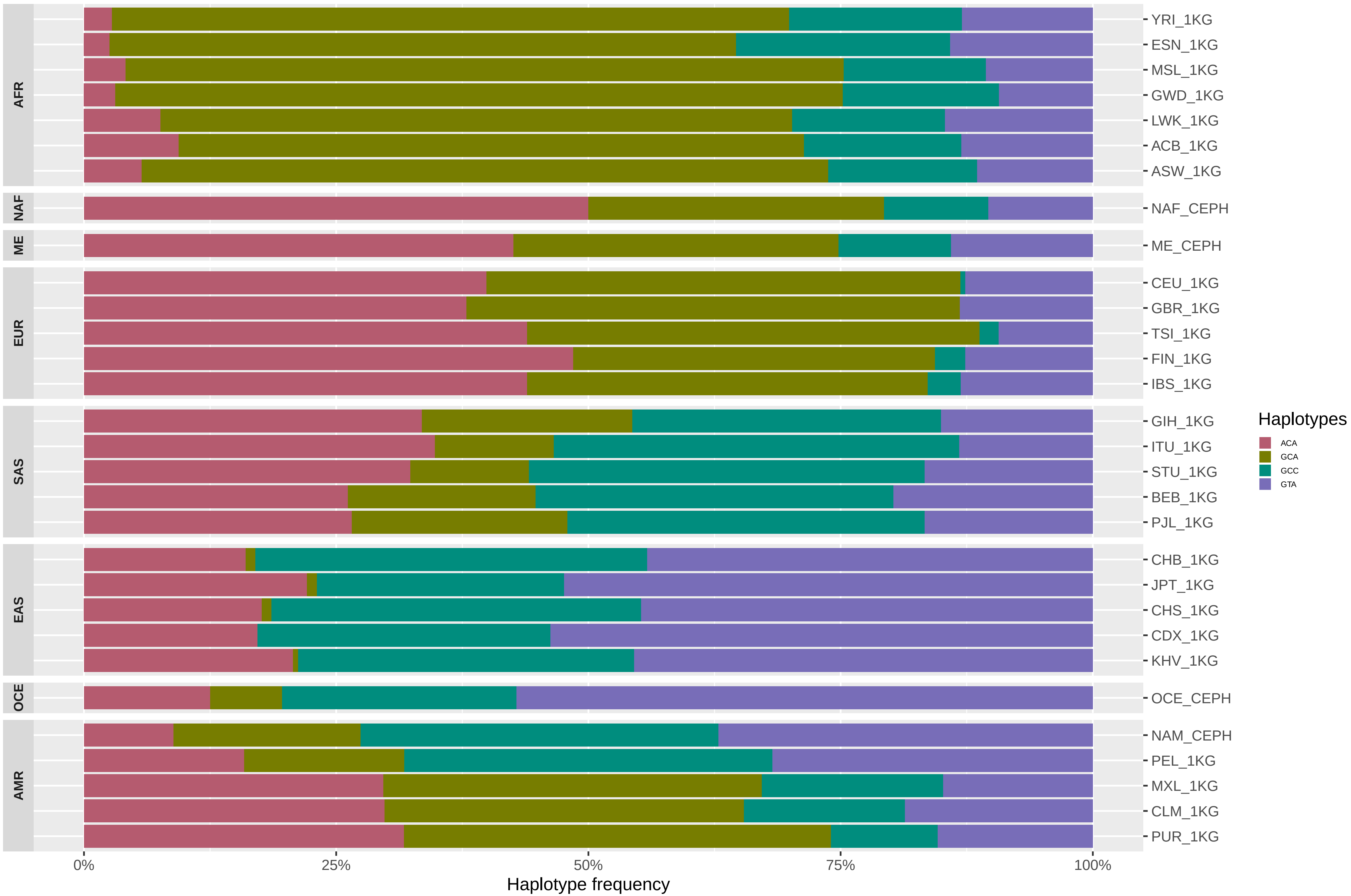
AFR
NAF
ME
EUR
SAS
EAS
OCE
AMR
YRI_1KG
ESN_1KG
MSL_1KG
GWD_1KG
LWK_1KG
ACB_1KG
ASW_1KG
NAF_CEPH
ME_CEPH
CEU_1KG
GBR_1KG
TSI_1KG
FIN_1KG
IBS_1KG
GIH_1KG
ITU_1KG
STU_1KG
BEB_1KG
PJL_1KG
CHB_1KG
JPT_1KG
CHS_1KG
CDX_1KG
KHV_1KG
OCE_CEPH
NAM_CEPH
PEL_1KG
MXL_1KG
CLM_1KG
PUR_1KG
0%
25%
50%
75%
100%
Haplotype frequency
Haplotypes
ACA
GCA
GCC
GTA

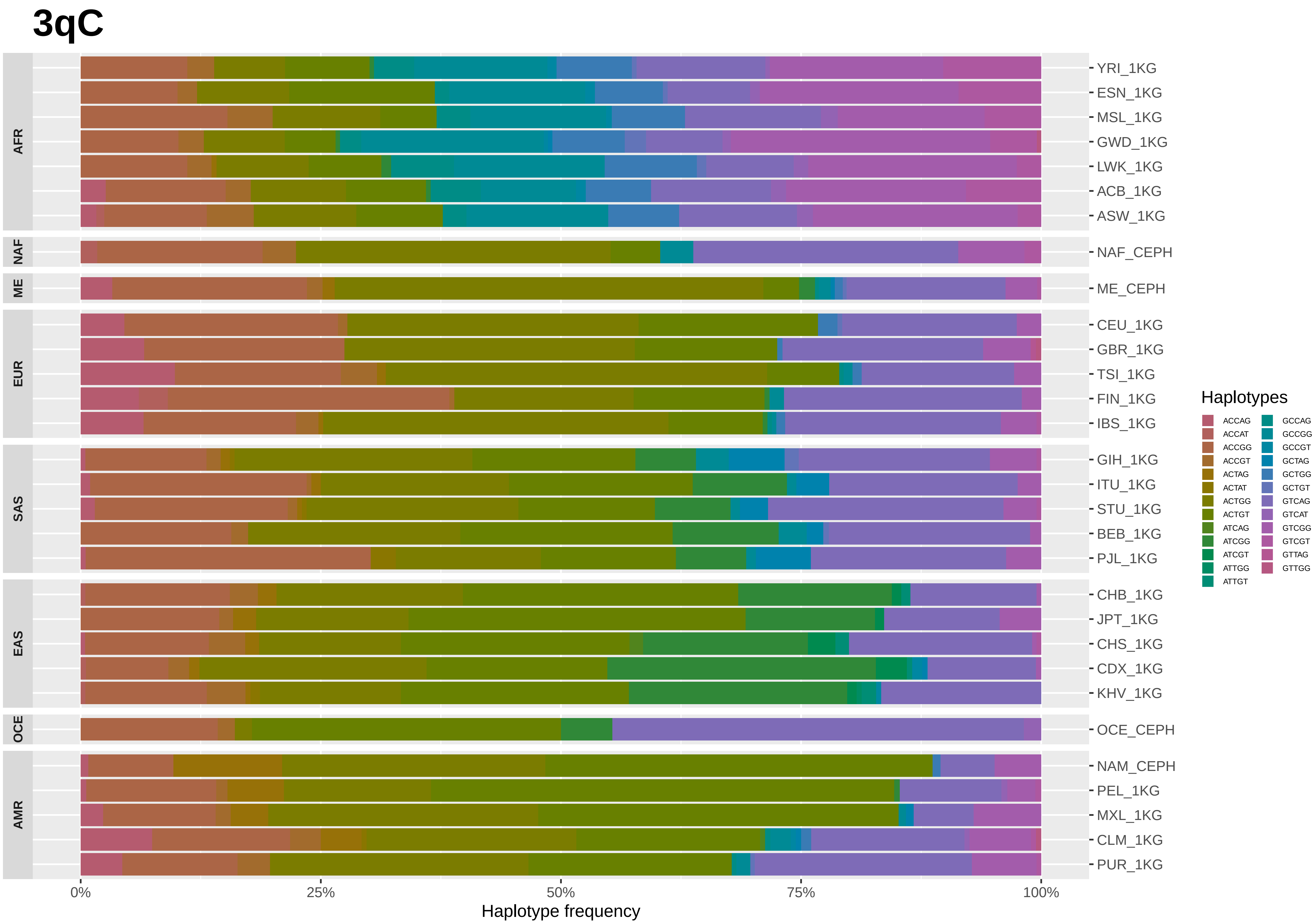
3qC
AFR
NAF
ME
EUR
SAS
EAS
OCE
AMR
YRI_1KG
ESN_1KG
MSL_1KG
GWD_1KG
LWK_1KG
ACB_1KG
ASW_1KG
NAF_CEPH
ME_CEPH
CEU_1KG
GBR_1KG
TSI_1KG
FIN_1KG
IBS_1KG
GIH_1KG
ITU_1KG
STU_1KG
BEB_1KG
PJL_1KG
CHB_1KG
JPT_1KG
CHS_1KG
CDX_1KG
KHV_1KG
OCE_CEPH
NAM_CEPH
PEL_1KG
MXL_1KG
CLM_1KG
PUR_1KG
0%
25%
50%
75%
100%
Haplotype frequency
Haplotypes
ACCAG
ACCAT
ACCGG
ACCGT
ACTAG
ACTAT
ACTGG
ACTGT
ATCAG
ATCGG
ATCGT
ATTGG
ATTGT
GCCAG
GCCGG
GCCGT
GCTAG
GCTGG
GCTGT
GTCAG
GTCAT
GTCGG
GTCGT
GTTAG
GTTGG

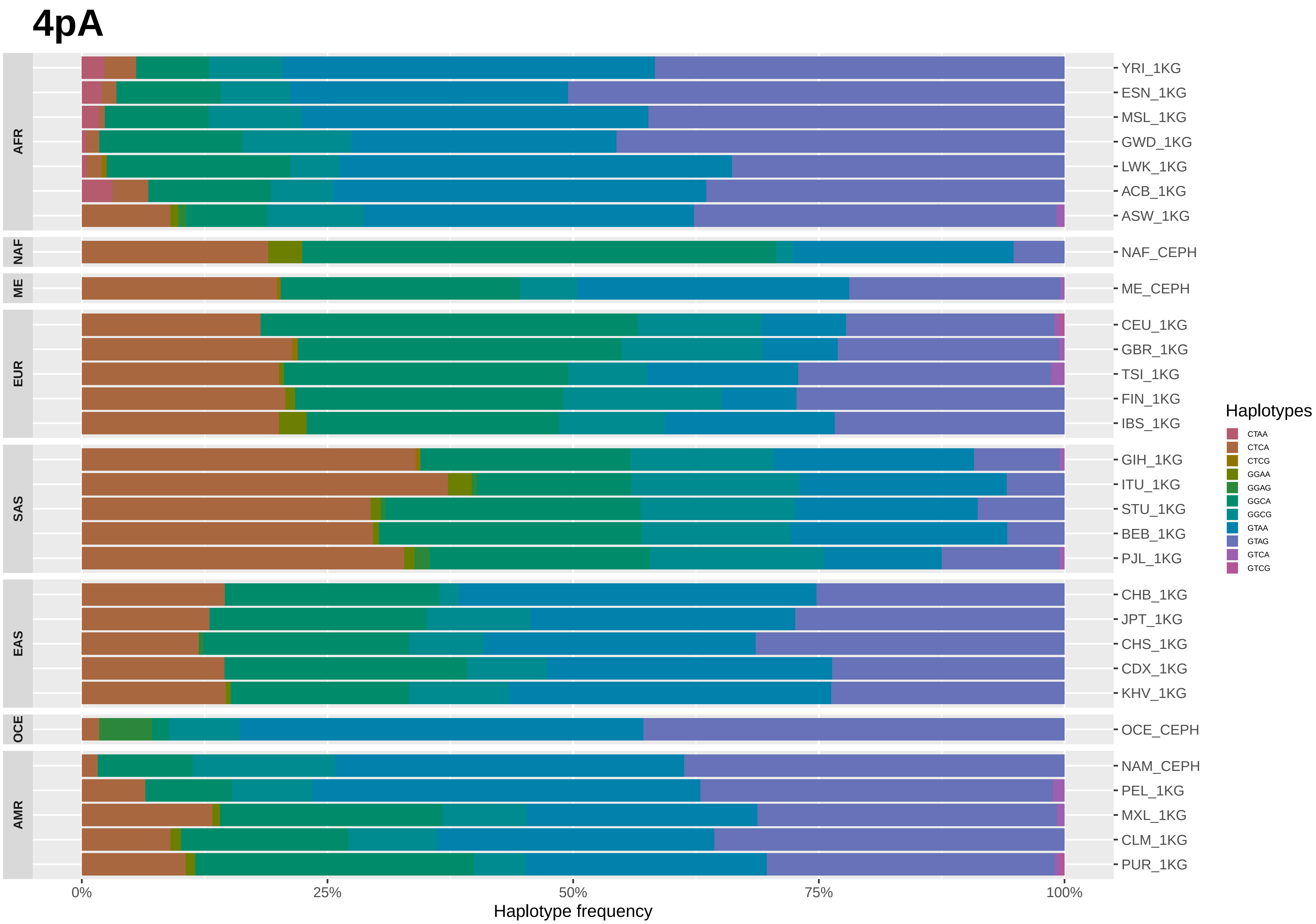
4pA
AFR
NAF
ME
EUR
SAS
EAS
OCE
AMR
YRI_1KG
ESN_1KG
MSL_1KG
GWD_1KG
LWK_1KG
ACB_1KG
ASW_1KG
NAF_CEPH
ME_CEPH
CEU_1KG
GBR_1KG
TSI_1KG
FIN_1KG
IBS_1KG
GIH_1KG
ITU_1KG
STU_1KG
BEB_1KG
PJL_1KG
CHB_1KG
JPT_1KG
CHS_1KG
CDX_1KG
KHV_1KG
OCE_CEPH
NAM_CEPH
PEL_1KG
MXL_1KG
CLM_1KG
PUR_1KG
0%
25%
50%
75%
100%
Haplotype frequency
Haplotypes
CTAA
CTCA
CTCG
GGAA
GGAG
GGCA
GGCG
GTAA
GTAG
GTCA
GTCG

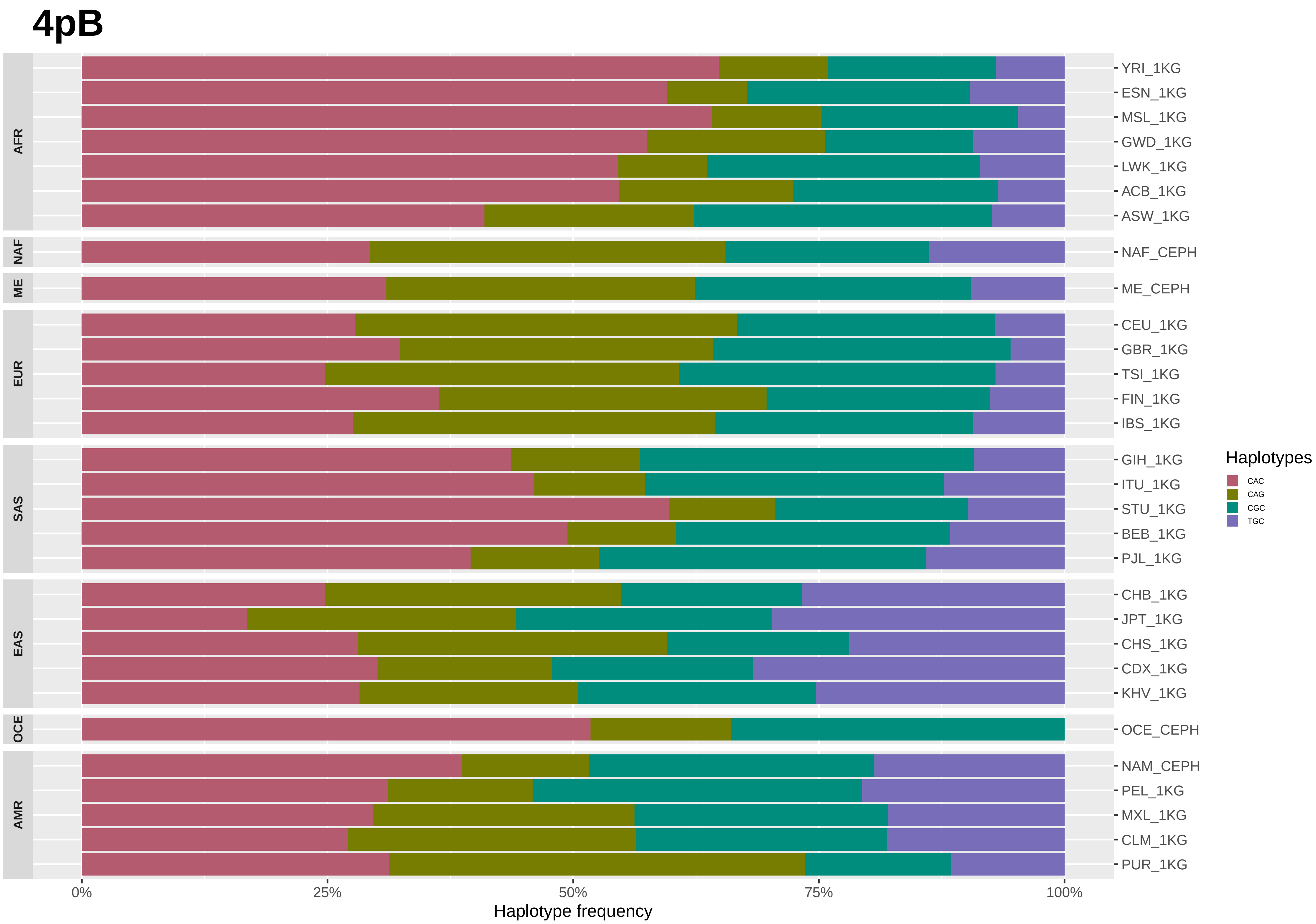
4pB
AFR
NAF
ME
EUR
SAS
EAS
OCE
AMR
YRI_1KG
ESN_1KG
MSL_1KG
GWD_1KG
LWK_1KG
ACB_1KG
ASW_1KG
NAF_CEPH
ME_CEPH
CEU_1KG
GBR_1KG
TSI_1KG
FIN_1KG
IBS_1KG
GIH_1KG
ITU_1KG
STU_1KG
BEB_1KG
PJL_1KG
CHB_1KG
JPT_1KG
CHS_1KG
CDX_1KG
KHV_1KG
OCE_CEPH
NAM_CEPH
PEL_1KG
MXL_1KG
CLM_1KG
PUR_1KG
0%
25%
50%
75%
100%
Haplotype frequency
Haplotypes
CAC
CAG
CGC
TGC

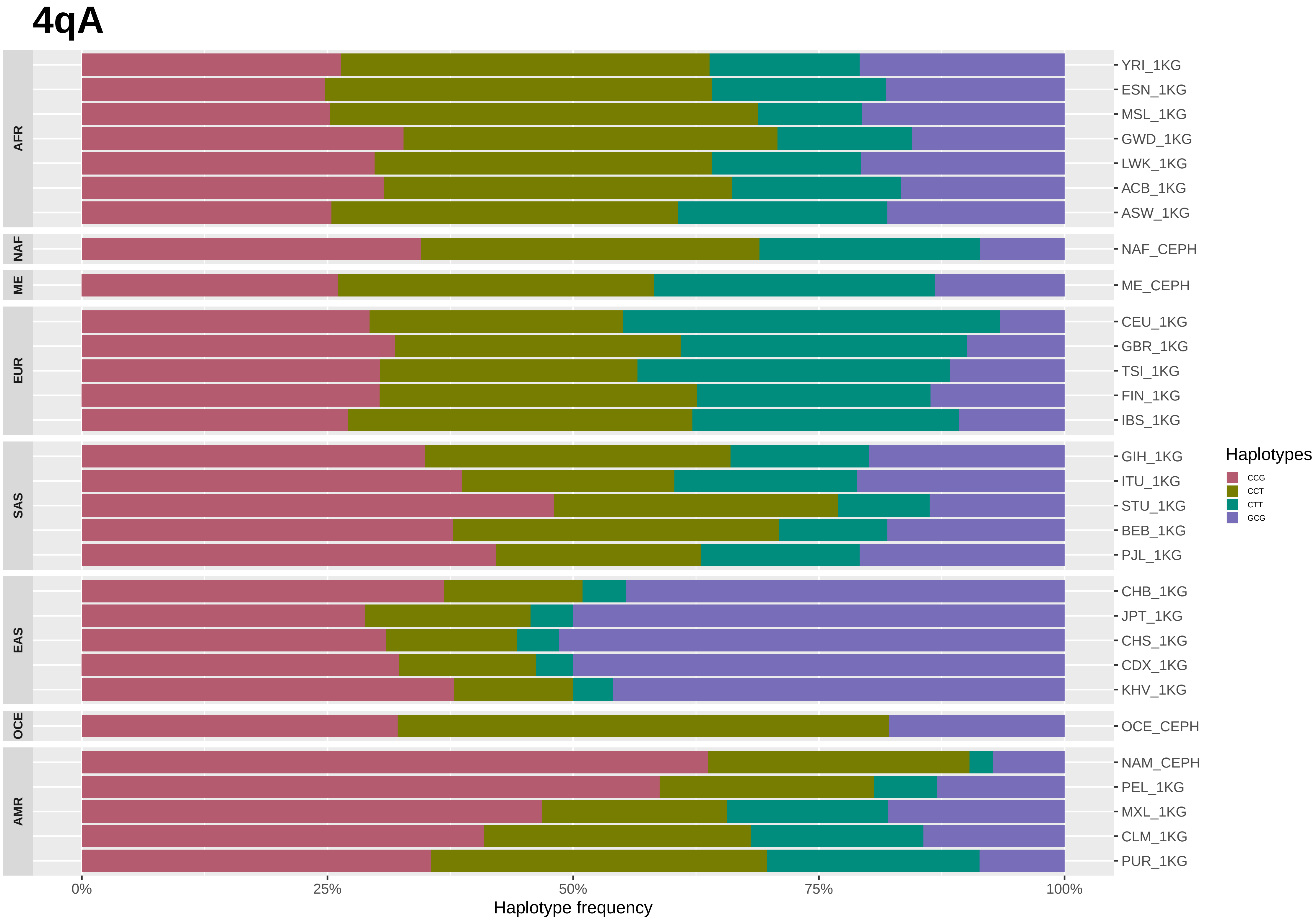

4qA
AFR
NAF
ME
EUR
SAS
EAS
OCE
AMR
YRI_1KG
ESN_1KG
MSL_1KG
GWD_1KG
LWK_1KG
ACB_1KG
ASW_1KG
NAF_CEPH
ME_CEPH
CEU_1KG
GBR_1KG
TSI_1KG
FIN_1KG
IBS_1KG
GIH_1KG
ITU_1KG
STU_1KG
BEB_1KG
PJL_1KG
CHB_1KG
JPT_1KG
CHS_1KG
CDX_1KG
KHV_1KG
OCE_CEPH
NAM_CEPH
PEL_1KG
MXL_1KG
CLM_1KG
PUR_1KG
0%
25%
50%
75%
100%
Haplotype frequency
Haplotypes
CCG
CCT
CTT
GCG

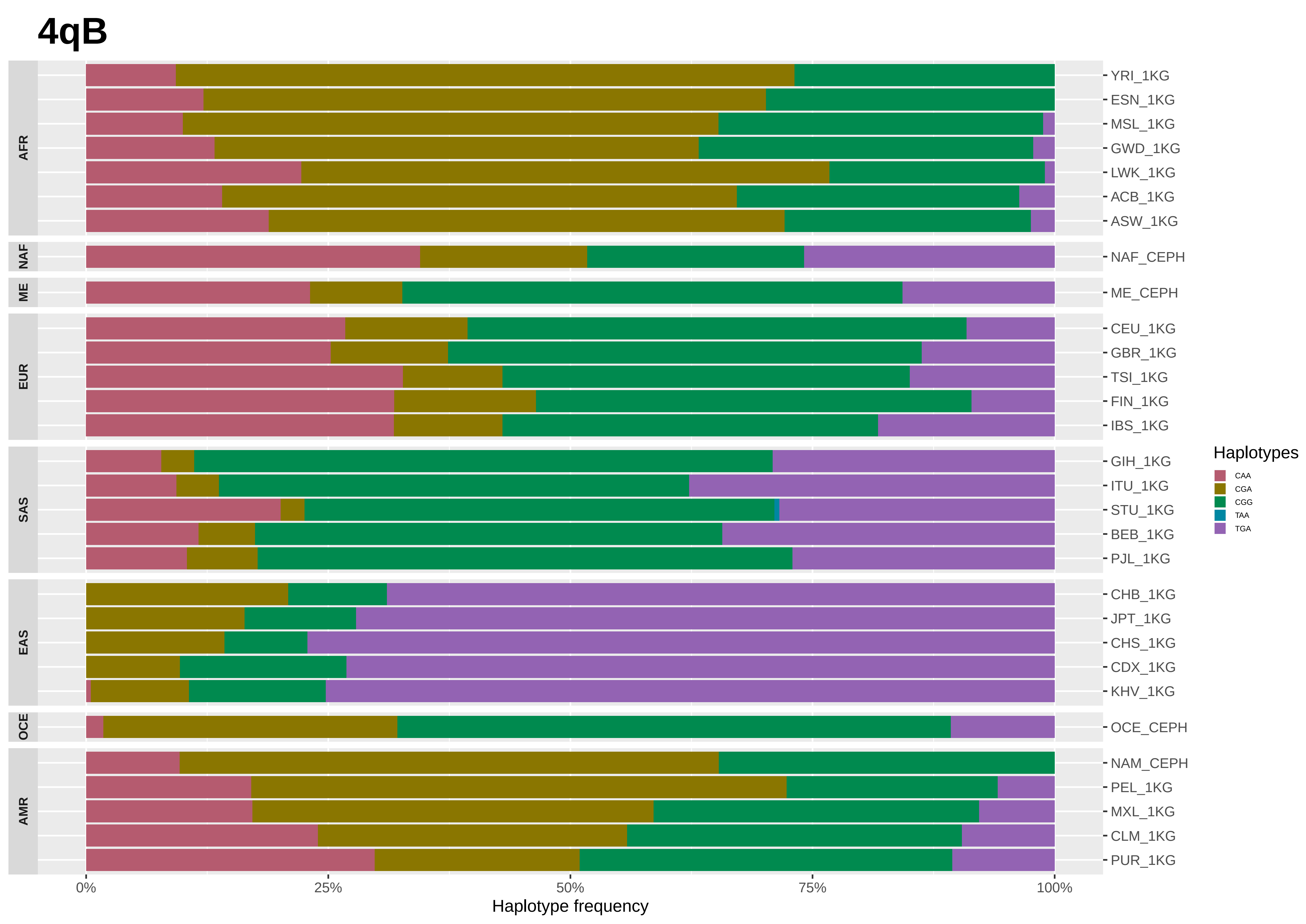
4qB
AFR
NAF
ME
EUR
SAS
EAS
OCE
AMR
YRI_1KG
ESN_1KG
MSL_1KG
GWD_1KG
LWK_1KG
ACB_1KG
ASW_1KG
NAF_CEPH
ME_CEPH
CEU_1KG
GBR_1KG
TSI_1KG
FIN_1KG
IBS_1KG
GIH_1KG
ITU_1KG
STU_1KG
BEB_1KG
PJL_1KG
CHB_1KG
JPT_1KG
CHS_1KG
CDX_1KG
KHV_1KG
OCE_CEPH
NAM_CEPH
PEL_1KG
MXL_1KG
CLM_1KG
PUR_1KG
0%
25%
50%
75%
100%
Haplotype frequency
Haplotypes
CAA
CGA
CGG
TAA
TGA

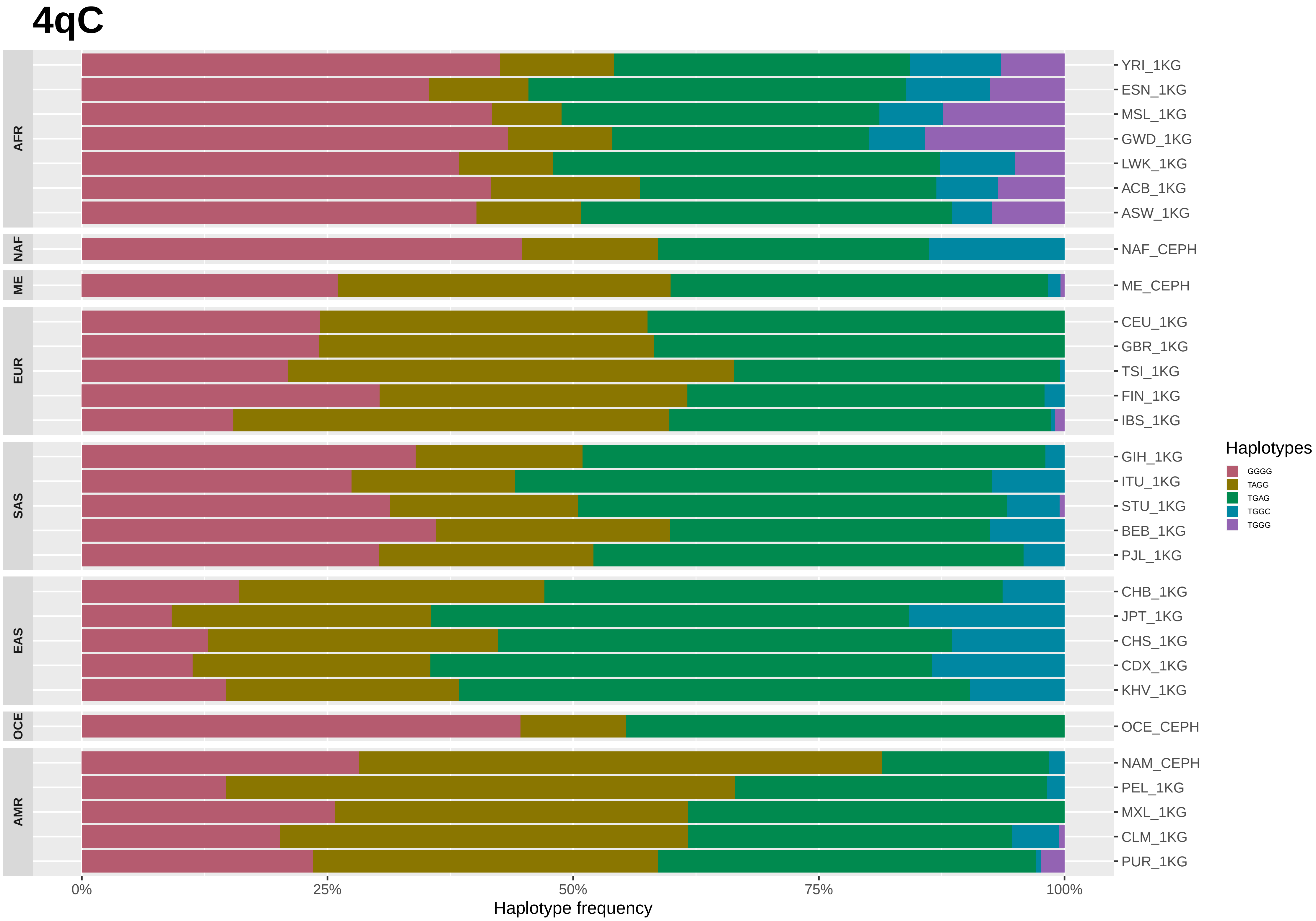
4qC
AFR
NAF
ME
EUR
SAS
EAS
OCE
AMR
YRI_1KG
ESN_1KG
MSL_1KG
GWD_1KG
LWK_1KG
ACB_1KG
ASW_1KG
NAF_CEPH
ME_CEPH
CEU_1KG
GBR_1KG
TSI_1KG
FIN_1KG
IBS_1KG
GIH_1KG
ITU_1KG
STU_1KG
BEB_1KG
PJL_1KG
CHB_1KG
JPT_1KG
CHS_1KG
CDX_1KG
KHV_1KG
OCE_CEPH
NAM_CEPH
PEL_1KG
MXL_1KG
CLM_1KG
PUR_1KG
0%
25%
50%
75%
100%
Haplotype frequency
Haplotypes
GGGG
TAGG
TGAG
TGGC
TGGG

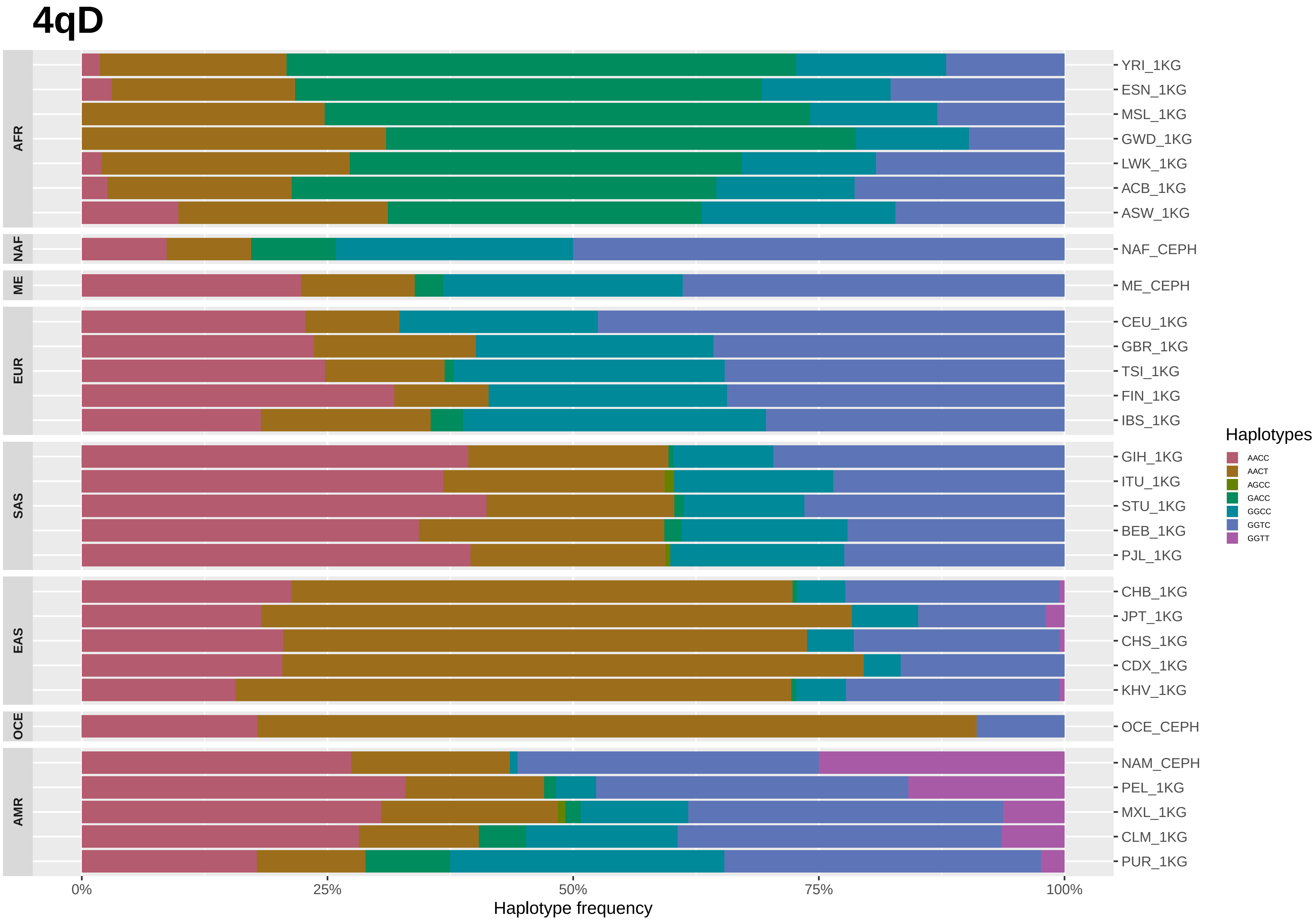
4qD
AFR
NAF
ME
EUR
SAS
EAS
OCE
AMR
YRI_1KG
ESN_1KG
MSL_1KG
GWD_1KG
LWK_1KG
ACB_1KG
ASW_1KG
NAF_CEPH
ME_CEPH
CEU_1KG
GBR_1KG
TSI_1KG
FIN_1KG
IBS_1KG
GIH_1KG
ITU_1KG
STU_1KG
BEB_1KG
PJL_1KG
CHB_1KG
JPT_1KG
CHS_1KG
CDX_1KG
KHV_1KG
OCE_CEPH
NAM_CEPH
PEL_1KG
MXL_1KG
CLM_1KG
PUR_1KG
0%
25%
50%
75%
100%
Haplotype frequency
Haplotypes
AACC
AACT
AGCC
GACC
GGCC
GGTC
GGTT

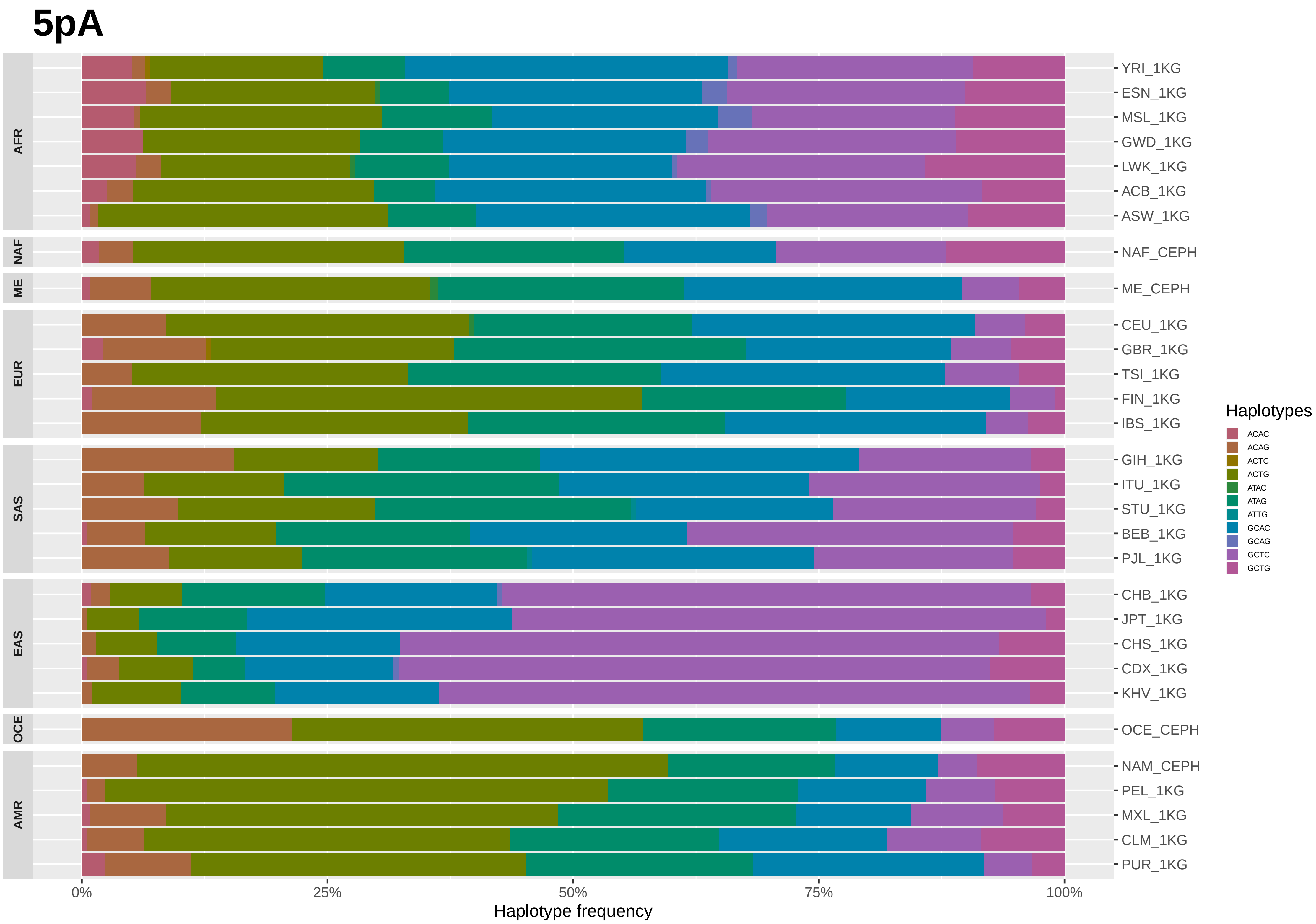
5pA
AFR
NAF
ME
EUR
SAS
EAS
OCE
AMR
YRI_1KG
ESN_1KG
MSL_1KG
GWD_1KG
LWK_1KG
ACB_1KG
ASW_1KG
NAF_CEPH
ME_CEPH
CEU_1KG
GBR_1KG
TSI_1KG
FIN_1KG
IBS_1KG
GIH_1KG
ITU_1KG
STU_1KG
BEB_1KG
PJL_1KG
CHB_1KG
JPT_1KG
CHS_1KG
CDX_1KG
KHV_1KG
OCE_CEPH
NAM_CEPH
PEL_1KG
MXL_1KG
CLM_1KG
PUR_1KG
0%
25%
50%
75%
100%
Haplotype frequency
Haplotypes
ACAC
ACAG
ACTC
ACTG
ATAC
ATAG
ATTG
GCAC
GCAG
GCTC
GCTG

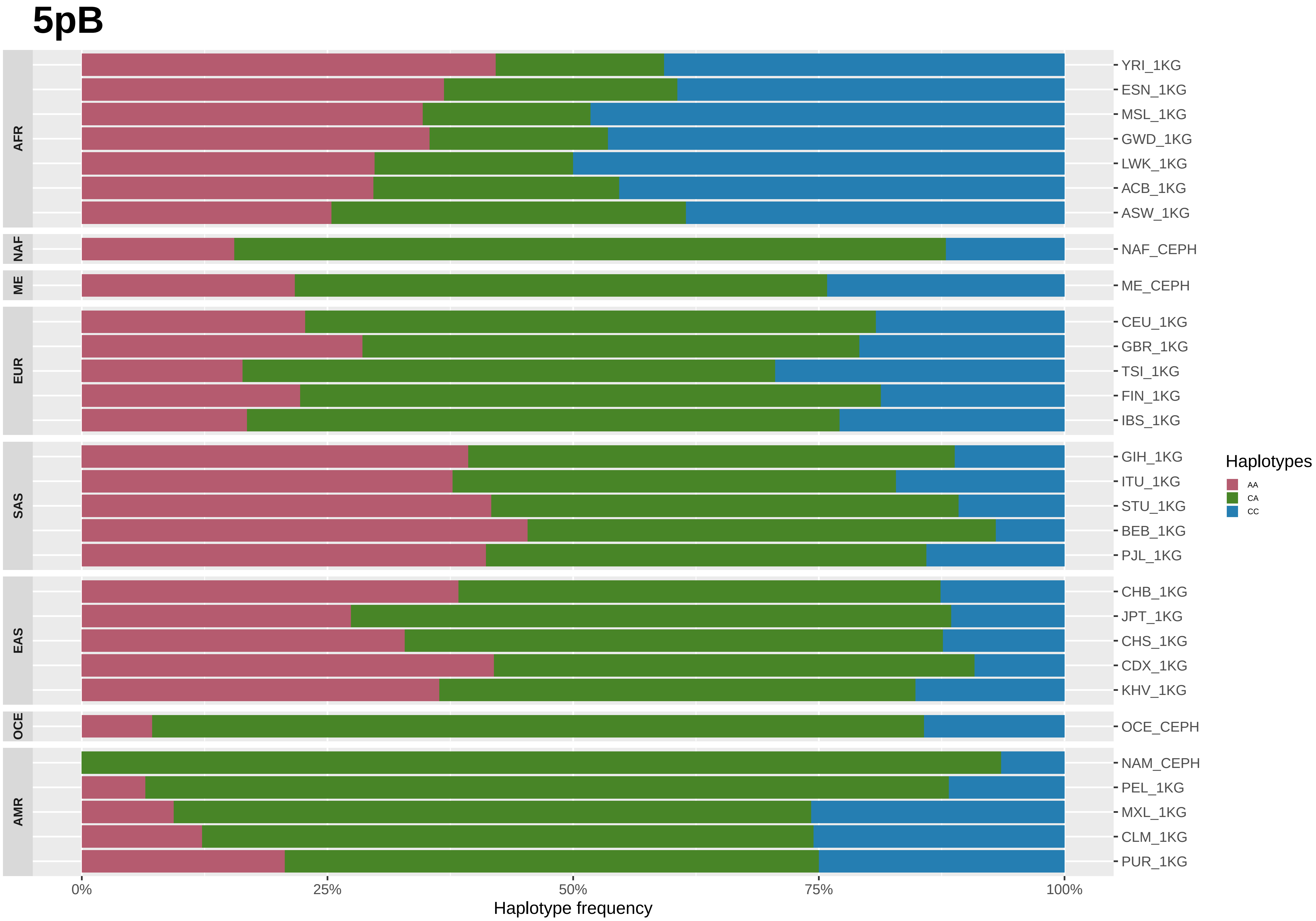
5pB
AFR
NAF
ME
EUR
SAS
EAS
OCE
AMR
YRI_1KG
ESN_1KG
MSL_1KG
GWD_1KG
LWK_1KG
ACB_1KG
ASW_1KG
NAF_CEPH
ME_CEPH
CEU_1KG
GBR_1KG
TSI_1KG
FIN_1KG
IBS_1KG
GIH_1KG
ITU_1KG
STU_1KG
BEB_1KG
PJL_1KG
CHB_1KG
JPT_1KG
CHS_1KG
CDX_1KG
KHV_1KG
OCE_CEPH
NAM_CEPH
PEL_1KG
MXL_1KG
CLM_1KG
PUR_1KG
0%
25%
50%
75%
100%
Haplotype frequency
Haplotypes
AA
CA
CC

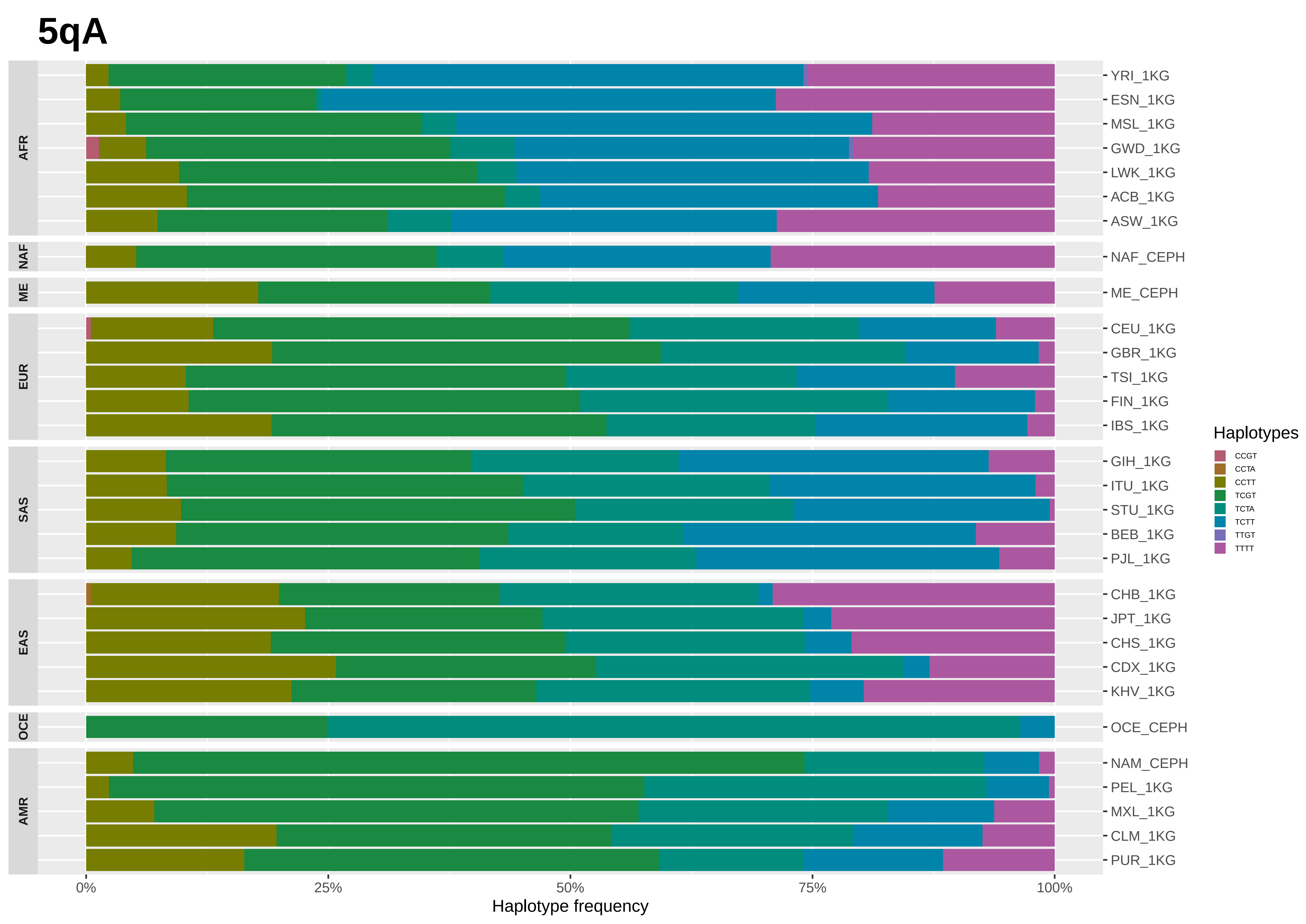

5qA
AFR
NAF
ME
EUR
SAS
EAS
OCE
AMR
YRI_1KG
ESN_1KG
MSL_1KG
GWD_1KG
LWK_1KG
ACB_1KG
ASW_1KG
NAF_CEPH
ME_CEPH
CEU_1KG
GBR_1KG
TSI_1KG
FIN_1KG
IBS_1KG
GIH_1KG
ITU_1KG
STU_1KG
BEB_1KG
PJL_1KG
CHB_1KG
JPT_1KG
CHS_1KG
CDX_1KG
KHV_1KG
OCE_CEPH
NAM_CEPH
PEL_1KG
MXL_1KG
CLM_1KG
PUR_1KG
0%
25%
50%
75%
100%
Haplotype frequency
Haplotypes
CCGT
CCTA
CCTT
TCGT
TCTA
TCTT
TTGT
TTTT

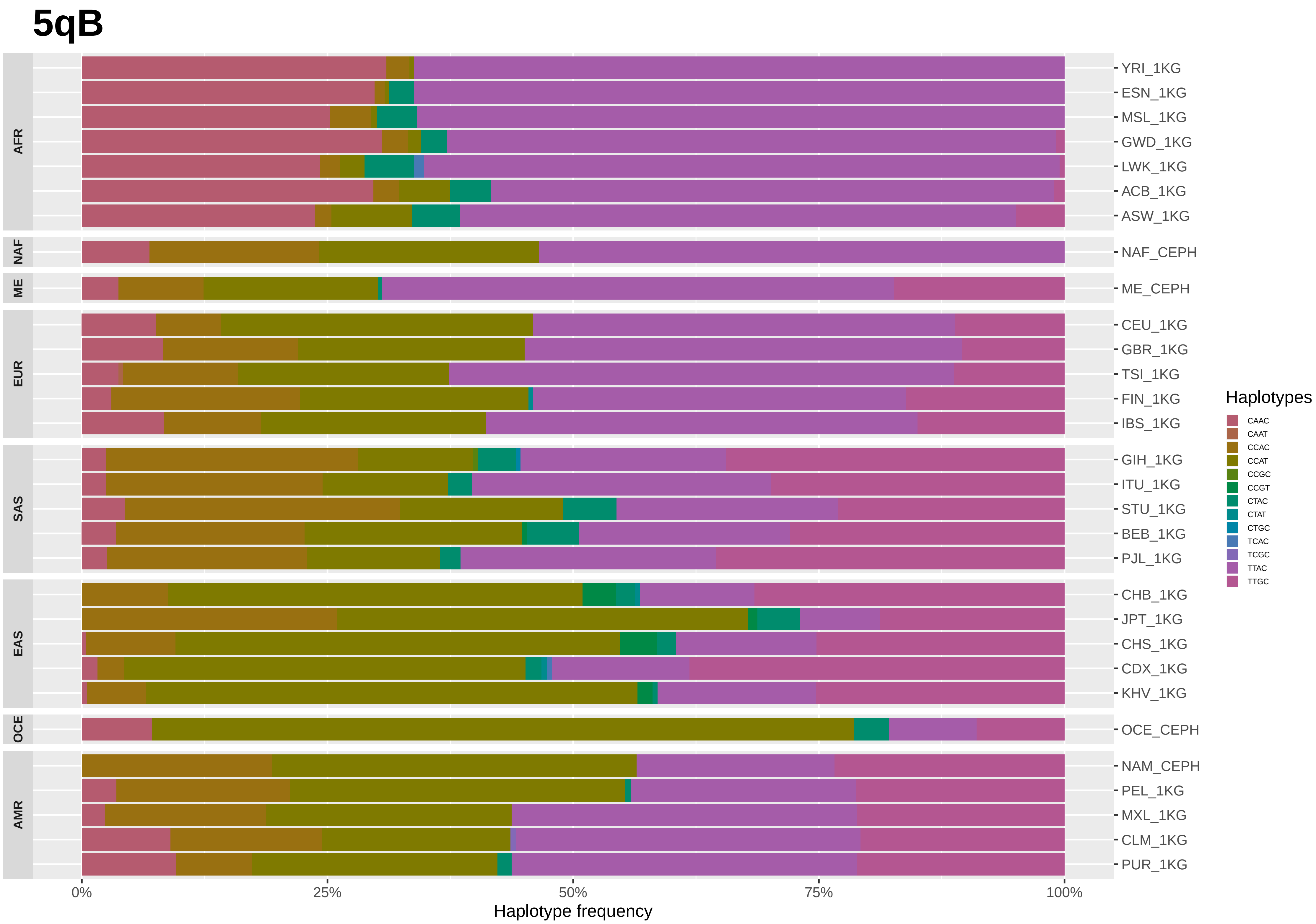
5qB
AFR
NAF
ME
EUR
SAS
EAS
OCE
AMR
YRI_1KG
ESN_1KG
MSL_1KG
GWD_1KG
LWK_1KG
ACB_1KG
ASW_1KG
NAF_CEPH
ME_CEPH
CEU_1KG
GBR_1KG
TSI_1KG
FIN_1KG
IBS_1KG
GIH_1KG
ITU_1KG
STU_1KG
BEB_1KG
PJL_1KG
CHB_1KG
JPT_1KG
CHS_1KG
CDX_1KG
KHV_1KG
OCE_CEPH
NAM_CEPH
PEL_1KG
MXL_1KG
CLM_1KG
PUR_1KG
0%
25%
50%
75%
100%
Haplotype frequency
Haplotypes
CAAC
CAAT
CCAC
CCAT
CCGC
CCGT
CTAC
CTAT
CTGC
TCAC
TCGC
TTAC
TTGC

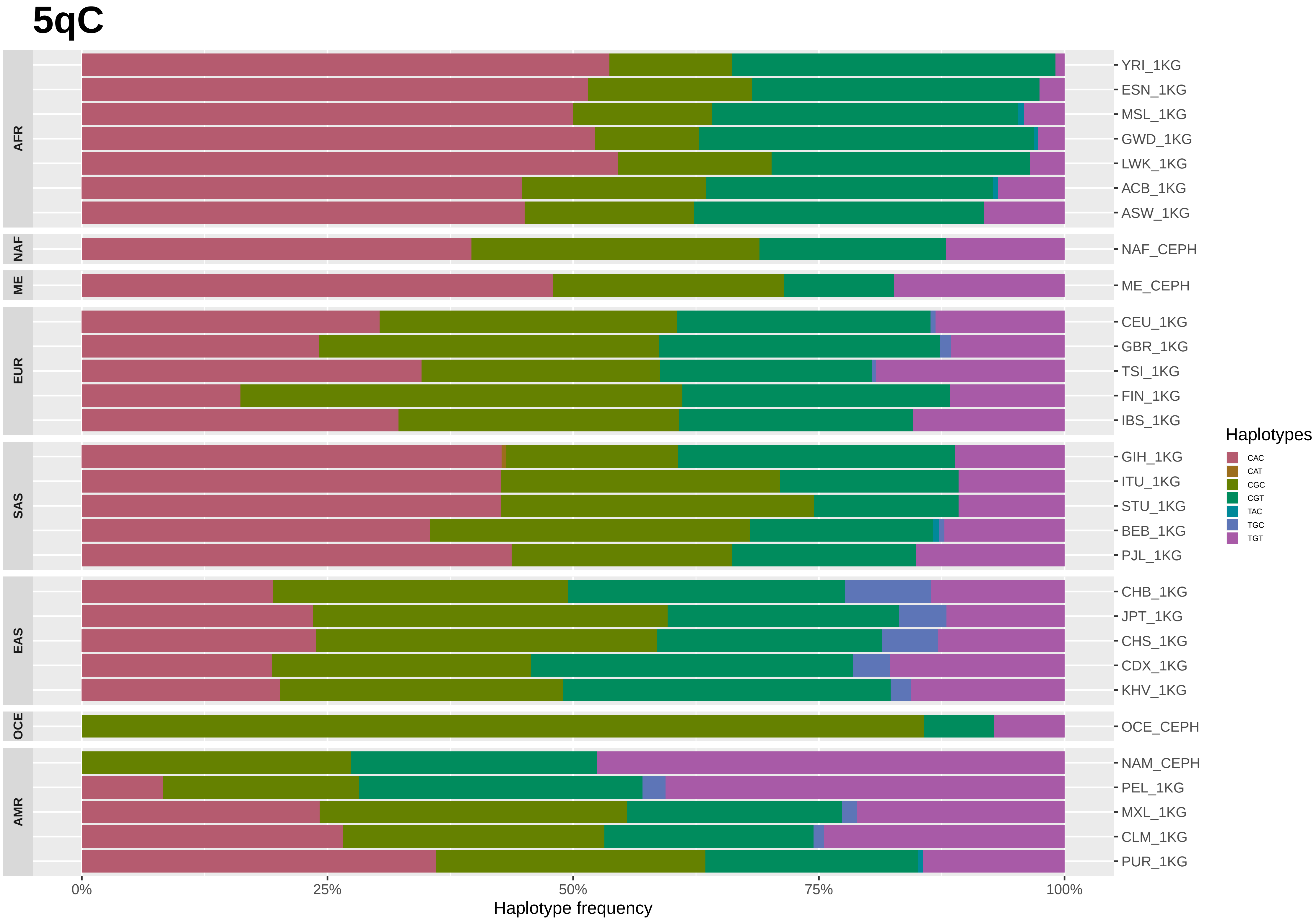

5qC
AFR
NAF
ME
EUR
SAS
EAS
OCE
AMR
YRI_1KG
ESN_1KG
MSL_1KG
GWD_1KG
LWK_1KG
ACB_1KG
ASW_1KG
NAF_CEPH
ME_CEPH
CEU_1KG
GBR_1KG
TSI_1KG
FIN_1KG
IBS_1KG
GIH_1KG
ITU_1KG
STU_1KG
BEB_1KG
PJL_1KG
CHB_1KG
JPT_1KG
CHS_1KG
CDX_1KG
KHV_1KG
OCE_CEPH
NAM_CEPH
PEL_1KG
MXL_1KG
CLM_1KG
PUR_1KG
0%
25%
50%
75%
100%
Haplotype frequency
Haplotypes
CAC
CAT
CGC
CGT
TAC
TGC
TGT

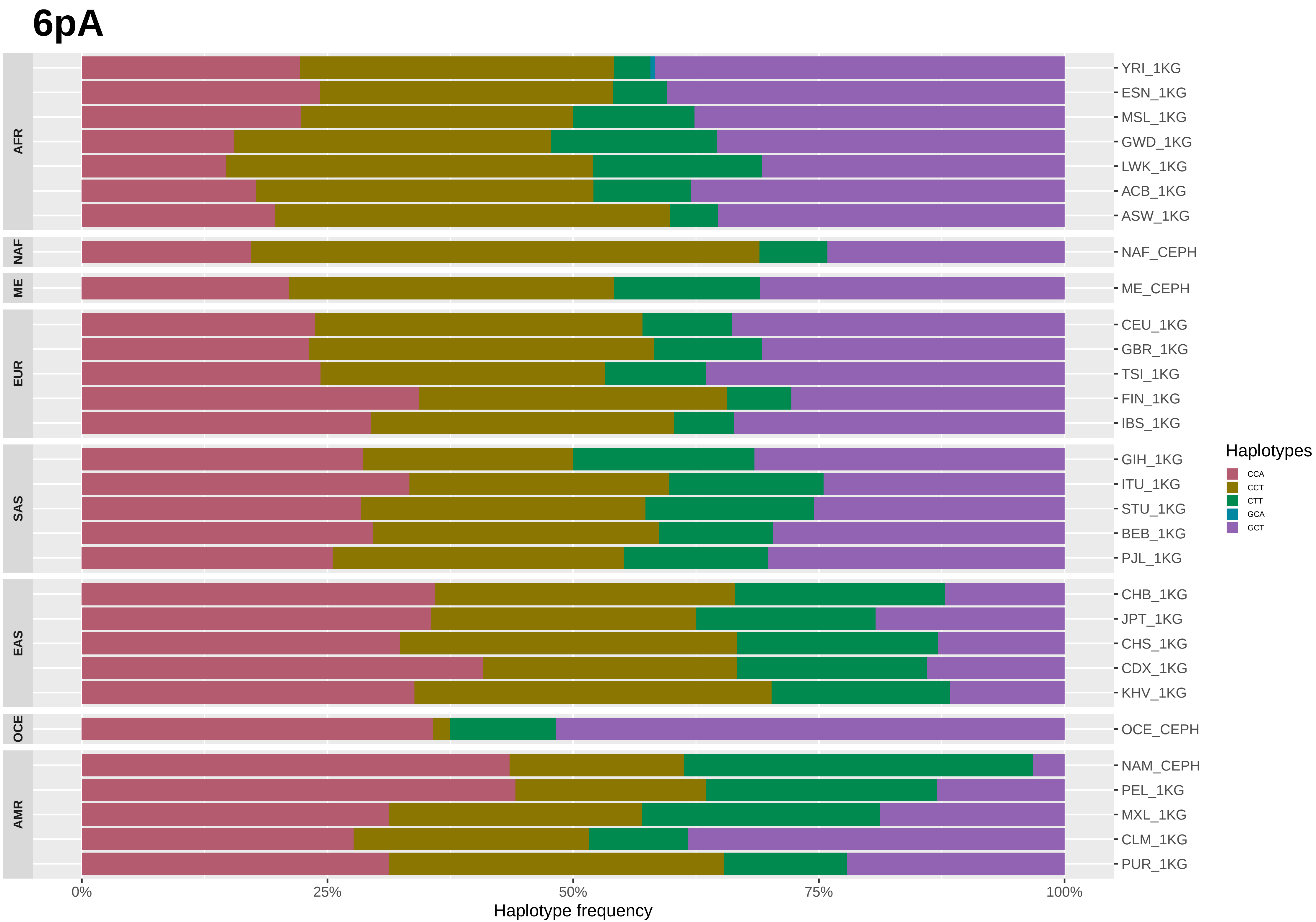
6pA
AFR
NAF
ME
EUR
SAS
EAS
OCE
AMR
YRI_1KG
ESN_1KG
MSL_1KG
GWD_1KG
LWK_1KG
ACB_1KG
ASW_1KG
NAF_CEPH
ME_CEPH
CEU_1KG
GBR_1KG
TSI_1KG
FIN_1KG
IBS_1KG
GIH_1KG
ITU_1KG
STU_1KG
BEB_1KG
PJL_1KG
CHB_1KG
JPT_1KG
CHS_1KG
CDX_1KG
KHV_1KG
OCE_CEPH
NAM_CEPH
PEL_1KG
MXL_1KG
CLM_1KG
PUR_1KG
0%
25%
50%
75%
100%
Haplotype frequency
Haplotypes
CCA
CCT
CTT
GCA
GCT

# 6pB

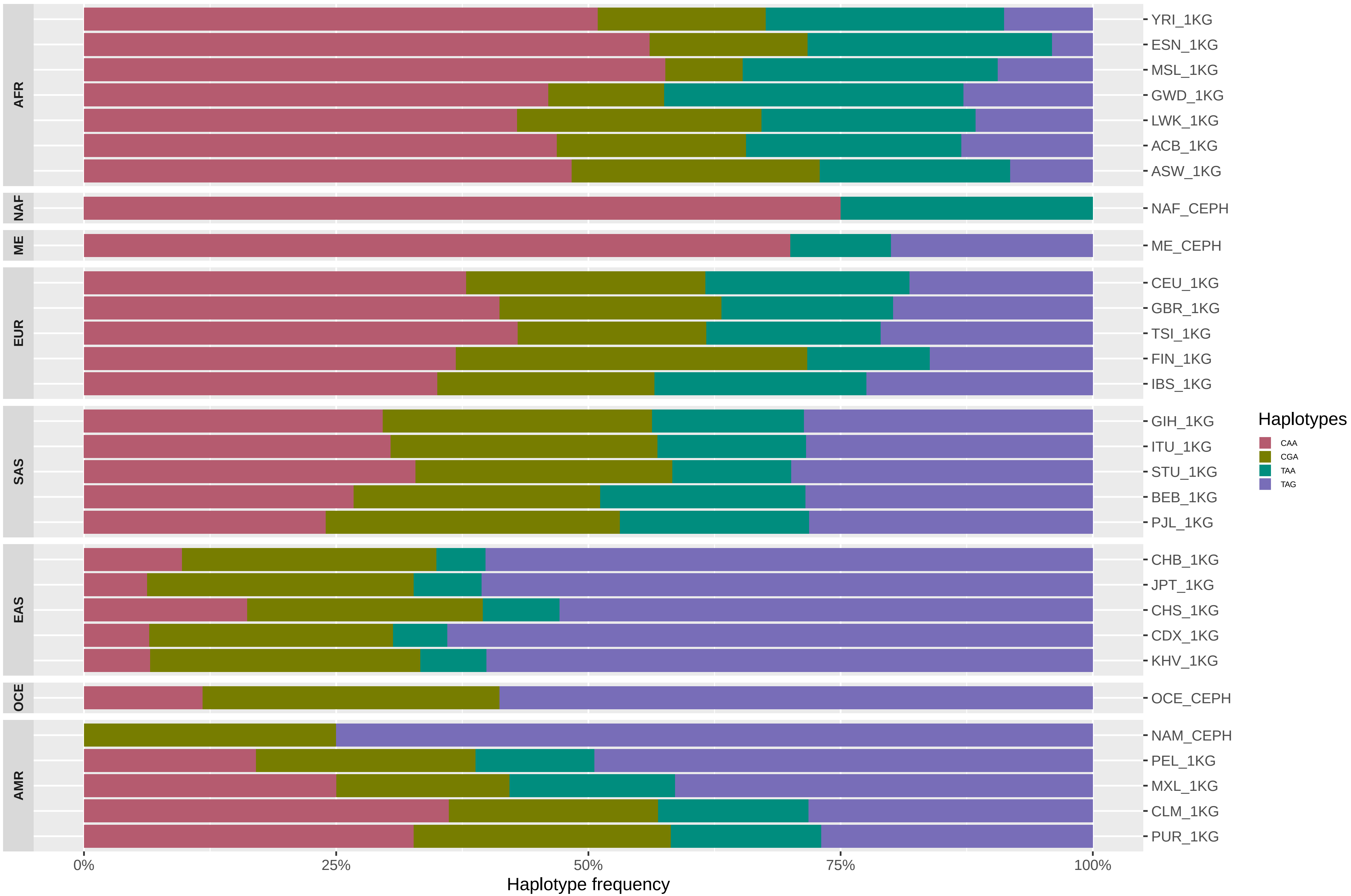
AFR
NAF
ME
EUR
SAS
EAS
OCE
AMR
YRI_1KG
ESN_1KG
MSL_1KG
GWD_1KG
LWK_1KG
ACB_1KG
ASW_1KG
NAF_CEPH
ME_CEPH
CEU_1KG
GBR_1KG
TSI_1KG
FIN_1KG
IBS_1KG
GIH_1KG
ITU_1KG
STU_1KG
BEB_1KG
PJL_1KG
CHB_1KG
JPT_1KG
CHS_1KG
CDX_1KG
KHV_1KG
OCE_CEPH
NAM_CEPH
PEL_1KG
MXL_1KG
CLM_1KG
PUR_1KG
0%
25%
50%
75%
100%
Haplotype frequency
Haplotypes
CAA
CGA
TAA
TAG

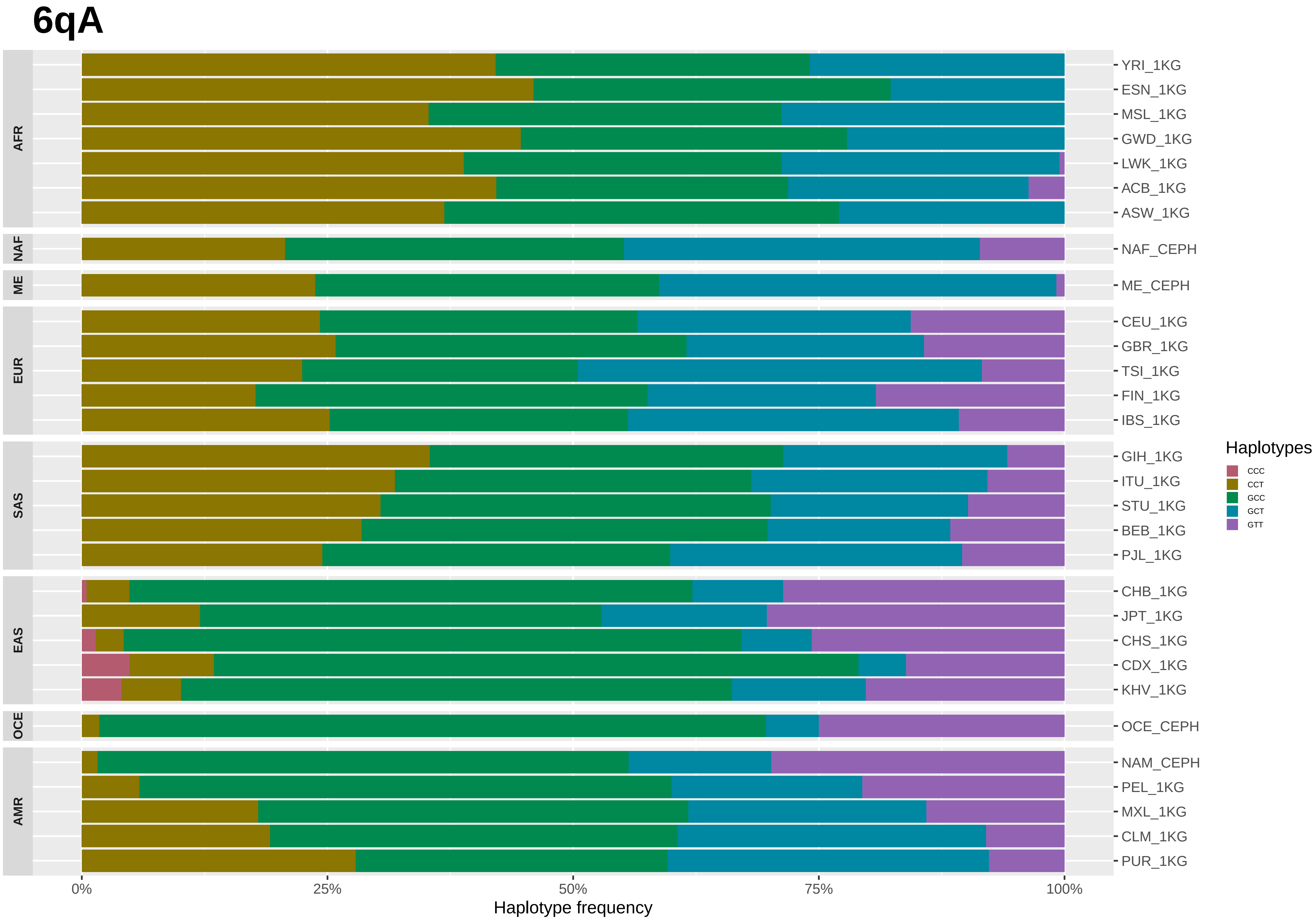

6qA
AFR
NAF
ME
EUR
SAS
EAS
OCE
AMR
YRI_1KG
ESN_1KG
MSL_1KG
GWD_1KG
LWK_1KG
ACB_1KG
ASW_1KG
NAF_CEPH
ME_CEPH
CEU_1KG
GBR_1KG
TSI_1KG
FIN_1KG
IBS_1KG
GIH_1KG
ITU_1KG
STU_1KG
BEB_1KG
PJL_1KG
CHB_1KG
JPT_1KG
CHS_1KG
CDX_1KG
KHV_1KG
OCE_CEPH
NAM_CEPH
PEL_1KG
MXL_1KG
CLM_1KG
PUR_1KG
0%
25%
50%
75%
100%
Haplotype frequency
Haplotypes
CCC
CCT
GCC
GCT
GTT

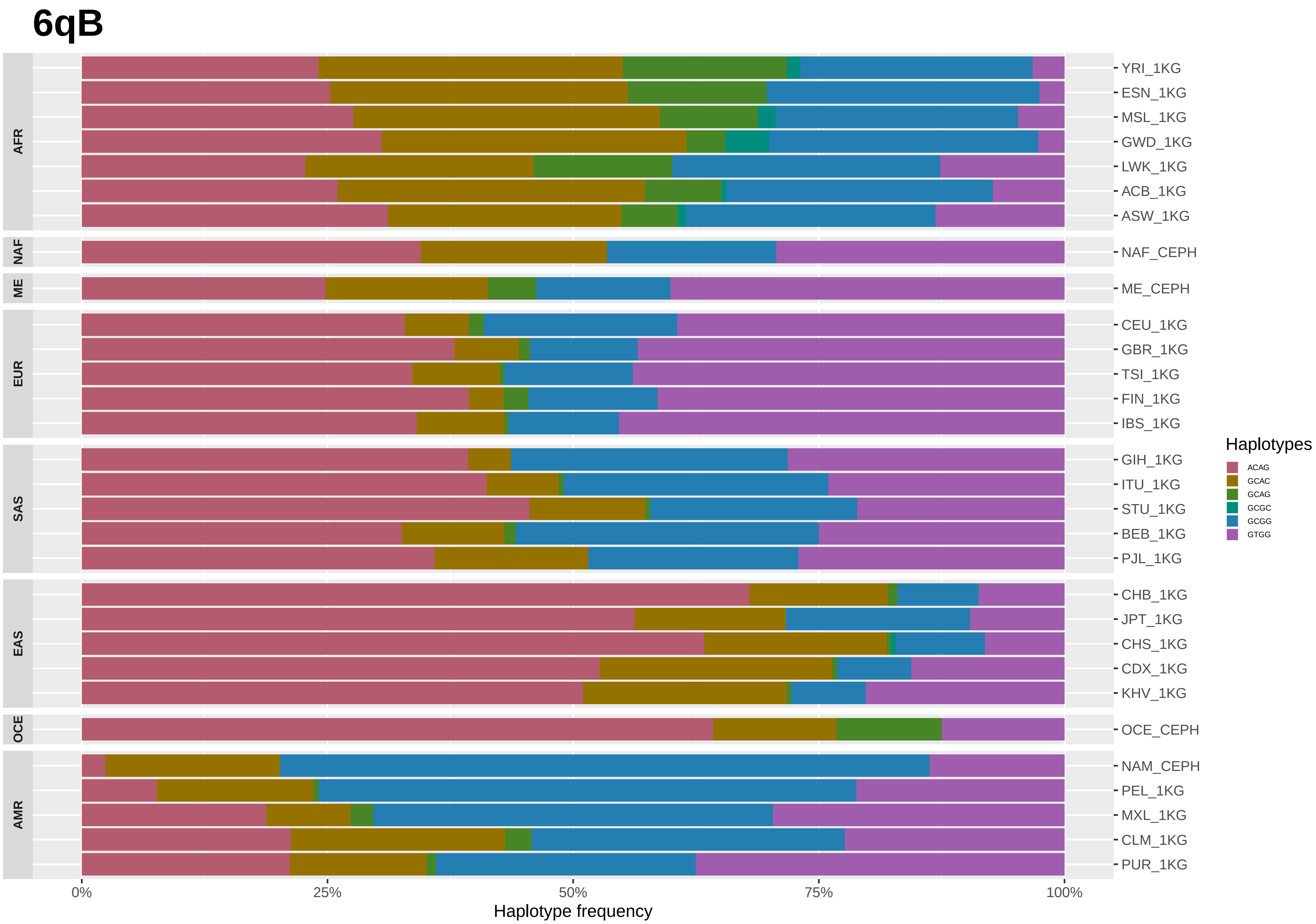
6qB
AFR
NAF
ME
EUR
SAS
EAS
OCE
AMR
YRI_1KG
ESN_1KG
MSL_1KG
GWD_1KG
LWK_1KG
ACB_1KG
ASW_1KG
NAF_CEPH
ME_CEPH
CEU_1KG
GBR_1KG
TSI_1KG
FIN_1KG
IBS_1KG
GIH_1KG
ITU_1KG
STU_1KG
BEB_1KG
PJL_1KG
CHB_1KG
JPT_1KG
CHS_1KG
CDX_1KG
KHV_1KG
OCE_CEPH
NAM_CEPH
PEL_1KG
MXL_1KG
CLM_1KG
PUR_1KG
0%
25%
50%
75%
100%
Haplotype frequency
Haplotypes
ACAG
GCAC
GCAG
GCGC
GCGG
GTGG

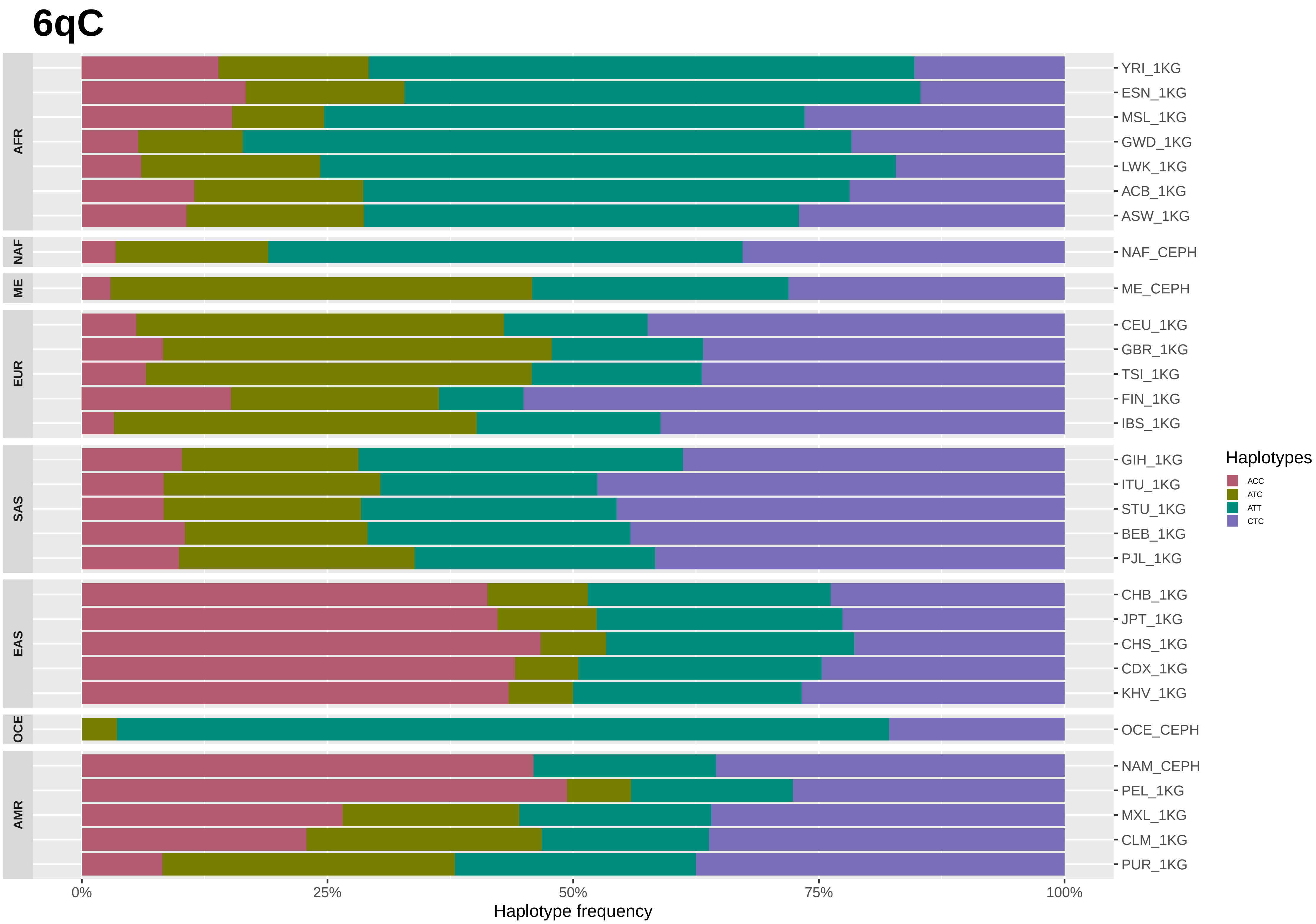

6qC
AFR
NAF
ME
EUR
SAS
EAS
OCE
AMR
YRI_1KG
ESN_1KG
MSL_1KG
GWD_1KG
LWK_1KG
ACB_1KG
ASW_1KG
NAF_CEPH
ME_CEPH
CEU_1KG
GBR_1KG
TSI_1KG
FIN_1KG
IBS_1KG
GIH_1KG
ITU_1KG
STU_1KG
BEB_1KG
PJL_1KG
CHB_1KG
JPT_1KG
CHS_1KG
CDX_1KG
KHV_1KG
OCE_CEPH
NAM_CEPH
PEL_1KG
MXL_1KG
CLM_1KG
PUR_1KG
0%
25%
50%
75%
100%
Haplotype frequency
Haplotypes
ACC
ATC
ATT
CTC

# 6qD

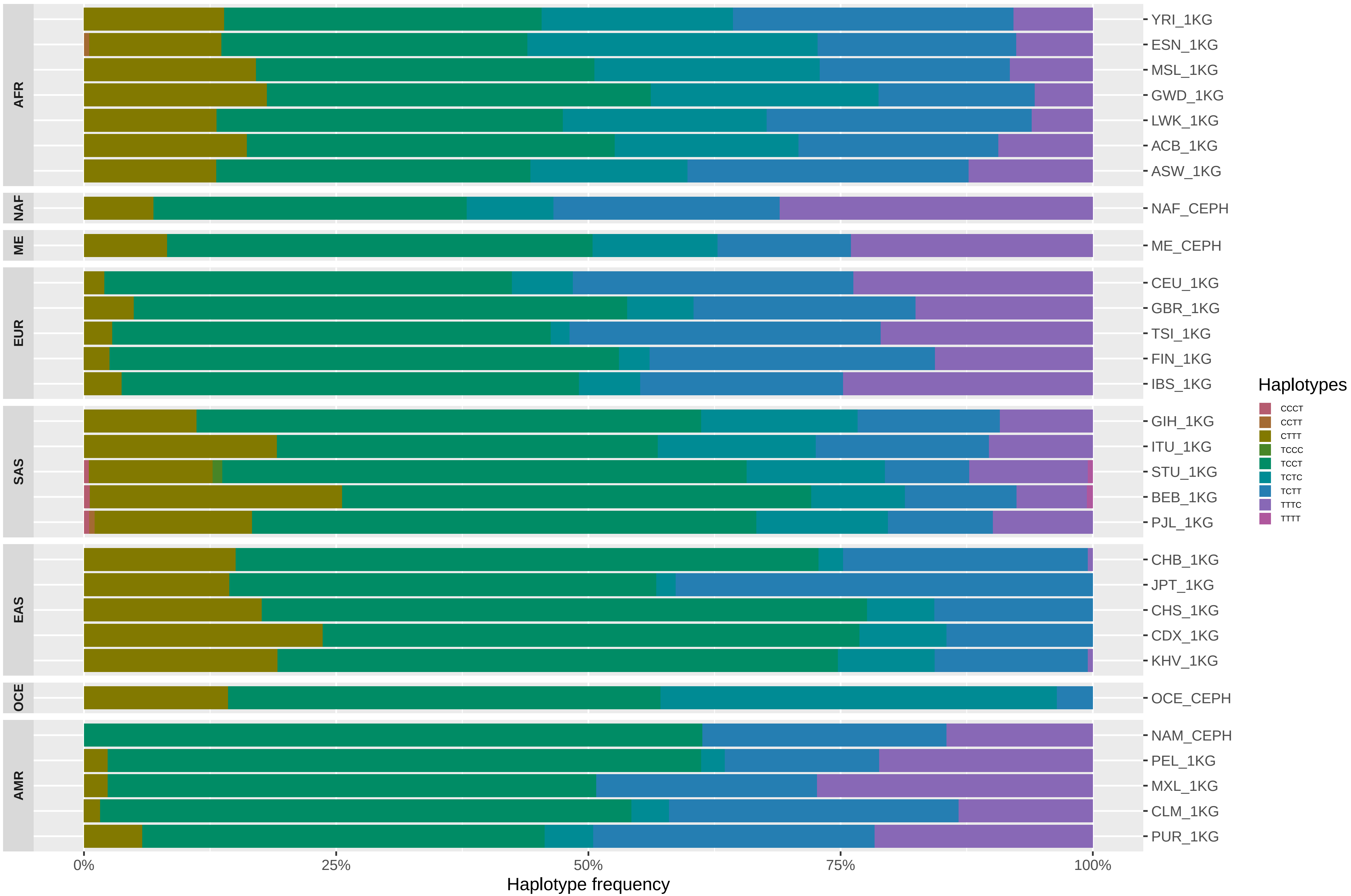

AFR
NAF
ME
EUR
SAS
EAS
OCE
AMR
YRI_1KG
ESN_1KG
MSL_1KG
GWD_1KG
LWK_1KG
ACB_1KG
ASW_1KG
NAF_CEPH
ME_CEPH
CEU_1KG
GBR_1KG
TSI_1KG
FIN_1KG
IBS_1KG
GIH_1KG
ITU_1KG
STU_1KG
BEB_1KG
PJL_1KG
CHB_1KG
JPT_1KG
CHS_1KG
CDX_1KG
KHV_1KG
OCE_CEPH
NAM_CEPH
PEL_1KG
MXL_1KG
CLM_1KG
PUR_1KG
0%
25%
50%
75%
100%
Haplotype frequency
Haplotypes
CCCT
CCTT
CTTT
TCCC
TCCT
TCTC
TCTT
TTTC
TTTT

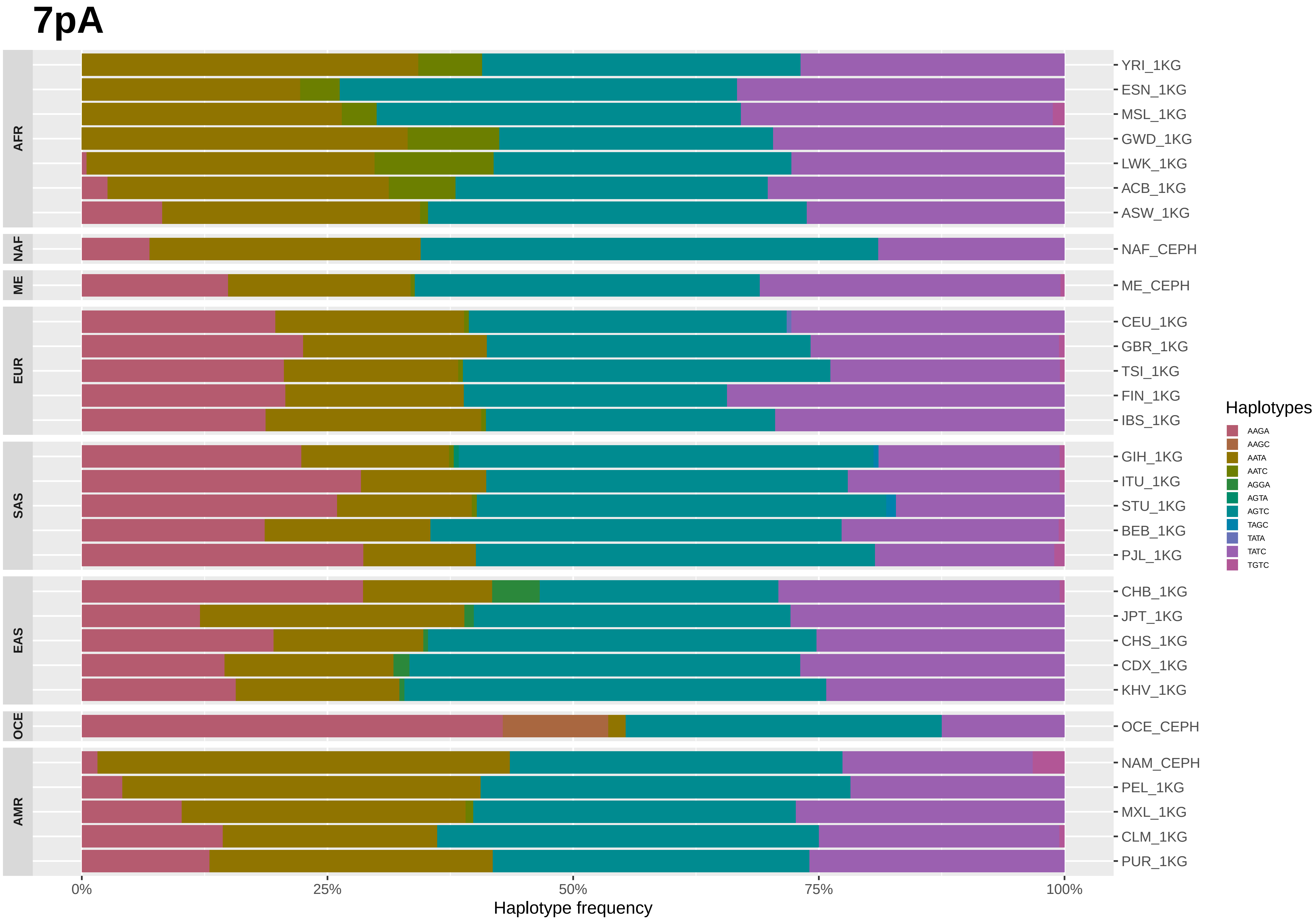

7pA
AFR
NAF
ME
EUR
SAS
EAS
OCE
AMR
YRI_1KG
ESN_1KG
MSL_1KG
GWD_1KG
LWK_1KG
ACB_1KG
ASW_1KG
NAF_CEPH
ME_CEPH
CEU_1KG
GBR_1KG
TSI_1KG
FIN_1KG
IBS_1KG
GIH_1KG
ITU_1KG
STU_1KG
BEB_1KG
PJL_1KG
CHB_1KG
JPT_1KG
CHS_1KG
CDX_1KG
KHV_1KG
OCE_CEPH
NAM_CEPH
PEL_1KG
MXL_1KG
CLM_1KG
PUR_1KG
0%
25%
50%
75%
100%
Haplotype frequency
Haplotypes
AAGA
AAGC
AATA
AATC
AGGA
AGTA
AGTC
TAGC
TATA
TATC
TGTC

# 7pB

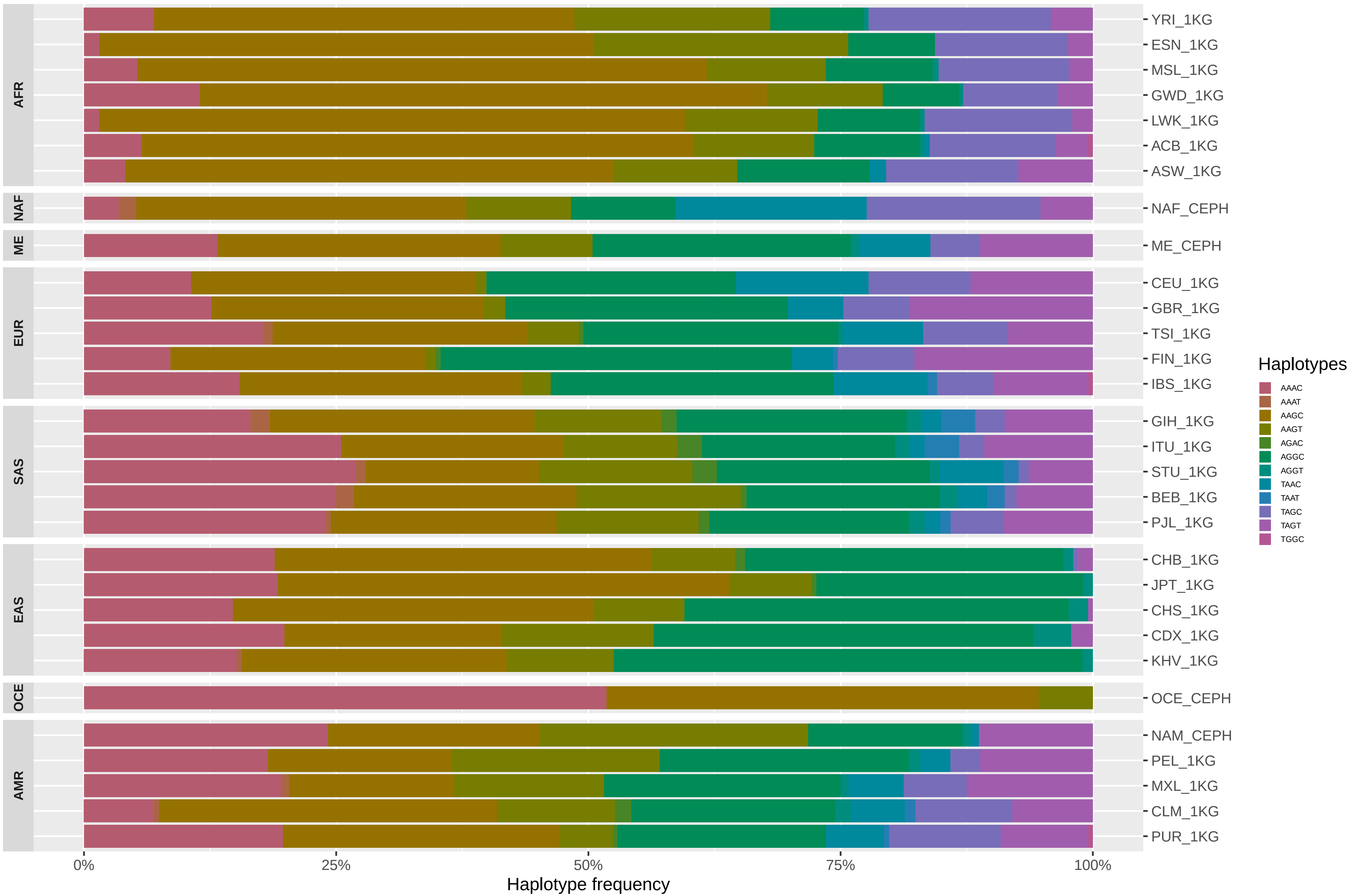

AFR
NAF
ME
EUR
SAS
EAS
OCE
AMR
YRI_1KG
ESN_1KG
MSL_1KG
GWD_1KG
LWK_1KG
ACB_1KG
ASW_1KG
NAF_CEPH
ME_CEPH
CEU_1KG
GBR_1KG
TSI_1KG
FIN_1KG
IBS_1KG
GIH_1KG
ITU_1KG
STU_1KG
BEB_1KG
PJL_1KG
CHB_1KG
JPT_1KG
CHS_1KG
CDX_1KG
KHV_1KG
OCE_CEPH
NAM_CEPH
PEL_1KG
MXL_1KG
CLM_1KG
PUR_1KG
0%
25%
50%
75%
100%
Haplotype frequency
Haplotypes
AAAC
AAAT
AAGC
AAGT
AGAC
AGGC
AGGT
TAAC
TAAT
TAGC
TAGT
TGGC

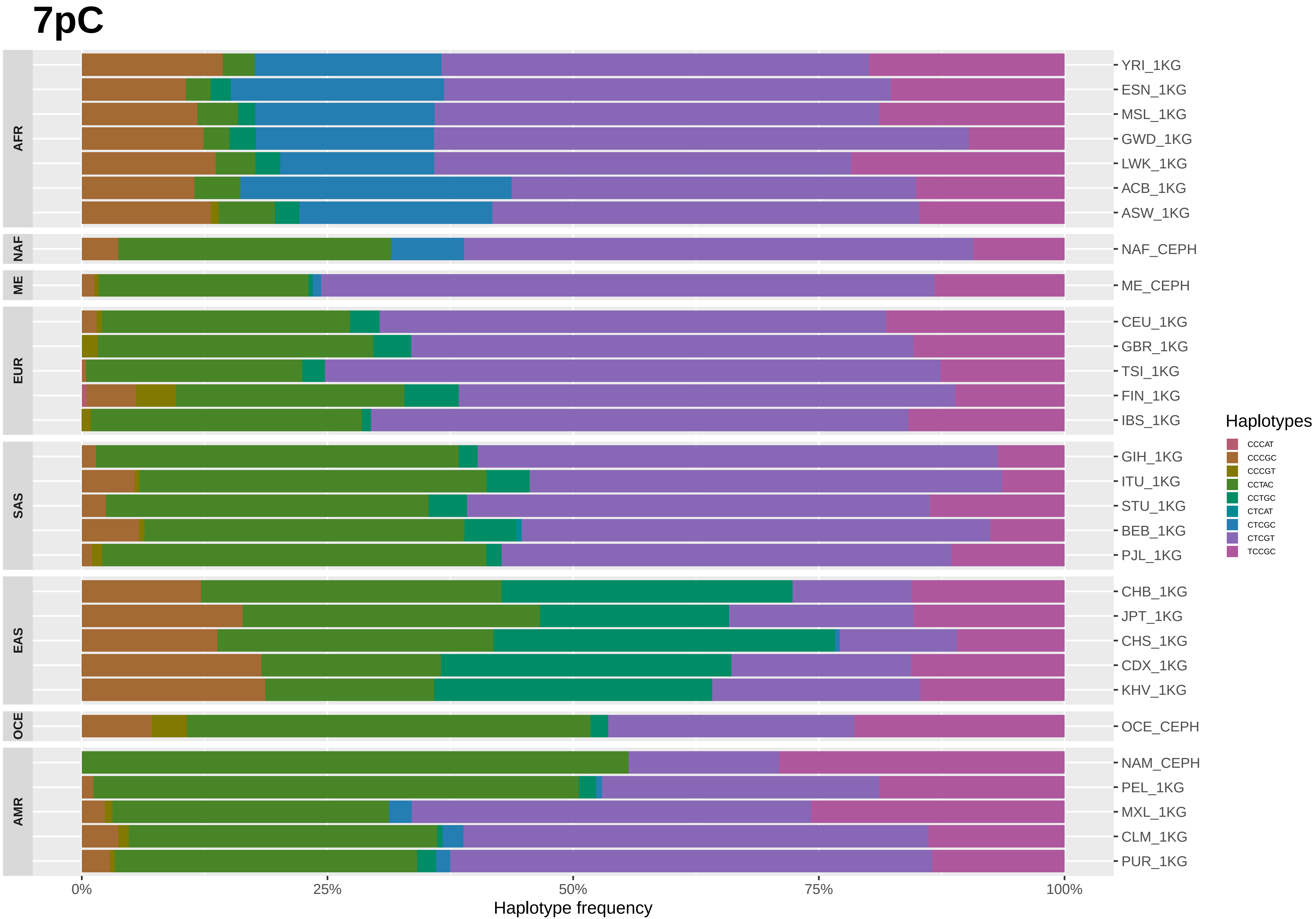

7pC
AFR
NAF
ME
EUR
SAS
EAS
OCE
AMR
YRI_1KG
ESN_1KG
MSL_1KG
GWD_1KG
LWK_1KG
ACB_1KG
ASW_1KG
NAF_CEPH
ME_CEPH
CEU_1KG
GBR_1KG
TSI_1KG
FIN_1KG
IBS_1KG
GIH_1KG
ITU_1KG
STU_1KG
BEB_1KG
PJL_1KG
CHB_1KG
JPT_1KG
CHS_1KG
CDX_1KG
KHV_1KG
OCE_CEPH
NAM_CEPH
PEL_1KG
MXL_1KG
CLM_1KG
PUR_1KG
0%
25%
50%
75%
100%
Haplotype frequency
Haplotypes
CCCAT
CCCGC
CCCGT
CCTAC
CCTGC
CTCAT
CTCGC
CTCGT
TCCGC

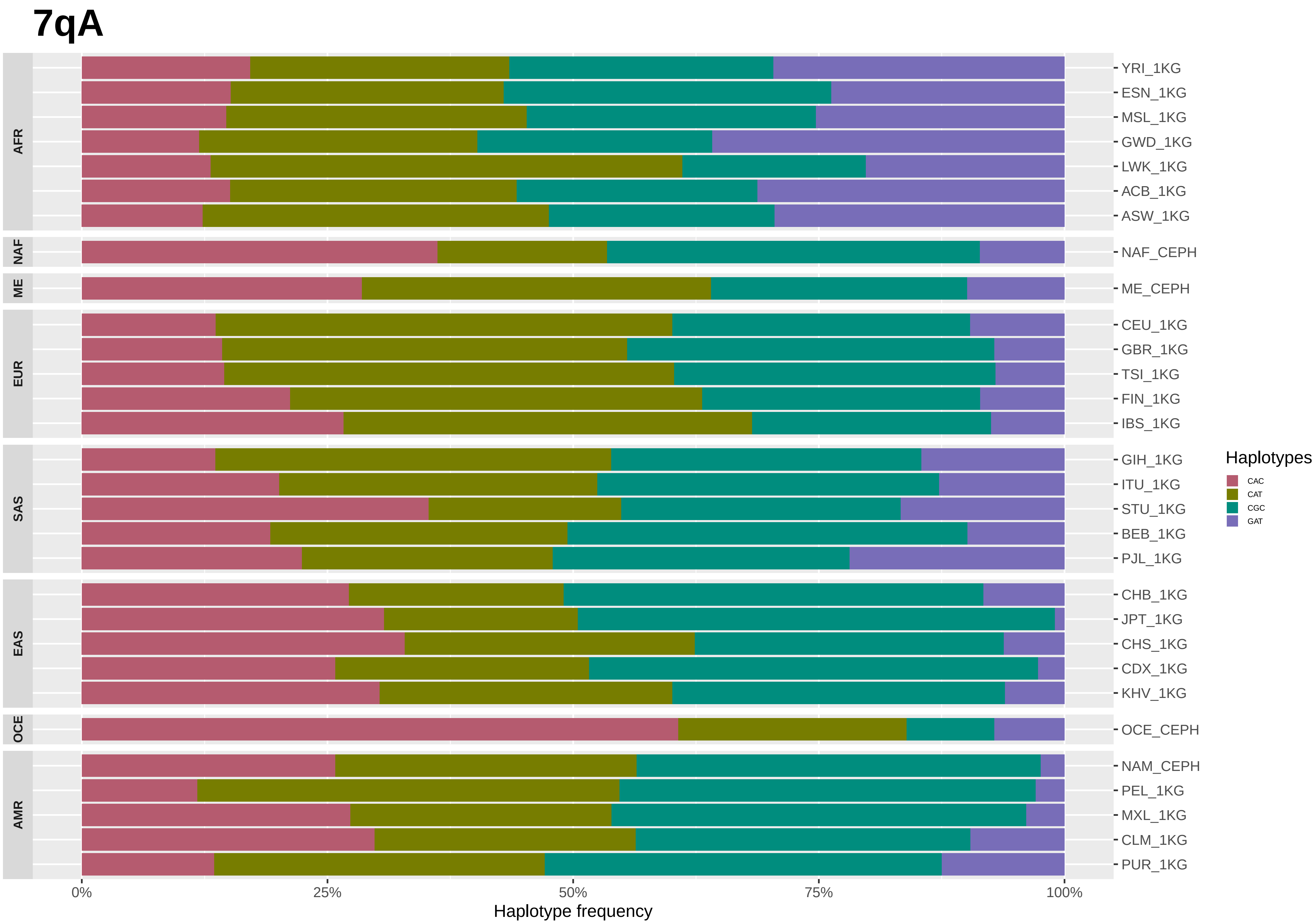

7qA
AFR
NAF
ME
EUR
SAS
EAS
OCE
AMR
YRI_1KG
ESN_1KG
MSL_1KG
GWD_1KG
LWK_1KG
ACB_1KG
ASW_1KG
NAF_CEPH
ME_CEPH
CEU_1KG
GBR_1KG
TSI_1KG
FIN_1KG
IBS_1KG
GIH_1KG
ITU_1KG
STU_1KG
BEB_1KG
PJL_1KG
CHB_1KG
JPT_1KG
CHS_1KG
CDX_1KG
KHV_1KG
OCE_CEPH
NAM_CEPH
PEL_1KG
MXL_1KG
CLM_1KG
PUR_1KG
0%
25%
50%
75%
100%
Haplotype frequency
Haplotypes
CAC
CAT
CGC
GAT

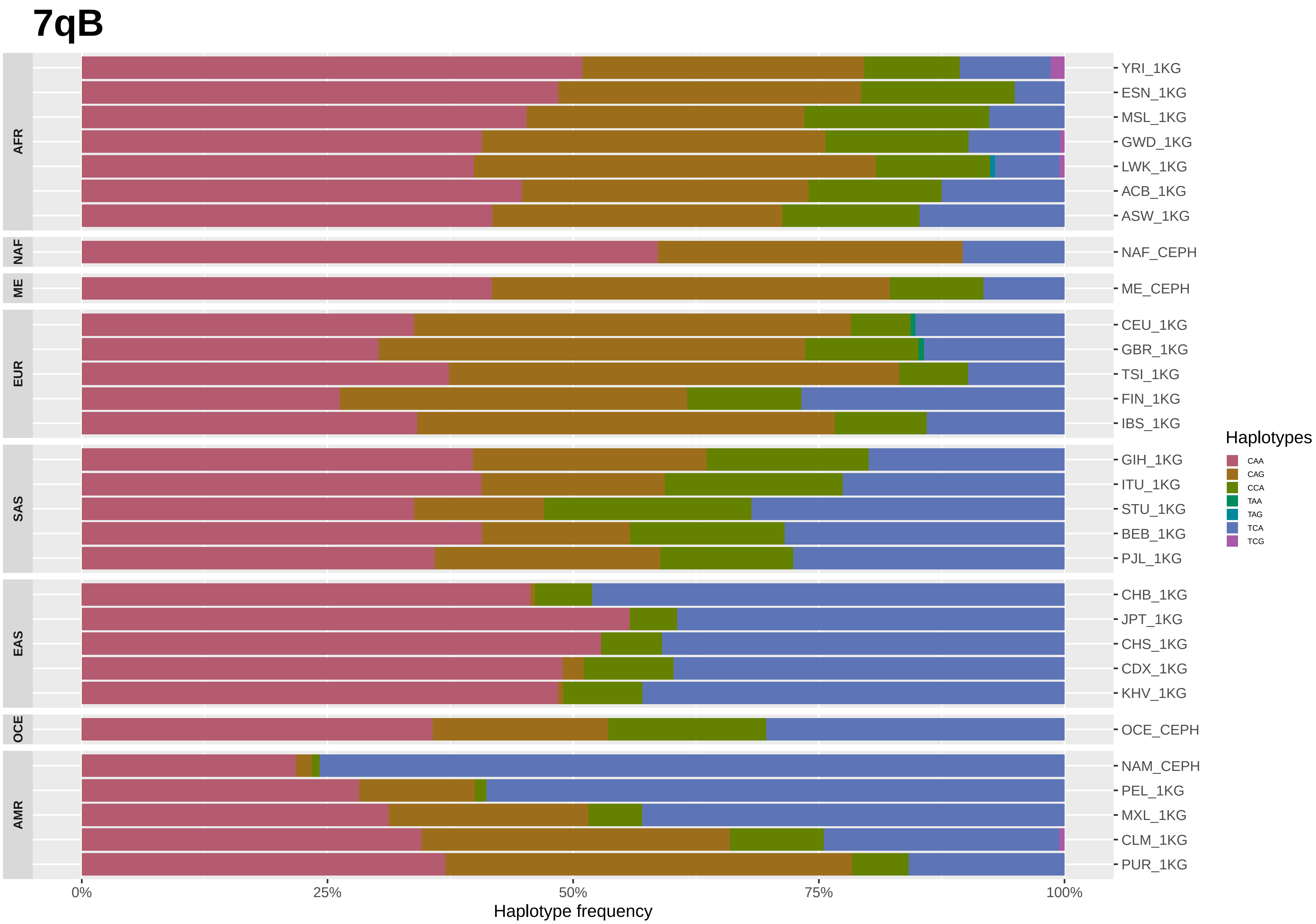

7qB
AFR
NAF
ME
EUR
SAS
EAS
OCE
AMR
YRI_1KG
ESN_1KG
MSL_1KG
GWD_1KG
LWK_1KG
ACB_1KG
ASW_1KG
NAF_CEPH
ME_CEPH
CEU_1KG
GBR_1KG
TSI_1KG
FIN_1KG
IBS_1KG
GIH_1KG
ITU_1KG
STU_1KG
BEB_1KG
PJL_1KG
CHB_1KG
JPT_1KG
CHS_1KG
CDX_1KG
KHV_1KG
OCE_CEPH
NAM_CEPH
PEL_1KG
MXL_1KG
CLM_1KG
PUR_1KG
0%
25%
50%
75%
100%
Haplotype frequency
Haplotypes
CAA
CAG
CCA
TAA
TAG
TCA
TCG

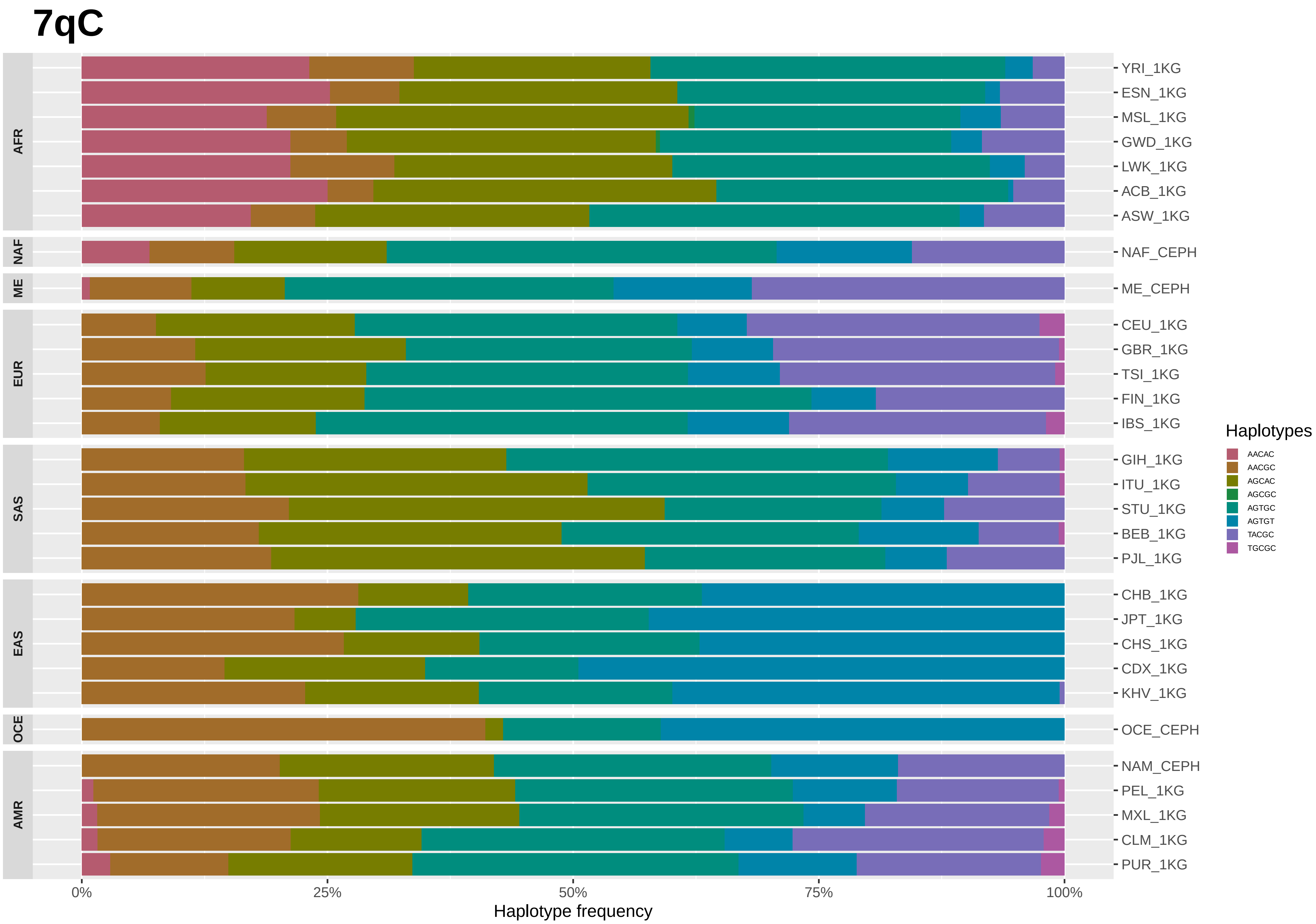
7qC
AFR
NAF
ME
EUR
SAS
EAS
OCE
AMR
YRI_1KG
ESN_1KG
MSL_1KG
GWD_1KG
LWK_1KG
ACB_1KG
ASW_1KG
NAF_CEPH
ME_CEPH
CEU_1KG
GBR_1KG
TSI_1KG
FIN_1KG
IBS_1KG
GIH_1KG
ITU_1KG
STU_1KG
BEB_1KG
PJL_1KG
CHB_1KG
JPT_1KG
CHS_1KG
CDX_1KG
KHV_1KG
OCE_CEPH
NAM_CEPH
PEL_1KG
MXL_1KG
CLM_1KG
PUR_1KG
0%
25%
50%
75%
100%
Haplotype frequency
Haplotypes
AACAC
AACGC
AGCAC
AGCGC
AGTGC
AGTGT
TACGC
TGCGC

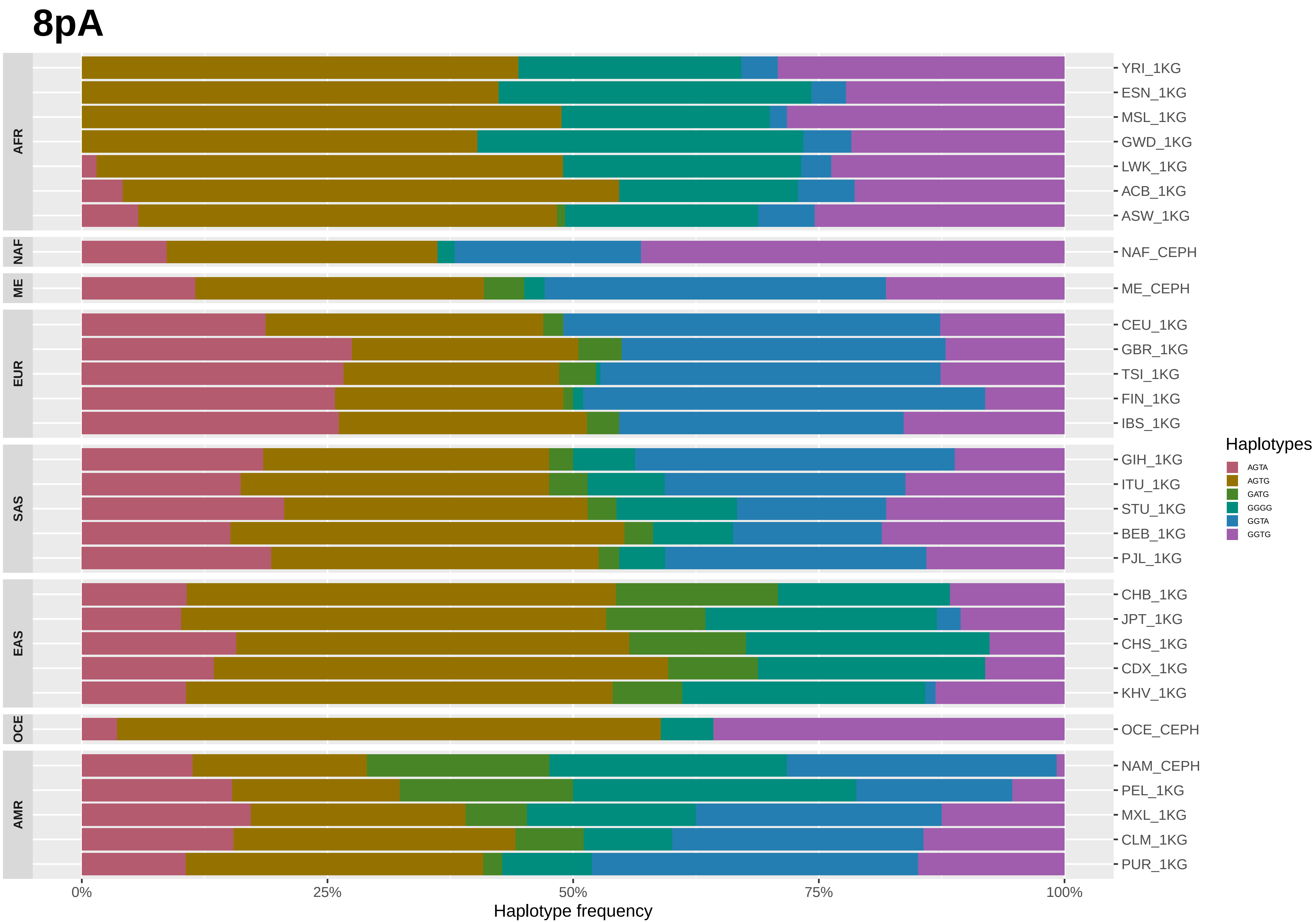
8pA
AFR
NAF
ME
EUR
SAS
EAS
OCE
AMR
YRI_1KG
ESN_1KG
MSL_1KG
GWD_1KG
LWK_1KG
ACB_1KG
ASW_1KG
NAF_CEPH
ME_CEPH
CEU_1KG
GBR_1KG
TSI_1KG
FIN_1KG
IBS_1KG
GIH_1KG
ITU_1KG
STU_1KG
BEB_1KG
PJL_1KG
CHB_1KG
JPT_1KG
CHS_1KG
CDX_1KG
KHV_1KG
OCE_CEPH
NAM_CEPH
PEL_1KG
MXL_1KG
CLM_1KG
PUR_1KG
0%
25%
50%
75%
100%
Haplotype frequency
Haplotypes
AGTA
AGTG
GATG
GGGG
GGTA
GGTG

# 8pB

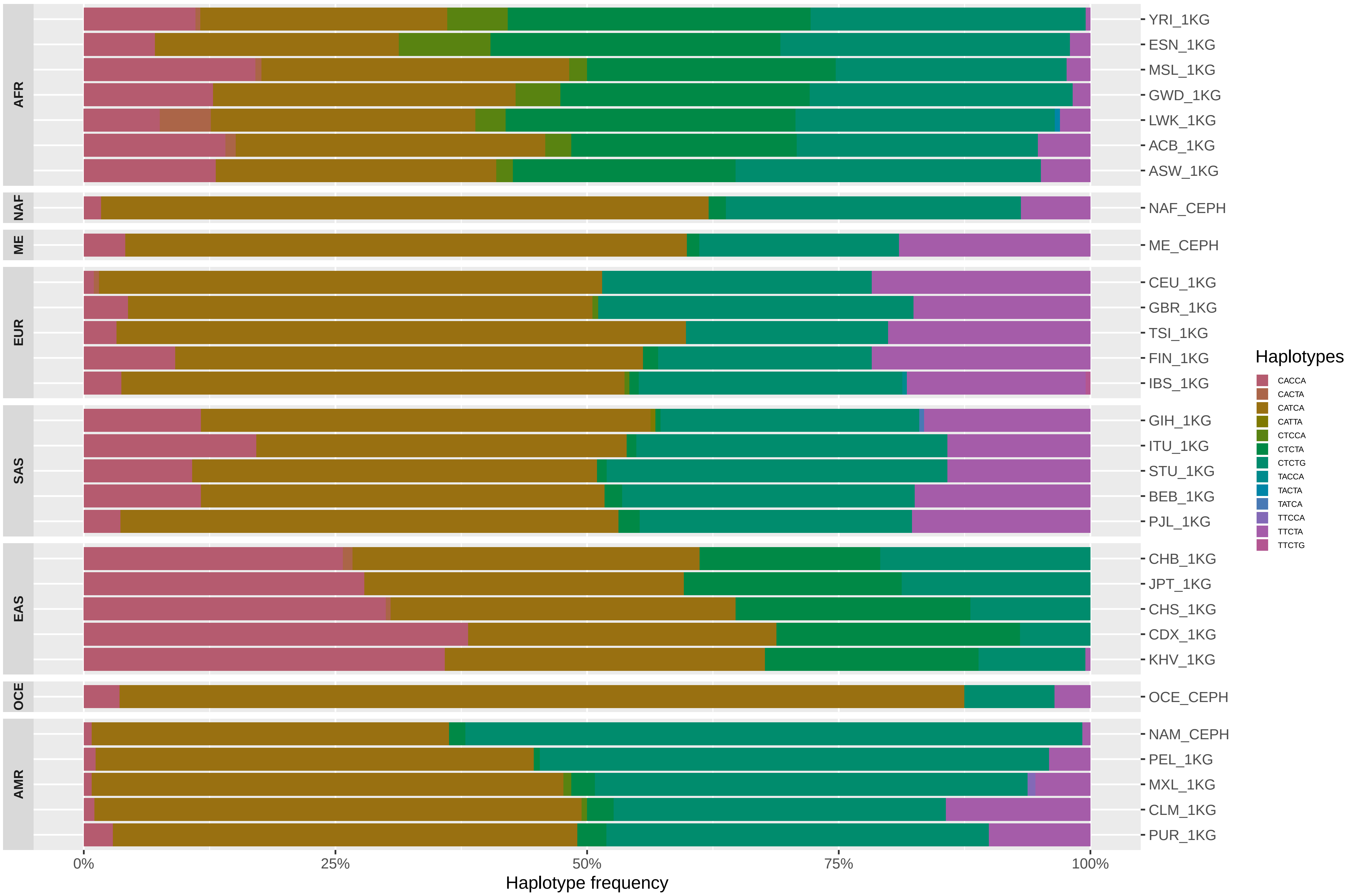

AFR
NAF
ME
EUR
SAS
EAS
OCE
AMR
YRI_1KG
ESN_1KG
MSL_1KG
GWD_1KG
LWK_1KG
ACB_1KG
ASW_1KG
NAF_CEPH
ME_CEPH
CEU_1KG
GBR_1KG
TSI_1KG
FIN_1KG
IBS_1KG
GIH_1KG
ITU_1KG
STU_1KG
BEB_1KG
PJL_1KG
CHB_1KG
JPT_1KG
CHS_1KG
CDX_1KG
KHV_1KG
OCE_CEPH
NAM_CEPH
PEL_1KG
MXL_1KG
CLM_1KG
PUR_1KG
0%
25%
50%
75%
100%
Haplotype frequency
Haplotypes
CACCA
CACTA
CATCA
CATTA
CTCCA
CTCTA
CTCTG
TACCA
TACTA
TATCA
TTCCA
TTCTA
TTCTG

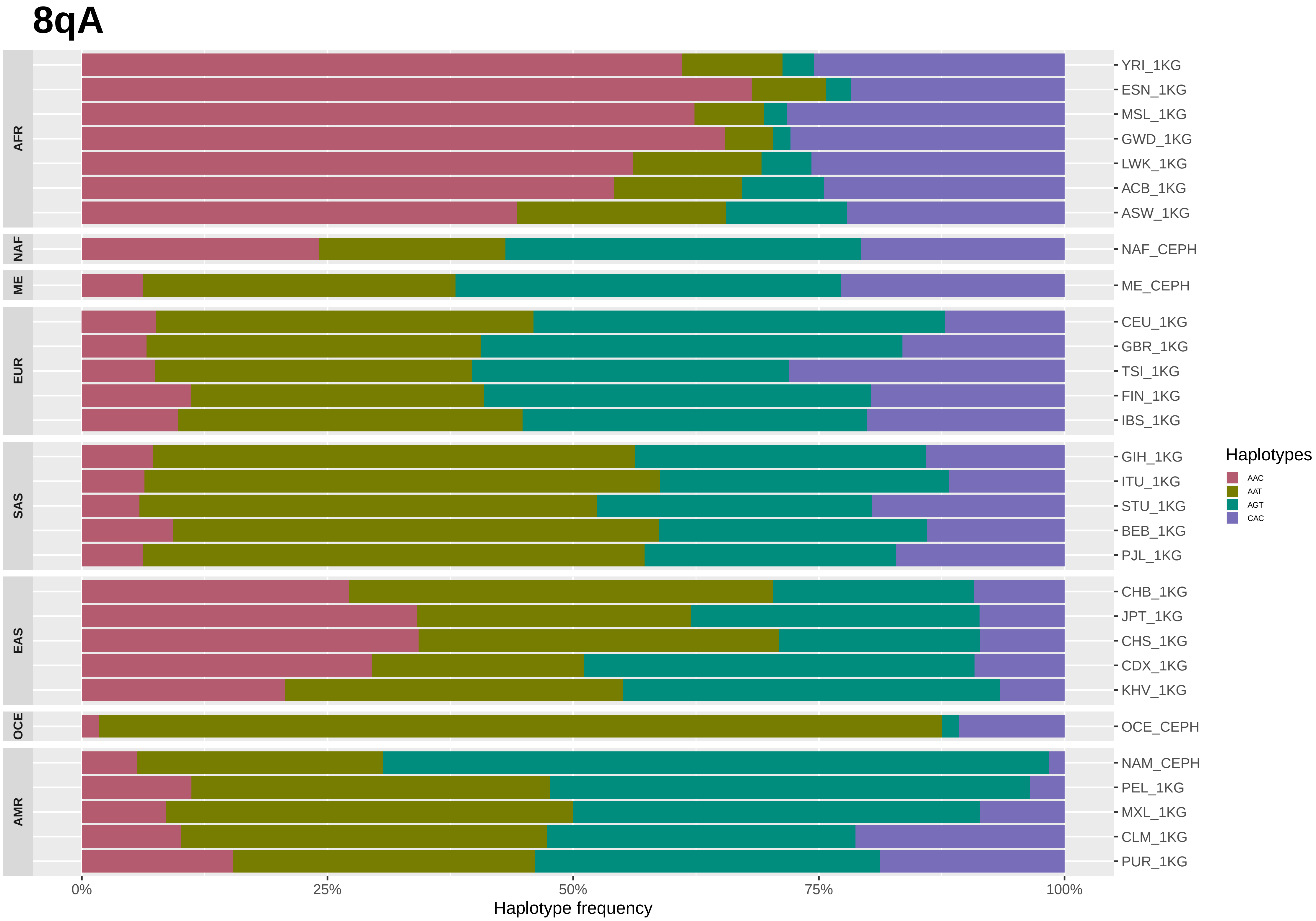

8qA
AFR
NAF
ME
EUR
SAS
EAS
OCE
AMR
YRI_1KG
ESN_1KG
MSL_1KG
GWD_1KG
LWK_1KG
ACB_1KG
ASW_1KG
NAF_CEPH
ME_CEPH
CEU_1KG
GBR_1KG
TSI_1KG
FIN_1KG
IBS_1KG
GIH_1KG
ITU_1KG
STU_1KG
BEB_1KG
PJL_1KG
CHB_1KG
JPT_1KG
CHS_1KG
CDX_1KG
KHV_1KG
OCE_CEPH
NAM_CEPH
PEL_1KG
MXL_1KG
CLM_1KG
PUR_1KG
0%
25%
50%
75%
100%
Haplotype frequency
Haplotypes
AAC
AAT
AGT
CAC

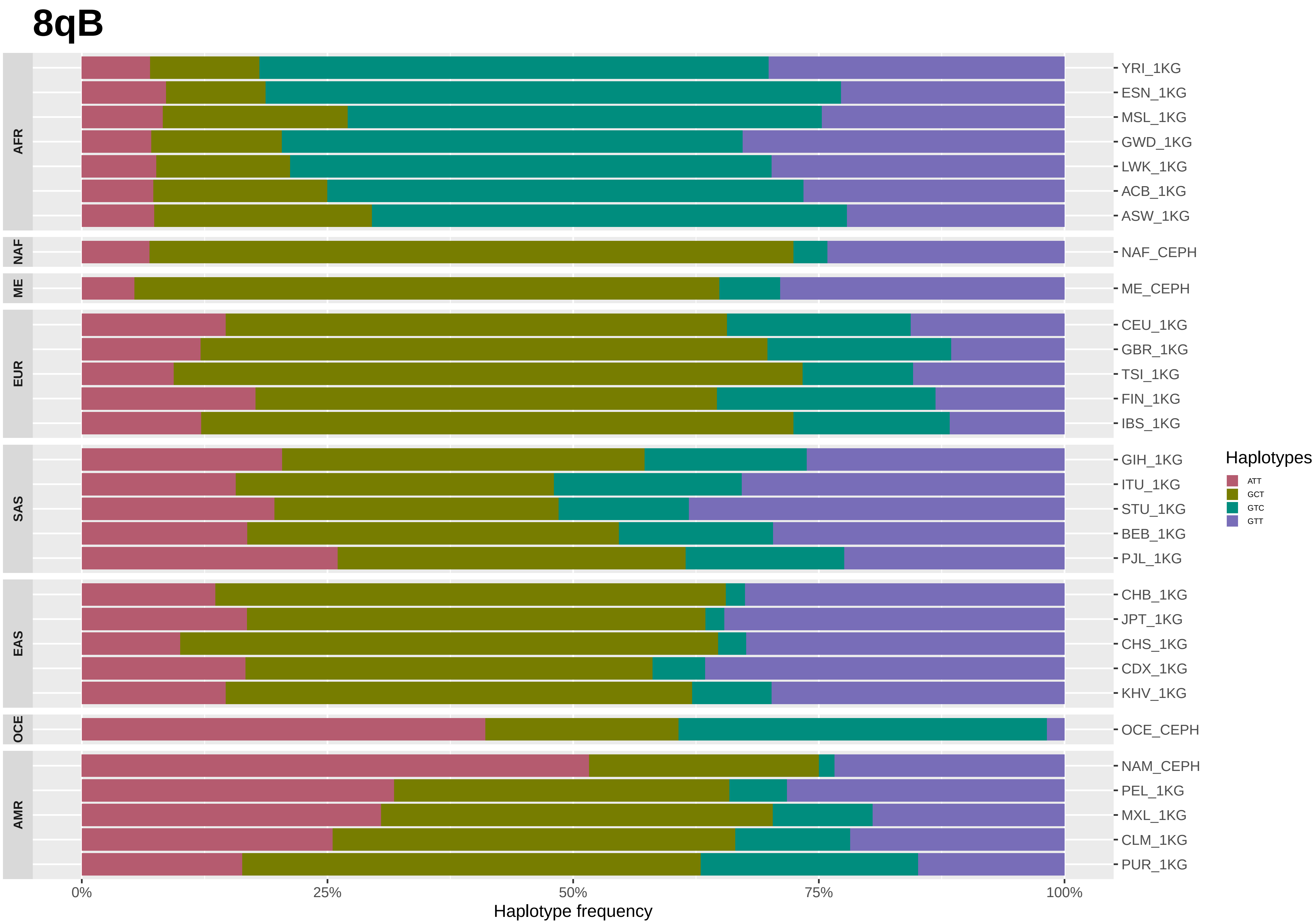

8qB
AFR
NAF
ME
EUR
SAS
EAS
OCE
AMR
YRI_1KG
ESN_1KG
MSL_1KG
GWD_1KG
LWK_1KG
ACB_1KG
ASW_1KG
NAF_CEPH
ME_CEPH
CEU_1KG
GBR_1KG
TSI_1KG
FIN_1KG
IBS_1KG
GIH_1KG
ITU_1KG
STU_1KG
BEB_1KG
PJL_1KG
CHB_1KG
JPT_1KG
CHS_1KG
CDX_1KG
KHV_1KG
OCE_CEPH
NAM_CEPH
PEL_1KG
MXL_1KG
CLM_1KG
PUR_1KG
0%
25%
50%
75%
100%
Haplotype frequency
Haplotypes
ATT
GCT
GTC
GTT

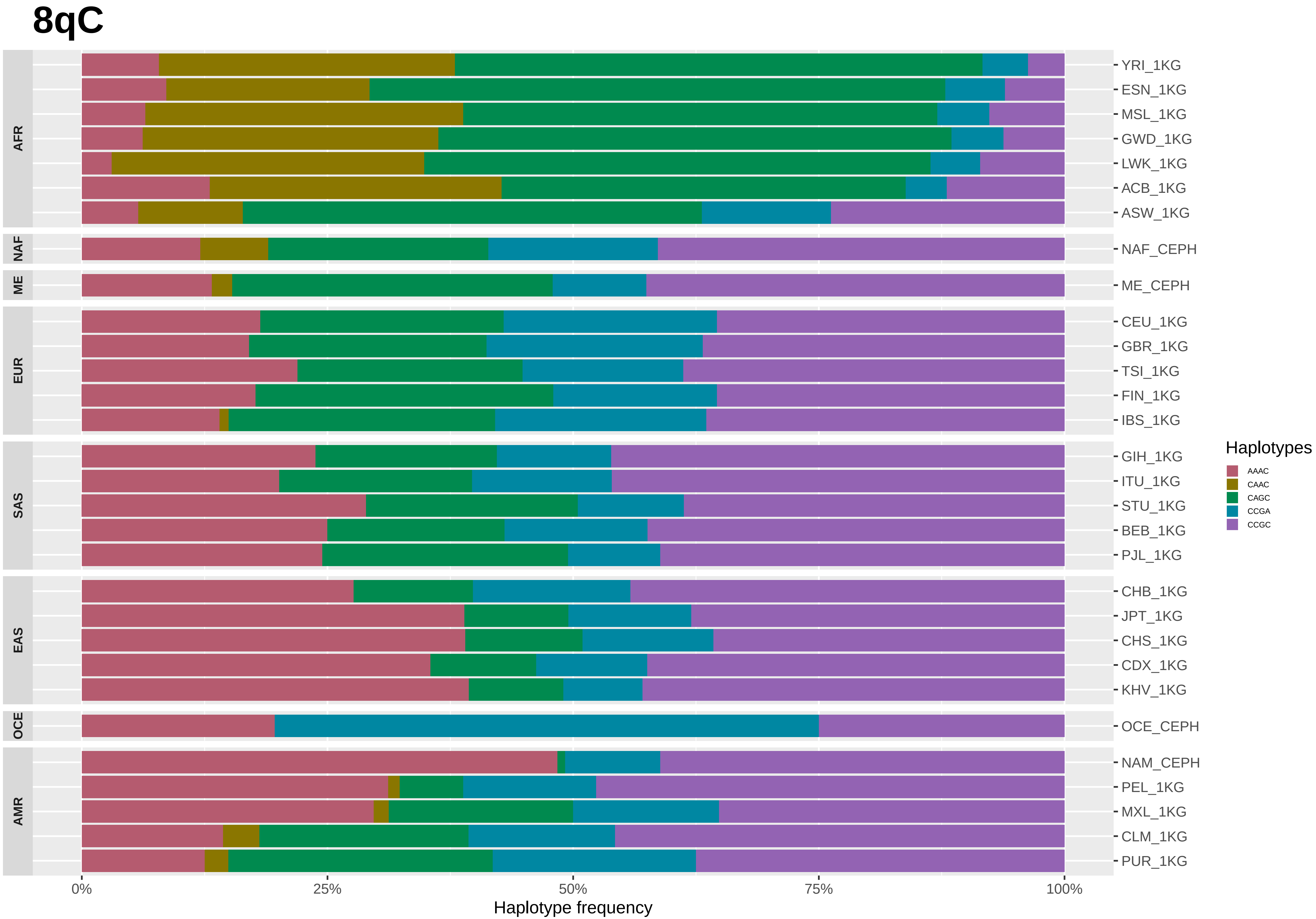
8qC
AFR
NAF
ME
EUR
SAS
EAS
OCE
AMR
YRI_1KG
ESN_1KG
MSL_1KG
GWD_1KG
LWK_1KG
ACB_1KG
ASW_1KG
NAF_CEPH
ME_CEPH
CEU_1KG
GBR_1KG
TSI_1KG
FIN_1KG
IBS_1KG
GIH_1KG
ITU_1KG
STU_1KG
BEB_1KG
PJL_1KG
CHB_1KG
JPT_1KG
CHS_1KG
CDX_1KG
KHV_1KG
OCE_CEPH
NAM_CEPH
PEL_1KG
MXL_1KG
CLM_1KG
PUR_1KG
0%
25%
50%
75%
100%
Haplotype frequency
Haplotypes
AAAC
CAAC
CAGC
CCGA
CCGC

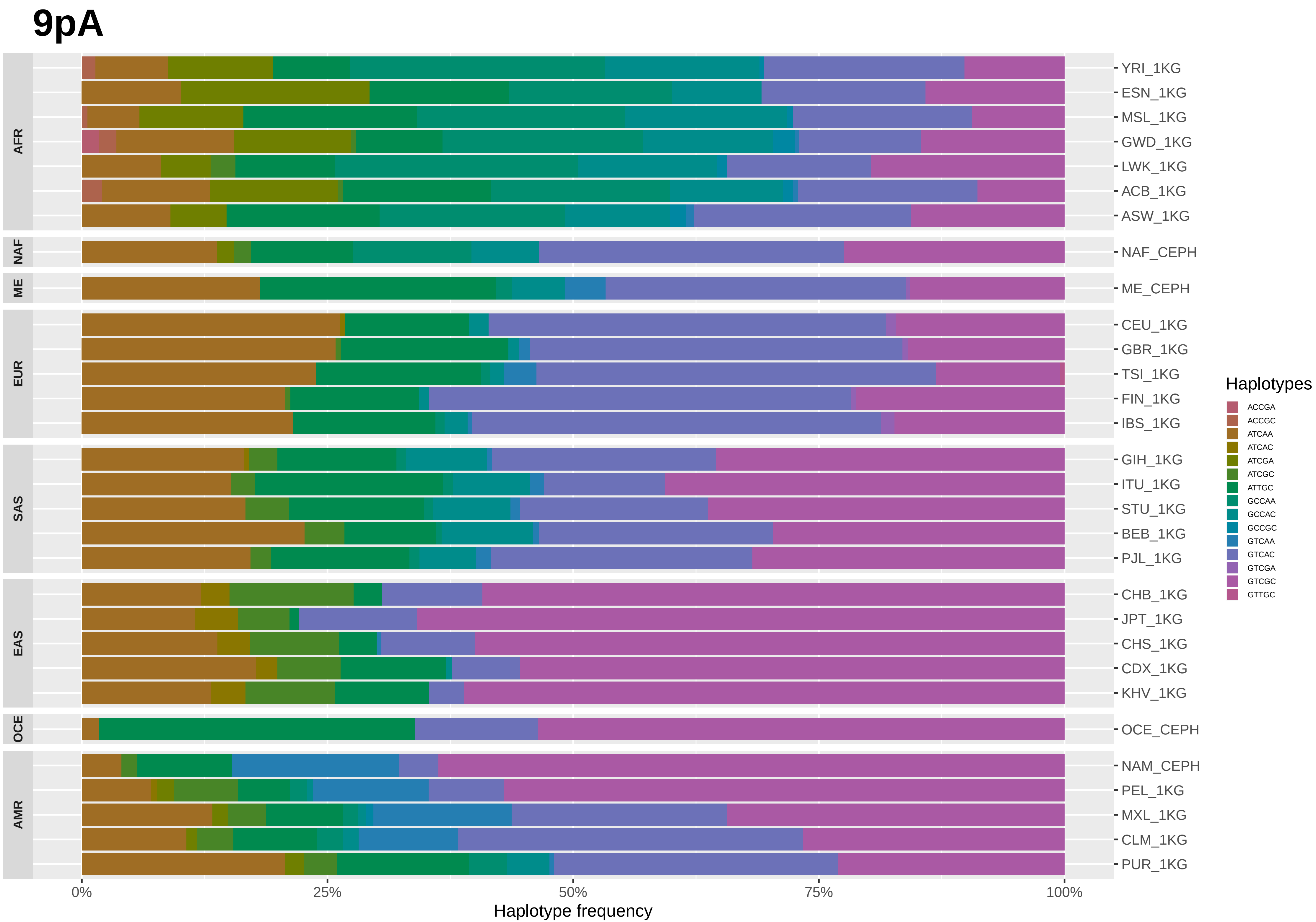
9pA
AFR
NAF
ME
EUR
SAS
EAS
OCE
AMR
YRI_1KG
ESN_1KG
MSL_1KG
GWD_1KG
LWK_1KG
ACB_1KG
ASW_1KG
NAF_CEPH
ME_CEPH
CEU_1KG
GBR_1KG
TSI_1KG
FIN_1KG
IBS_1KG
GIH_1KG
ITU_1KG
STU_1KG
BEB_1KG
PJL_1KG
CHB_1KG
JPT_1KG
CHS_1KG
CDX_1KG
KHV_1KG
OCE_CEPH
NAM_CEPH
PEL_1KG
MXL_1KG
CLM_1KG
PUR_1KG
0%
25%
50%
75%
100%
Haplotype frequency
Haplotypes
ACCGA
ACCGC
ATCAA
ATCAC
ATCGA
ATCGC
ATTGC
GCCAA
GCCAC
GCCGC
GTCAA
GTCAC
GTCGA
GTCGC
GTTGC

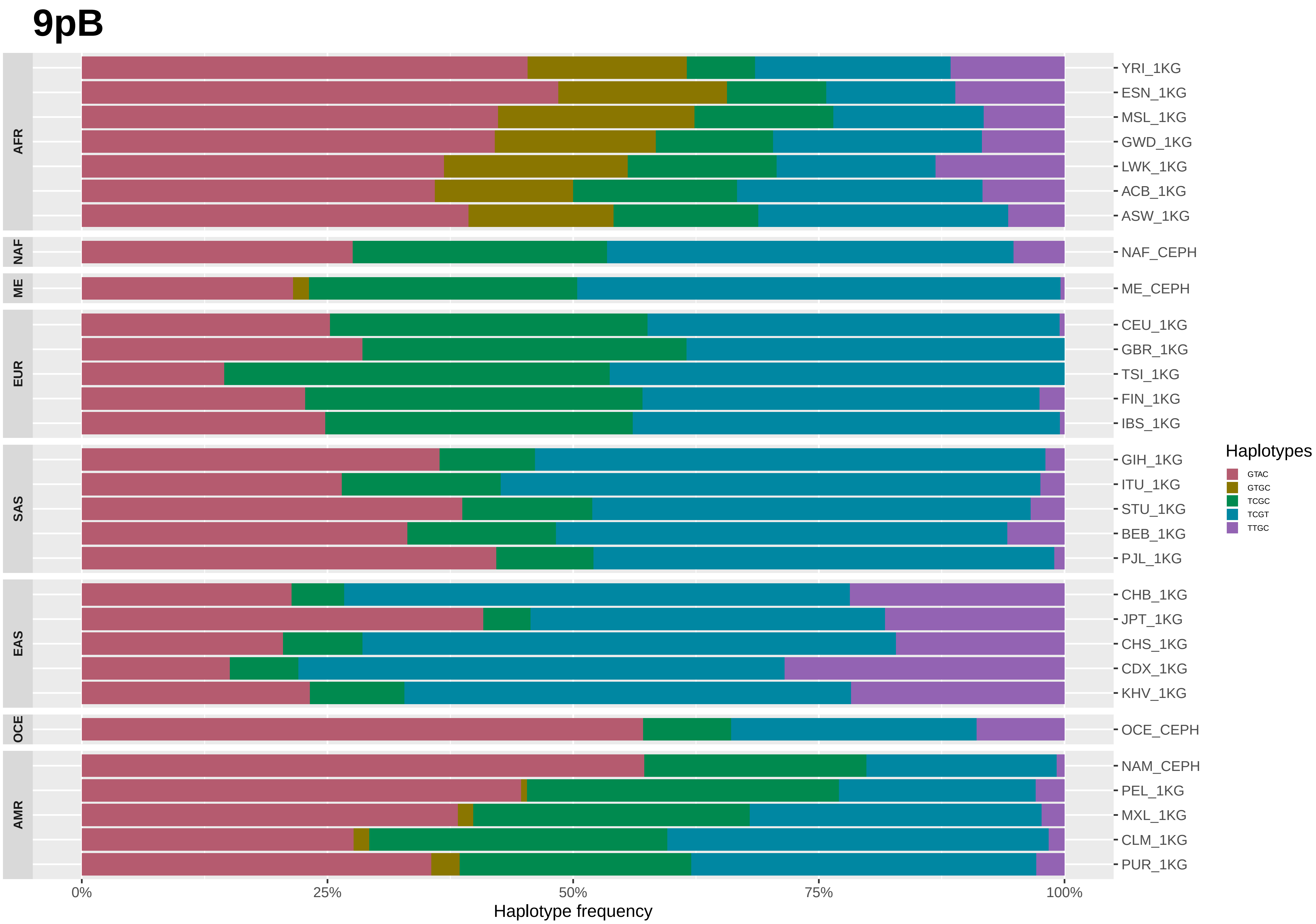

9pB
AFR
NAF
ME
EUR
SAS
EAS
OCE
AMR
YRI_1KG
ESN_1KG
MSL_1KG
GWD_1KG
LWK_1KG
ACB_1KG
ASW_1KG
NAF_CEPH
ME_CEPH
CEU_1KG
GBR_1KG
TSI_1KG
FIN_1KG
IBS_1KG
GIH_1KG
ITU_1KG
STU_1KG
BEB_1KG
PJL_1KG
CHB_1KG
JPT_1KG
CHS_1KG
CDX_1KG
KHV_1KG
OCE_CEPH
NAM_CEPH
PEL_1KG
MXL_1KG
CLM_1KG
PUR_1KG
0%
25%
50%
75%
100%
Haplotype frequency
Haplotypes
GTAC
GTGC
TCGC
TCGT
TTGC

# 9qA

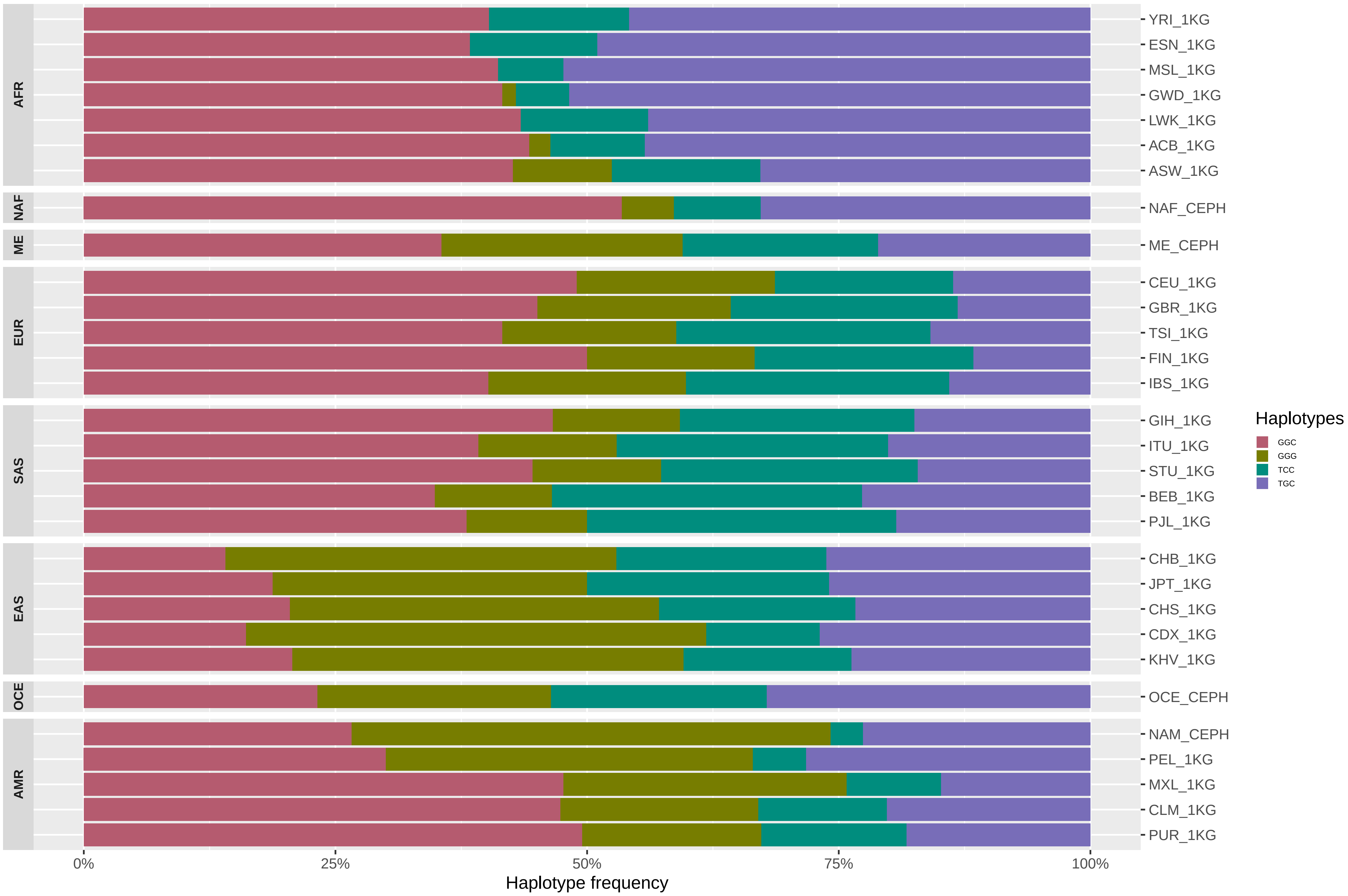

AFR
NAF
ME
EUR
SAS
EAS
OCE
AMR
YRI_1KG
ESN_1KG
MSL_1KG
GWD_1KG
LWK_1KG
ACB_1KG
ASW_1KG
NAF_CEPH
ME_CEPH
CEU_1KG
GBR_1KG
TSI_1KG
FIN_1KG
IBS_1KG
GIH_1KG
ITU_1KG
STU_1KG
BEB_1KG
PJL_1KG
CHB_1KG
JPT_1KG
CHS_1KG
CDX_1KG
KHV_1KG
OCE_CEPH
NAM_CEPH
PEL_1KG
MXL_1KG
CLM_1KG
PUR_1KG
0%
25%
50%
75%
100%
Haplotype frequency
Haplotypes
GGC
GGG
TCC
TGC

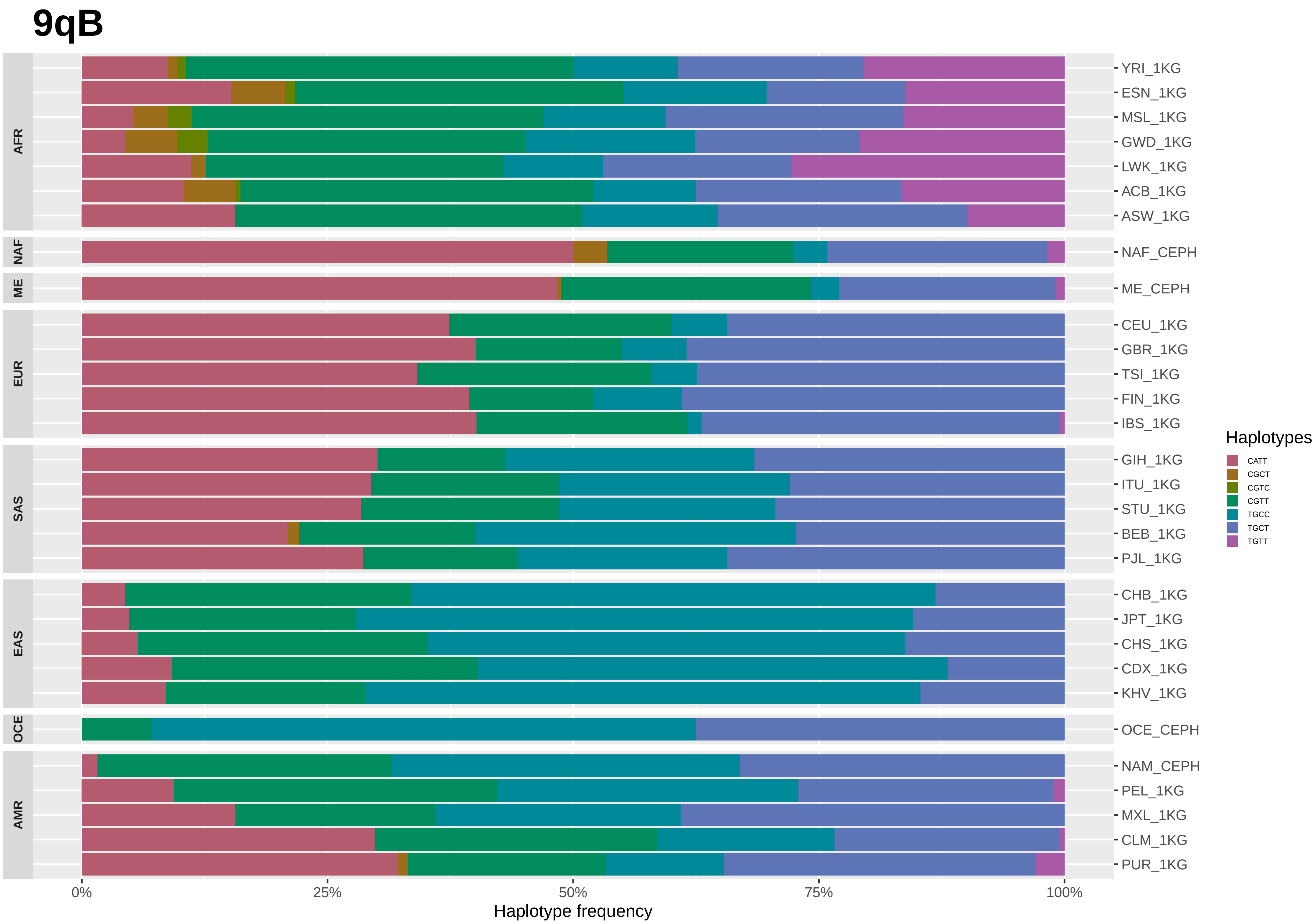
9qB
AFR
NAF
ME
EUR
SAS
EAS
OCE
AMR
YRI_1KG
ESN_1KG
MSL_1KG
GWD_1KG
LWK_1KG
ACB_1KG
ASW_1KG
NAF_CEPH
ME_CEPH
CEU_1KG
GBR_1KG
TSI_1KG
FIN_1KG
IBS_1KG
GIH_1KG
ITU_1KG
STU_1KG
BEB_1KG
PJL_1KG
CHB_1KG
JPT_1KG
CHS_1KG
CDX_1KG
KHV_1KG
OCE_CEPH
NAM_CEPH
PEL_1KG
MXL_1KG
CLM_1KG
PUR_1KG
0%
25%
50%
75%
100%
Haplotype frequency
Haplotypes
CATT
CGCT
CGTC
CGTT
TGCC
TGCT
TGTT

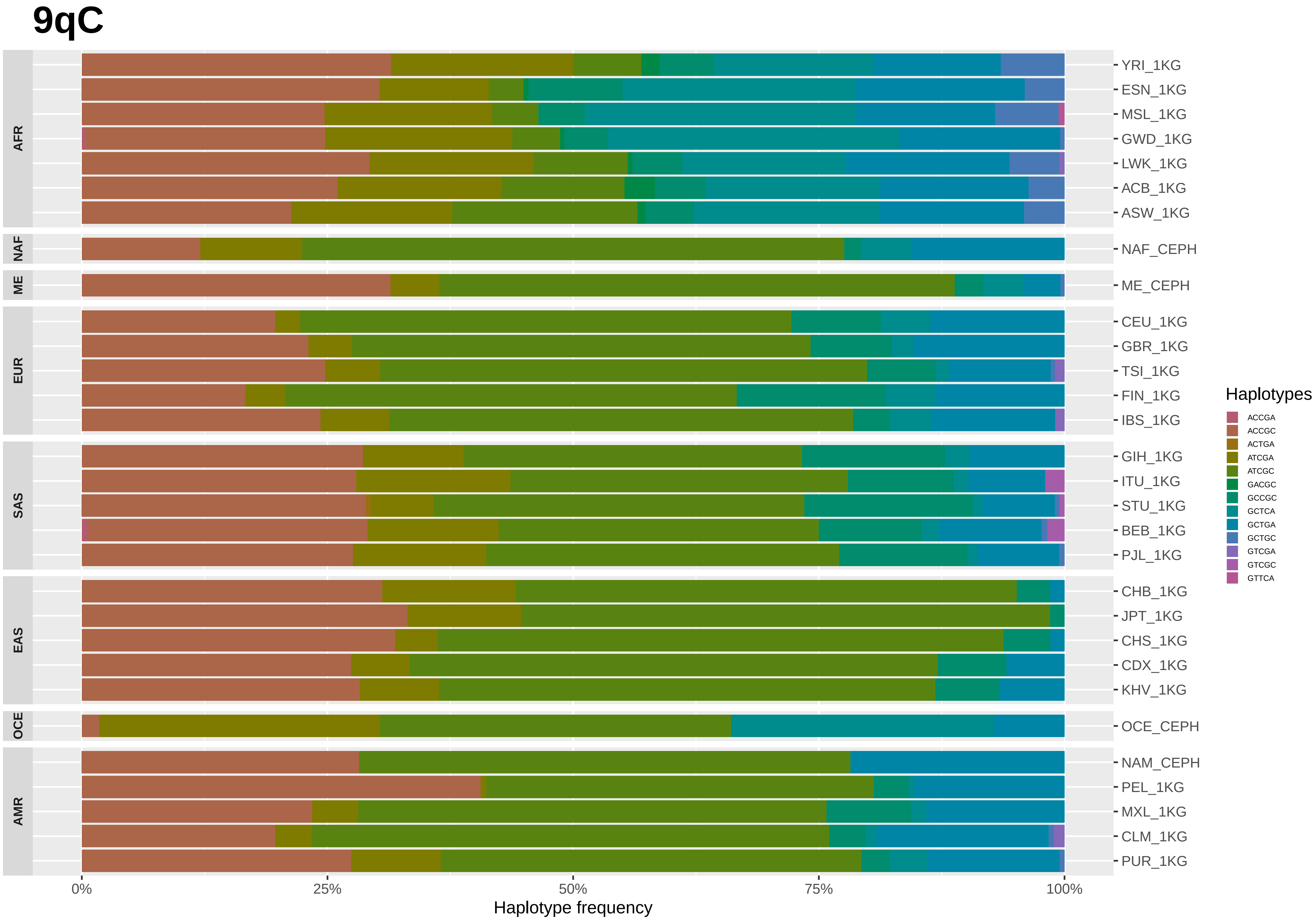

9qC
AFR
NAF
ME
EUR
SAS
EAS
OCE
AMR
YRI_1KG
ESN_1KG
MSL_1KG
GWD_1KG
LWK_1KG
ACB_1KG
ASW_1KG
NAF_CEPH
ME_CEPH
CEU_1KG
GBR_1KG
TSI_1KG
FIN_1KG
IBS_1KG
GIH_1KG
ITU_1KG
STU_1KG
BEB_1KG
PJL_1KG
CHB_1KG
JPT_1KG
CHS_1KG
CDX_1KG
KHV_1KG
OCE_CEPH
NAM_CEPH
PEL_1KG
MXL_1KG
CLM_1KG
PUR_1KG
0%
25%
50%
75%
100%
Haplotype frequency
Haplotypes
ACCGA
ACCGC
ACTGA
ATCGA
ATCGC
GACGC
GCCGC
GCTCA
GCTGA
GCTGC
GTCGA
GTCGC
GTTCA

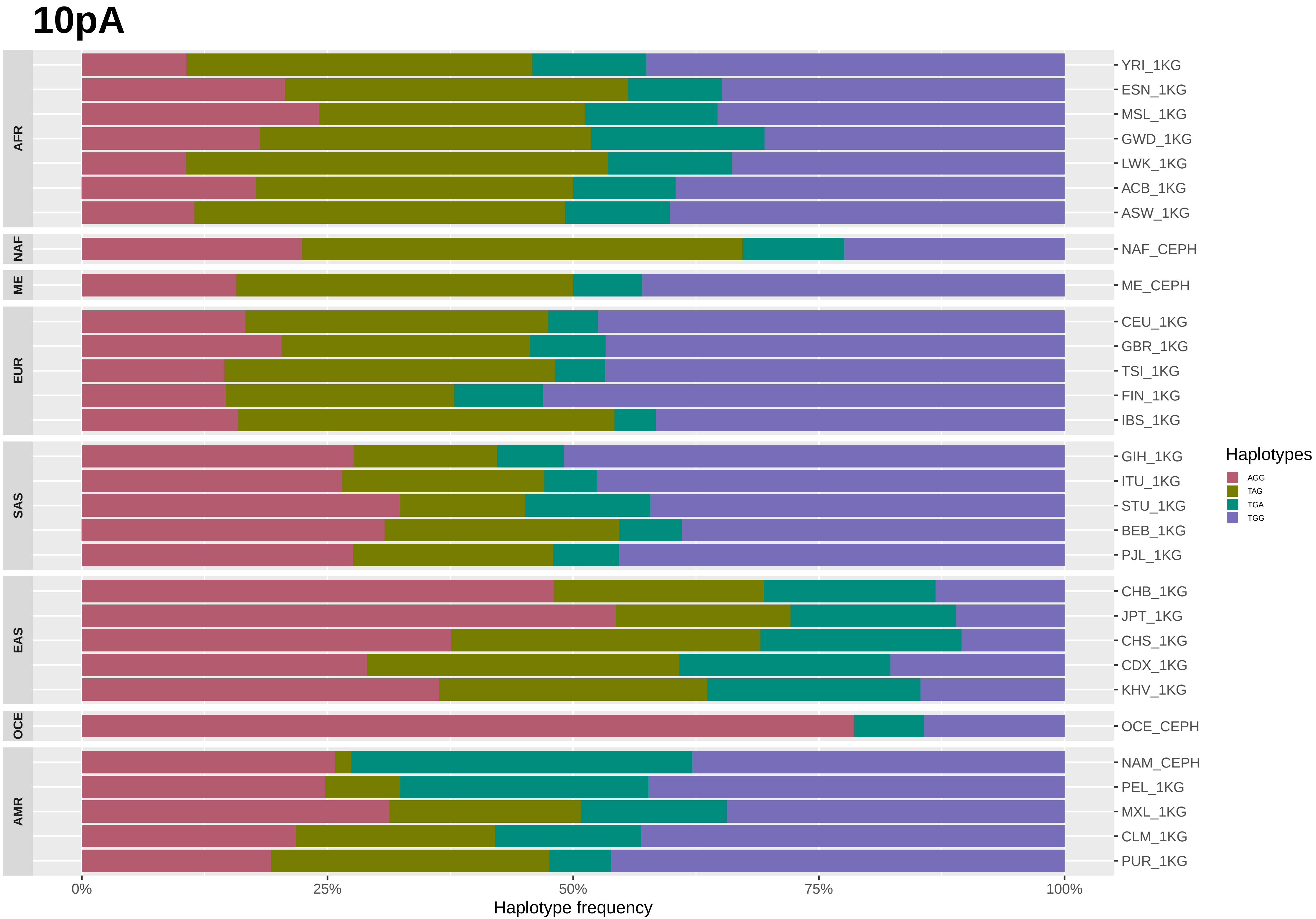
10pA
AFR
NAF
ME
EUR
SAS
EAS
OCE
AMR
YRI_1KG
ESN_1KG
MSL_1KG
GWD_1KG
LWK_1KG
ACB_1KG
ASW_1KG
NAF_CEPH
ME_CEPH
CEU_1KG
GBR_1KG
TSI_1KG
FIN_1KG
IBS_1KG
GIH_1KG
ITU_1KG
STU_1KG
BEB_1KG
PJL_1KG
CHB_1KG
JPT_1KG
CHS_1KG
CDX_1KG
KHV_1KG
OCE_CEPH
NAM_CEPH
PEL_1KG
MXL_1KG
CLM_1KG
PUR_1KG
0%
25%
50%
75%
100%
Haplotype frequency
Haplotypes
AGG
TAG
TGA
TGG

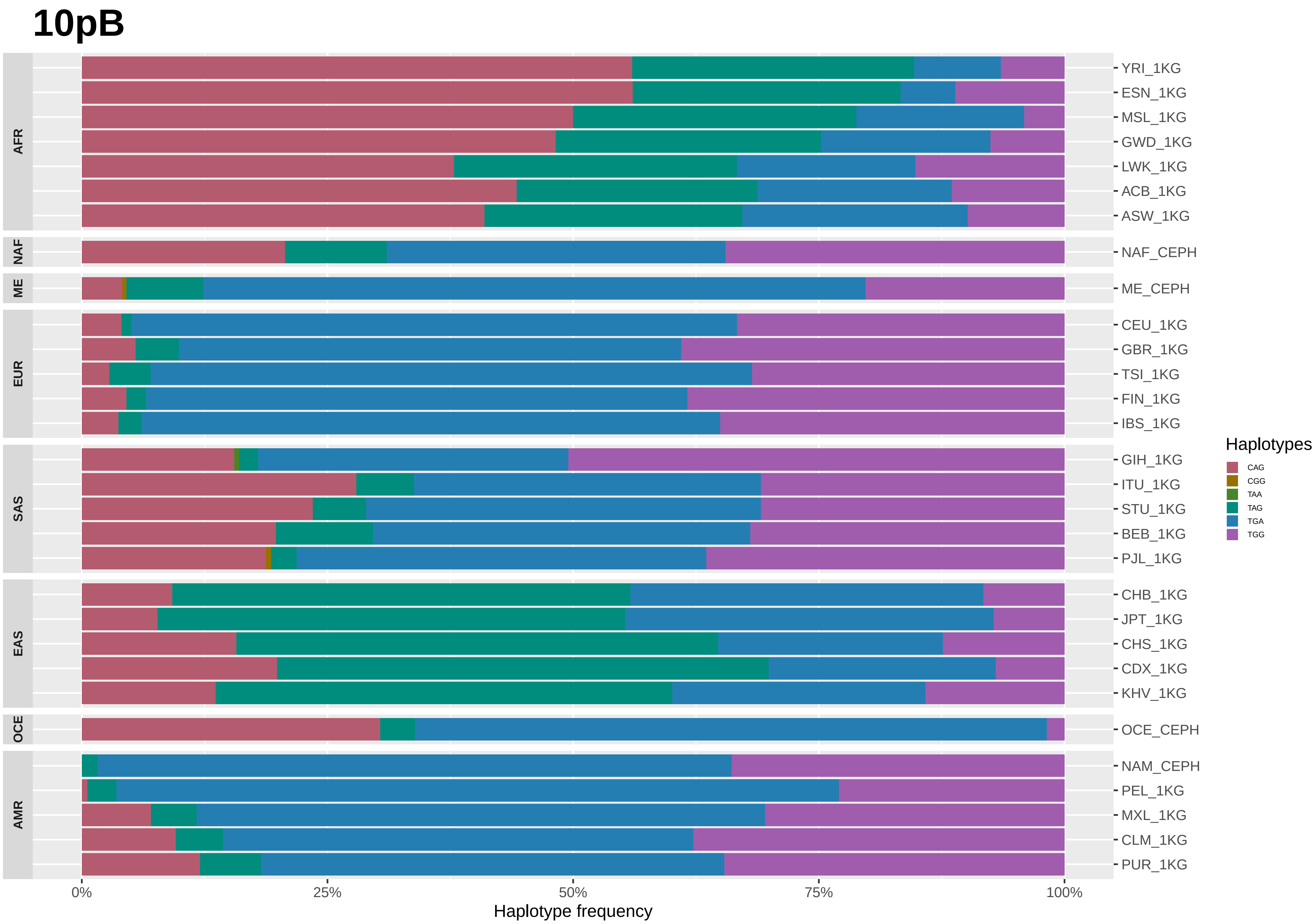

10pB
AFR
NAF
ME
EUR
SAS
EAS
OCE
AMR
YRI_1KG
ESN_1KG
MSL_1KG
GWD_1KG
LWK_1KG
ACB_1KG
ASW_1KG
NAF_CEPH
ME_CEPH
CEU_1KG
GBR_1KG
TSI_1KG
FIN_1KG
IBS_1KG
GIH_1KG
ITU_1KG
STU_1KG
BEB_1KG
PJL_1KG
CHB_1KG
JPT_1KG
CHS_1KG
CDX_1KG
KHV_1KG
OCE_CEPH
NAM_CEPH
PEL_1KG
MXL_1KG
CLM_1KG
PUR_1KG
0%
25%
50%
75%
100%
Haplotype frequency
Haplotypes
CAG
CGG
TAA
TAG
TGA
TGG

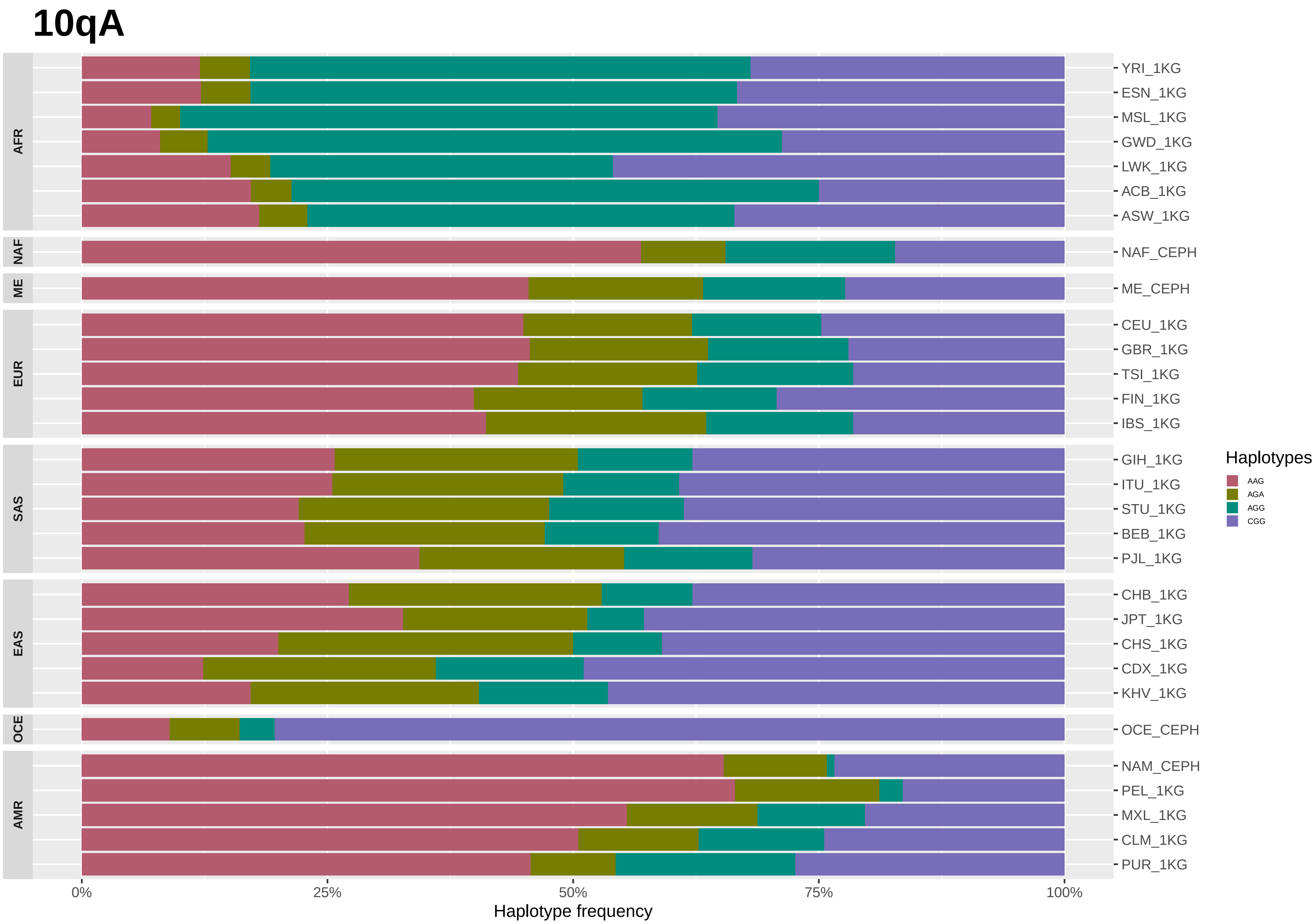
10qA
AFR
NAF
ME
EUR
SAS
EAS
OCE
AMR
YRI_1KG
ESN_1KG
MSL_1KG
GWD_1KG
LWK_1KG
ACB_1KG
ASW_1KG
NAF_CEPH
ME_CEPH
CEU_1KG
GBR_1KG
TSI_1KG
FIN_1KG
IBS_1KG
GIH_1KG
ITU_1KG
STU_1KG
BEB_1KG
PJL_1KG
CHB_1KG
JPT_1KG
CHS_1KG
CDX_1KG
KHV_1KG
OCE_CEPH
NAM_CEPH
PEL_1KG
MXL_1KG
CLM_1KG
PUR_1KG
0%
25%
50%
75%
100%
Haplotype frequency
Haplotypes
AAG
AGA
AGG
CGG

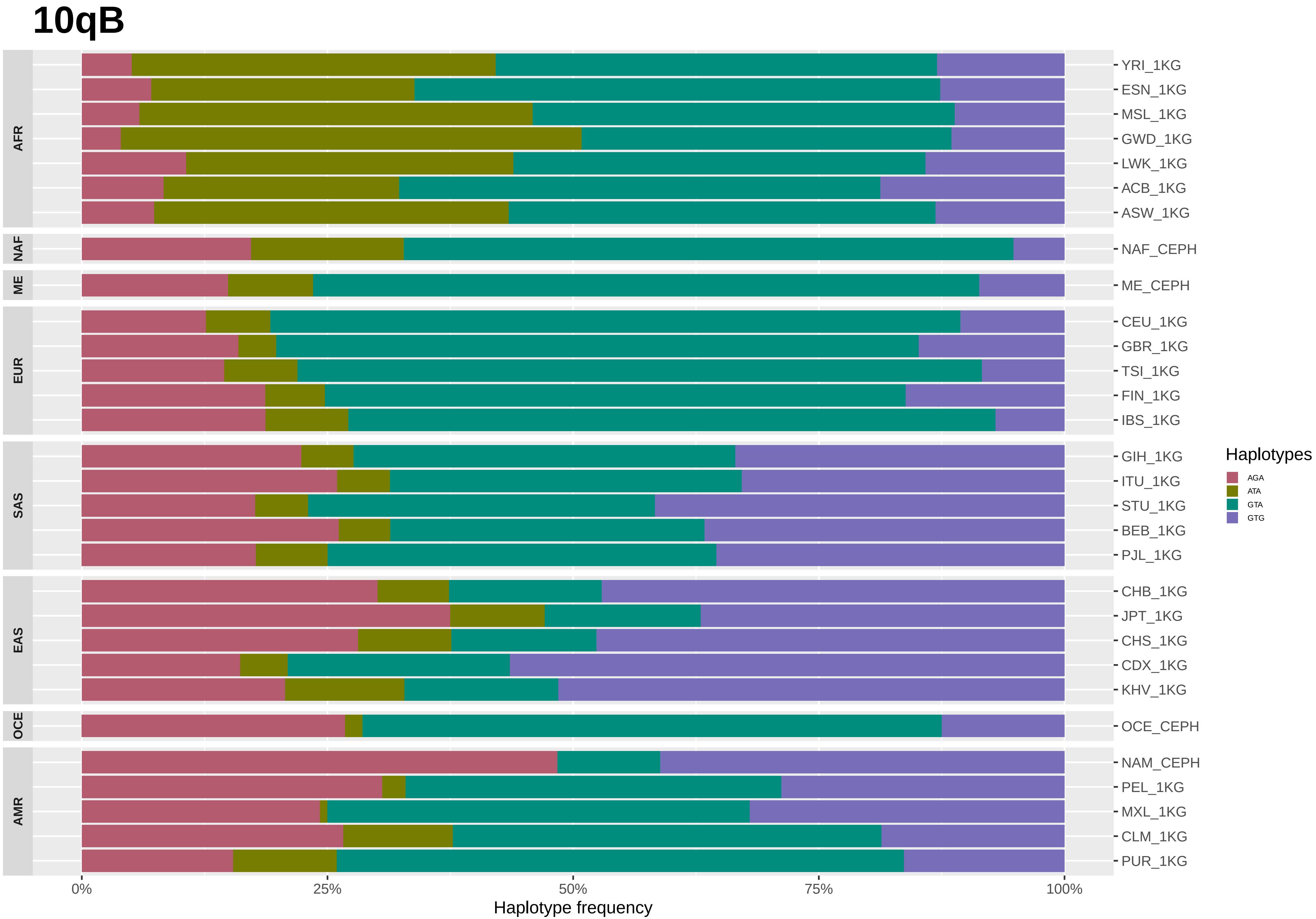
10qB
AFR
NAF
ME
EUR
SAS
EAS
OCE
AMR
YRI_1KG
ESN_1KG
MSL_1KG
GWD_1KG
LWK_1KG
ACB_1KG
ASW_1KG
NAF_CEPH
ME_CEPH
CEU_1KG
GBR_1KG
TSI_1KG
FIN_1KG
IBS_1KG
GIH_1KG
ITU_1KG
STU_1KG
BEB_1KG
PJL_1KG
CHB_1KG
JPT_1KG
CHS_1KG
CDX_1KG
KHV_1KG
OCE_CEPH
NAM_CEPH
PEL_1KG
MXL_1KG
CLM_1KG
PUR_1KG
0%
25%
50%
75%
100%
Haplotype frequency
Haplotypes
AGA
ATA
GTA
GTG

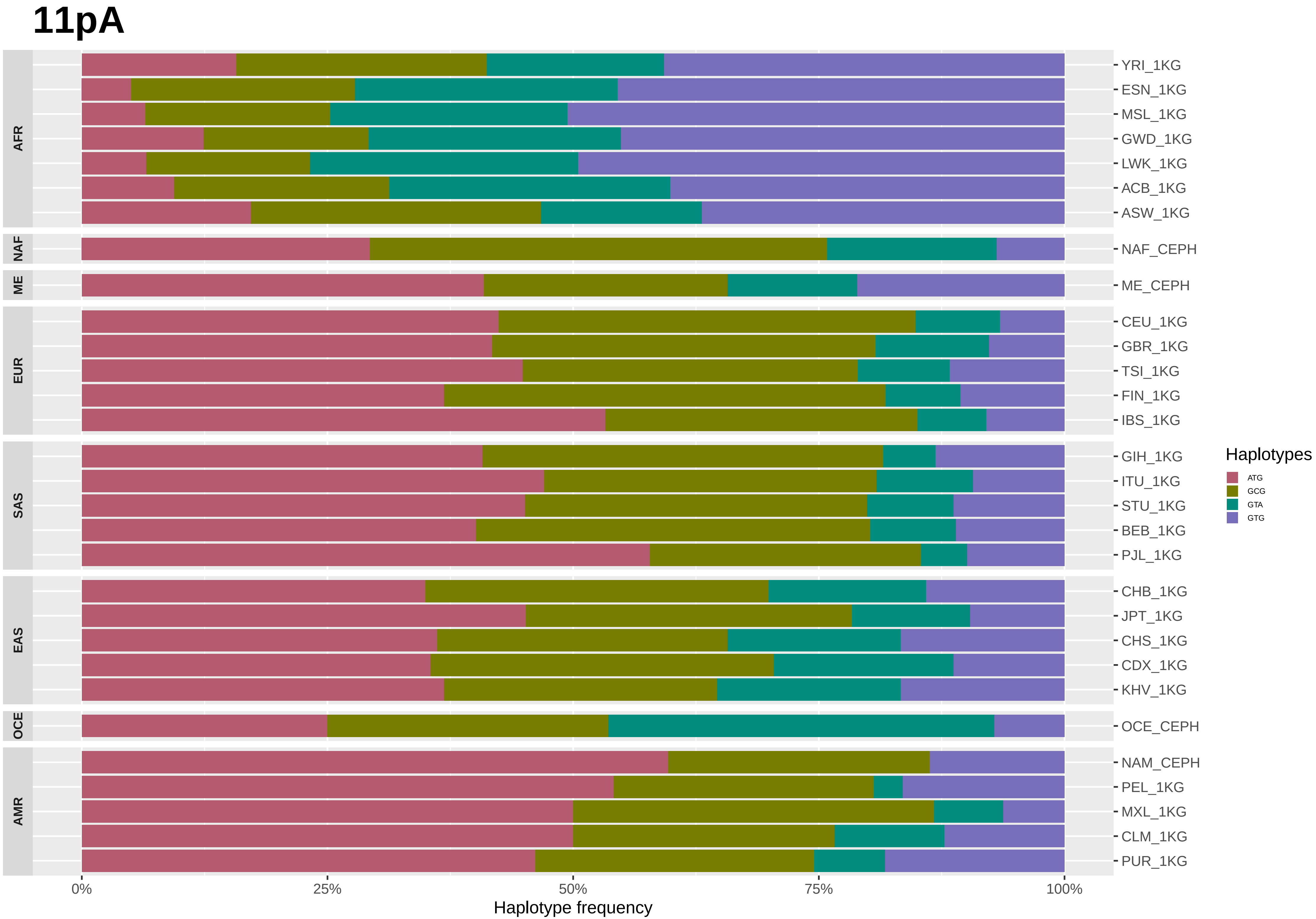

11pA
AFR
NAF
ME
EUR
SAS
EAS
OCE
AMR
YRI_1KG
ESN_1KG
MSL_1KG
GWD_1KG
LWK_1KG
ACB_1KG
ASW_1KG
NAF_CEPH
ME_CEPH
CEU_1KG
GBR_1KG
TSI_1KG
FIN_1KG
IBS_1KG
GIH_1KG
ITU_1KG
STU_1KG
BEB_1KG
PJL_1KG
CHB_1KG
JPT_1KG
CHS_1KG
CDX_1KG
KHV_1KG
OCE_CEPH
NAM_CEPH
PEL_1KG
MXL_1KG
CLM_1KG
PUR_1KG
0%
25%
50%
75%
100%
Haplotype frequency
Haplotypes
ATG
GCG
GTA
GTG

# 11pB

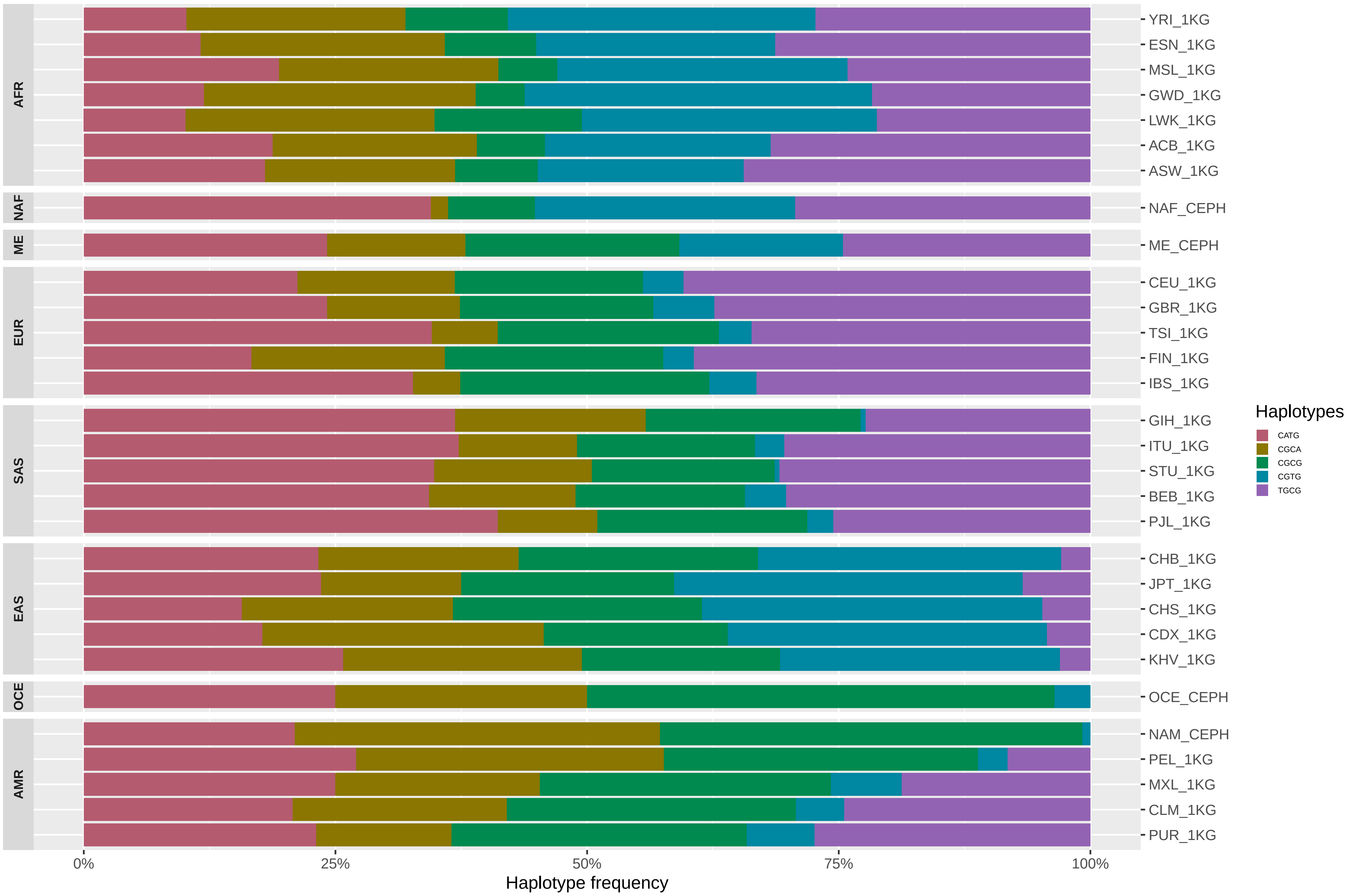
AFR
NAF
ME
EUR
SAS
EAS
OCE
AMR
YRI_1KG
ESN_1KG
MSL_1KG
GWD_1KG
LWK_1KG
ACB_1KG
ASW_1KG
NAF_CEPH
ME_CEPH
CEU_1KG
GBR_1KG
TSI_1KG
FIN_1KG
IBS_1KG
GIH_1KG
ITU_1KG
STU_1KG
BEB_1KG
PJL_1KG
CHB_1KG
JPT_1KG
CHS_1KG
CDX_1KG
KHV_1KG
OCE_CEPH
NAM_CEPH
PEL_1KG
MXL_1KG
CLM_1KG
PUR_1KG
0%
25%
50%
75%
100%
Haplotype frequency
Haplotypes
CATG
CGCA
CGCG
CGTG
TGCG

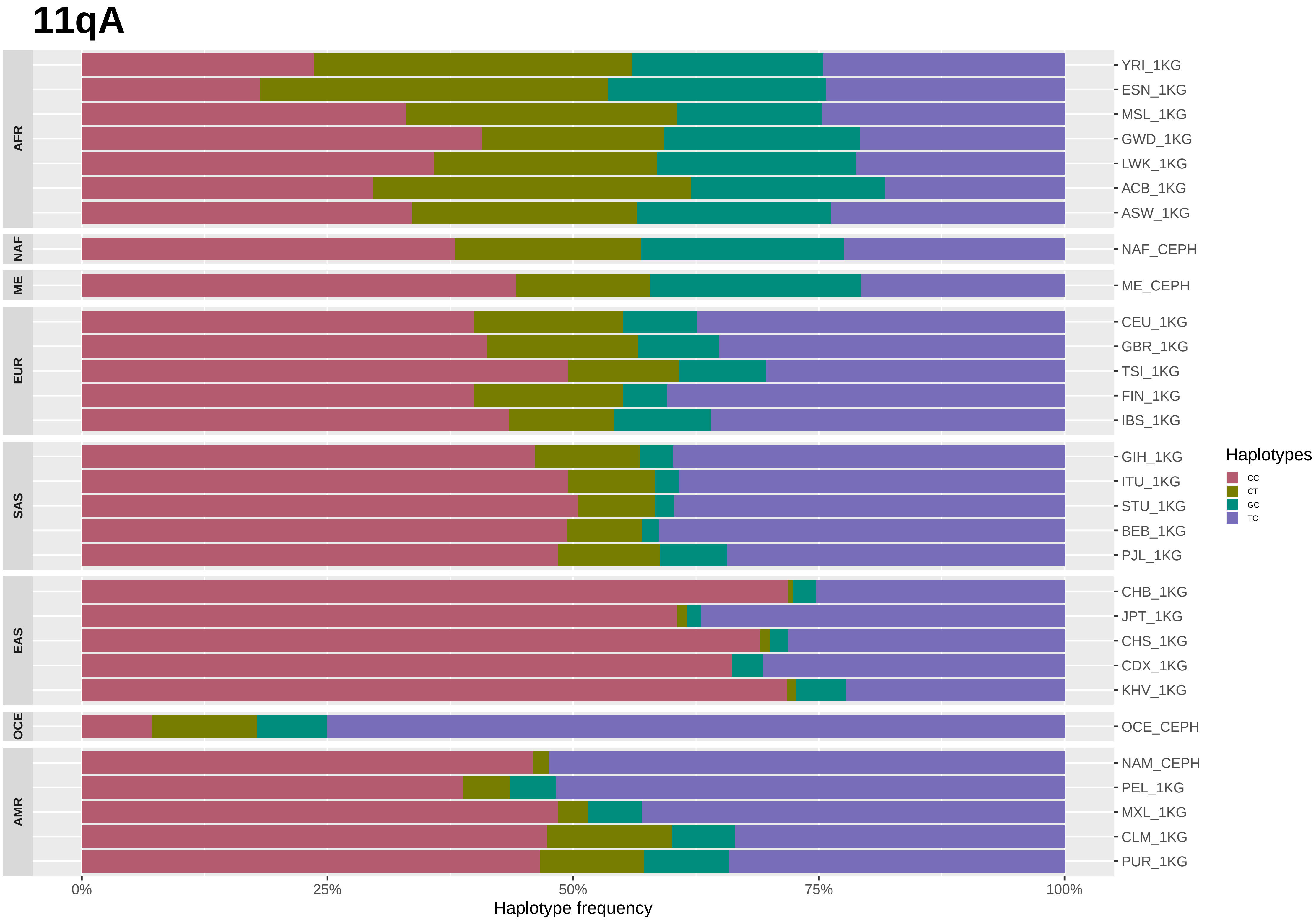
11qA
AFR
NAF
ME
EUR
SAS
EAS
OCE
AMR
YRI_1KG
ESN_1KG
MSL_1KG
GWD_1KG
LWK_1KG
ACB_1KG
ASW_1KG
NAF_CEPH
ME_CEPH
CEU_1KG
GBR_1KG
TSI_1KG
FIN_1KG
IBS_1KG
GIH_1KG
ITU_1KG
STU_1KG
BEB_1KG
PJL_1KG
CHB_1KG
JPT_1KG
CHS_1KG
CDX_1KG
KHV_1KG
OCE_CEPH
NAM_CEPH
PEL_1KG
MXL_1KG
CLM_1KG
PUR_1KG
0%
25%
50%
75%
100%
Haplotype frequency
Haplotypes
CC
CT
GC
TC

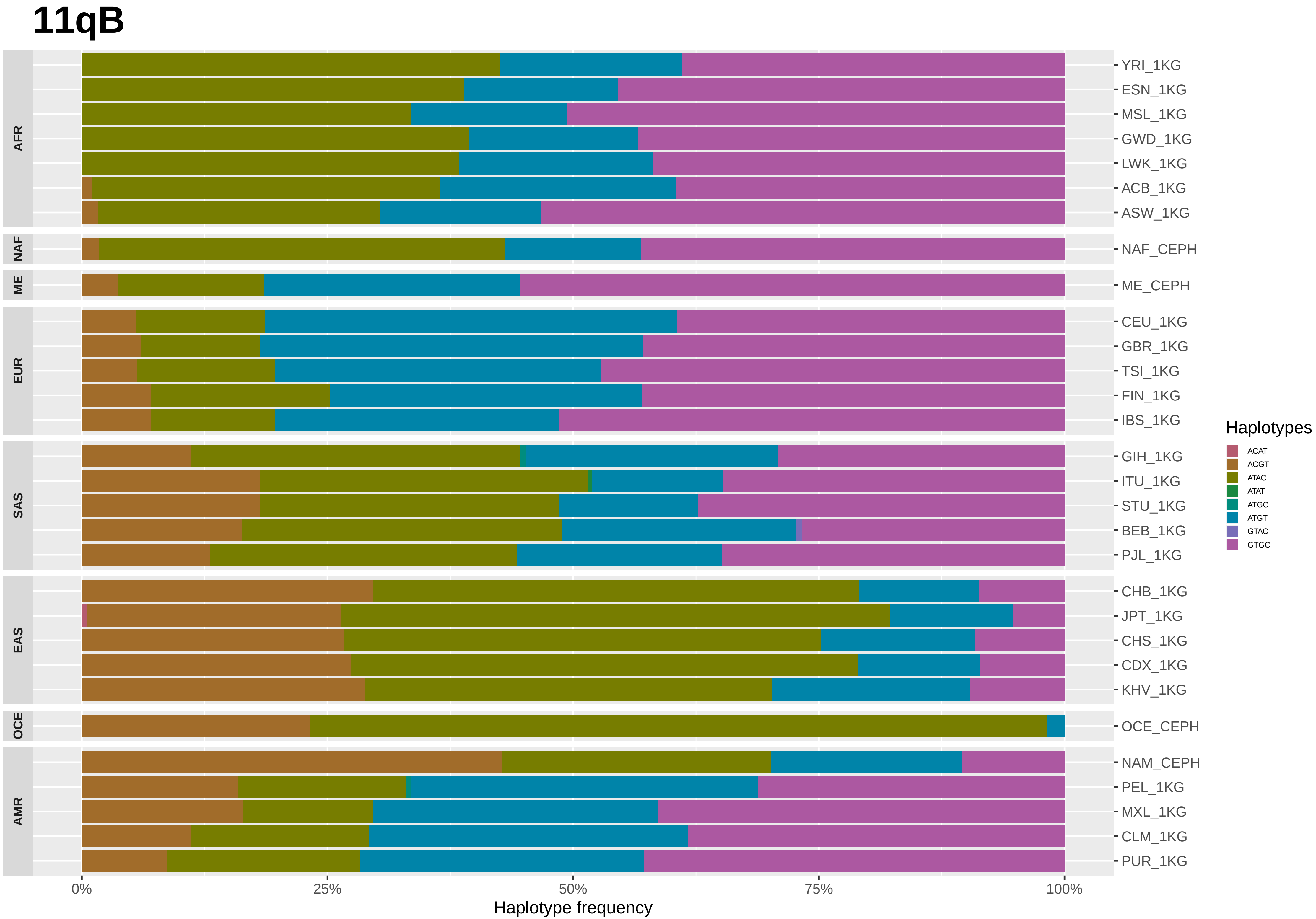
11qB
AFR
NAF
ME
EUR
SAS
EAS
OCE
AMR
YRI_1KG
ESN_1KG
MSL_1KG
GWD_1KG
LWK_1KG
ACB_1KG
ASW_1KG
NAF_CEPH
ME_CEPH
CEU_1KG
GBR_1KG
TSI_1KG
FIN_1KG
IBS_1KG
GIH_1KG
ITU_1KG
STU_1KG
BEB_1KG
PJL_1KG
CHB_1KG
JPT_1KG
CHS_1KG
CDX_1KG
KHV_1KG
OCE_CEPH
NAM_CEPH
PEL_1KG
MXL_1KG
CLM_1KG
PUR_1KG
0%
25%
50%
75%
100%
Haplotype frequency
Haplotypes
ACAT
ACGT
ATAC
ATAT
ATGC
ATGT
GTAC
GTGC

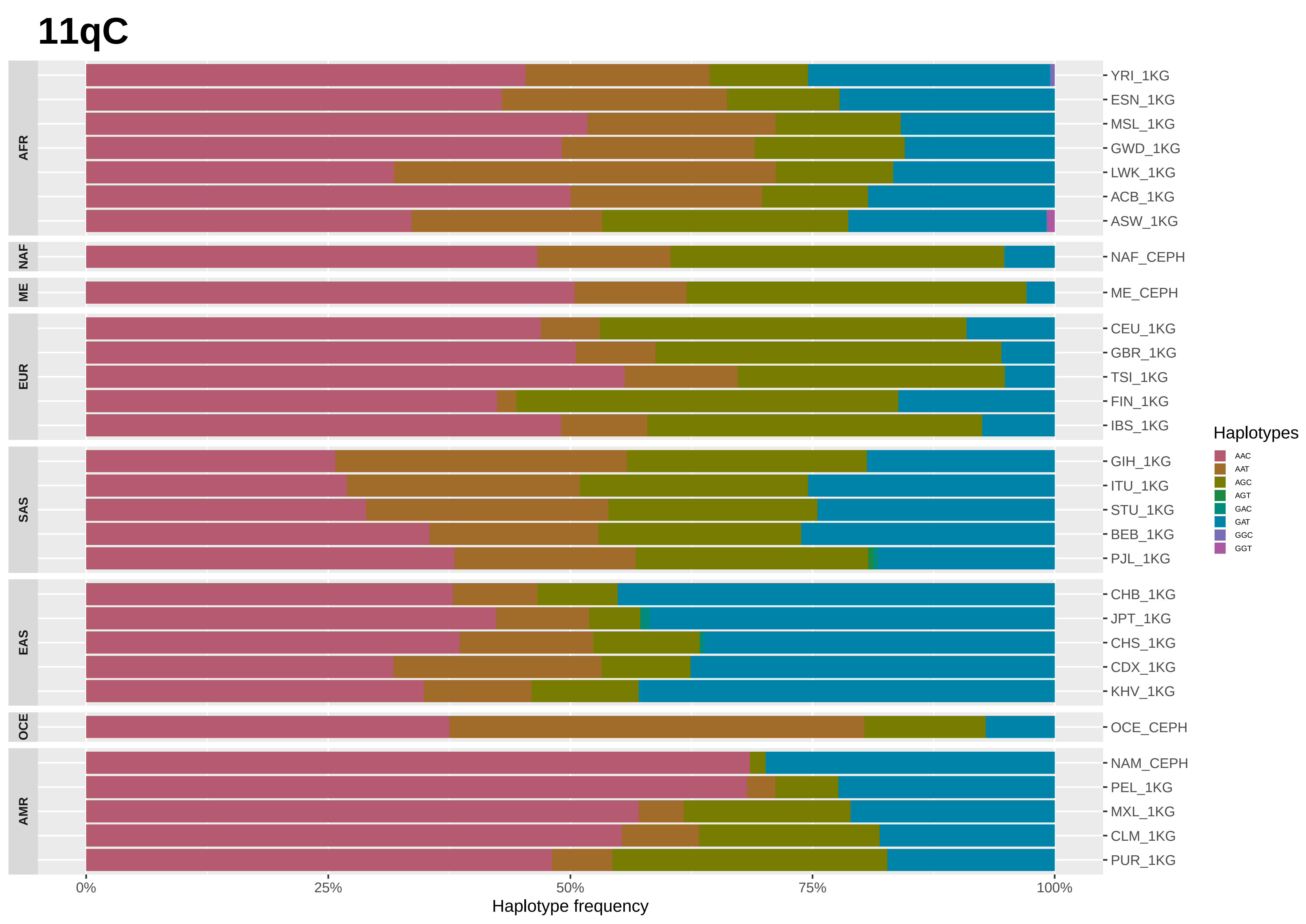

11qC
AFR
NAF
ME
EUR
SAS
EAS
OCE
AMR
YRI_1KG
ESN_1KG
MSL_1KG
GWD_1KG
LWK_1KG
ACB_1KG
ASW_1KG
NAF_CEPH
ME_CEPH
CEU_1KG
GBR_1KG
TSI_1KG
FIN_1KG
IBS_1KG
GIH_1KG
ITU_1KG
STU_1KG
BEB_1KG
PJL_1KG
CHB_1KG
JPT_1KG
CHS_1KG
CDX_1KG
KHV_1KG
OCE_CEPH
NAM_CEPH
PEL_1KG
MXL_1KG
CLM_1KG
PUR_1KG
0%
25%
50%
75%
100%
Haplotype frequency
Haplotypes
AAC
AAT
AGC
AGT
GAC
GAT
GGC
GGT

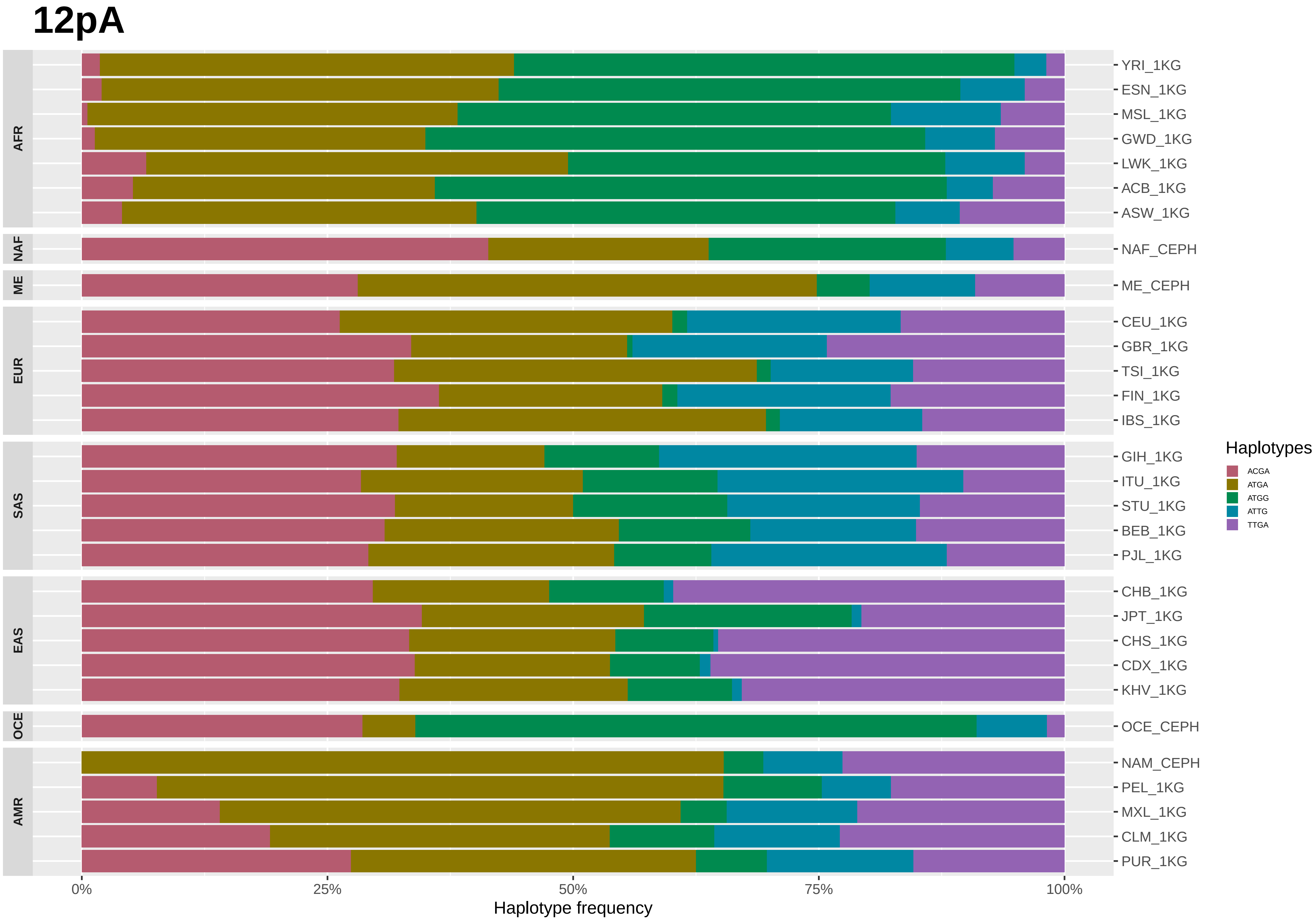

12pA
AFR
NAF
ME
EUR
SAS
EAS
OCE
AMR
YRI_1KG
ESN_1KG
MSL_1KG
GWD_1KG
LWK_1KG
ACB_1KG
ASW_1KG
NAF_CEPH
ME_CEPH
CEU_1KG
GBR_1KG
TSI_1KG
FIN_1KG
IBS_1KG
GIH_1KG
ITU_1KG
STU_1KG
BEB_1KG
PJL_1KG
CHB_1KG
JPT_1KG
CHS_1KG
CDX_1KG
KHV_1KG
OCE_CEPH
NAM_CEPH
PEL_1KG
MXL_1KG
CLM_1KG
PUR_1KG
0%
25%
50%
75%
100%
Haplotype frequency
Haplotypes
ACGA
ATGA
ATGG
ATTG
TTGA

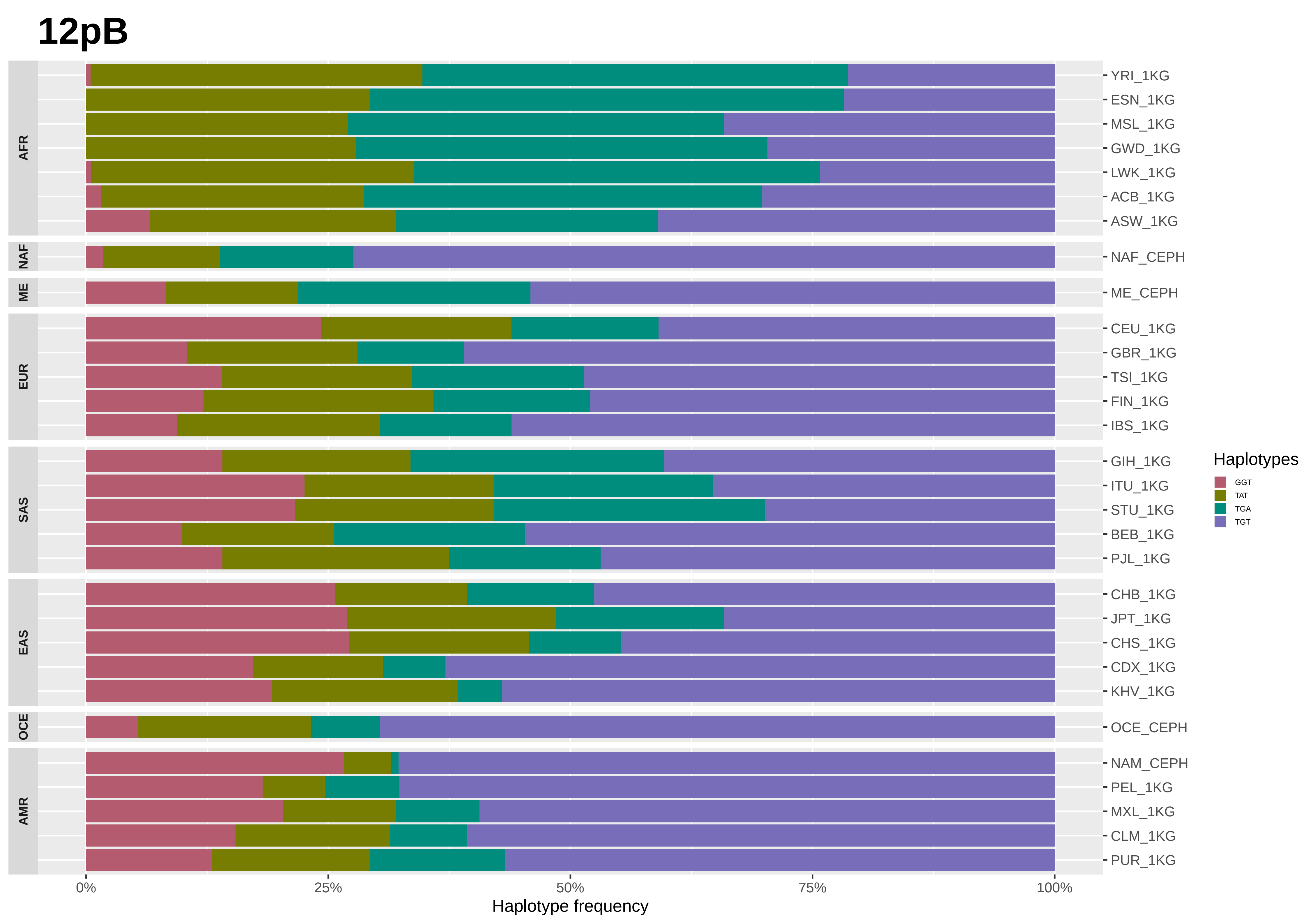

12pB
AFR
NAF
ME
EUR
SAS
EAS
OCE
AMR
YRI_1KG
ESN_1KG
MSL_1KG
GWD_1KG
LWK_1KG
ACB_1KG
ASW_1KG
NAF_CEPH
ME_CEPH
CEU_1KG
GBR_1KG
TSI_1KG
FIN_1KG
IBS_1KG
GIH_1KG
ITU_1KG
STU_1KG
BEB_1KG
PJL_1KG
CHB_1KG
JPT_1KG
CHS_1KG
CDX_1KG
KHV_1KG
OCE_CEPH
NAM_CEPH
PEL_1KG
MXL_1KG
CLM_1KG
PUR_1KG
0%
25%
50%
75%
100%
Haplotype frequency
Haplotypes
GGT
TAT
TGA
TGT

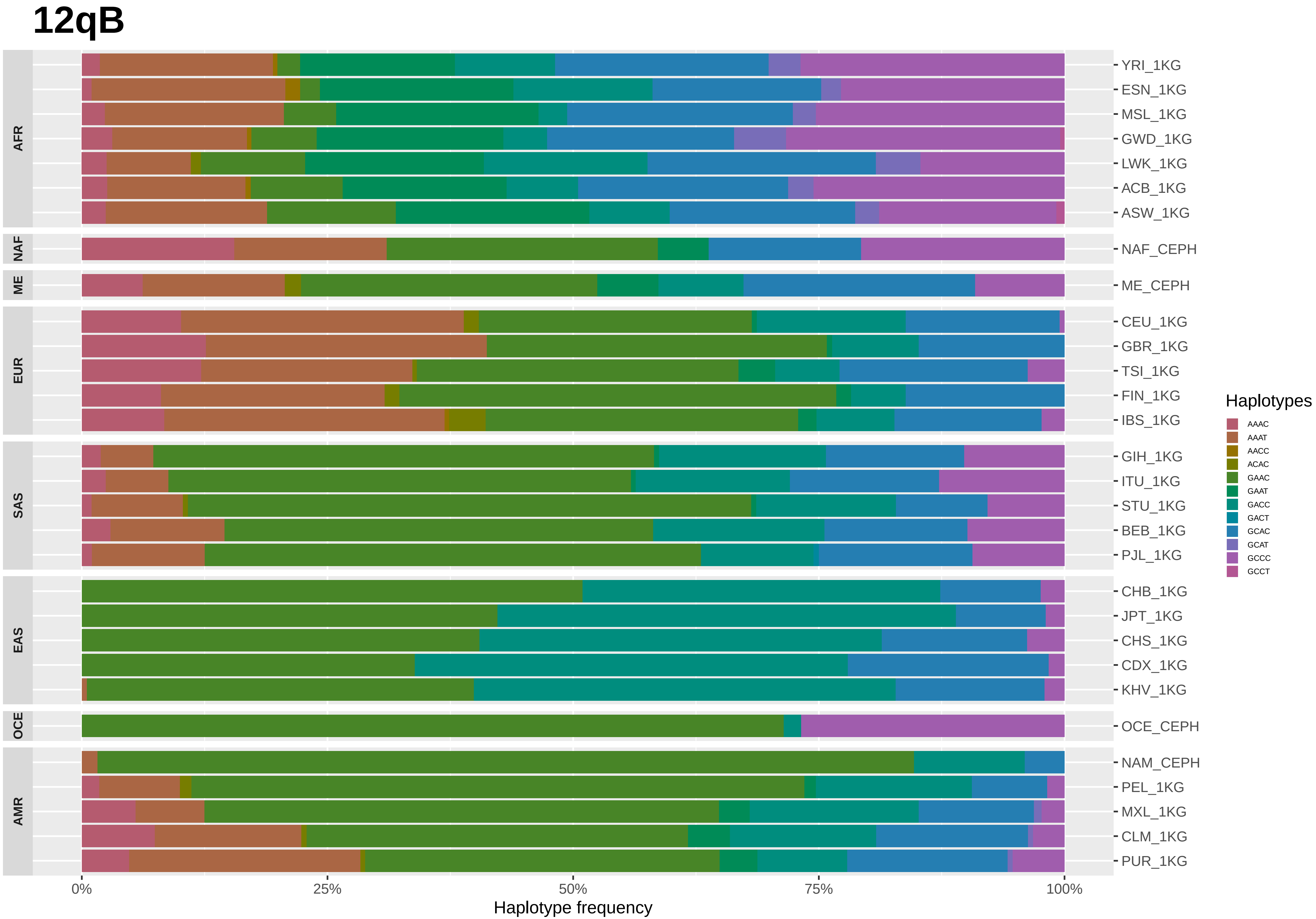
12qB
AFR
NAF
ME
EUR
SAS
EAS
OCE
AMR
YRI_1KG
ESN_1KG
MSL_1KG
GWD_1KG
LWK_1KG
ACB_1KG
ASW_1KG
NAF_CEPH
ME_CEPH
CEU_1KG
GBR_1KG
TSI_1KG
FIN_1KG
IBS_1KG
GIH_1KG
ITU_1KG
STU_1KG
BEB_1KG
PJL_1KG
CHB_1KG
JPT_1KG
CHS_1KG
CDX_1KG
KHV_1KG
OCE_CEPH
NAM_CEPH
PEL_1KG
MXL_1KG
CLM_1KG
PUR_1KG
0%
25%
50%
75%
100%
Haplotype frequency
Haplotypes
AAAC
AAAT
AACC
ACAC
GAAC
GAAT
GACC
GACT
GCAC
GCAT
GCCC
GCCT

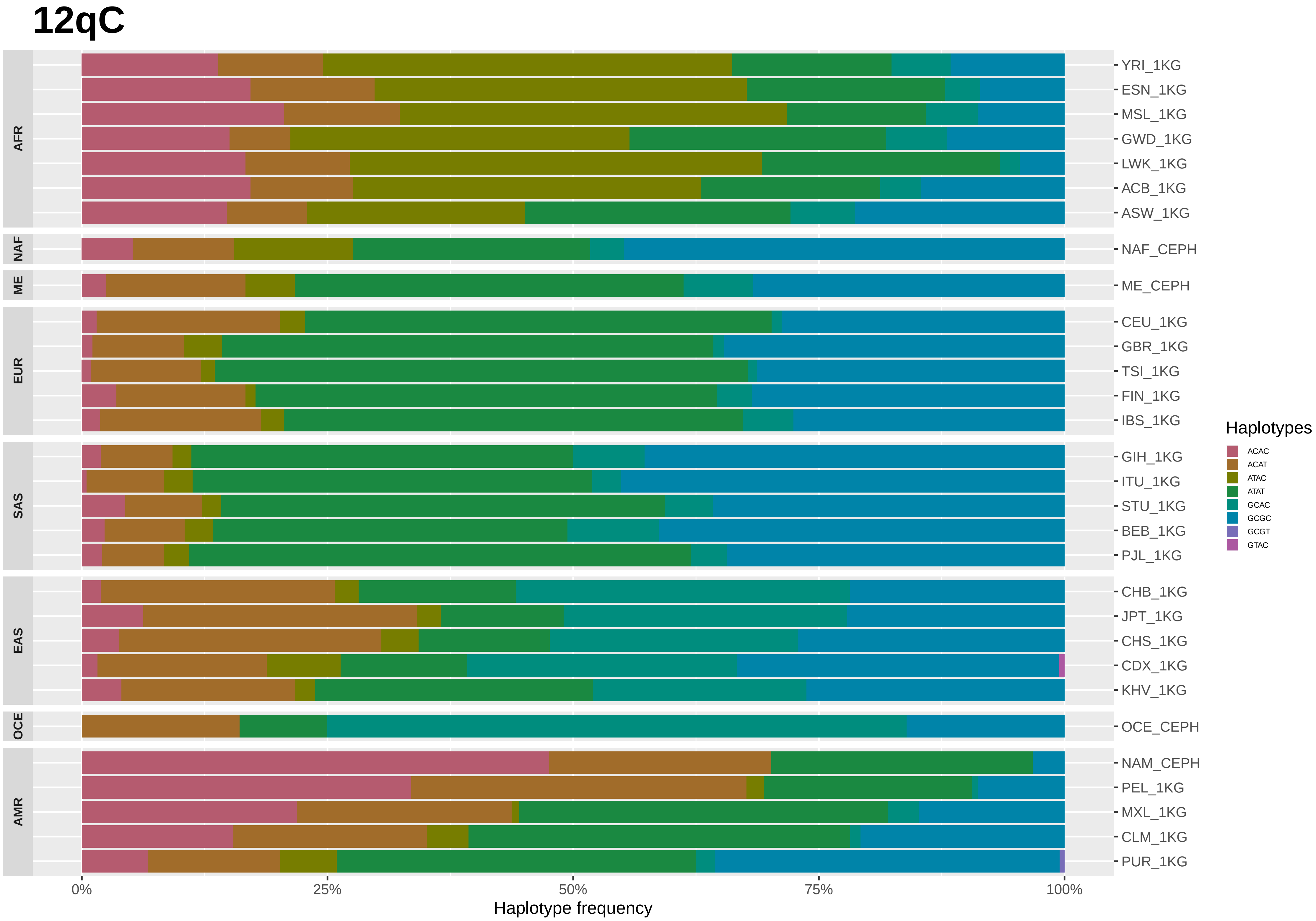
12qC
AFR
NAF
ME
EUR
SAS
EAS
OCE
AMR
YRI_1KG
ESN_1KG
MSL_1KG
GWD_1KG
LWK_1KG
ACB_1KG
ASW_1KG
NAF_CEPH
ME_CEPH
CEU_1KG
GBR_1KG
TSI_1KG
FIN_1KG
IBS_1KG
GIH_1KG
ITU_1KG
STU_1KG
BEB_1KG
PJL_1KG
CHB_1KG
JPT_1KG
CHS_1KG
CDX_1KG
KHV_1KG
OCE_CEPH
NAM_CEPH
PEL_1KG
MXL_1KG
CLM_1KG
PUR_1KG
0%
25%
50%
75%
100%
Haplotype frequency
Haplotypes
ACAC
ACAT
ATAC
ATAT
GCAC
GCGC
GCGT
GTAC

## 13qA

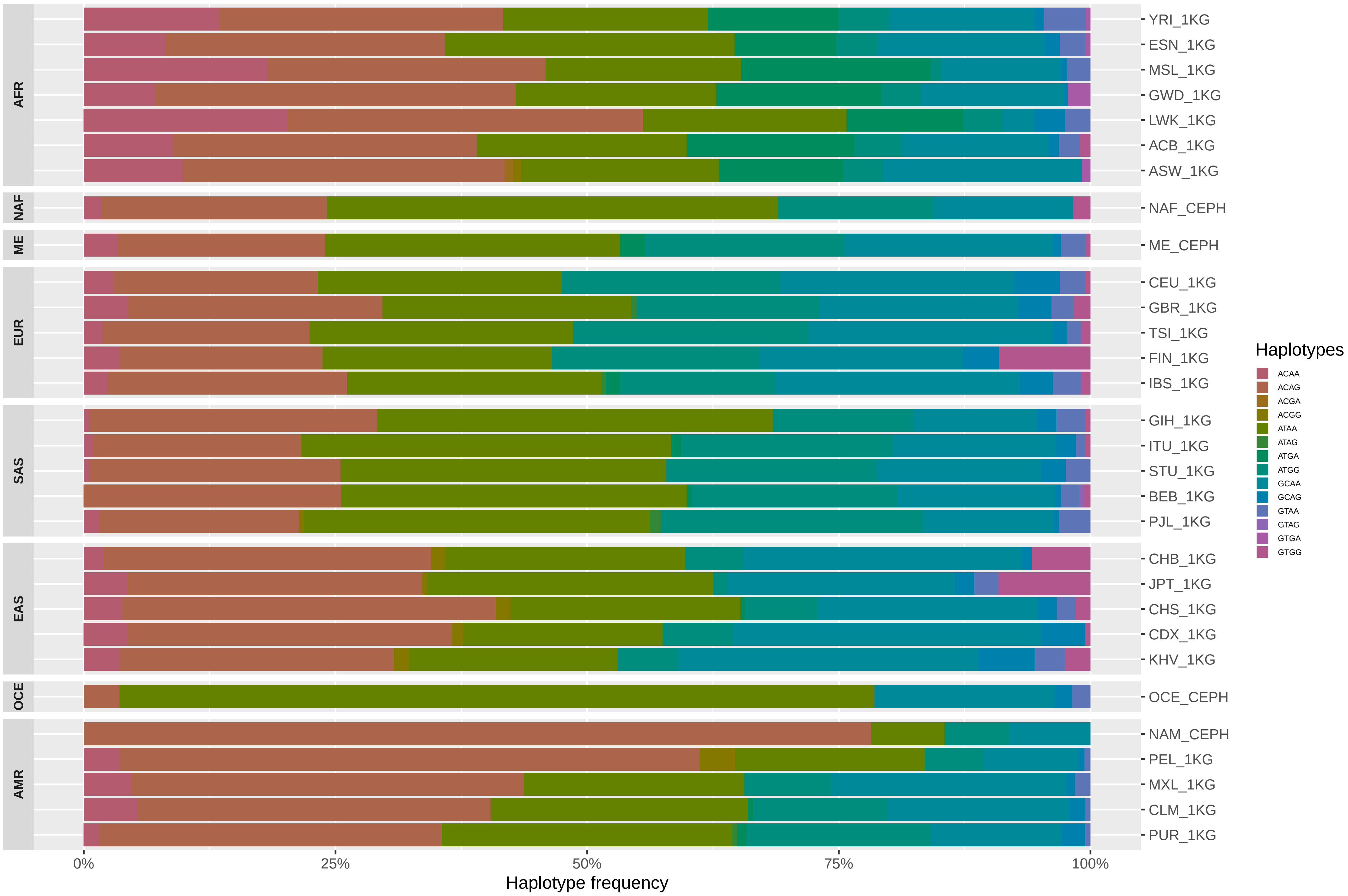

AFR
NAF
ME
EUR
SAS
EAS
OCE
AMR
YRI_1KG
ESN_1KG
MSL_1KG
GWD_1KG
LWK_1KG
ACB_1KG
ASW_1KG
NAF_CEPH
ME_CEPH
CEU_1KG
GBR_1KG
TSI_1KG
FIN_1KG
IBS_1KG
GIH_1KG
ITU_1KG
STU_1KG
BEB_1KG
PJL_1KG
CHB_1KG
JPT_1KG
CHS_1KG
CDX_1KG
KHV_1KG
OCE_CEPH
NAM_CEPH
PEL_1KG
MXL_1KG
CLM_1KG
PUR_1KG
0%
25%
50%
75%
100%
Haplotype frequency
Haplotypes
ACAA
ACAG
ACGA
ACGG
ATAA
ATAG
ATGA
ATGG
GCAA
GCAG
GTAA
GTAG
GTGA
GTGG

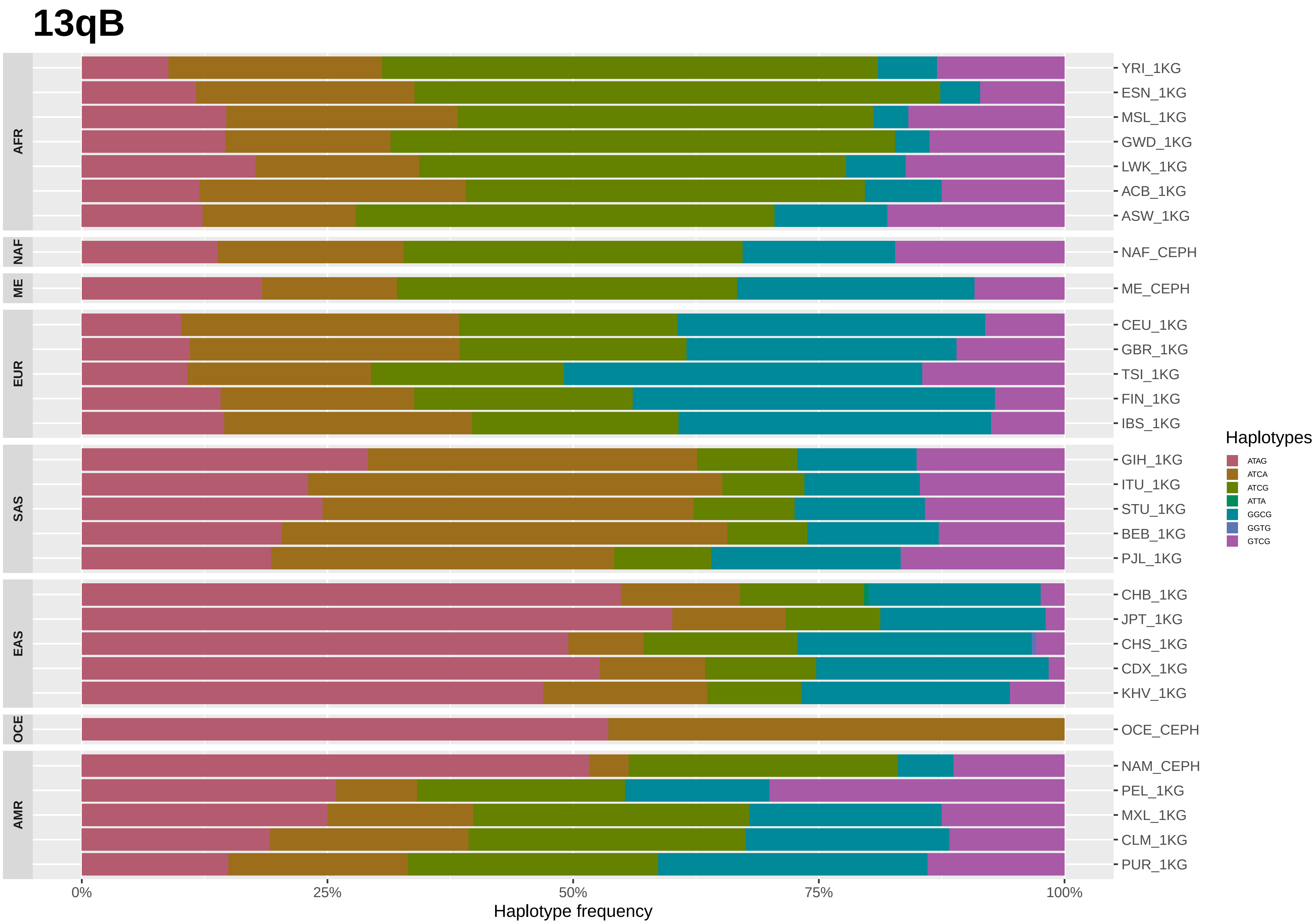

13qB
AFR
NAF
ME
EUR
SAS
EAS
OCE
AMR
YRI_1KG
ESN_1KG
MSL_1KG
GWD_1KG
LWK_1KG
ACB_1KG
ASW_1KG
NAF_CEPH
ME_CEPH
CEU_1KG
GBR_1KG
TSI_1KG
FIN_1KG
IBS_1KG
GIH_1KG
ITU_1KG
STU_1KG
BEB_1KG
PJL_1KG
CHB_1KG
JPT_1KG
CHS_1KG
CDX_1KG
KHV_1KG
OCE_CEPH
NAM_CEPH
PEL_1KG
MXL_1KG
CLM_1KG
PUR_1KG
0%
25%
50%
75%
100%
Haplotype frequency
Haplotypes
ATAG
ATCA
ATCG
ATTA
GGCG
GGTG
GTCG

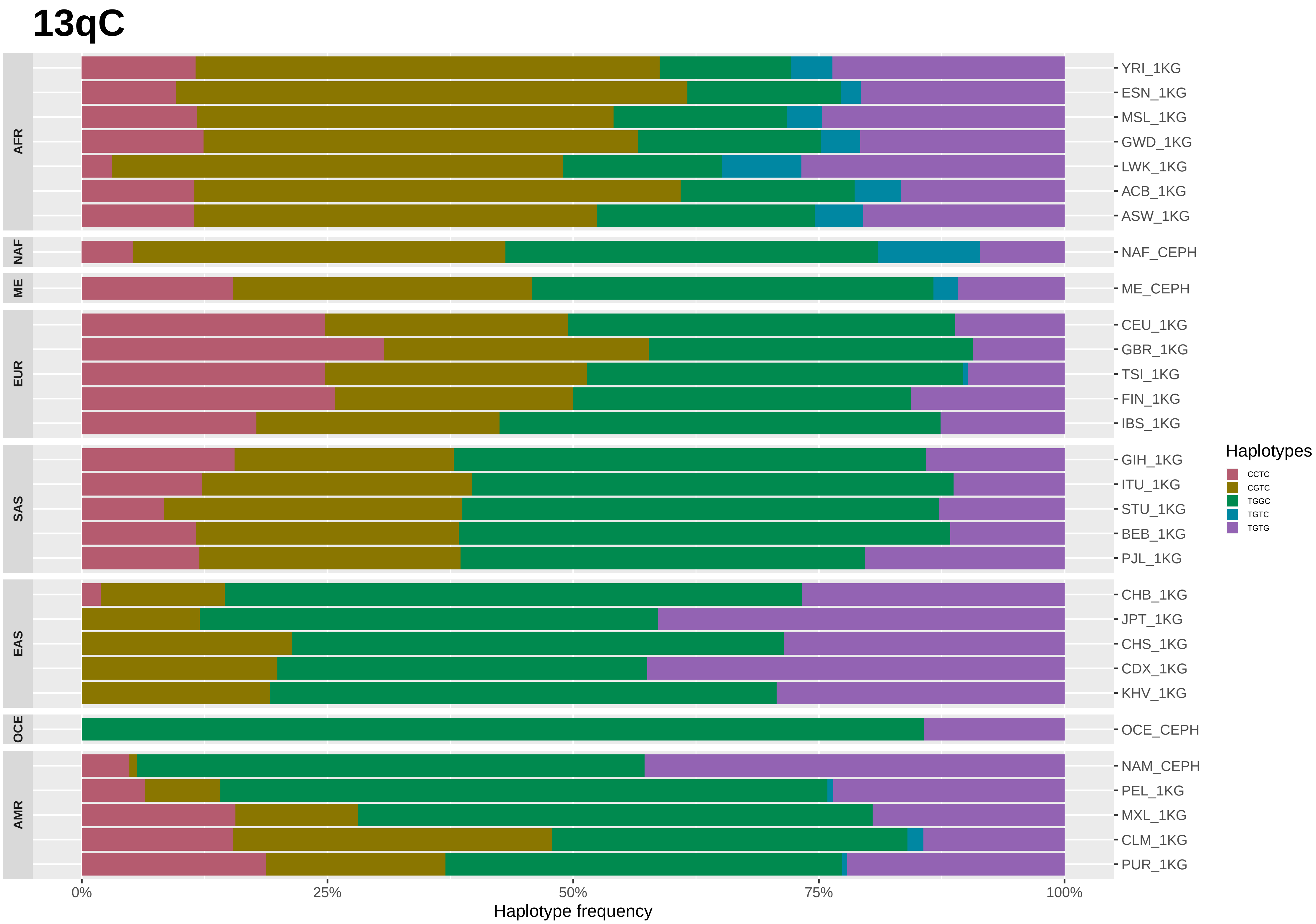
13qC
AFR
NAF
ME
EUR
SAS
EAS
OCE
AMR
YRI_1KG
ESN_1KG
MSL_1KG
GWD_1KG
LWK_1KG
ACB_1KG
ASW_1KG
NAF_CEPH
ME_CEPH
CEU_1KG
GBR_1KG
TSI_1KG
FIN_1KG
IBS_1KG
GIH_1KG
ITU_1KG
STU_1KG
BEB_1KG
PJL_1KG
CHB_1KG
JPT_1KG
CHS_1KG
CDX_1KG
KHV_1KG
OCE_CEPH
NAM_CEPH
PEL_1KG
MXL_1KG
CLM_1KG
PUR_1KG
0%
25%
50%
75%
100%
Haplotype frequency
Haplotypes
CCTC
CGTC
TGGC
TGTC
TGTG

# 13qD

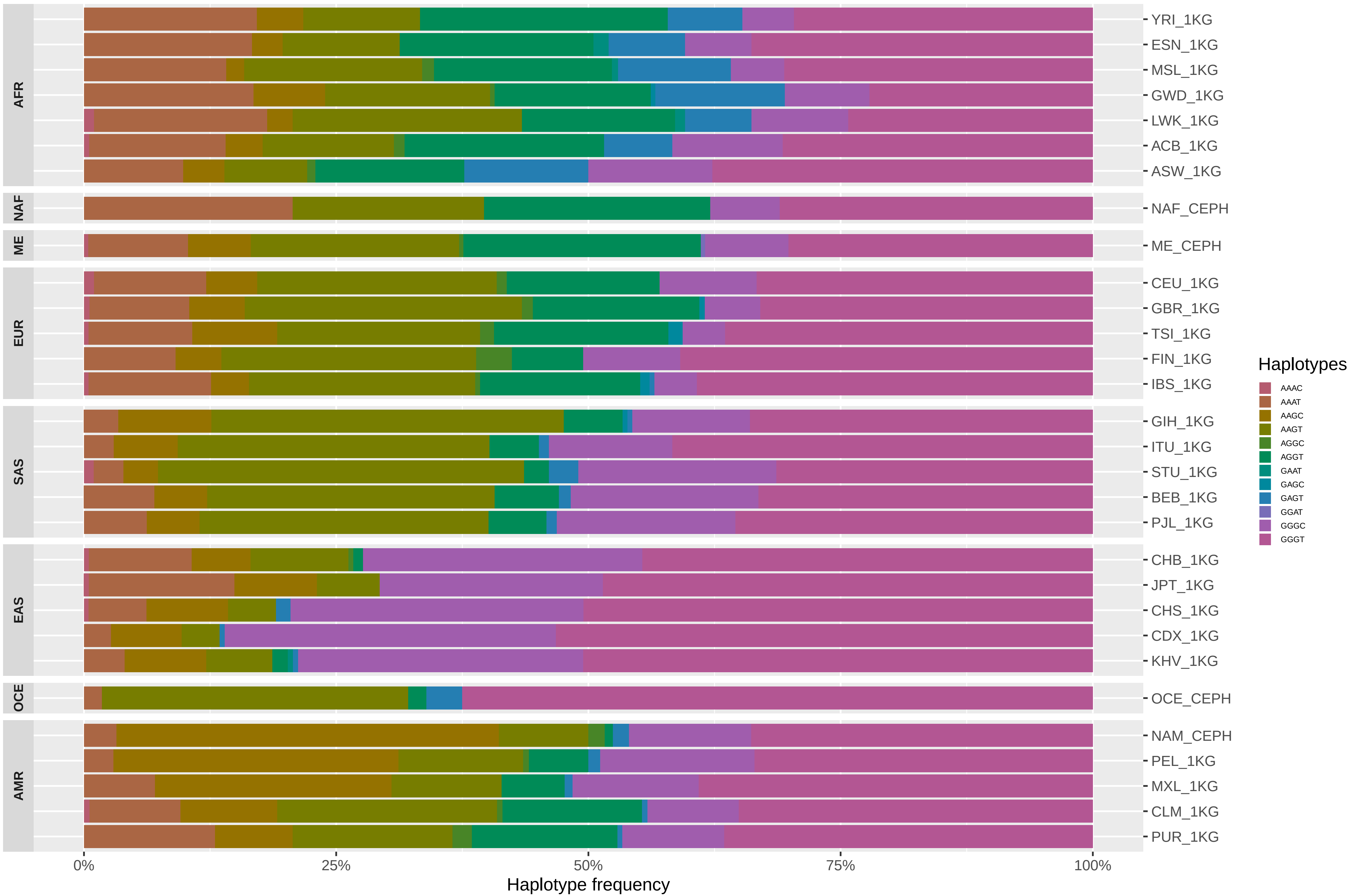

AFR
NAF
ME
EUR
SAS
EAS
OCE
AMR
YRI_1KG
ESN_1KG
MSL_1KG
GWD_1KG
LWK_1KG
ACB_1KG
ASW_1KG
NAF_CEPH
ME_CEPH
CEU_1KG
GBR_1KG
TSI_1KG
FIN_1KG
IBS_1KG
GIH_1KG
ITU_1KG
STU_1KG
BEB_1KG
PJL_1KG
CHB_1KG
JPT_1KG
CHS_1KG
CDX_1KG
KHV_1KG
OCE_CEPH
NAM_CEPH
PEL_1KG
MXL_1KG
CLM_1KG
PUR_1KG
0%
25%
50%
75%
100%
Haplotype frequency
Haplotypes
AAAC
AAAT
AAGC
AAGT
AGGC
AGGT
GAAT
GAGC
GAGT
GGAT
GGGC
GGGT

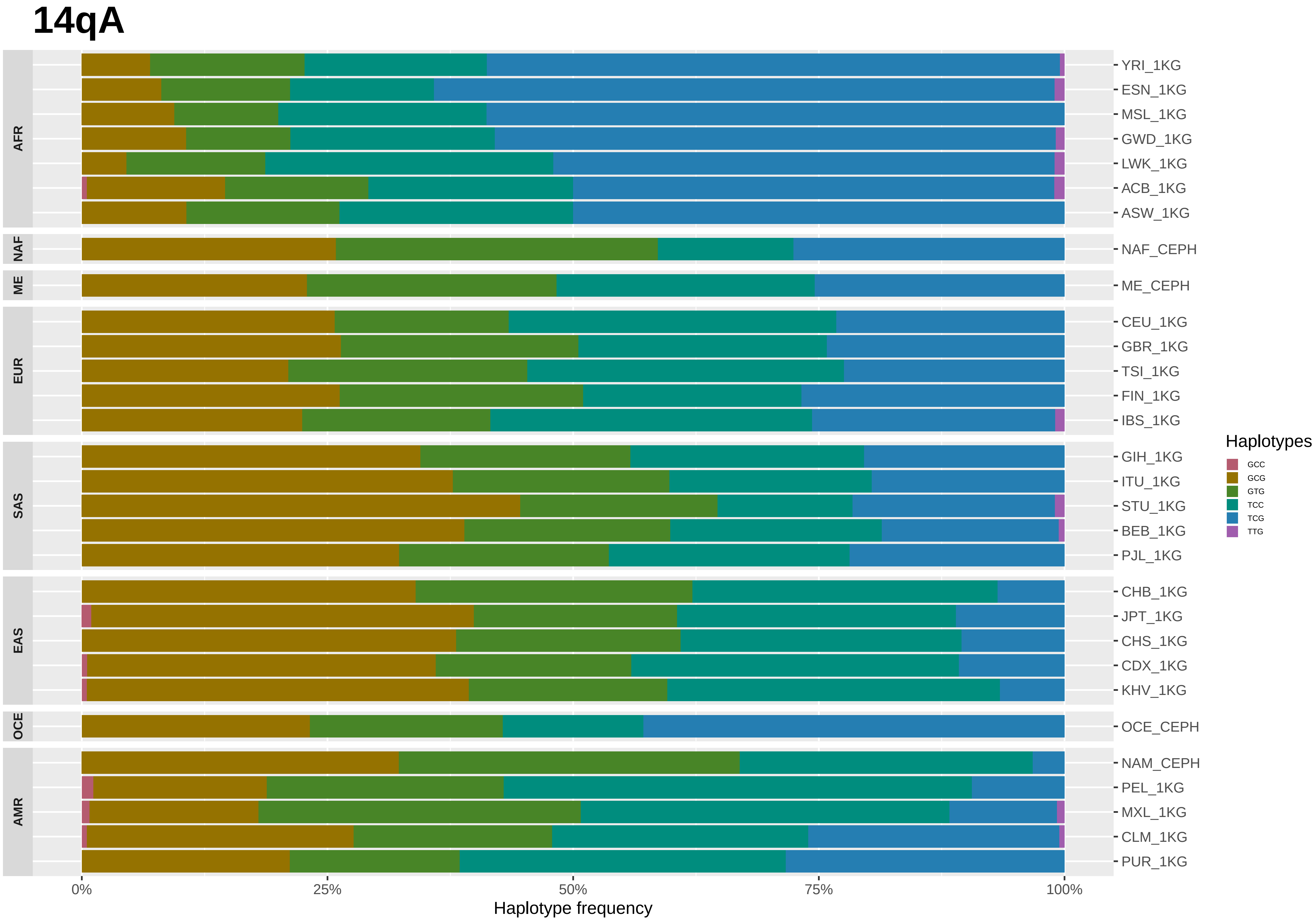

14qA
AFR
NAF
ME
EUR
SAS
EAS
OCE
AMR
YRI_1KG
ESN_1KG
MSL_1KG
GWD_1KG
LWK_1KG
ACB_1KG
ASW_1KG
NAF_CEPH
ME_CEPH
CEU_1KG
GBR_1KG
TSI_1KG
FIN_1KG
IBS_1KG
GIH_1KG
ITU_1KG
STU_1KG
BEB_1KG
PJL_1KG
CHB_1KG
JPT_1KG
CHS_1KG
CDX_1KG
KHV_1KG
OCE_CEPH
NAM_CEPH
PEL_1KG
MXL_1KG
CLM_1KG
PUR_1KG
0%
25%
50%
75%
100%
Haplotype frequency
Haplotypes
GCC
GCG
GTG
TCC
TCG
TTG

# 14qB

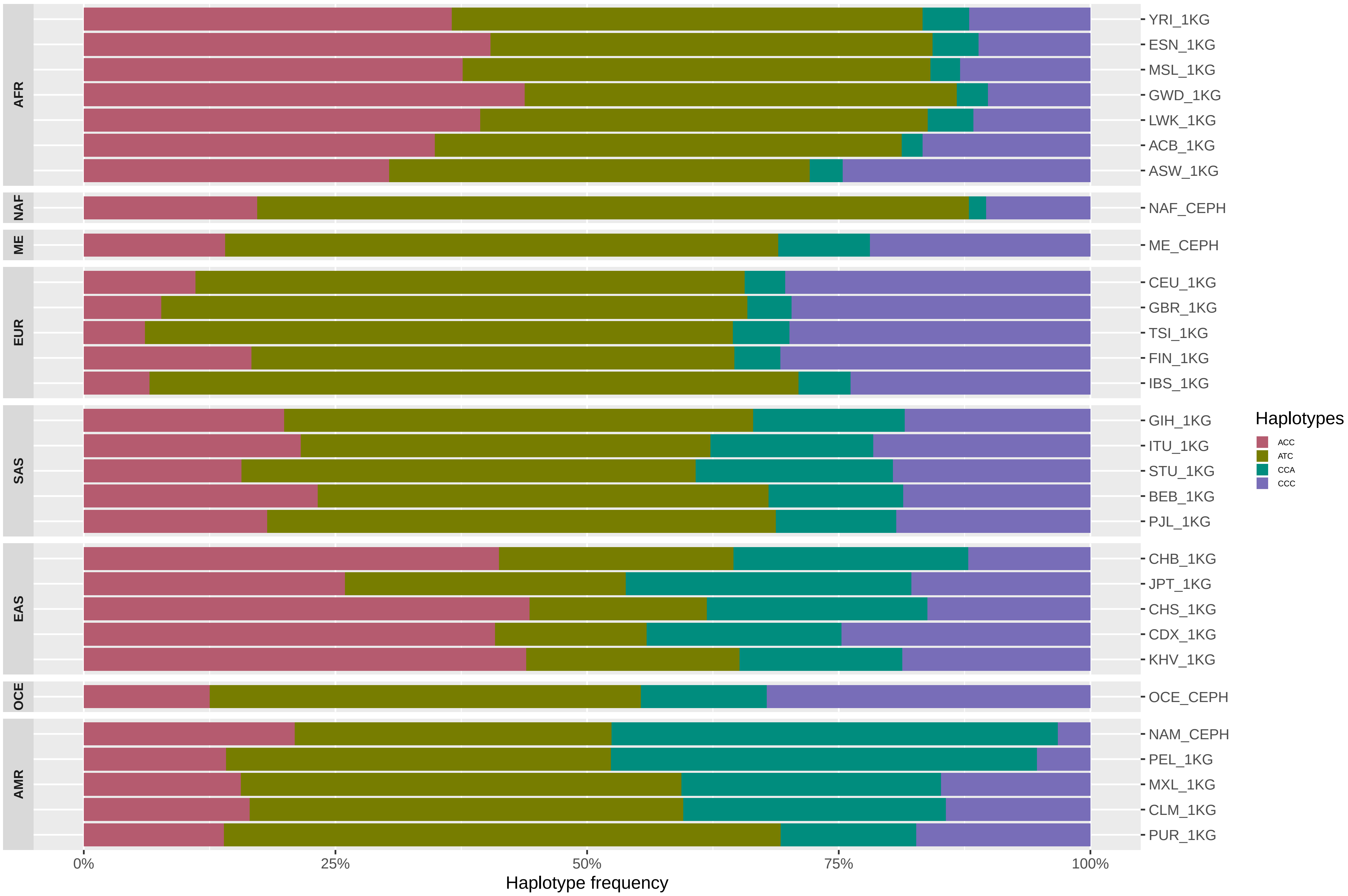

AFR
NAF
ME
EUR
SAS
EAS
OCE
AMR
YRI_1KG
ESN_1KG
MSL_1KG
GWD_1KG
LWK_1KG
ACB_1KG
ASW_1KG
NAF_CEPH
ME_CEPH
CEU_1KG
GBR_1KG
TSI_1KG
FIN_1KG
IBS_1KG
GIH_1KG
ITU_1KG
STU_1KG
BEB_1KG
PJL_1KG
CHB_1KG
JPT_1KG
CHS_1KG
CDX_1KG
KHV_1KG
OCE_CEPH
NAM_CEPH
PEL_1KG
MXL_1KG
CLM_1KG
PUR_1KG
0%
25%
50%
75%
100%
Haplotype frequency
Haplotypes
ACC
ATC
CCA
CCC

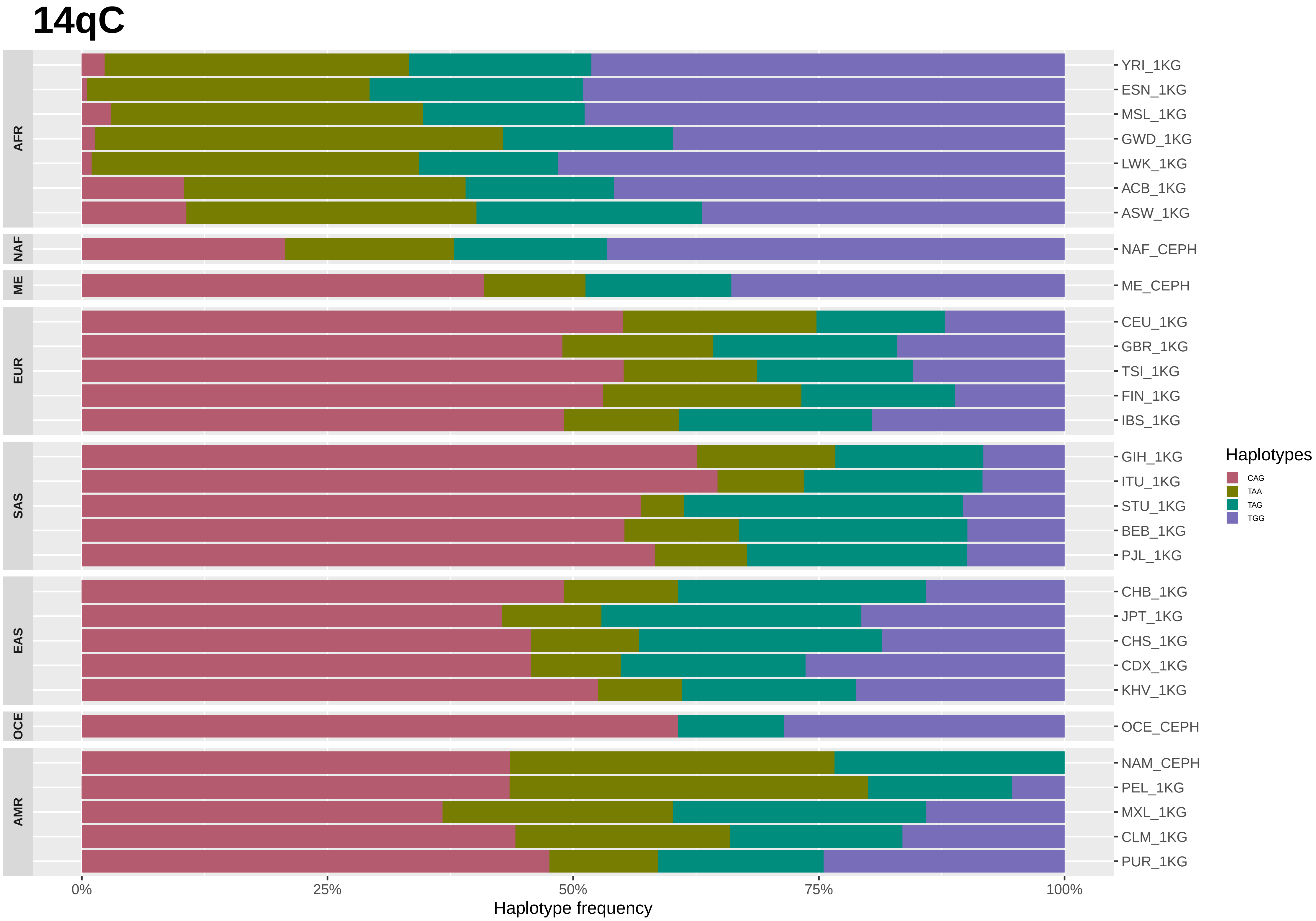
14qC
AFR
NAF
ME
EUR
SAS
EAS
OCE
AMR
YRI_1KG
ESN_1KG
MSL_1KG
GWD_1KG
LWK_1KG
ACB_1KG
ASW_1KG
NAF_CEPH
ME_CEPH
CEU_1KG
GBR_1KG
TSI_1KG
FIN_1KG
IBS_1KG
GIH_1KG
ITU_1KG
STU_1KG
BEB_1KG
PJL_1KG
CHB_1KG
JPT_1KG
CHS_1KG
CDX_1KG
KHV_1KG
OCE_CEPH
NAM_CEPH
PEL_1KG
MXL_1KG
CLM_1KG
PUR_1KG
0%
25%
50%
75%
100%
Haplotype frequency
Haplotypes
CAG
TAA
TAG
TGG

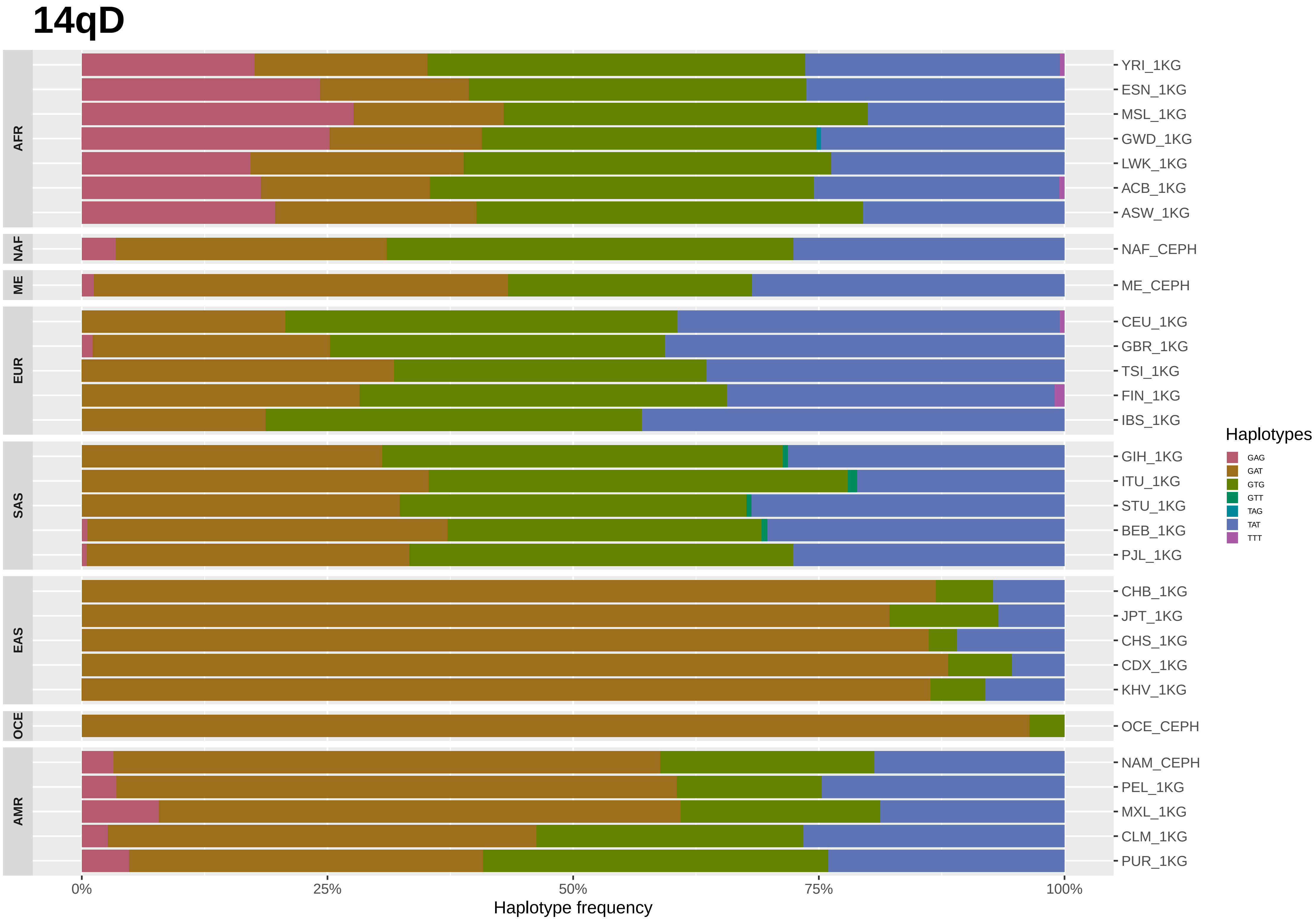

14qD
AFR
NAF
ME
EUR
SAS
EAS
OCE
AMR
YRI_1KG
ESN_1KG
MSL_1KG
GWD_1KG
LWK_1KG
ACB_1KG
ASW_1KG
NAF_CEPH
ME_CEPH
CEU_1KG
GBR_1KG
TSI_1KG
FIN_1KG
IBS_1KG
GIH_1KG
ITU_1KG
STU_1KG
BEB_1KG
PJL_1KG
CHB_1KG
JPT_1KG
CHS_1KG
CDX_1KG
KHV_1KG
OCE_CEPH
NAM_CEPH
PEL_1KG
MXL_1KG
CLM_1KG
PUR_1KG
0%
25%
50%
75%
100%
Haplotype frequency
Haplotypes
GAG
GAT
GTG
GTT
TAG
TAT
TTT

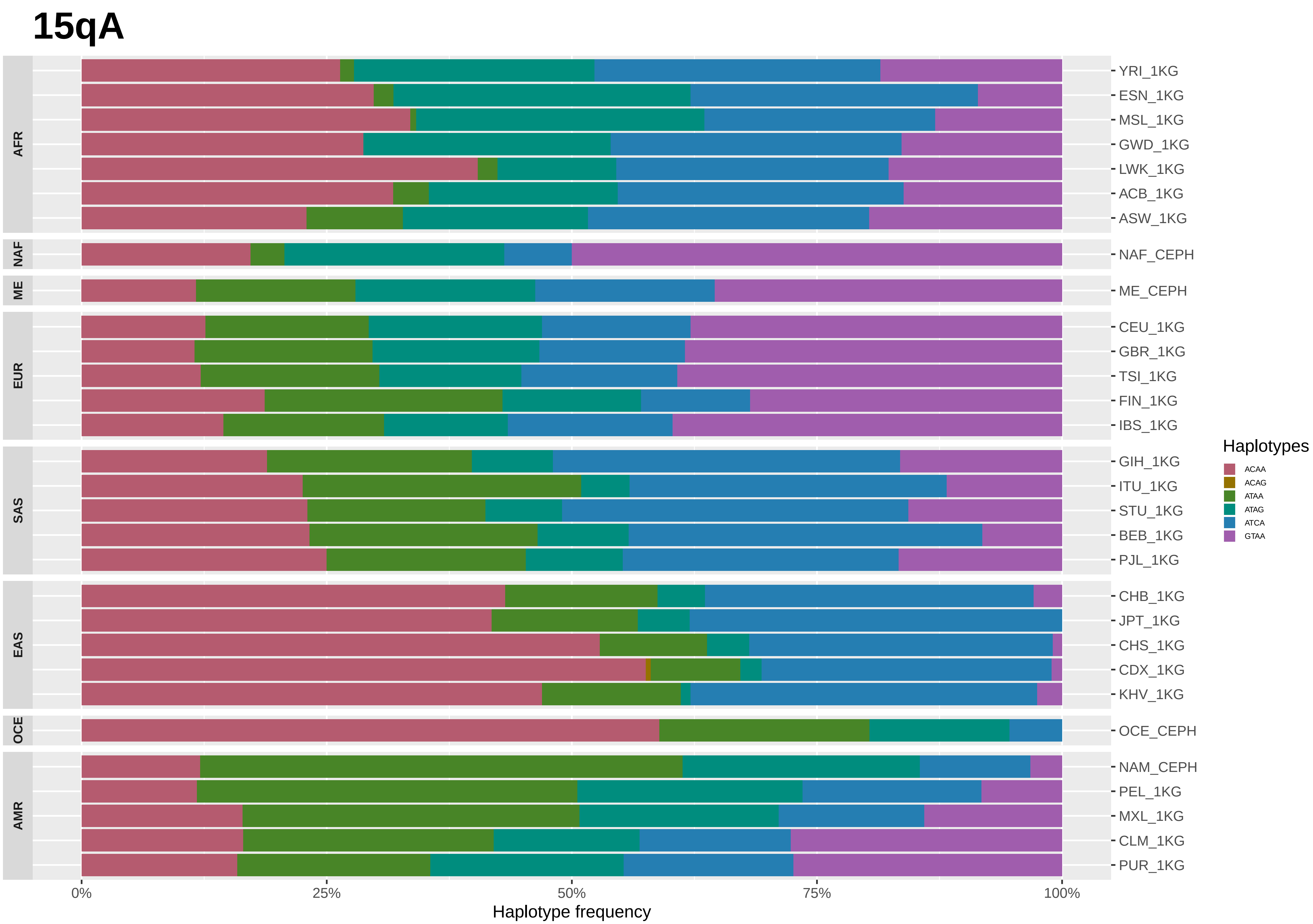

15qA
AFR
NAF
ME
EUR
SAS
EAS
OCE
AMR
YRI_1KG
ESN_1KG
MSL_1KG
GWD_1KG
LWK_1KG
ACB_1KG
ASW_1KG
NAF_CEPH
ME_CEPH
CEU_1KG
GBR_1KG
TSI_1KG
FIN_1KG
IBS_1KG
GIH_1KG
ITU_1KG
STU_1KG
BEB_1KG
PJL_1KG
CHB_1KG
JPT_1KG
CHS_1KG
CDX_1KG
KHV_1KG
OCE_CEPH
NAM_CEPH
PEL_1KG
MXL_1KG
CLM_1KG
PUR_1KG
0%
25%
50%
75%
100%
Haplotype frequency
Haplotypes
ACAA
ACAG
ATAA
ATAG
ATCA
GTAA

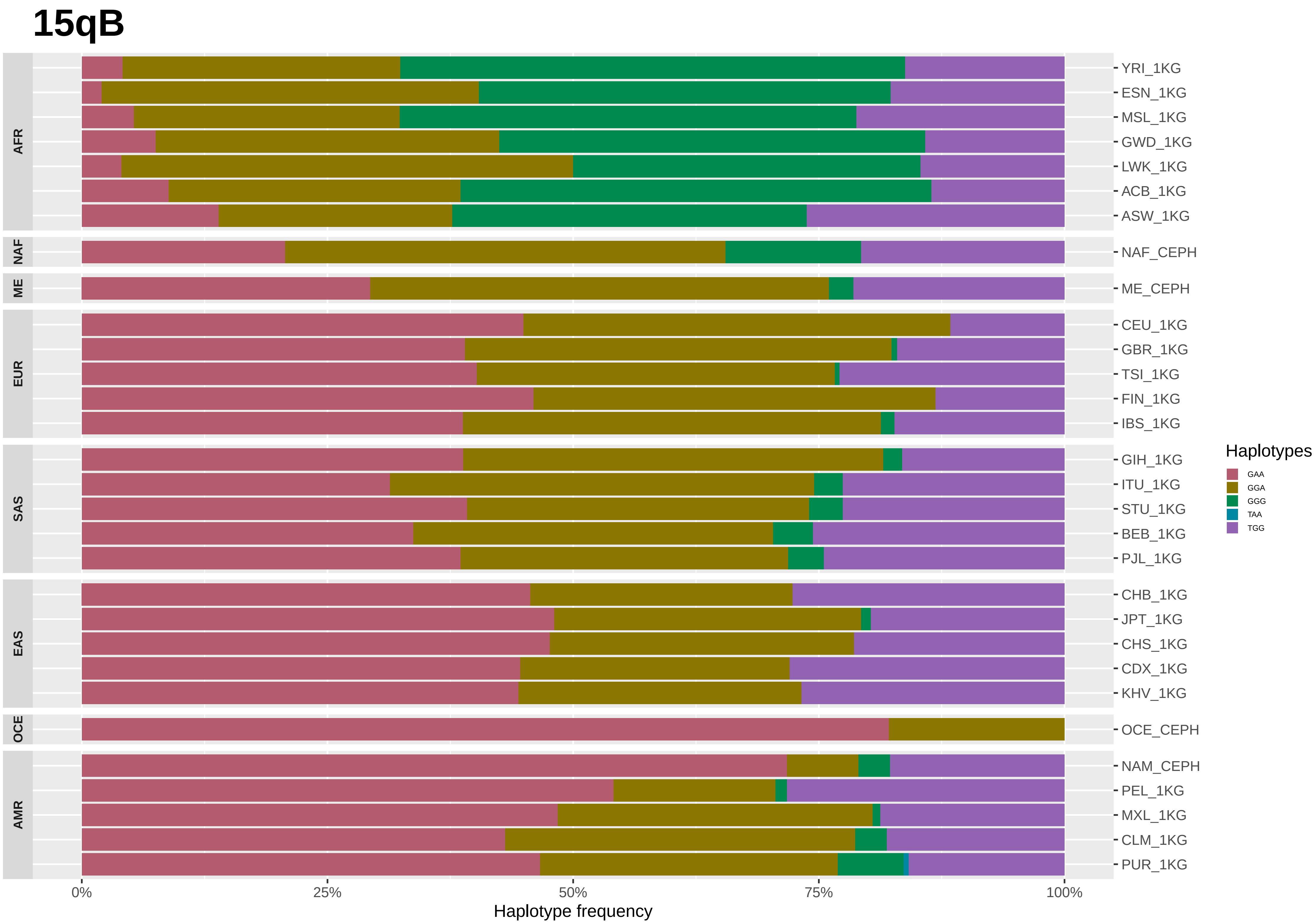

15qB
AFR
NAF
ME
EUR
SAS
EAS
OCE
AMR
YRI_1KG
ESN_1KG
MSL_1KG
GWD_1KG
LWK_1KG
ACB_1KG
ASW_1KG
NAF_CEPH
ME_CEPH
CEU_1KG
GBR_1KG
TSI_1KG
FIN_1KG
IBS_1KG
GIH_1KG
ITU_1KG
STU_1KG
BEB_1KG
PJL_1KG
CHB_1KG
JPT_1KG
CHS_1KG
CDX_1KG
KHV_1KG
OCE_CEPH
NAM_CEPH
PEL_1KG
MXL_1KG
CLM_1KG
PUR_1KG
0%
25%
50%
75%
100%
Haplotype frequency
Haplotypes
GAA
GGA
GGG
TAA
TGG

# 15qC

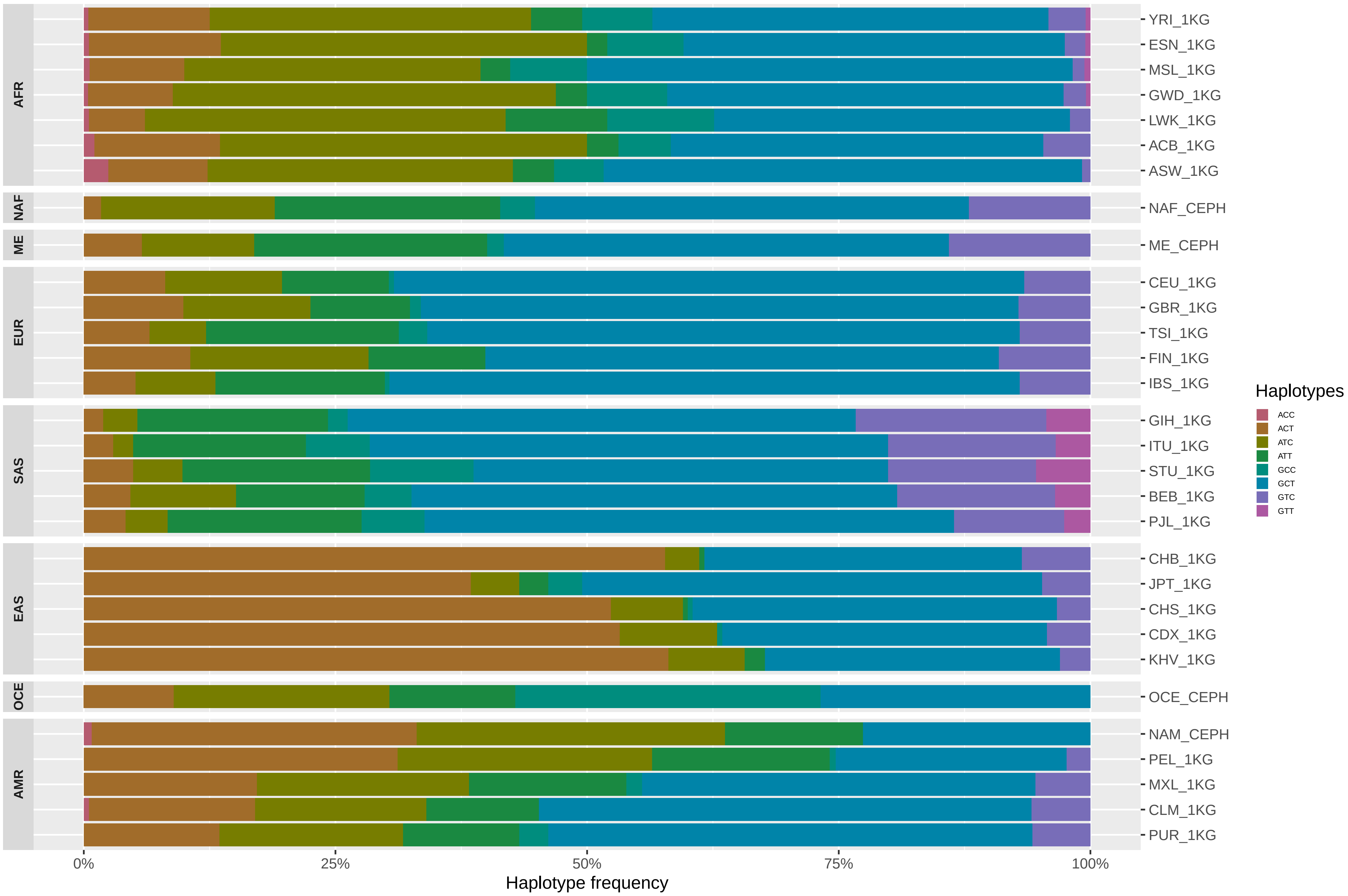

AFR
NAF
ME
EUR
SAS
EAS
OCE
AMR
YRI_1KG
ESN_1KG
MSL_1KG
GWD_1KG
LWK_1KG
ACB_1KG
ASW_1KG
NAF_CEPH
ME_CEPH
CEU_1KG
GBR_1KG
TSI_1KG
FIN_1KG
IBS_1KG
GIH_1KG
ITU_1KG
STU_1KG
BEB_1KG
PJL_1KG
CHB_1KG
JPT_1KG
CHS_1KG
CDX_1KG
KHV_1KG
OCE_CEPH
NAM_CEPH
PEL_1KG
MXL_1KG
CLM_1KG
PUR_1KG
0%
25%
50%
75%
100%
Haplotype frequency
Haplotypes
ACC
ACT
ATC
ATT
GCC
GCT
GTC
GTT

# 15qD

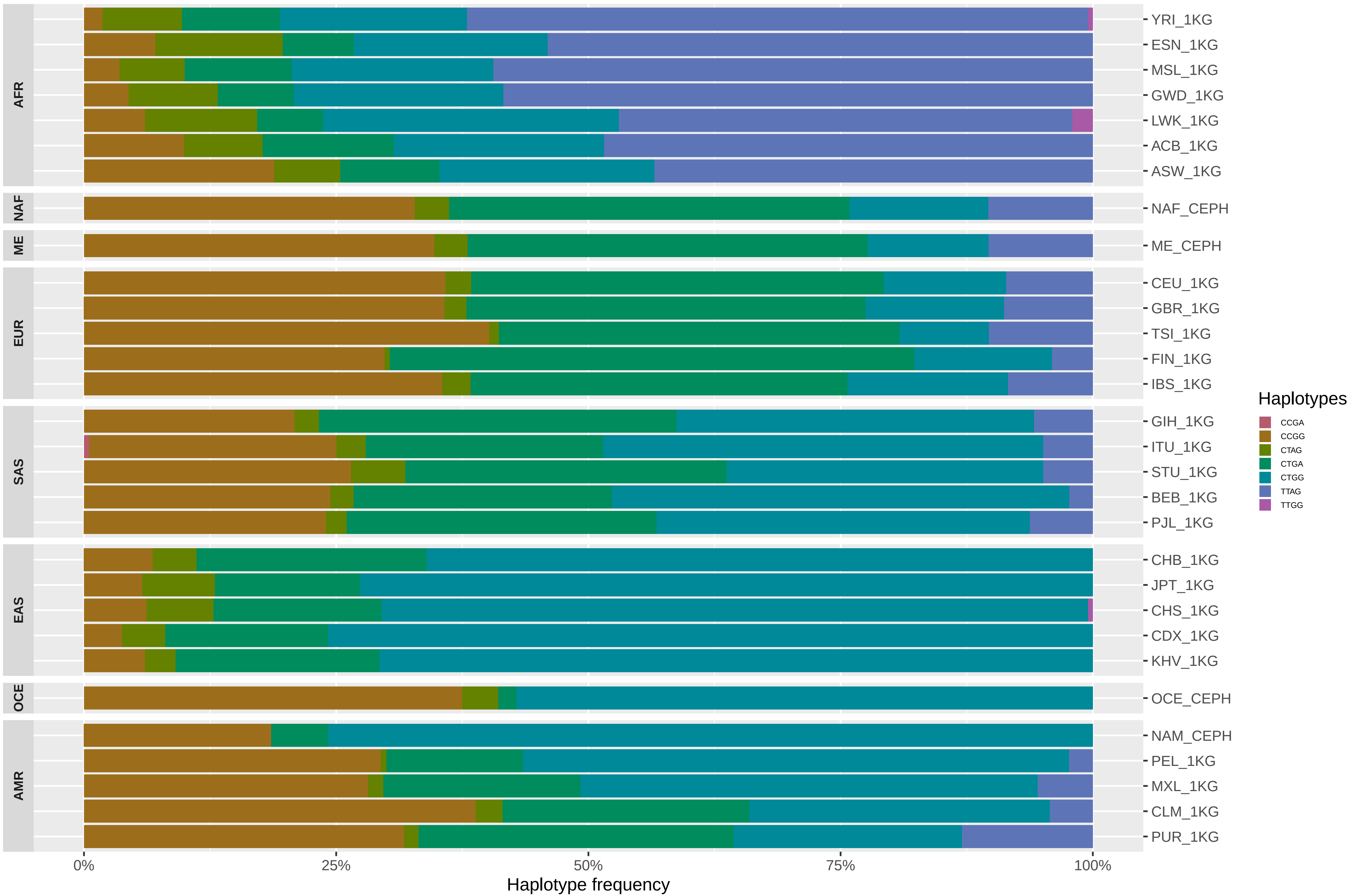

AFR
NAF
ME
EUR
SAS
EAS
OCE
AMR
YRI_1KG
ESN_1KG
MSL_1KG
GWD_1KG
LWK_1KG
ACB_1KG
ASW_1KG
NAF_CEPH
ME_CEPH
CEU_1KG
GBR_1KG
TSI_1KG
FIN_1KG
IBS_1KG
GIH_1KG
ITU_1KG
STU_1KG
BEB_1KG
PJL_1KG
CHB_1KG
JPT_1KG
CHS_1KG
CDX_1KG
KHV_1KG
OCE_CEPH
NAM_CEPH
PEL_1KG
MXL_1KG
CLM_1KG
PUR_1KG
0%
25%
50%
75%
100%
Haplotype frequency
Haplotypes
CCGA
CCGG
CTAG
CTGA
CTGG
TTAG
TTGG

# 16pA

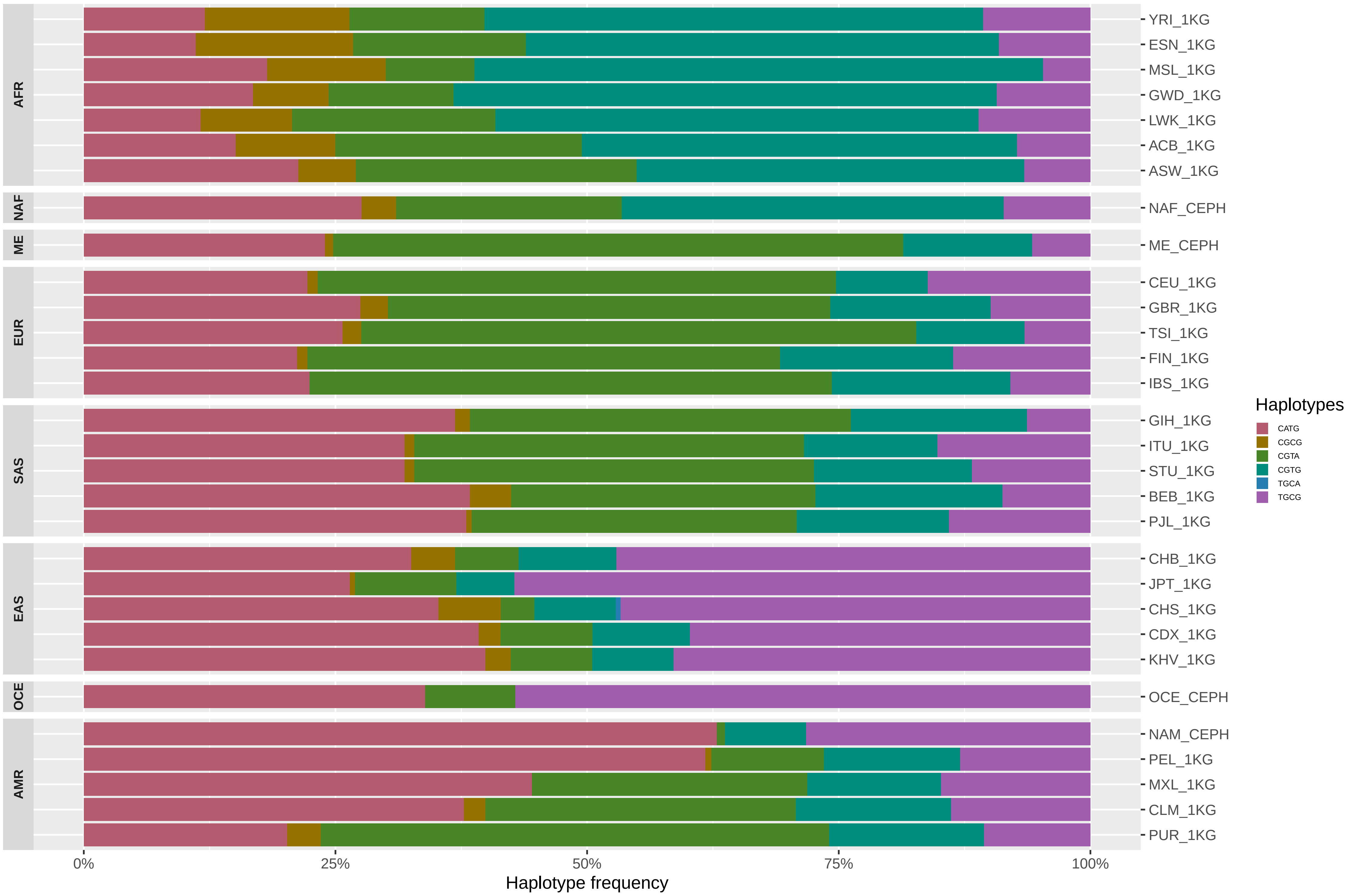

AFR
NAF
ME
EUR
SAS
EAS
OCE
AMR
YRI_1KG
ESN_1KG
MSL_1KG
GWD_1KG
LWK_1KG
ACB_1KG
ASW_1KG
NAF_CEPH
ME_CEPH
CEU_1KG
GBR_1KG
TSI_1KG
FIN_1KG
IBS_1KG
GIH_1KG
ITU_1KG
STU_1KG
BEB_1KG
PJL_1KG
CHB_1KG
JPT_1KG
CHS_1KG
CDX_1KG
KHV_1KG
OCE_CEPH
NAM_CEPH
PEL_1KG
MXL_1KG
CLM_1KG
PUR_1KG
0%
25%
50%
75%
100%
Haplotype frequency
Haplotypes
CATG
CGCG
CGTA
CGTG
TGCA
TGCG

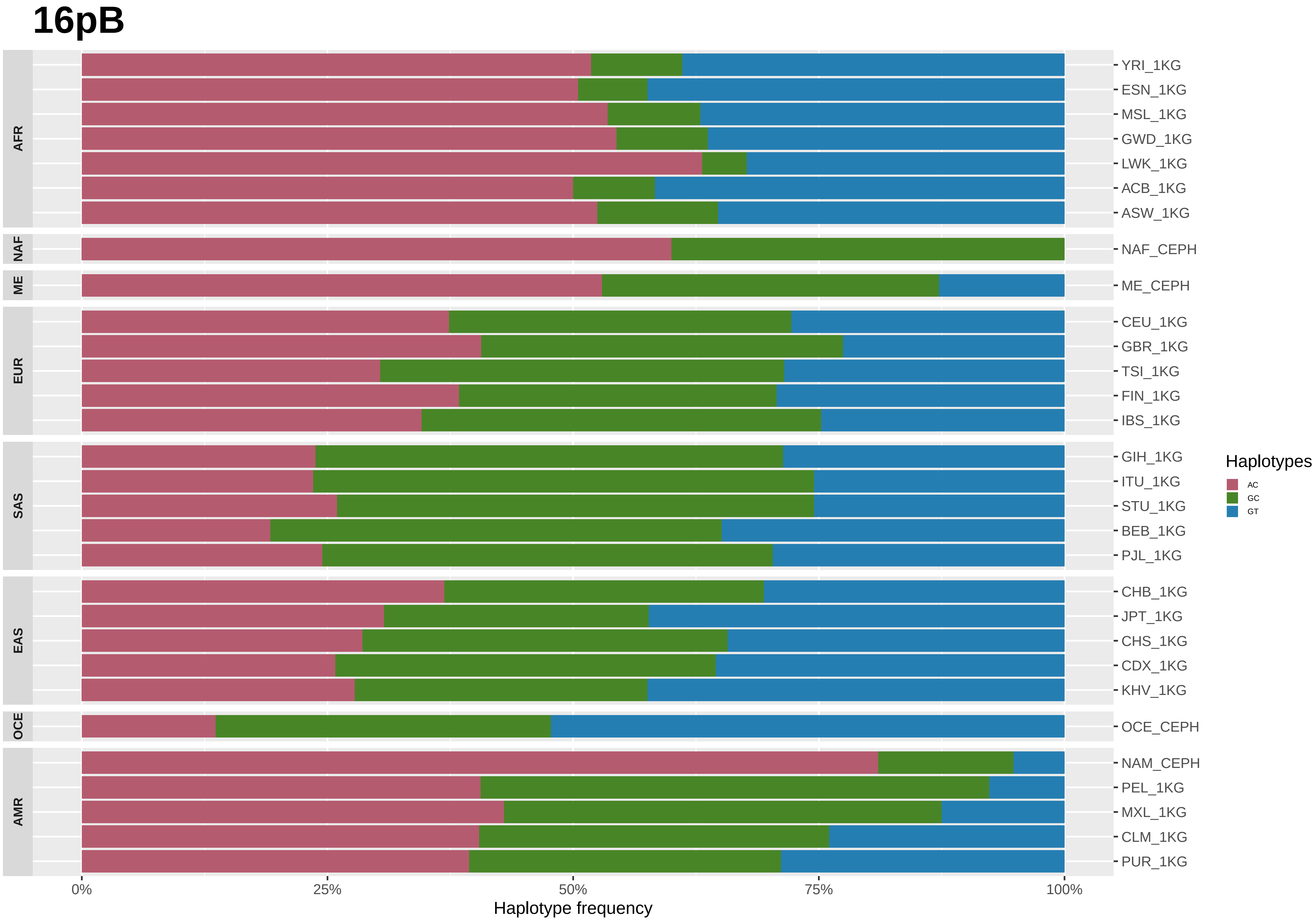
16pB
AFR
NAF
ME
EUR
SAS
EAS
OCE
AMR
YRI_1KG
ESN_1KG
MSL_1KG
GWD_1KG
LWK_1KG
ACB_1KG
ASW_1KG
NAF_CEPH
ME_CEPH
CEU_1KG
GBR_1KG
TSI_1KG
FIN_1KG
IBS_1KG
GIH_1KG
ITU_1KG
STU_1KG
BEB_1KG
PJL_1KG
CHB_1KG
JPT_1KG
CHS_1KG
CDX_1KG
KHV_1KG
OCE_CEPH
NAM_CEPH
PEL_1KG
MXL_1KG
CLM_1KG
PUR_1KG
0%
25%
50%
75%
100%
Haplotype frequency
Haplotypes
AC
GC
GT

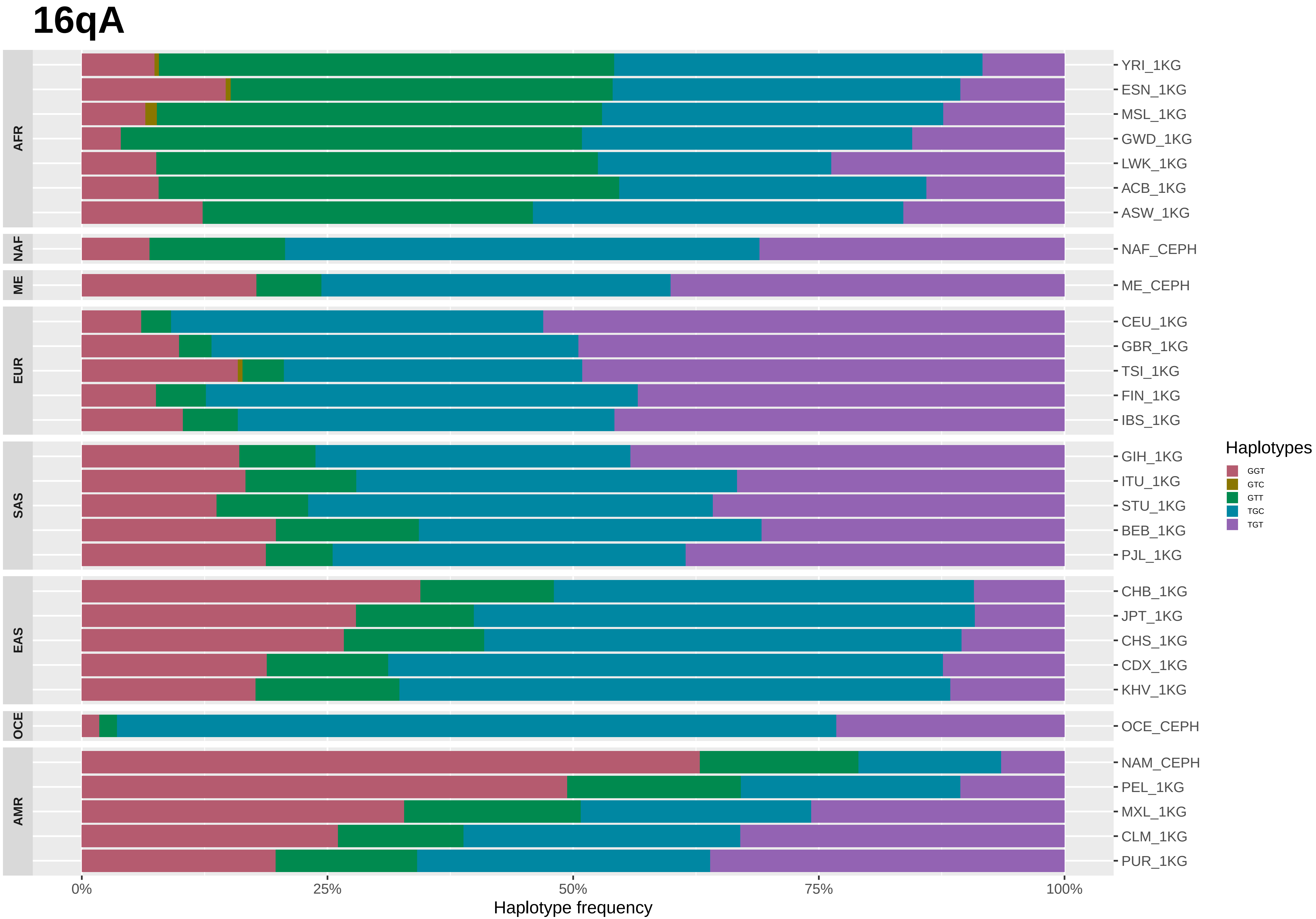
16qA
AFR
NAF
ME
EUR
SAS
EAS
OCE
AMR
YRI_1KG
ESN_1KG
MSL_1KG
GWD_1KG
LWK_1KG
ACB_1KG
ASW_1KG
NAF_CEPH
ME_CEPH
CEU_1KG
GBR_1KG
TSI_1KG
FIN_1KG
IBS_1KG
GIH_1KG
ITU_1KG
STU_1KG
BEB_1KG
PJL_1KG
CHB_1KG
JPT_1KG
CHS_1KG
CDX_1KG
KHV_1KG
OCE_CEPH
NAM_CEPH
PEL_1KG
MXL_1KG
CLM_1KG
PUR_1KG
0%
25%
50%
75%
100%
Haplotype frequency
Haplotypes
GGT
GTC
GTT
TGC
TGT

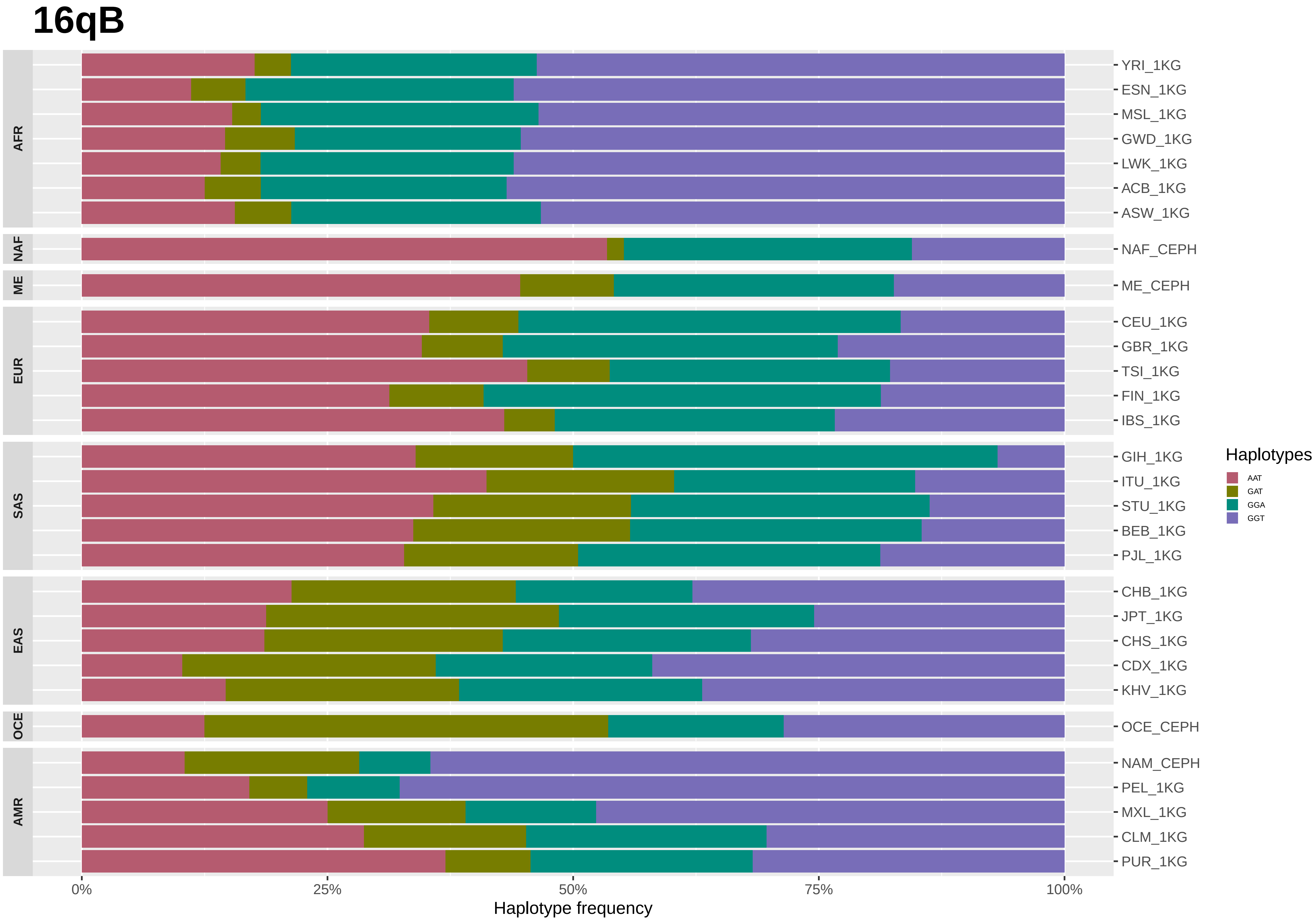
16qB
AFR
NAF
ME
EUR
SAS
EAS
OCE
AMR
YRI_1KG
ESN_1KG
MSL_1KG
GWD_1KG
LWK_1KG
ACB_1KG
ASW_1KG
NAF_CEPH
ME_CEPH
CEU_1KG
GBR_1KG
TSI_1KG
FIN_1KG
IBS_1KG
GIH_1KG
ITU_1KG
STU_1KG
BEB_1KG
PJL_1KG
CHB_1KG
JPT_1KG
CHS_1KG
CDX_1KG
KHV_1KG
OCE_CEPH
NAM_CEPH
PEL_1KG
MXL_1KG
CLM_1KG
PUR_1KG
0%
25%
50%
75%
100%
Haplotype frequency
Haplotypes
AAT
GAT
GGA
GGT

# 17pA

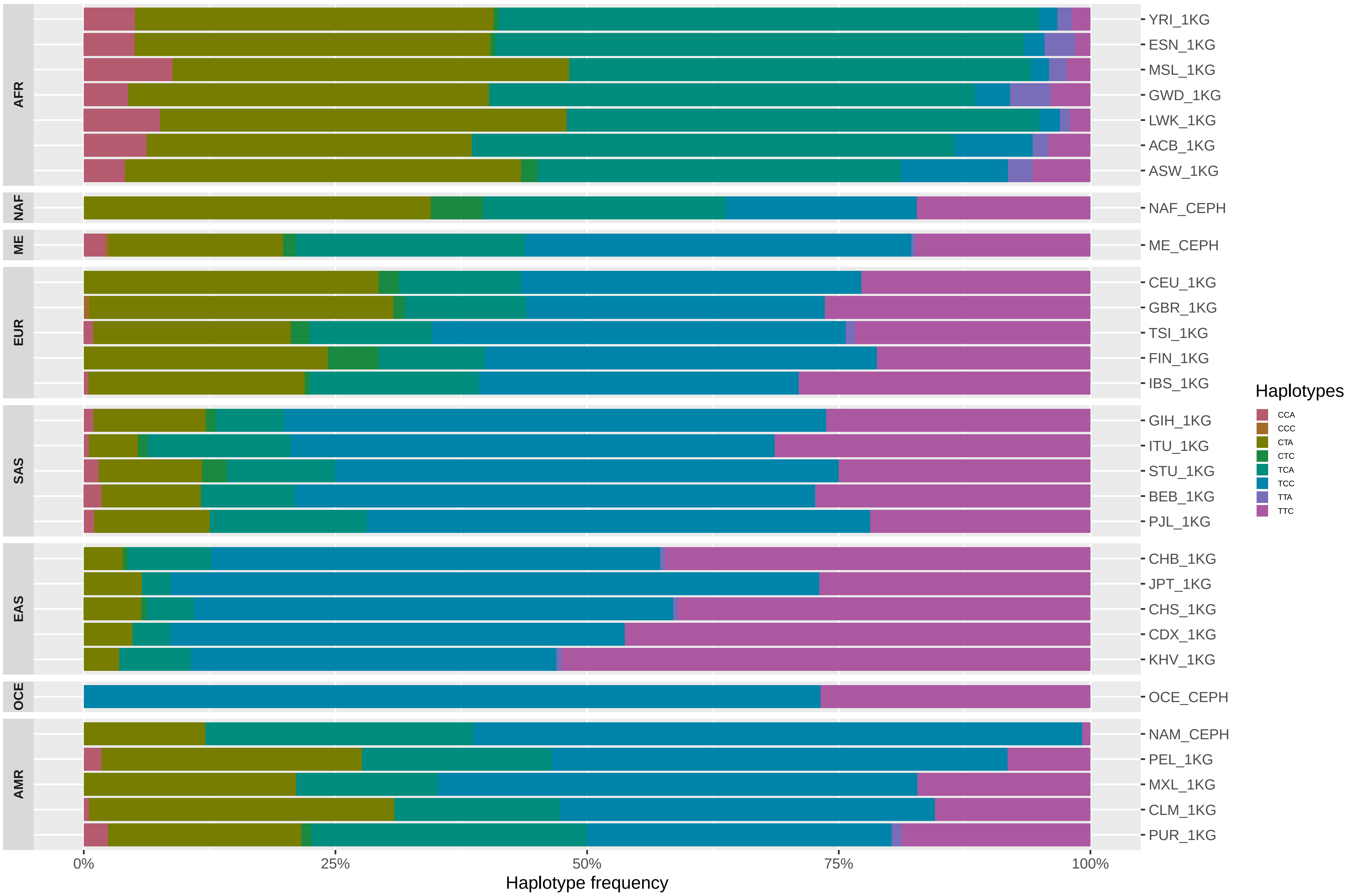

AFR
NAF
ME
EUR
SAS
EAS
OCE
AMR
YRI_1KG
ESN_1KG
MSL_1KG
GWD_1KG
LWK_1KG
ACB_1KG
ASW_1KG
NAF_CEPH
ME_CEPH
CEU_1KG
GBR_1KG
TSI_1KG
FIN_1KG
IBS_1KG
GIH_1KG
ITU_1KG
STU_1KG
BEB_1KG
PJL_1KG
CHB_1KG
JPT_1KG
CHS_1KG
CDX_1KG
KHV_1KG
OCE_CEPH
NAM_CEPH
PEL_1KG
MXL_1KG
CLM_1KG
PUR_1KG
0%
25%
50%
75%
100%
Haplotype frequency
Haplotypes
CCA
CCC
CTA
CTC
TCA
TCC
TTA
TTC

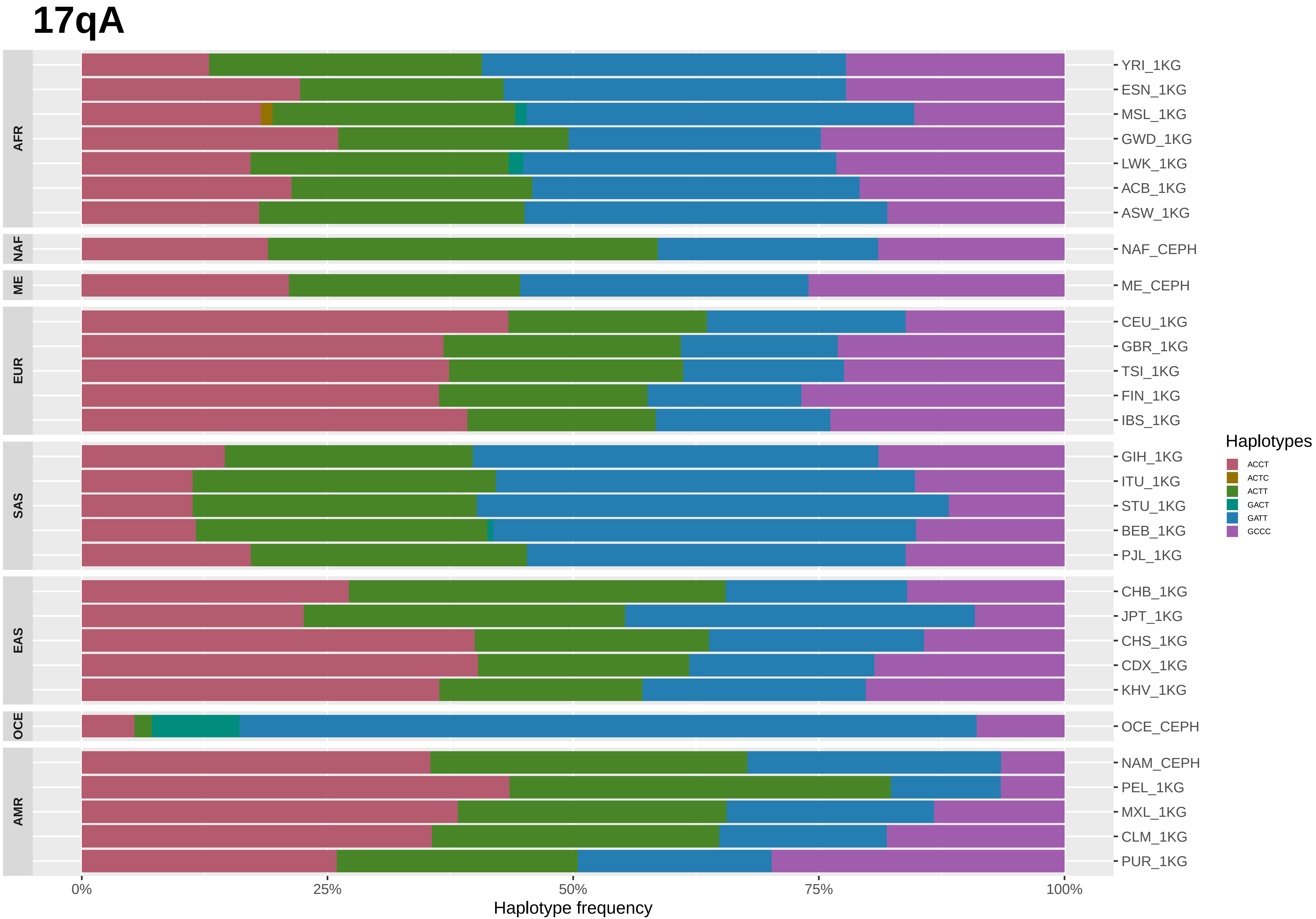

17qA
AFR
NAF
ME
EUR
SAS
EAS
OCE
AMR
YRI_1KG
ESN_1KG
MSL_1KG
GWD_1KG
LWK_1KG
ACB_1KG
ASW_1KG
NAF_CEPH
ME_CEPH
CEU_1KG
GBR_1KG
TSI_1KG
FIN_1KG
IBS_1KG
GIH_1KG
ITU_1KG
STU_1KG
BEB_1KG
PJL_1KG
CHB_1KG
JPT_1KG
CHS_1KG
CDX_1KG
KHV_1KG
OCE_CEPH
NAM_CEPH
PEL_1KG
MXL_1KG
CLM_1KG
PUR_1KG
0%
25%
50%
75%
100%
Haplotype frequency
Haplotypes
ACCT
ACTC
ACTT
GACT
GATT
GCCC

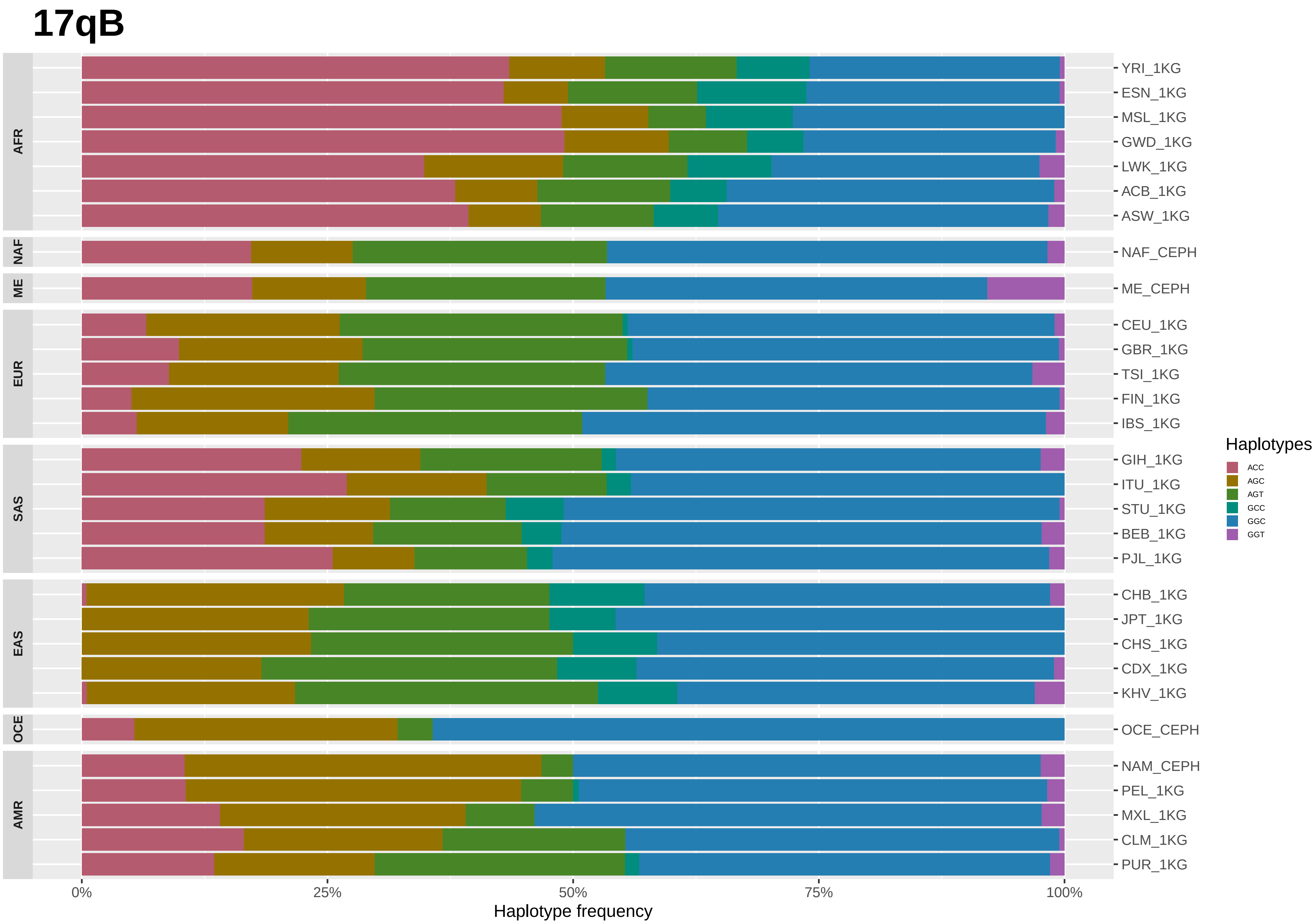

17qB
AFR
YRI_1KG
ESN_1KG
MSL_1KG
GWD_1KG
LWK_1KG
ACB_1KG
ASW_1KG
NAF
NAF_CEPH
ME
ME_CEPH
EUR
CEU_1KG
GBR_1KG
TSI_1KG
FIN_1KG
IBS_1KG
SAS
GIH_1KG
ITU_1KG
STU_1KG
BEB_1KG
PJL_1KG
EAS
CHB_1KG
JPT_1KG
CHS_1KG
CDX_1KG
KHV_1KG
OCE
OCE_CEPH
AMR
NAM_CEPH
PEL_1KG
MXL_1KG
CLM_1KG
PUR_1KG
0%
25%
50%
75%
100%
Haplotype frequency
Haplotypes
ACC
AGC
AGT
GCC
GGC
GGT

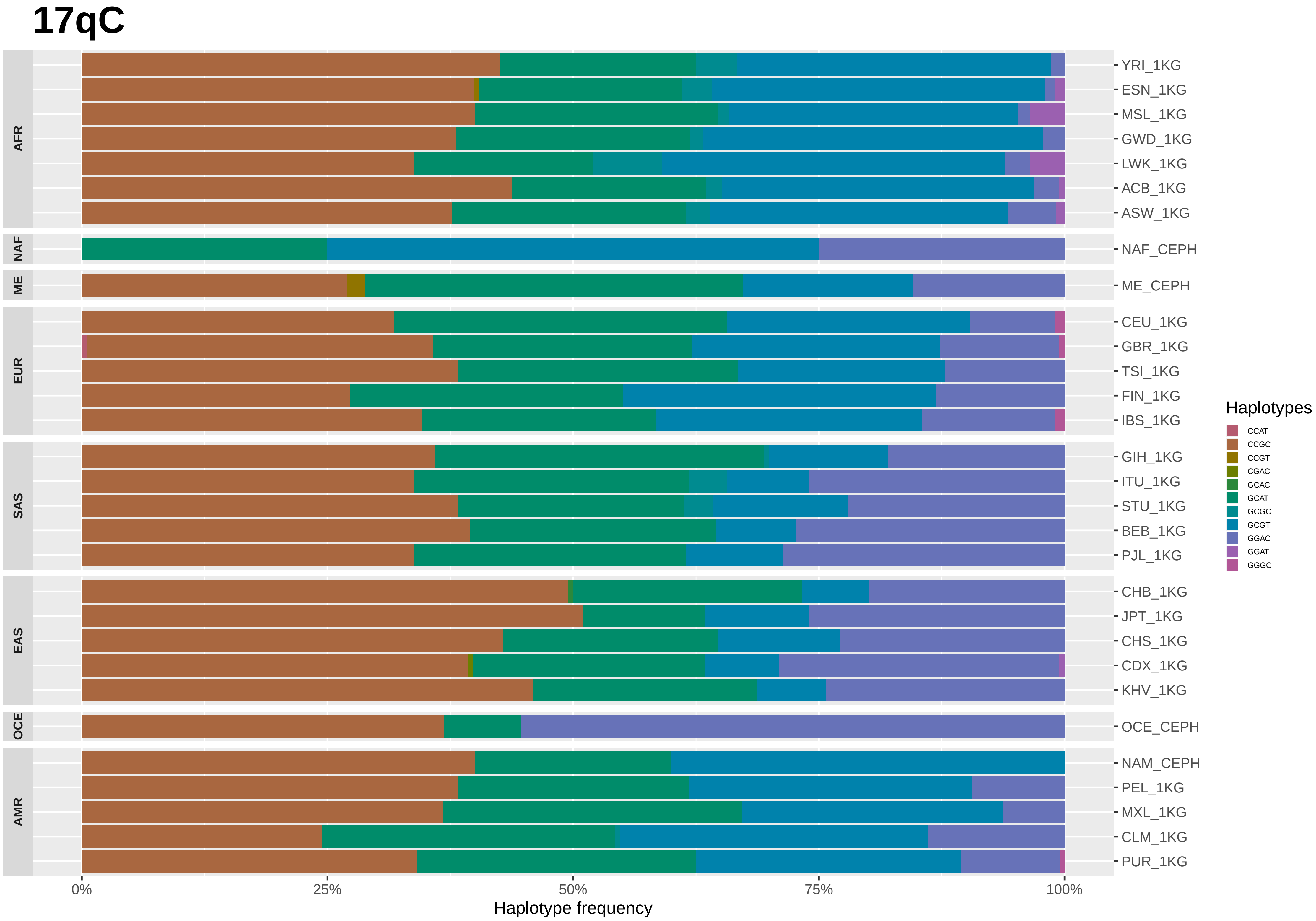

17qC
AFR
NAF
ME
EUR
SAS
EAS
OCE
AMR
YRI_1KG
ESN_1KG
MSL_1KG
GWD_1KG
LWK_1KG
ACB_1KG
ASW_1KG
NAF_CEPH
ME_CEPH
CEU_1KG
GBR_1KG
TSI_1KG
FIN_1KG
IBS_1KG
GIH_1KG
ITU_1KG
STU_1KG
BEB_1KG
PJL_1KG
CHB_1KG
JPT_1KG
CHS_1KG
CDX_1KG
KHV_1KG
OCE_CEPH
NAM_CEPH
PEL_1KG
MXL_1KG
CLM_1KG
PUR_1KG
0%
25%
50%
75%
100%
Haplotype frequency
Haplotypes
CCAT
CCGC
CCGT
CGAC
GCAC
GCAT
GCGC
GCGT
GGAC
GGAT
GGGC

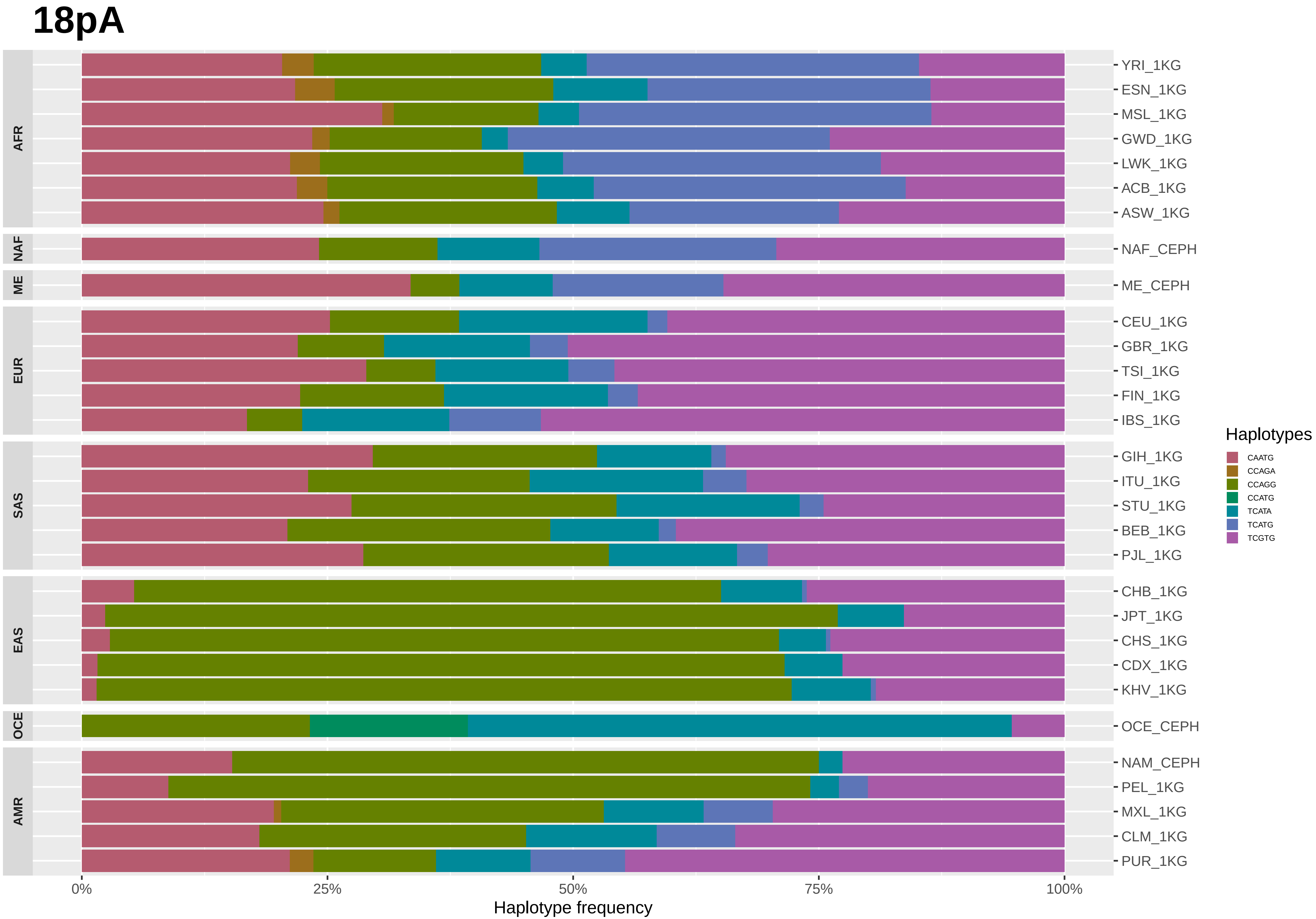

18pA
AFR
NAF
ME
EUR
SAS
EAS
OCE
AMR
YRI_1KG
ESN_1KG
MSL_1KG
GWD_1KG
LWK_1KG
ACB_1KG
ASW_1KG
NAF_CEPH
ME_CEPH
CEU_1KG
GBR_1KG
TSI_1KG
FIN_1KG
IBS_1KG
GIH_1KG
ITU_1KG
STU_1KG
BEB_1KG
PJL_1KG
CHB_1KG
JPT_1KG
CHS_1KG
CDX_1KG
KHV_1KG
OCE_CEPH
NAM_CEPH
PEL_1KG
MXL_1KG
CLM_1KG
PUR_1KG
0%
25%
50%
75%
100%
Haplotype frequency
Haplotypes
CAATG
CCAGA
CCAGG
CCATG
TCATA
TCATG
TCGTG

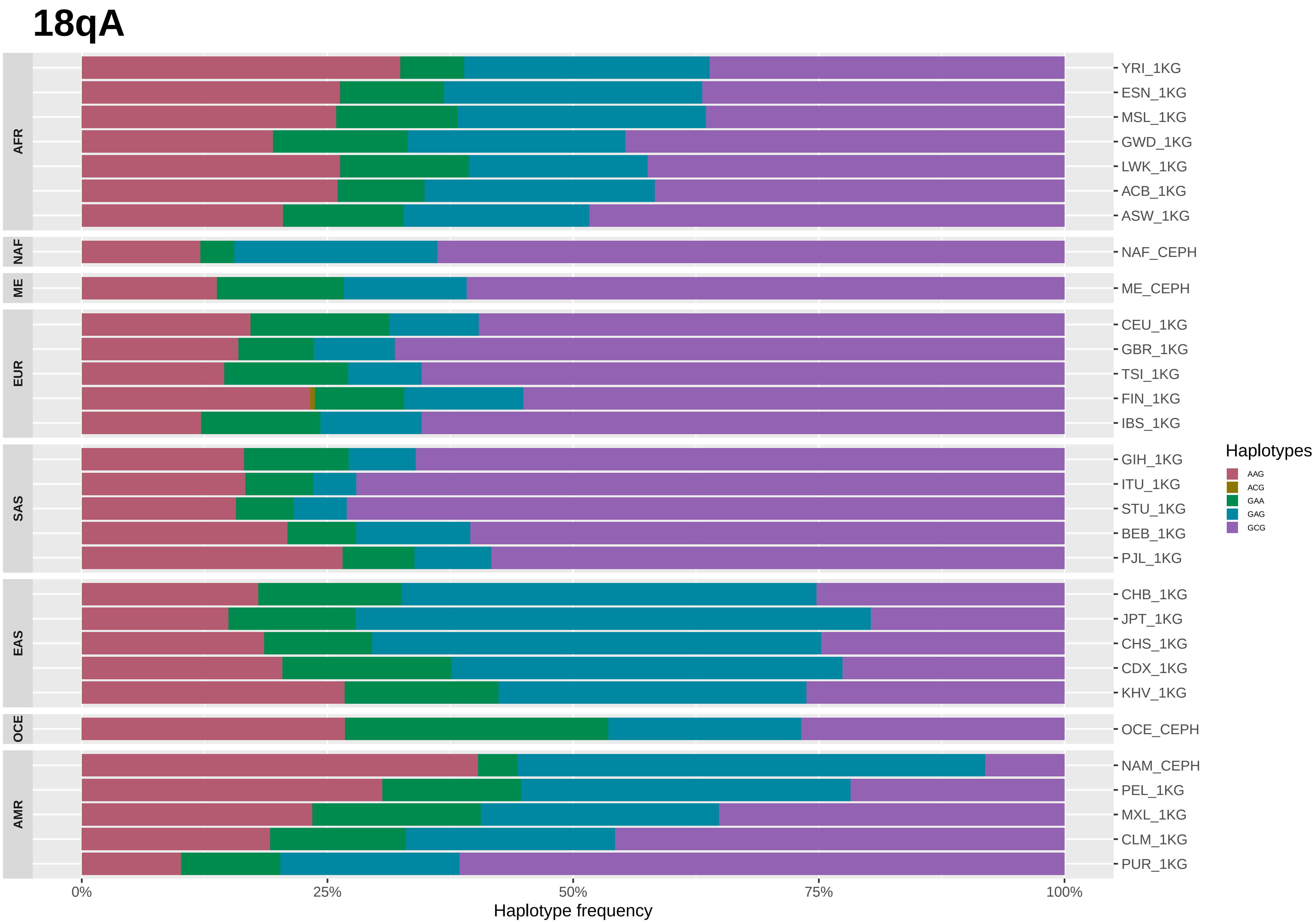
18qA
AFR
NAF
ME
EUR
SAS
EAS
OCE
AMR
YRI_1KG
ESN_1KG
MSL_1KG
GWD_1KG
LWK_1KG
ACB_1KG
ASW_1KG
NAF_CEPH
ME_CEPH
CEU_1KG
GBR_1KG
TSI_1KG
FIN_1KG
IBS_1KG
GIH_1KG
ITU_1KG
STU_1KG
BEB_1KG
PJL_1KG
CHB_1KG
JPT_1KG
CHS_1KG
CDX_1KG
KHV_1KG
OCE_CEPH
NAM_CEPH
PEL_1KG
MXL_1KG
CLM_1KG
PUR_1KG
0%
25%
50%
75%
100%
Haplotype frequency
Haplotypes
AAG
ACG
GAA
GAG
GCG

# 18qB

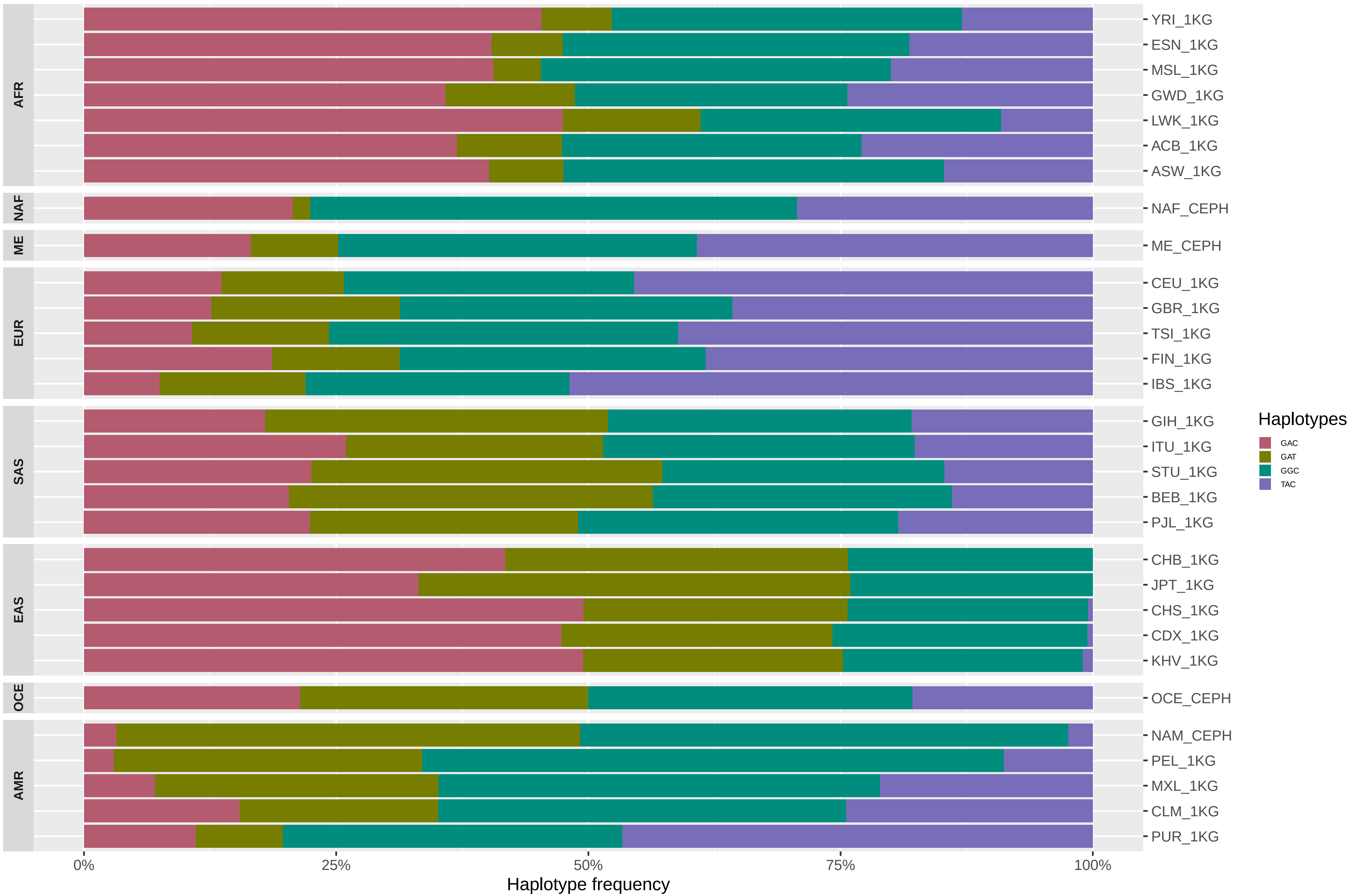

AFR
NAF
ME
EUR
SAS
EAS
OCE
AMR
YRI_1KG
ESN_1KG
MSL_1KG
GWD_1KG
LWK_1KG
ACB_1KG
ASW_1KG
NAF_CEPH
ME_CEPH
CEU_1KG
GBR_1KG
TSI_1KG
FIN_1KG
IBS_1KG
GIH_1KG
ITU_1KG
STU_1KG
BEB_1KG
PJL_1KG
CHB_1KG
JPT_1KG
CHS_1KG
CDX_1KG
KHV_1KG
OCE_CEPH
NAM_CEPH
PEL_1KG
MXL_1KG
CLM_1KG
PUR_1KG
0%
25%
50%
75%
100%
Haplotype frequency
Haplotypes
GAC
GAT
GGC
TAC

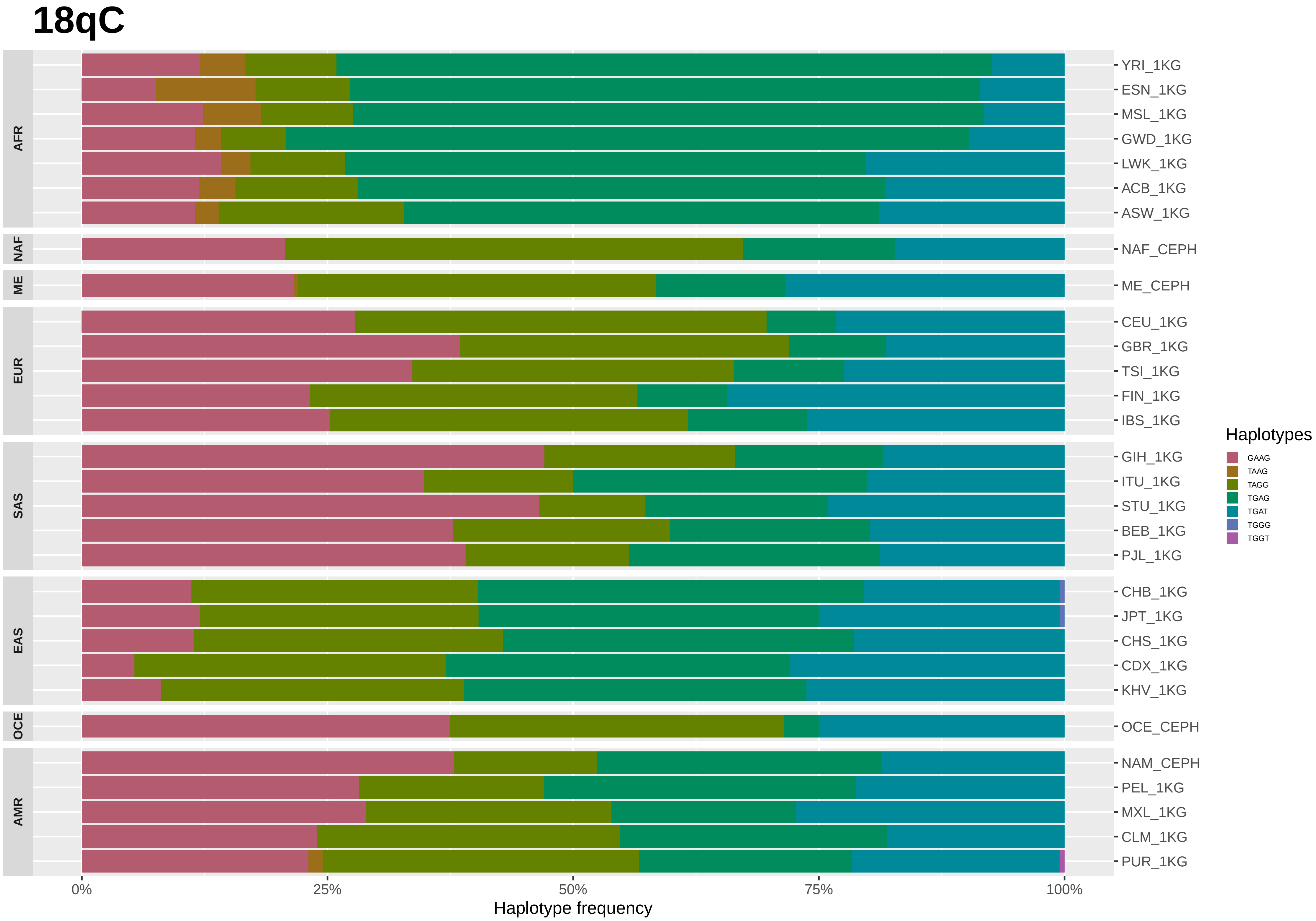
18qC
AFR
NAF
ME
EUR
SAS
EAS
OCE
AMR
YRI_1KG
ESN_1KG
MSL_1KG
GWD_1KG
LWK_1KG
ACB_1KG
ASW_1KG
NAF_CEPH
ME_CEPH
CEU_1KG
GBR_1KG
TSI_1KG
FIN_1KG
IBS_1KG
GIH_1KG
ITU_1KG
STU_1KG
BEB_1KG
PJL_1KG
CHB_1KG
JPT_1KG
CHS_1KG
CDX_1KG
KHV_1KG
OCE_CEPH
NAM_CEPH
PEL_1KG
MXL_1KG
CLM_1KG
PUR_1KG
0%
25%
50%
75%
100%
Haplotype frequency
Haplotypes
GAAG
TAAG
TAGG
TGAG
TGAT
TGGG
TGGT

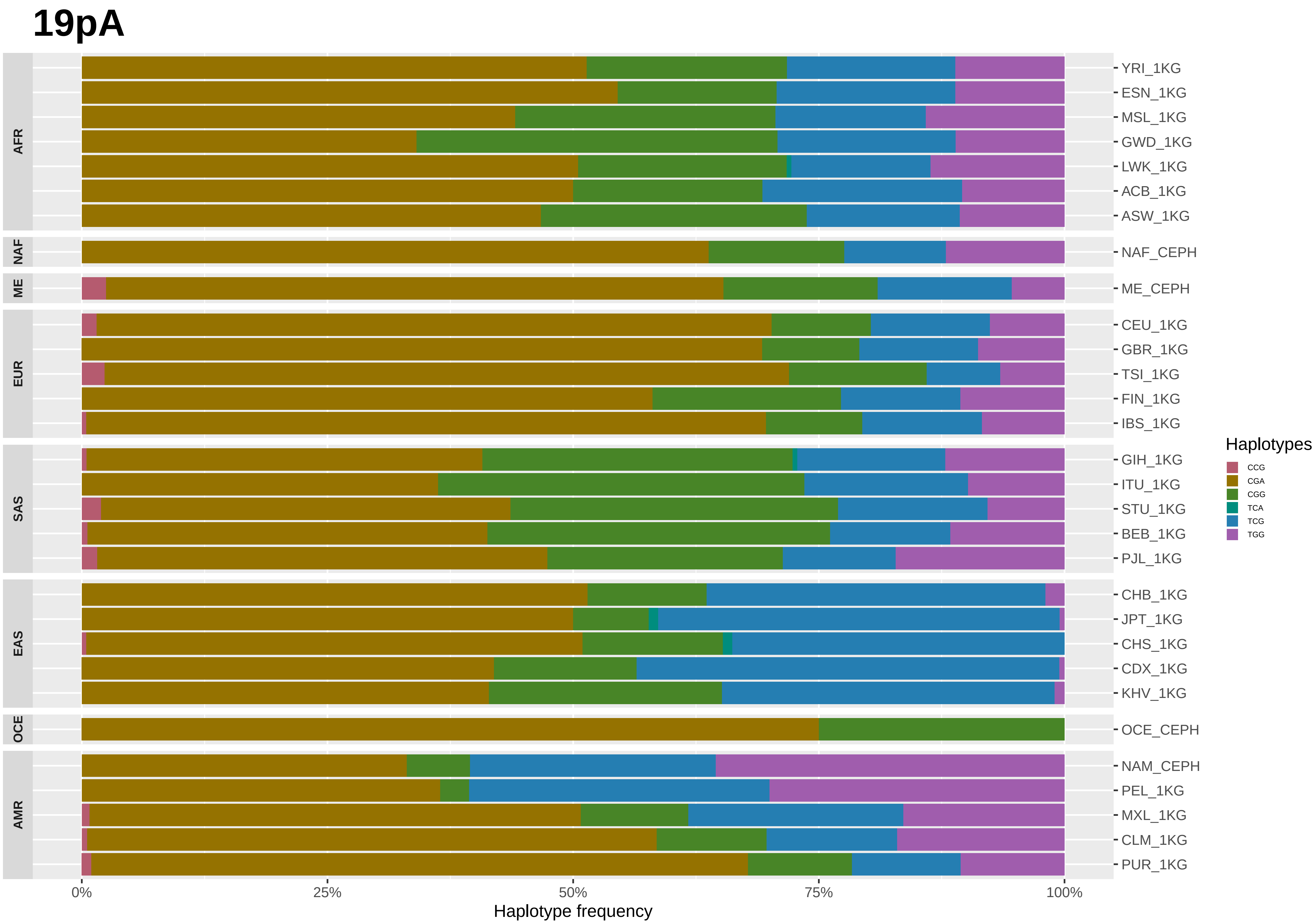

19pA
AFR
NAF
ME
EUR
SAS
EAS
OCE
AMR
YRI_1KG
ESN_1KG
MSL_1KG
GWD_1KG
LWK_1KG
ACB_1KG
ASW_1KG
NAF_CEPH
ME_CEPH
CEU_1KG
GBR_1KG
TSI_1KG
FIN_1KG
IBS_1KG
GIH_1KG
ITU_1KG
STU_1KG
BEB_1KG
PJL_1KG
CHB_1KG
JPT_1KG
CHS_1KG
CDX_1KG
KHV_1KG
OCE_CEPH
NAM_CEPH
PEL_1KG
MXL_1KG
CLM_1KG
PUR_1KG
0%
25%
50%
75%
100%
Haplotype frequency
Haplotypes
CCG
CGA
CGG
TCA
TCG
TGG

# 19pB

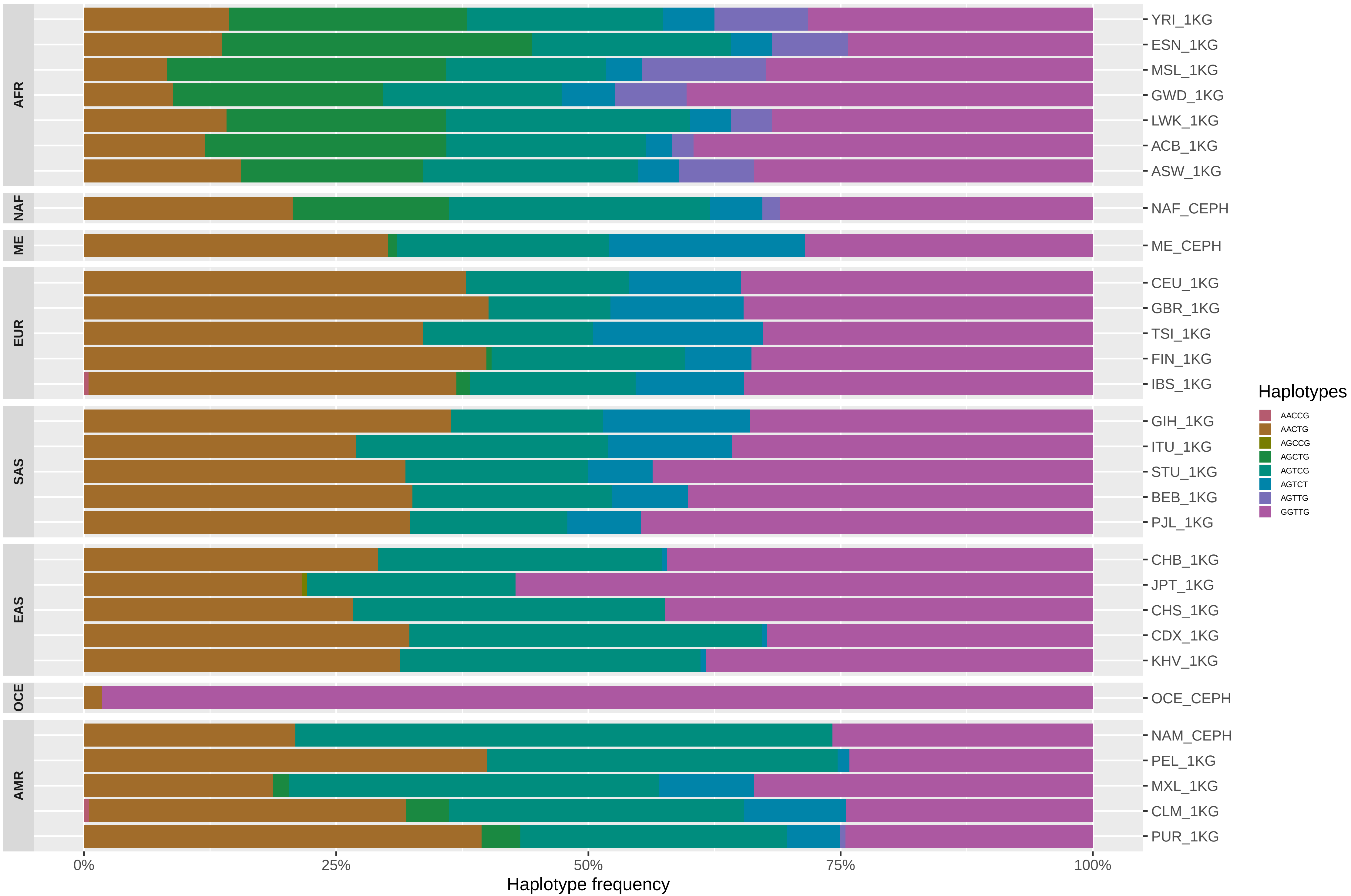

AFR
NAF
ME
EUR
SAS
EAS
OCE
AMR
YRI_1KG
ESN_1KG
MSL_1KG
GWD_1KG
LWK_1KG
ACB_1KG
ASW_1KG
NAF_CEPH
ME_CEPH
CEU_1KG
GBR_1KG
TSI_1KG
FIN_1KG
IBS_1KG
GIH_1KG
ITU_1KG
STU_1KG
BEB_1KG
PJL_1KG
CHB_1KG
JPT_1KG
CHS_1KG
CDX_1KG
KHV_1KG
OCE_CEPH
NAM_CEPH
PEL_1KG
MXL_1KG
CLM_1KG
PUR_1KG
0%
25%
50%
75%
100%
Haplotype frequency
Haplotypes
AACCG
AACTG
AGCCG
AGCTG
AGTCG
AGTCT
AGTTG
GGTTG

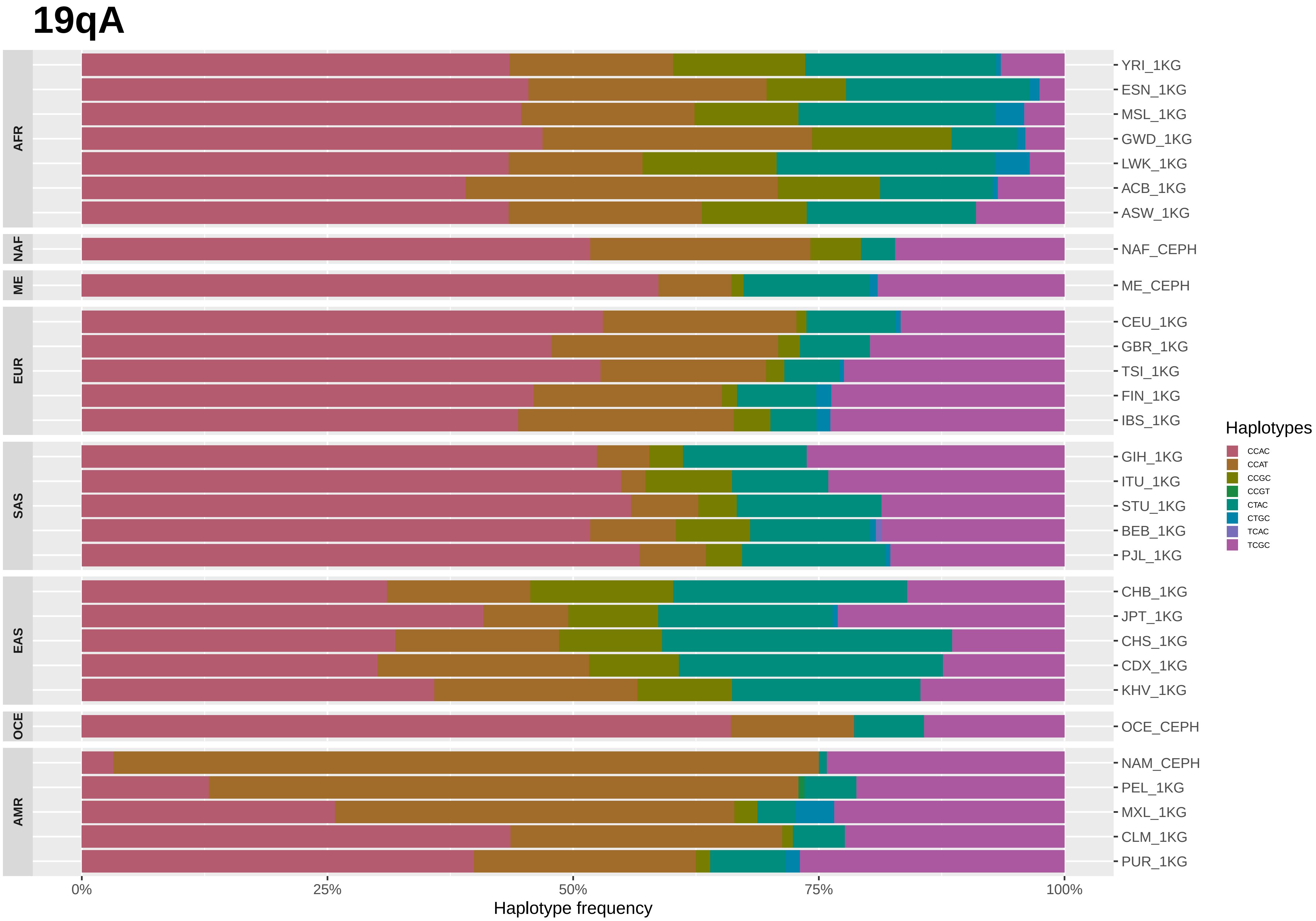

19qA
AFR
NAF
ME
EUR
SAS
EAS
OCE
AMR
YRI_1KG
ESN_1KG
MSL_1KG
GWD_1KG
LWK_1KG
ACB_1KG
ASW_1KG
NAF_CEPH
ME_CEPH
CEU_1KG
GBR_1KG
TSI_1KG
FIN_1KG
IBS_1KG
GIH_1KG
ITU_1KG
STU_1KG
BEB_1KG
PJL_1KG
CHB_1KG
JPT_1KG
CHS_1KG
CDX_1KG
KHV_1KG
OCE_CEPH
NAM_CEPH
PEL_1KG
MXL_1KG
CLM_1KG
PUR_1KG
0%
25%
50%
75%
100%
Haplotype frequency
Haplotypes
CCAC
CCAT
CCGC
CCGT
CTAC
CTGC
TCAC
TCGC

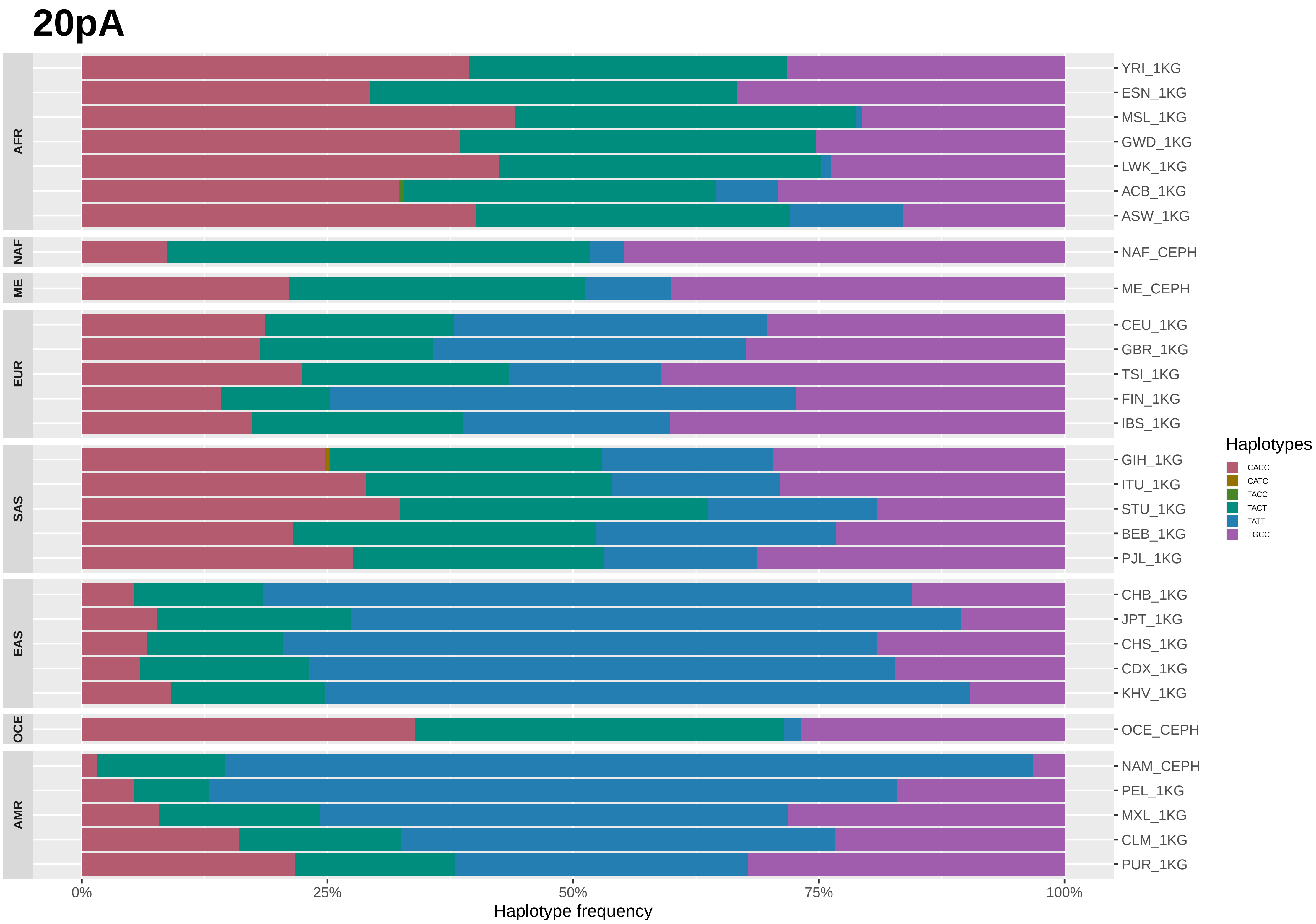

20pA
AFR
NAF
ME
EUR
SAS
EAS
OCE
AMR
YRI_1KG
ESN_1KG
MSL_1KG
GWD_1KG
LWK_1KG
ACB_1KG
ASW_1KG
NAF_CEPH
ME_CEPH
CEU_1KG
GBR_1KG
TSI_1KG
FIN_1KG
IBS_1KG
GIH_1KG
ITU_1KG
STU_1KG
BEB_1KG
PJL_1KG
CHB_1KG
JPT_1KG
CHS_1KG
CDX_1KG
KHV_1KG
OCE_CEPH
NAM_CEPH
PEL_1KG
MXL_1KG
CLM_1KG
PUR_1KG
0%
25%
50%
75%
100%
Haplotype frequency
Haplotypes
CACC
CATC
TACC
TACT
TATT
TGCC

# 20pB

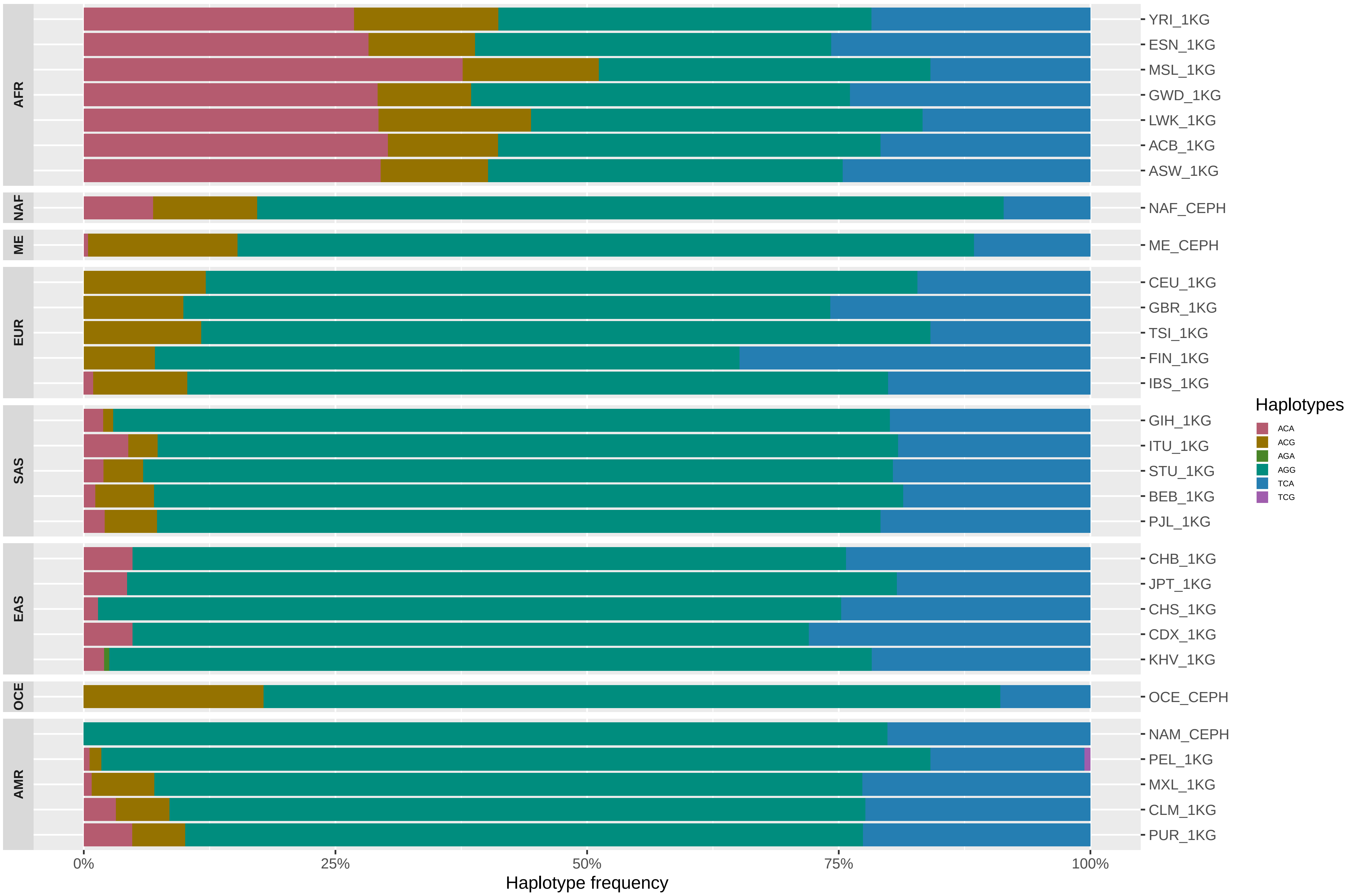
AFR
NAF
ME
EUR
SAS
EAS
OCE
AMR
YRI_1KG
ESN_1KG
MSL_1KG
GWD_1KG
LWK_1KG
ACB_1KG
ASW_1KG
NAF_CEPH
ME_CEPH
CEU_1KG
GBR_1KG
TSI_1KG
FIN_1KG
IBS_1KG
GIH_1KG
ITU_1KG
STU_1KG
BEB_1KG
PJL_1KG
CHB_1KG
JPT_1KG
CHS_1KG
CDX_1KG
KHV_1KG
OCE_CEPH
NAM_CEPH
PEL_1KG
MXL_1KG
CLM_1KG
PUR_1KG
0%
25%
50%
75%
100%
Haplotype frequency
Haplotypes
ACA
ACG
AGA
AGG
TCA
TCG

# 20qA

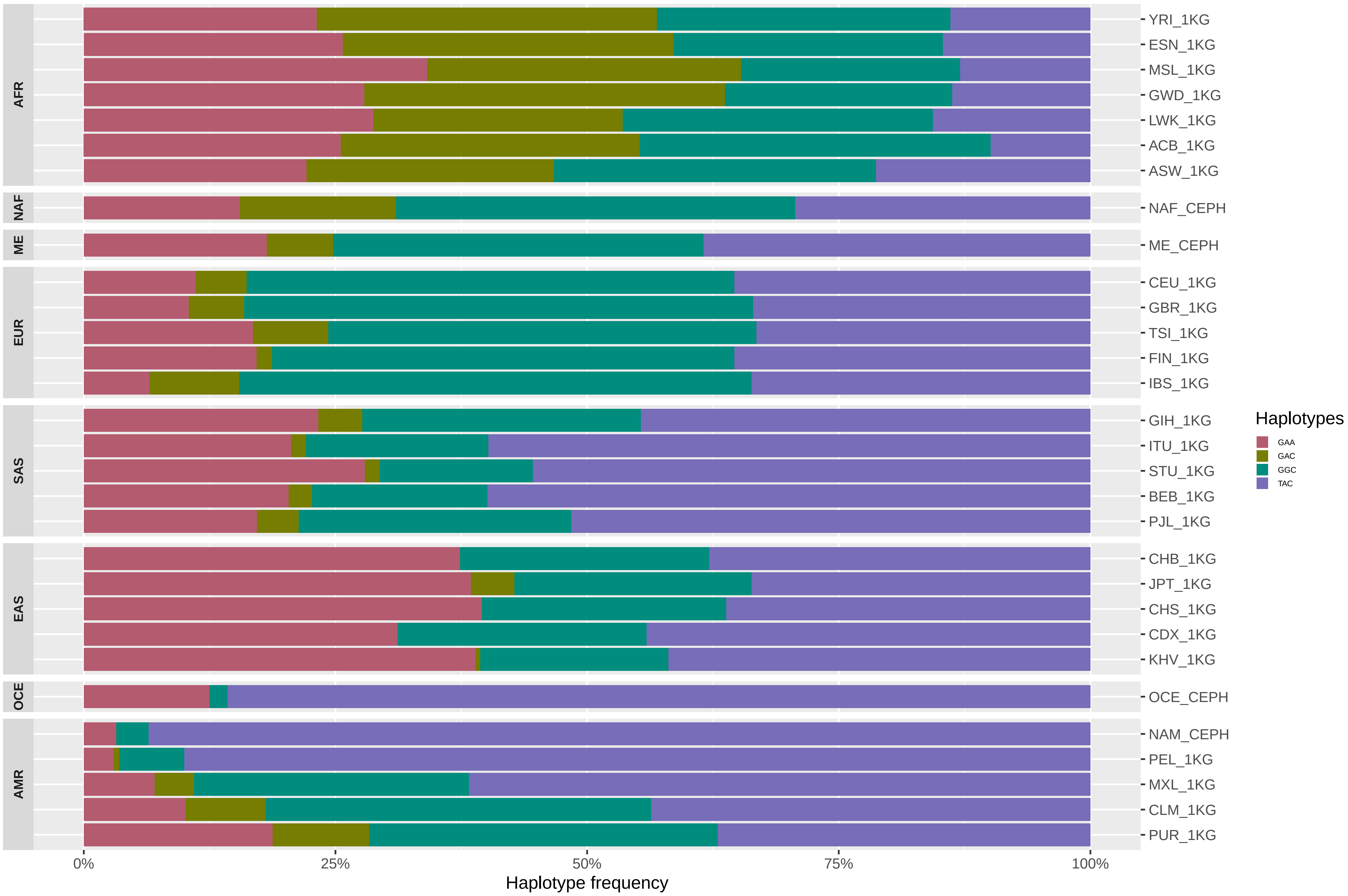

AFR
NAF
ME
EUR
SAS
EAS
OCE
AMR
YRI_1KG
ESN_1KG
MSL_1KG
GWD_1KG
LWK_1KG
ACB_1KG
ASW_1KG
NAF_CEPH
ME_CEPH
CEU_1KG
GBR_1KG
TSI_1KG
FIN_1KG
IBS_1KG
GIH_1KG
ITU_1KG
STU_1KG
BEB_1KG
PJL_1KG
CHB_1KG
JPT_1KG
CHS_1KG
CDX_1KG
KHV_1KG
OCE_CEPH
NAM_CEPH
PEL_1KG
MXL_1KG
CLM_1KG
PUR_1KG
0%
25%
50%
75%
100%
Haplotype frequency
Haplotypes
GAA
GAC
GGC
TAC

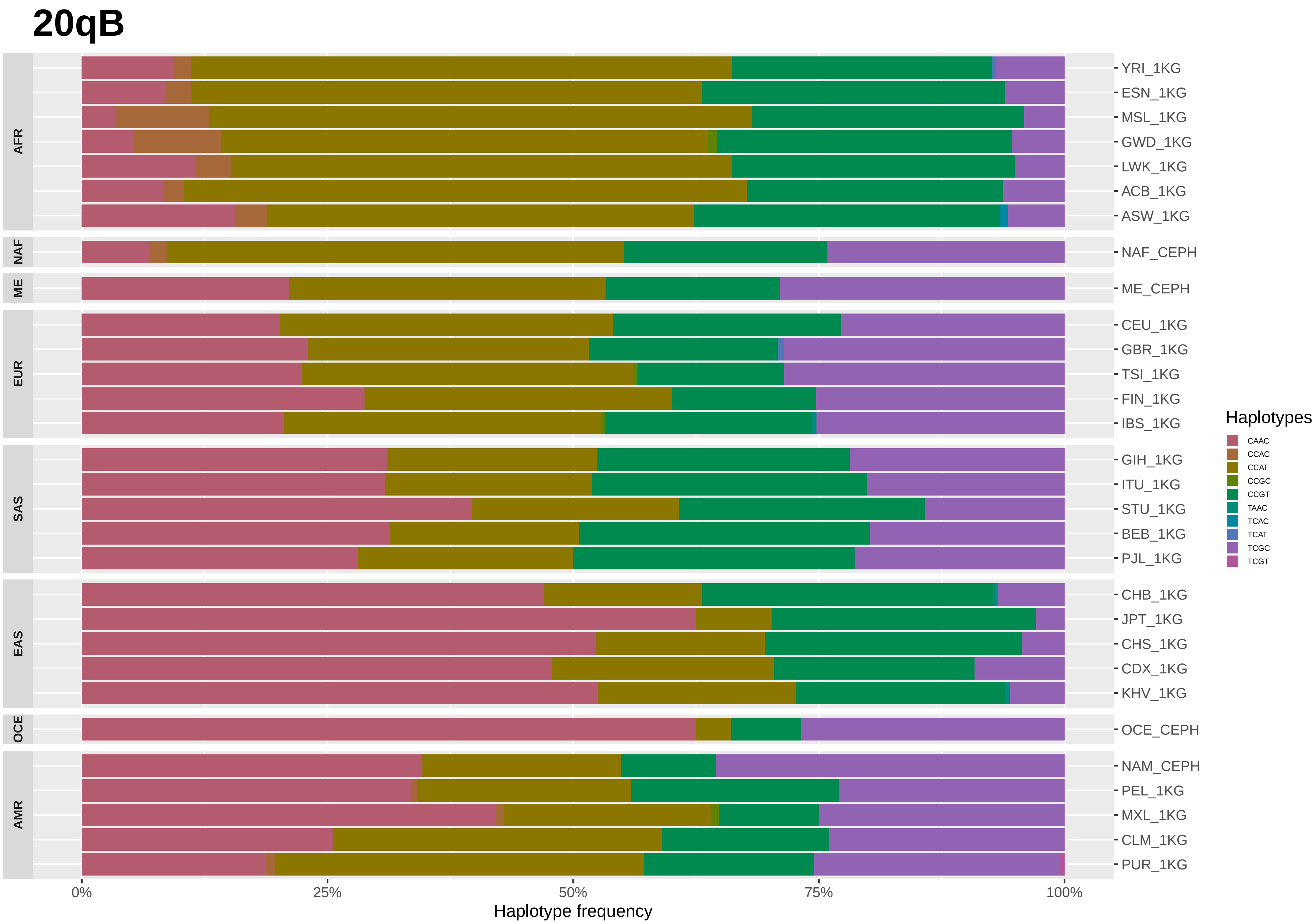

20qB
AFR
NAF
ME
EUR
SAS
EAS
OCE
AMR
YRI_1KG
ESN_1KG
MSL_1KG
GWD_1KG
LWK_1KG
ACB_1KG
ASW_1KG
NAF_CEPH
ME_CEPH
CEU_1KG
GBR_1KG
TSI_1KG
FIN_1KG
IBS_1KG
GIH_1KG
ITU_1KG
STU_1KG
BEB_1KG
PJL_1KG
CHB_1KG
JPT_1KG
CHS_1KG
CDX_1KG
KHV_1KG
OCE_CEPH
NAM_CEPH
PEL_1KG
MXL_1KG
CLM_1KG
PUR_1KG
0%
25%
50%
75%
100%
Haplotype frequency
Haplotypes
CAAC
CCAC
CCAT
CCGC
CCGT
TAAC
TCAC
TCAT
TCGC
TCGT

# 21qA

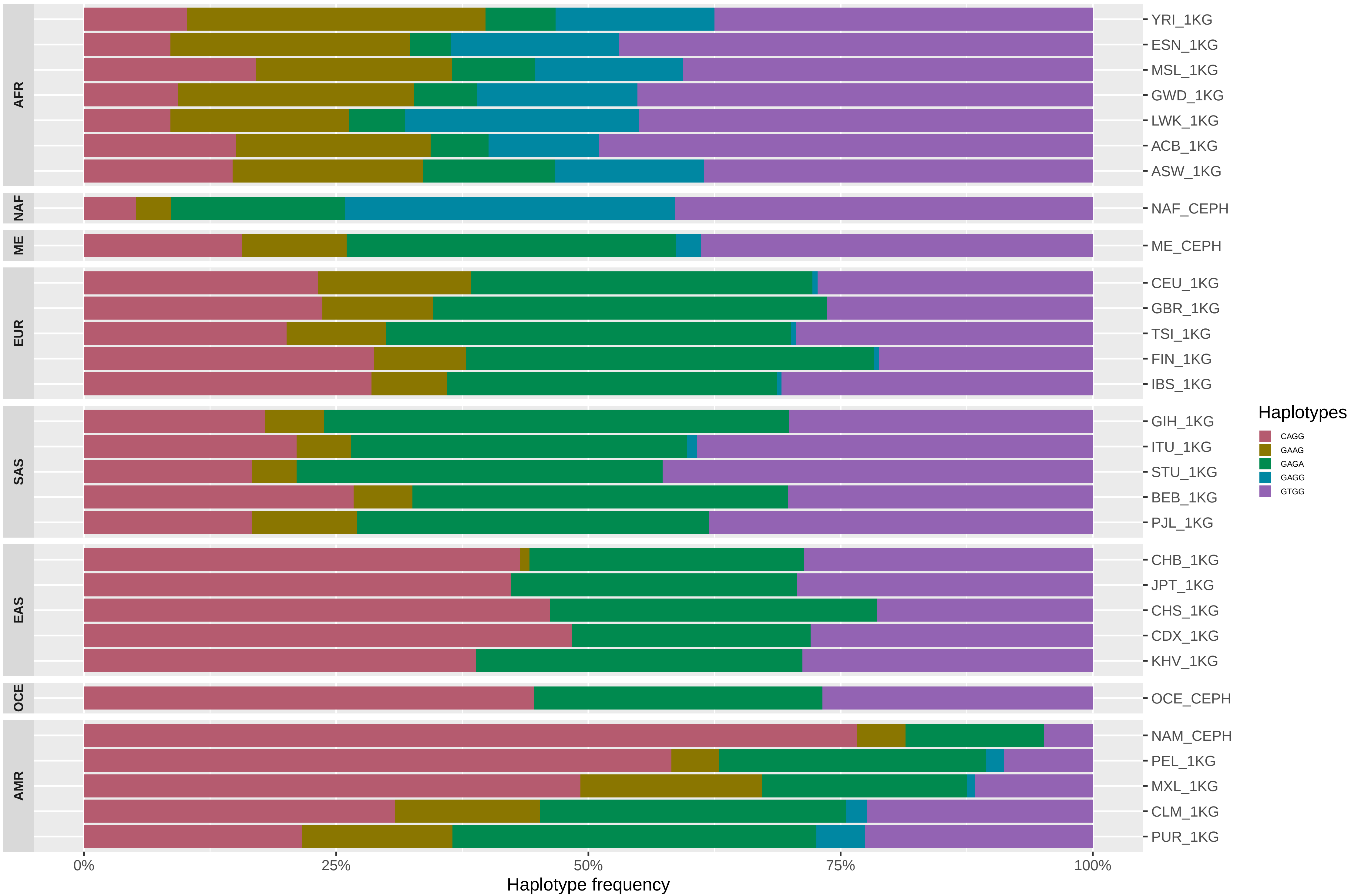

AFR
NAF
ME
EUR
SAS
EAS
OCE
AMR
YRI_1KG
ESN_1KG
MSL_1KG
GWD_1KG
LWK_1KG
ACB_1KG
ASW_1KG
NAF_CEPH
ME_CEPH
CEU_1KG
GBR_1KG
TSI_1KG
FIN_1KG
IBS_1KG
GIH_1KG
ITU_1KG
STU_1KG
BEB_1KG
PJL_1KG
CHB_1KG
JPT_1KG
CHS_1KG
CDX_1KG
KHV_1KG
OCE_CEPH
NAM_CEPH
PEL_1KG
MXL_1KG
CLM_1KG
PUR_1KG
0%
25%
50%
75%
100%
Haplotype frequency
Haplotypes
CAGG
GAAG
GAGA
GAGG
GTGG

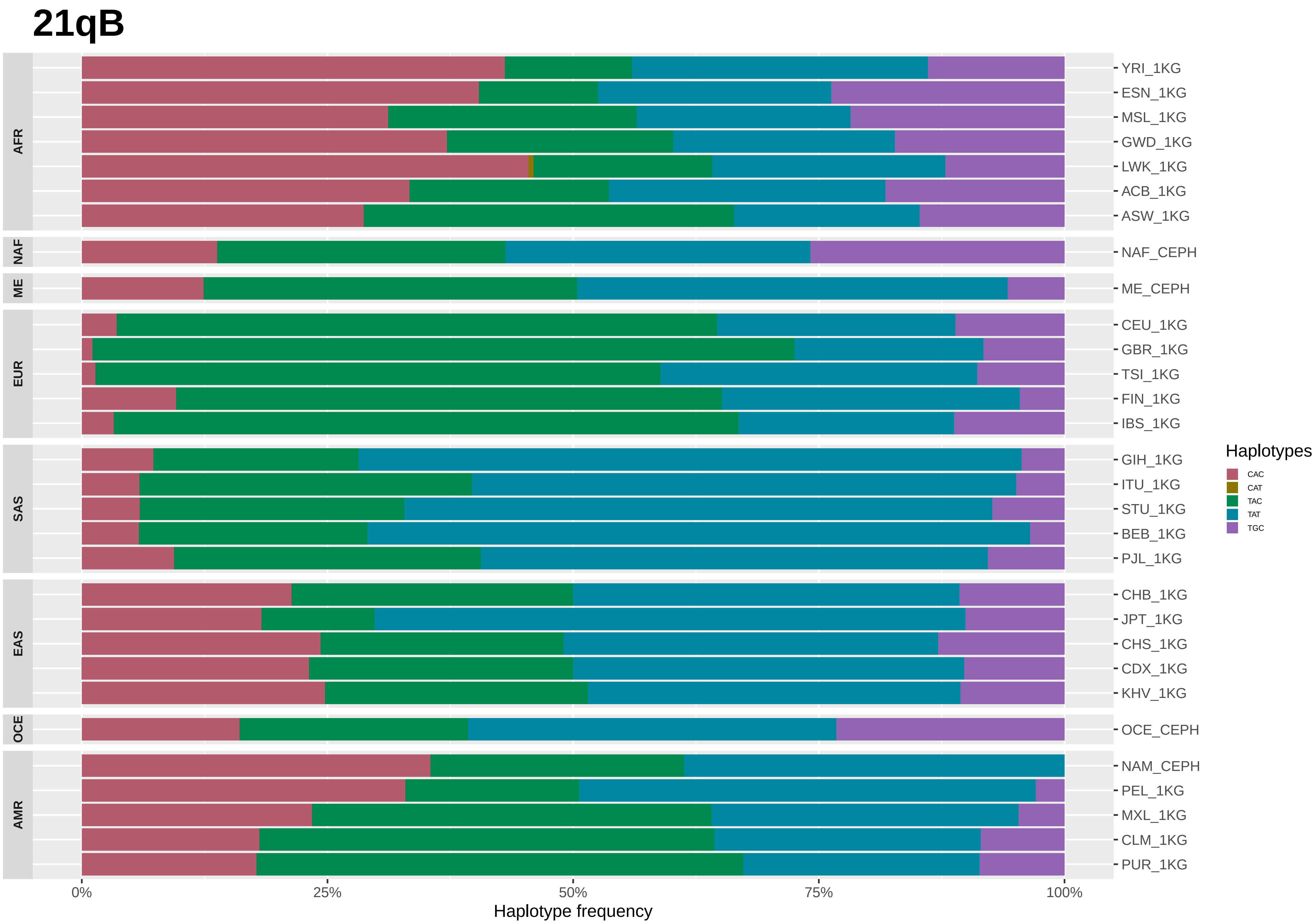

21qB
AFR
NAF
ME
EUR
SAS
EAS
OCE
AMR
YRI_1KG
ESN_1KG
MSL_1KG
GWD_1KG
LWK_1KG
ACB_1KG
ASW_1KG
NAF_CEPH
ME_CEPH
CEU_1KG
GBR_1KG
TSI_1KG
FIN_1KG
IBS_1KG
GIH_1KG
ITU_1KG
STU_1KG
BEB_1KG
PJL_1KG
CHB_1KG
JPT_1KG
CHS_1KG
CDX_1KG
KHV_1KG
OCE_CEPH
NAM_CEPH
PEL_1KG
MXL_1KG
CLM_1KG
PUR_1KG
0%
25%
50%
75%
100%
Haplotype frequency
Haplotypes
CAC
CAT
TAC
TAT
TGC

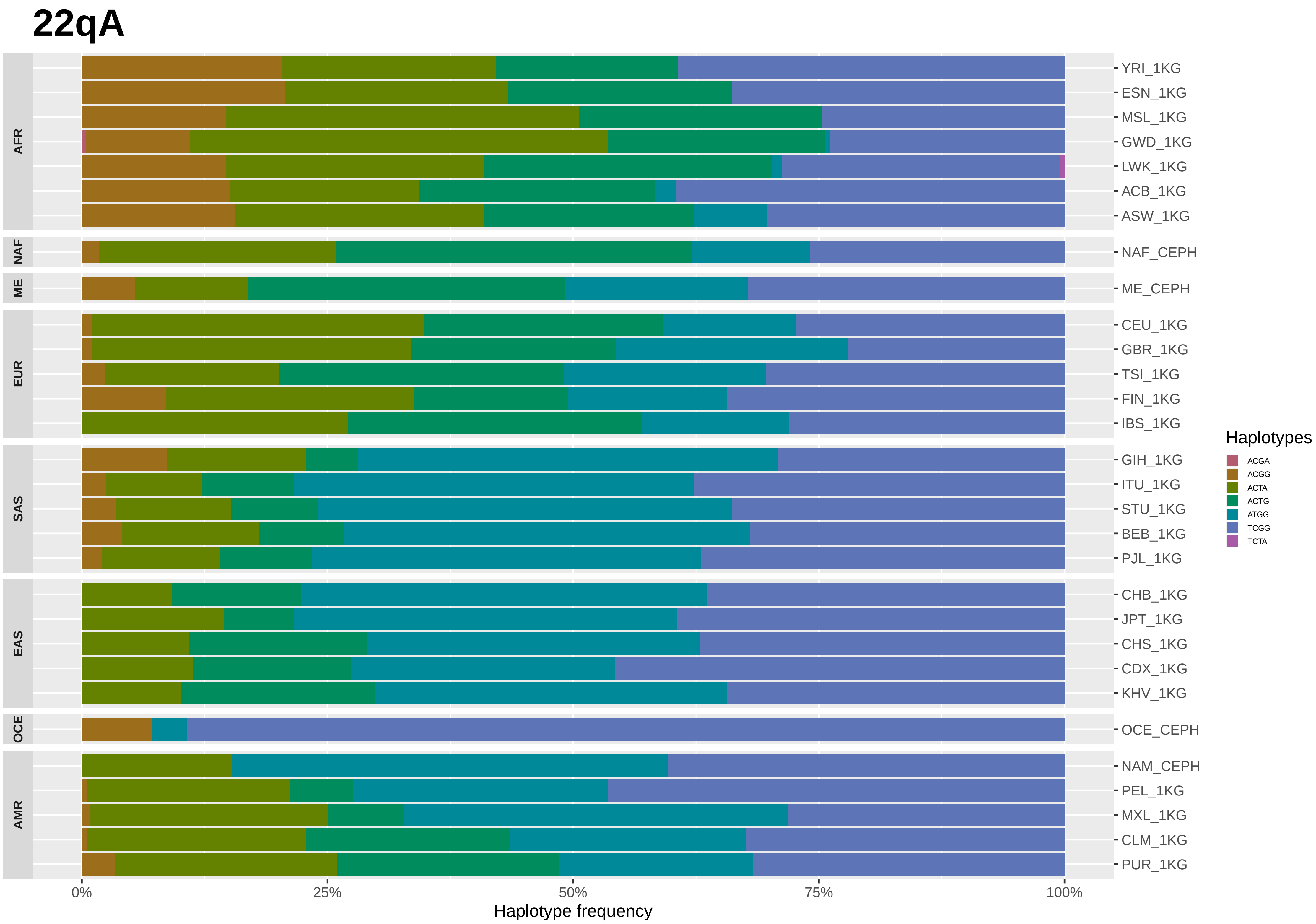
22qA
AFR
NAF
ME
EUR
SAS
EAS
OCE
AMR
YRI_1KG
ESN_1KG
MSL_1KG
GWD_1KG
LWK_1KG
ACB_1KG
ASW_1KG
NAF_CEPH
ME_CEPH
CEU_1KG
GBR_1KG
TSI_1KG
FIN_1KG
IBS_1KG
GIH_1KG
ITU_1KG
STU_1KG
BEB_1KG
PJL_1KG
CHB_1KG
JPT_1KG
CHS_1KG
CDX_1KG
KHV_1KG
OCE_CEPH
NAM_CEPH
PEL_1KG
MXL_1KG
CLM_1KG
PUR_1KG
0%
25%
50%
75%
100%
Haplotype frequency
Haplotypes
ACGA
ACGG
ACTA
ACTG
ATGG
TCGG
TCTA

# 22qB

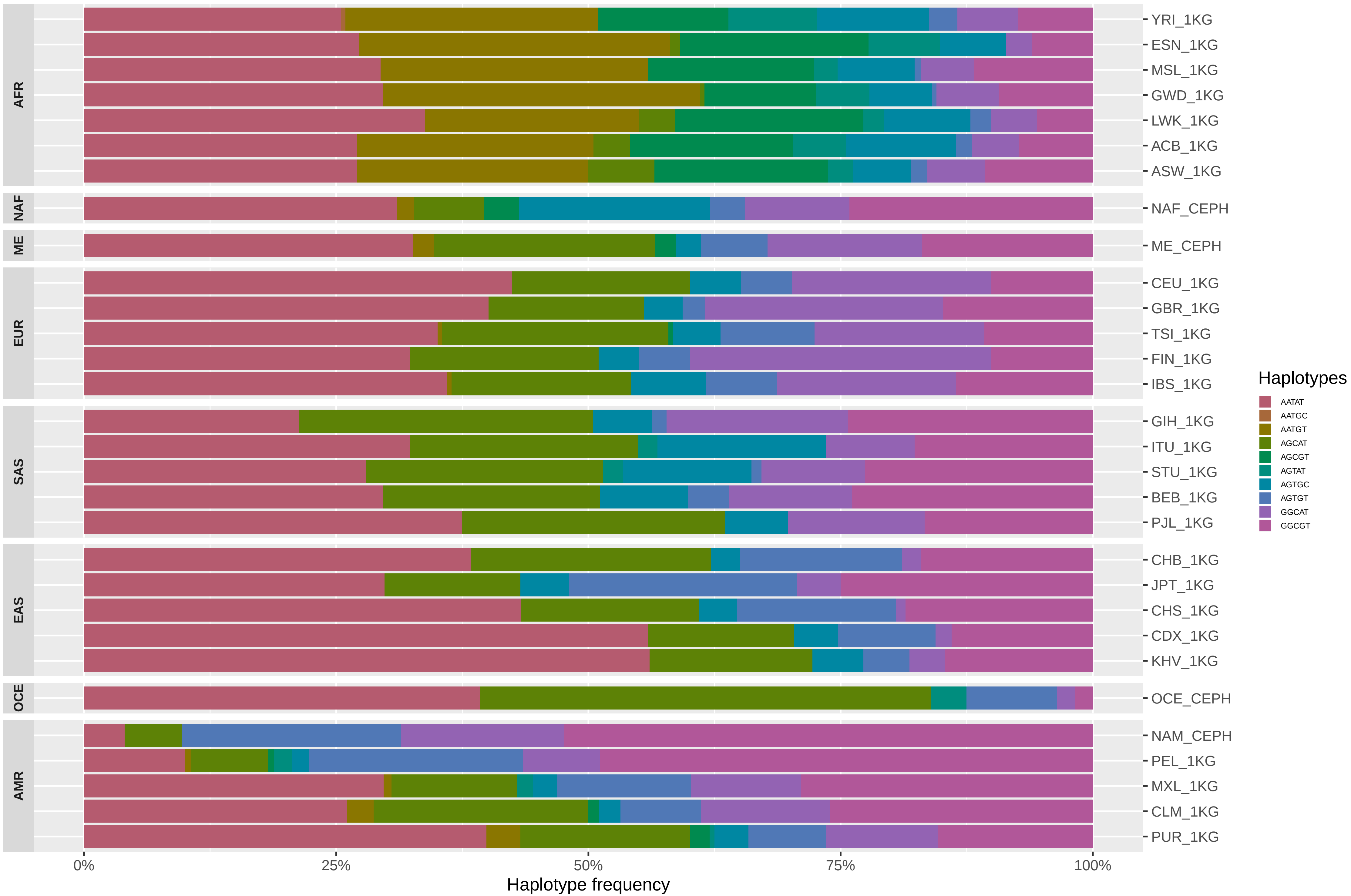
AFR
NAF
ME
EUR
SAS
EAS
OCE
AMR
YRI_1KG
ESN_1KG
MSL_1KG
GWD_1KG
LWK_1KG
ACB_1KG
ASW_1KG
NAF_CEPH
ME_CEPH
CEU_1KG
GBR_1KG
TSI_1KG
FIN_1KG
IBS_1KG
GIH_1KG
ITU_1KG
STU_1KG
BEB_1KG
PJL_1KG
CHB_1KG
JPT_1KG
CHS_1KG
CDX_1KG
KHV_1KG
OCE_CEPH
NAM_CEPH
PEL_1KG
MXL_1KG
CLM_1KG
PUR_1KG
0%
25%
50%
75%
100%
Haplotype frequency
Haplotypes
AATAT
AATGC
AATGT
AGCAT
AGCGT
AGTAT
AGTGC
AGTGT
GGCAT
GGCGT

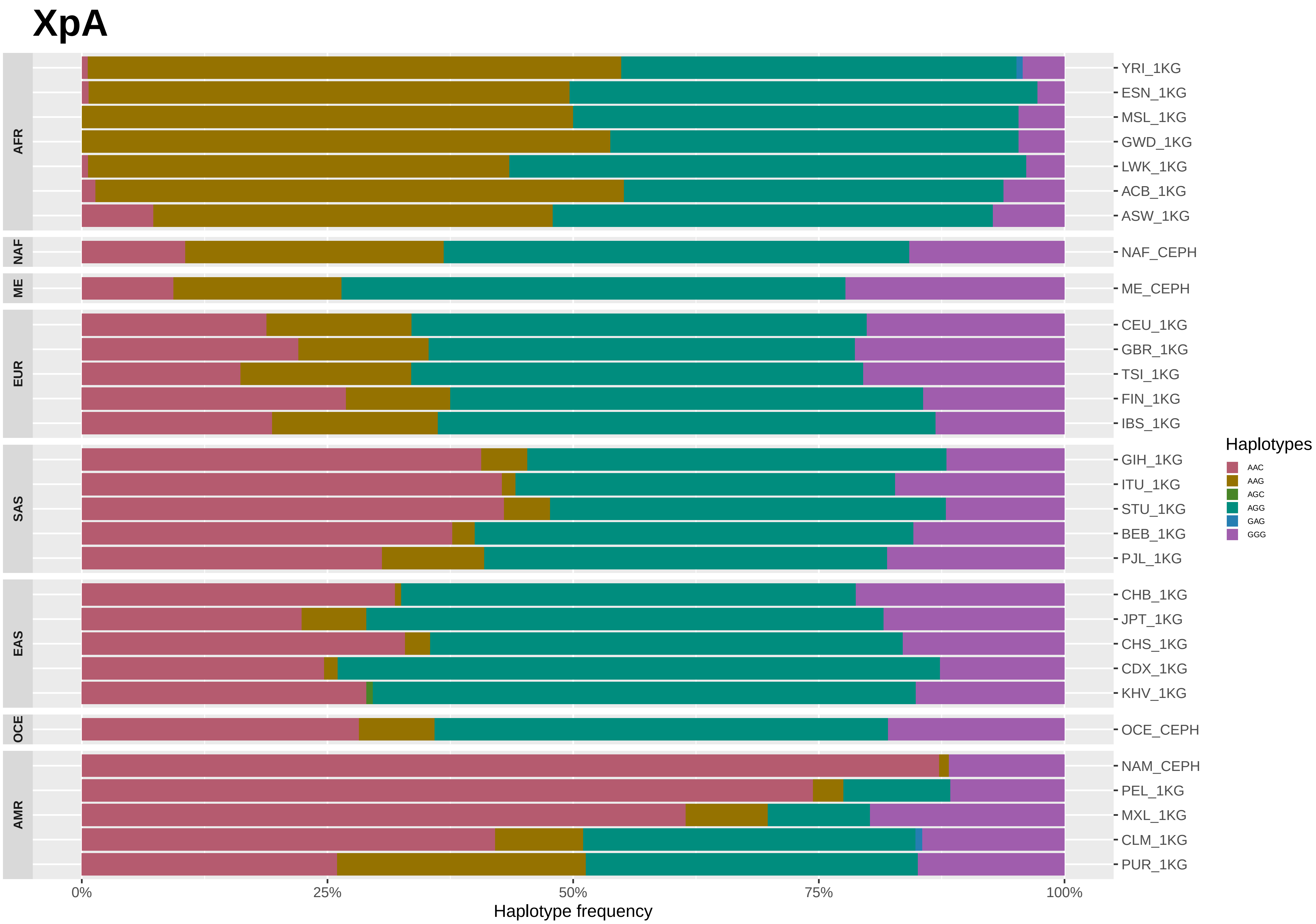
XpA
AFR
NAF
ME
EUR
SAS
EAS
OCE
AMR
YRI_1KG
ESN_1KG
MSL_1KG
GWD_1KG
LWK_1KG
ACB_1KG
ASW_1KG
NAF_CEPH
ME_CEPH
CEU_1KG
GBR_1KG
TSI_1KG
FIN_1KG
IBS_1KG
GIH_1KG
ITU_1KG
STU_1KG
BEB_1KG
PJL_1KG
CHB_1KG
JPT_1KG
CHS_1KG
CDX_1KG
KHV_1KG
OCE_CEPH
NAM_CEPH
PEL_1KG
MXL_1KG
CLM_1KG
PUR_1KG
0%
25%
50%
75%
100%
Haplotype frequency
Haplotypes
AAC
AAG
AGC
AGG
GAG
GGG

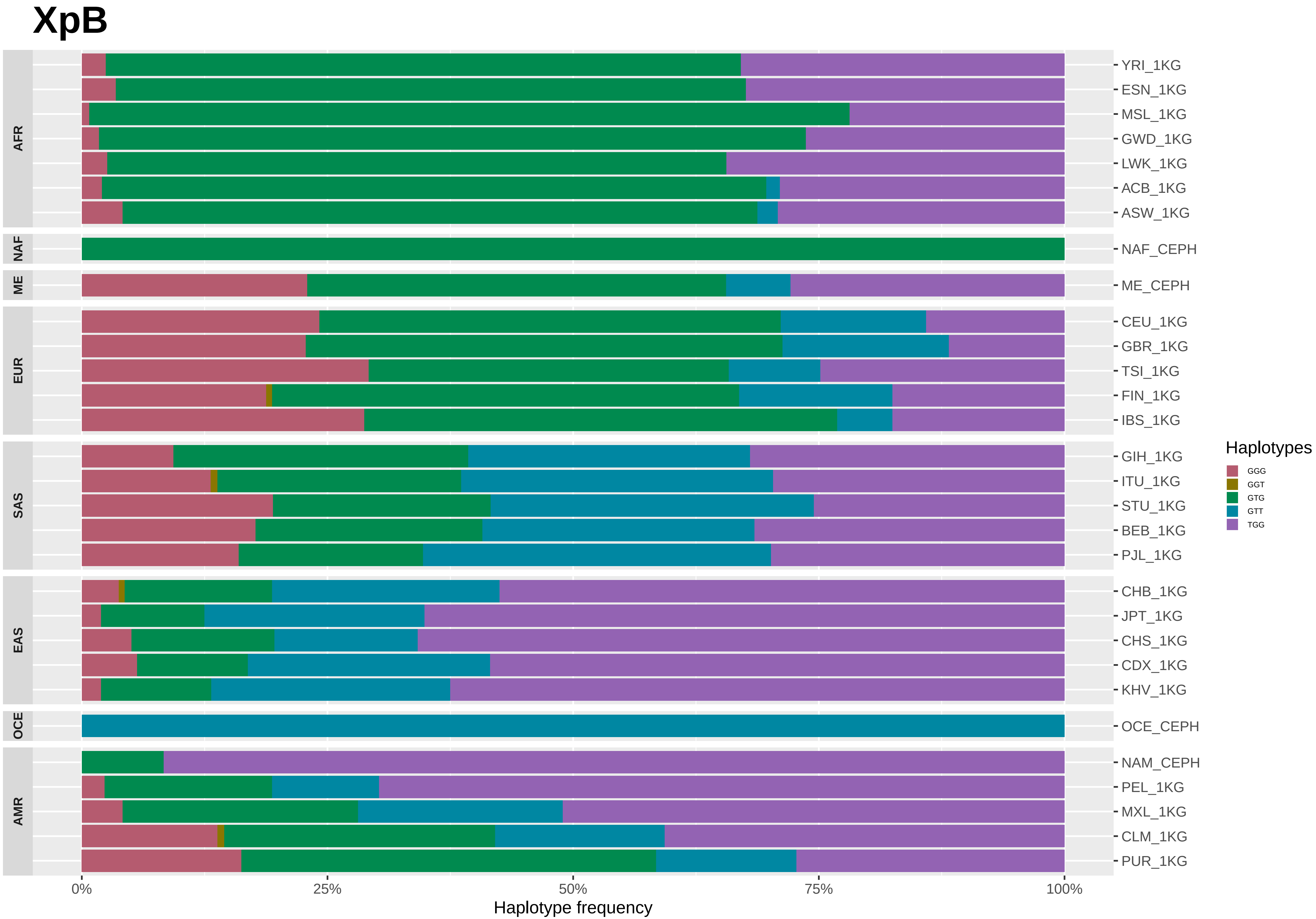
XpB
AFR
NAF
ME
EUR
SAS
EAS
OCE
AMR
YRI_1KG
ESN_1KG
MSL_1KG
GWD_1KG
LWK_1KG
ACB_1KG
ASW_1KG
NAF_CEPH
ME_CEPH
CEU_1KG
GBR_1KG
TSI_1KG
FIN_1KG
IBS_1KG
GIH_1KG
ITU_1KG
STU_1KG
BEB_1KG
PJL_1KG
CHB_1KG
JPT_1KG
CHS_1KG
CDX_1KG
KHV_1KG
OCE_CEPH
NAM_CEPH
PEL_1KG
MXL_1KG
CLM_1KG
PUR_1KG
0%
25%
50%
75%
100%
Haplotype frequency
Haplotypes
GGG
GGT
GTG
GTT
TGG

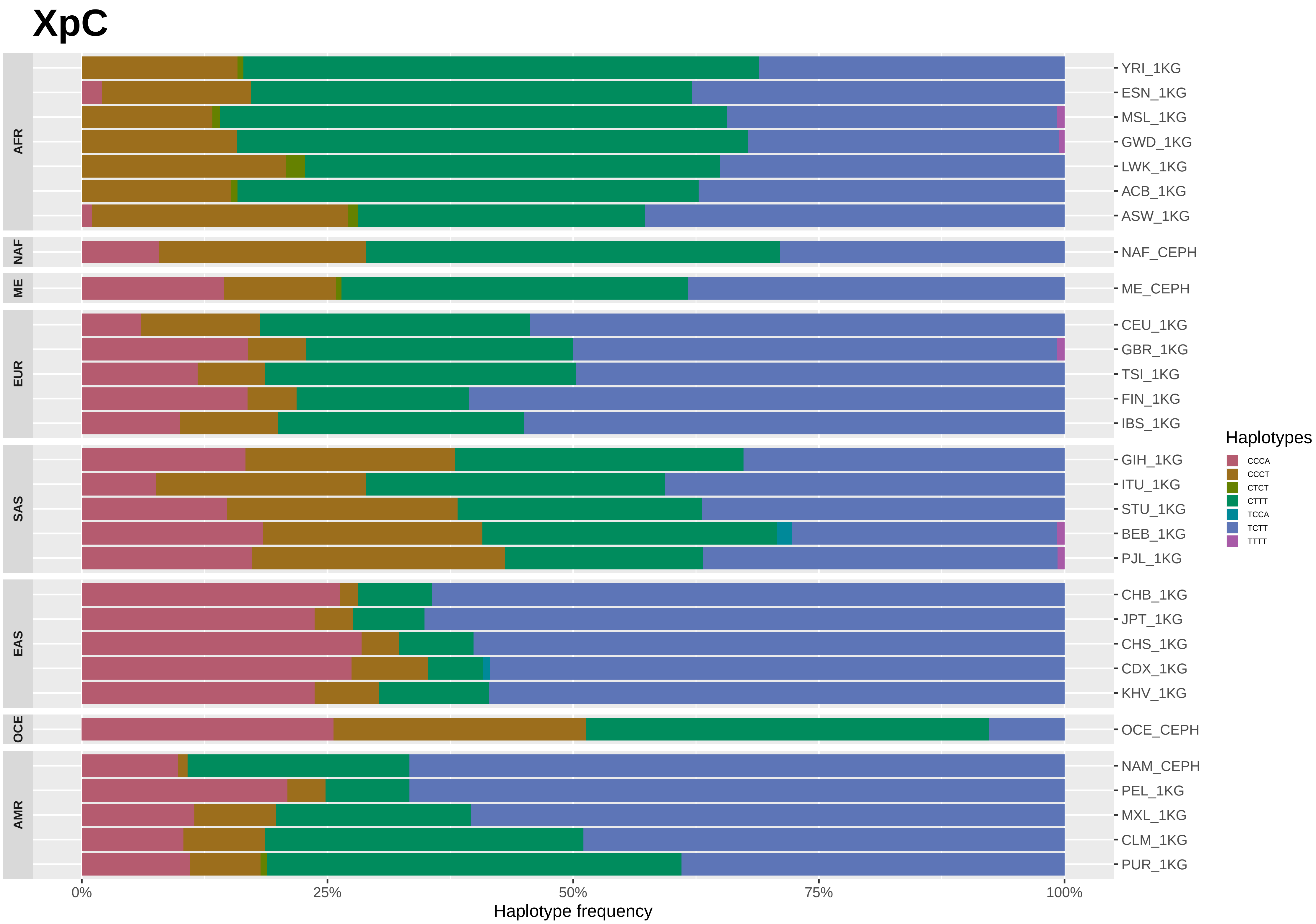
XpC
AFR
NAF
ME
EUR
SAS
EAS
OCE
AMR
YRI_1KG
ESN_1KG
MSL_1KG
GWD_1KG
LWK_1KG
ACB_1KG
ASW_1KG
NAF_CEPH
ME_CEPH
CEU_1KG
GBR_1KG
TSI_1KG
FIN_1KG
IBS_1KG
GIH_1KG
ITU_1KG
STU_1KG
BEB_1KG
PJL_1KG
CHB_1KG
JPT_1KG
CHS_1KG
CDX_1KG
KHV_1KG
OCE_CEPH
NAM_CEPH
PEL_1KG
MXL_1KG
CLM_1KG
PUR_1KG
0%
25%
50%
75%
100%
Haplotype frequency
Haplotypes
CCCA
CCCT
CTCT
CTTT
TCCA
TCTT
TTTT

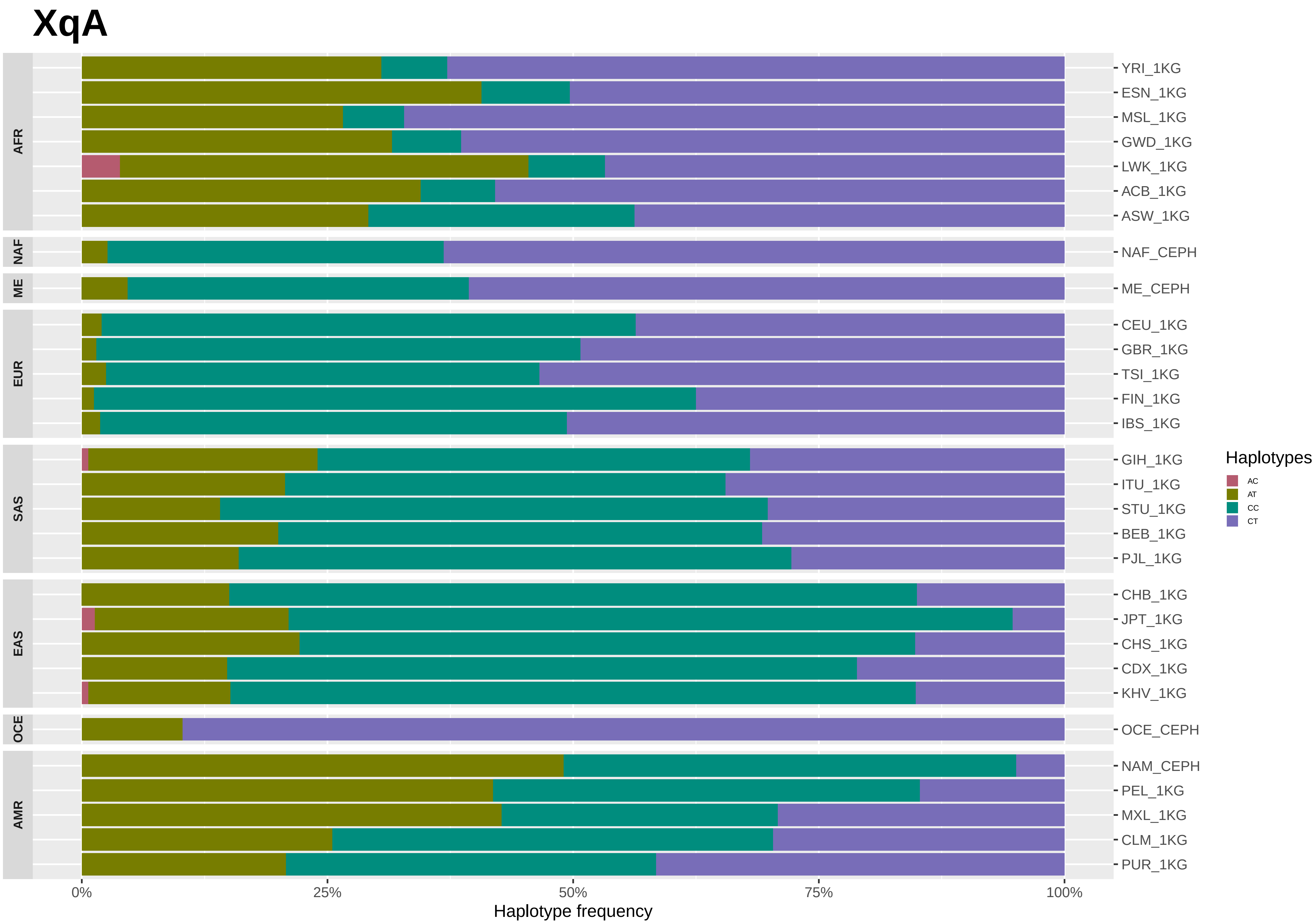

XqA
AFR
NAF
ME
EUR
SAS
EAS
OCE
AMR
YRI_1KG
ESN_1KG
MSL_1KG
GWD_1KG
LWK_1KG
ACB_1KG
ASW_1KG
NAF_CEPH
ME_CEPH
CEU_1KG
GBR_1KG
TSI_1KG
FIN_1KG
IBS_1KG
GIH_1KG
ITU_1KG
STU_1KG
BEB_1KG
PJL_1KG
CHB_1KG
JPT_1KG
CHS_1KG
CDX_1KG
KHV_1KG
OCE_CEPH
NAM_CEPH
PEL_1KG
MXL_1KG
CLM_1KG
PUR_1KG
Haplotypes
AC
AT
CC
CT
0%
25%
50%
75%
100%
Haplotype frequency

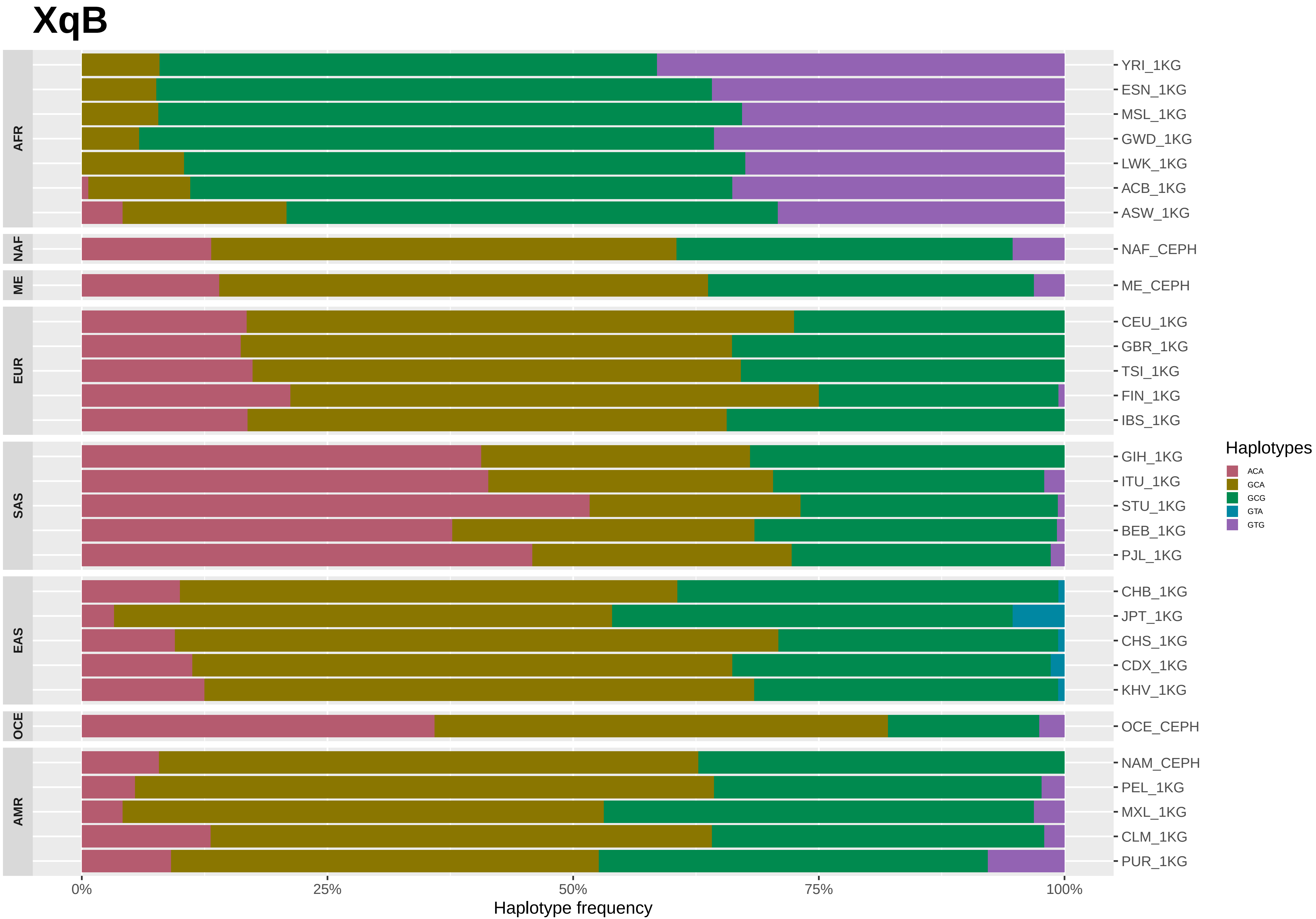

XqB
AFR
NAF
ME
EUR
SAS
EAS
OCE
AMR
YRI_1KG
ESN_1KG
MSL_1KG
GWD_1KG
LWK_1KG
ACB_1KG
ASW_1KG
NAF_CEPH
ME_CEPH
CEU_1KG
GBR_1KG
TSI_1KG
FIN_1KG
IBS_1KG
GIH_1KG
ITU_1KG
STU_1KG
BEB_1KG
PJL_1KG
CHB_1KG
JPT_1KG
CHS_1KG
CDX_1KG
KHV_1KG
OCE_CEPH
NAM_CEPH
PEL_1KG
MXL_1KG
CLM_1KG
PUR_1KG
Haplotypes
ACA
GCA
GCG
GTA
GTG
0%
25%
50%
75%
100%
Haplotype frequency

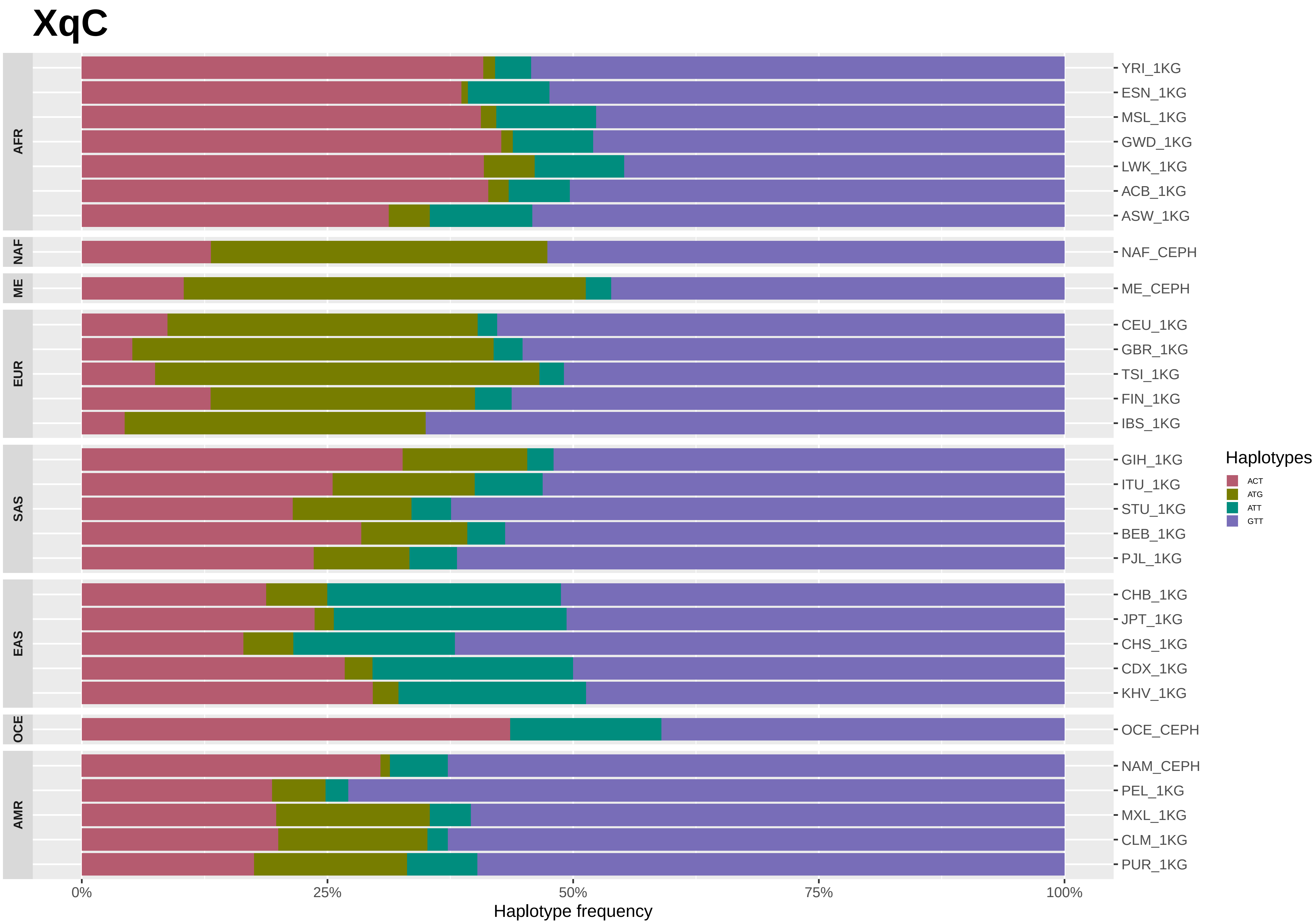
XqC
AFR
NAF
ME
EUR
SAS
EAS
OCE
AMR
YRI_1KG
ESN_1KG
MSL_1KG
GWD_1KG
LWK_1KG
ACB_1KG
ASW_1KG
NAF_CEPH
ME_CEPH
CEU_1KG
GBR_1KG
TSI_1KG
FIN_1KG
IBS_1KG
GIH_1KG
ITU_1KG
STU_1KG
BEB_1KG
PJL_1KG
CHB_1KG
JPT_1KG
CHS_1KG
CDX_1KG
KHV_1KG
OCE_CEPH
NAM_CEPH
PEL_1KG
MXL_1KG
CLM_1KG
PUR_1KG
0%
25%
50%
75%
100%
Haplotype frequency
Haplotypes
ACT
ATG
ATT
GTT

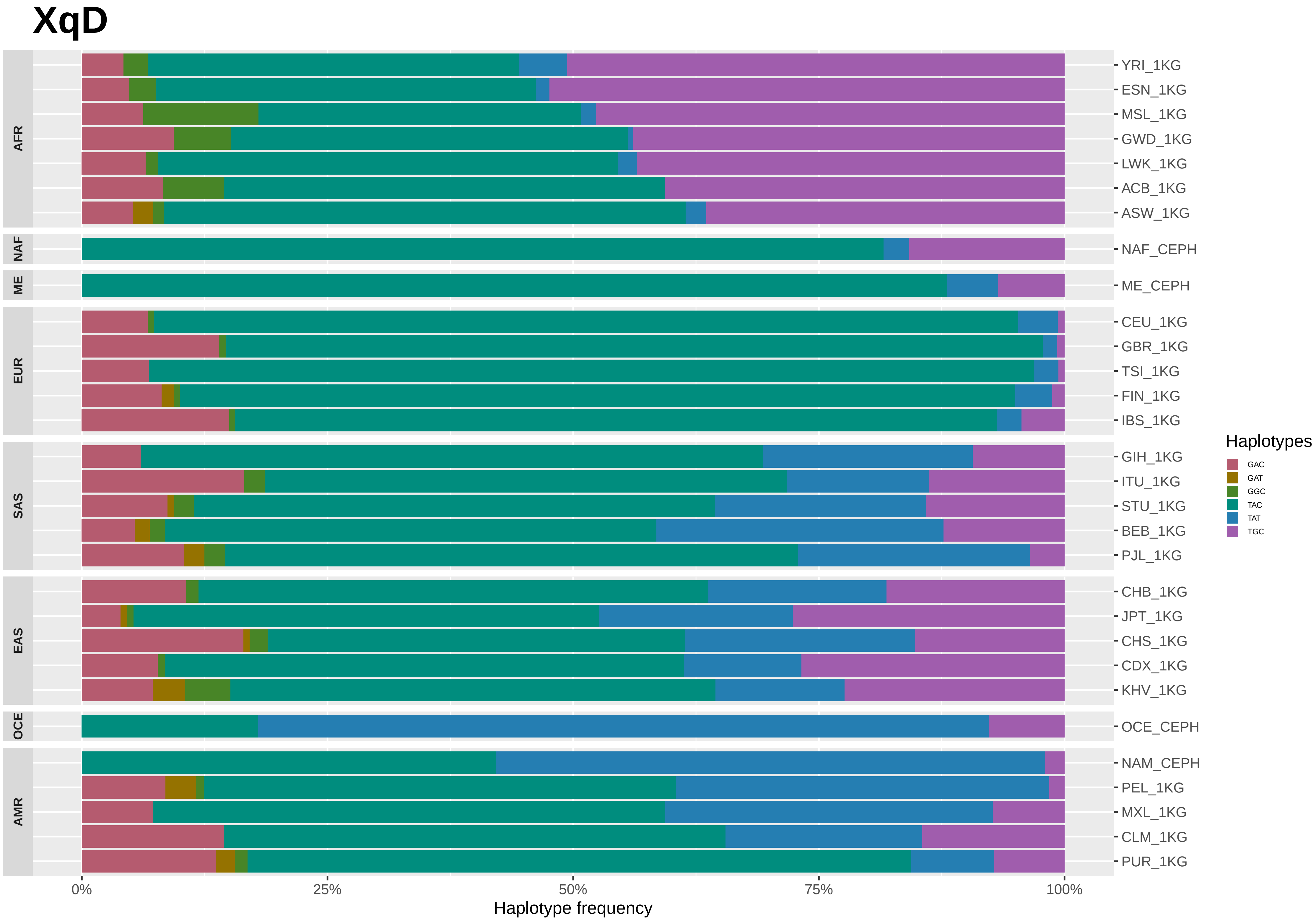

XqD
AFR
NAF
ME
EUR
SAS
EAS
OCE
AMR
YRI_1KG
ESN_1KG
MSL_1KG
GWD_1KG
LWK_1KG
ACB_1KG
ASW_1KG
NAF_CEPH
ME_CEPH
CEU_1KG
GBR_1KG
TSI_1KG
FIN_1KG
IBS_1KG
GIH_1KG
ITU_1KG
STU_1KG
BEB_1KG
PJL_1KG
CHB_1KG
JPT_1KG
CHS_1KG
CDX_1KG
KHV_1KG
OCE_CEPH
NAM_CEPH
PEL_1KG
MXL_1KG
CLM_1KG
PUR_1KG
0%
25%
50%
75%
100%
Haplotype frequency
Haplotypes
GAC
GAT
GGC
TAC
TAT
TGC

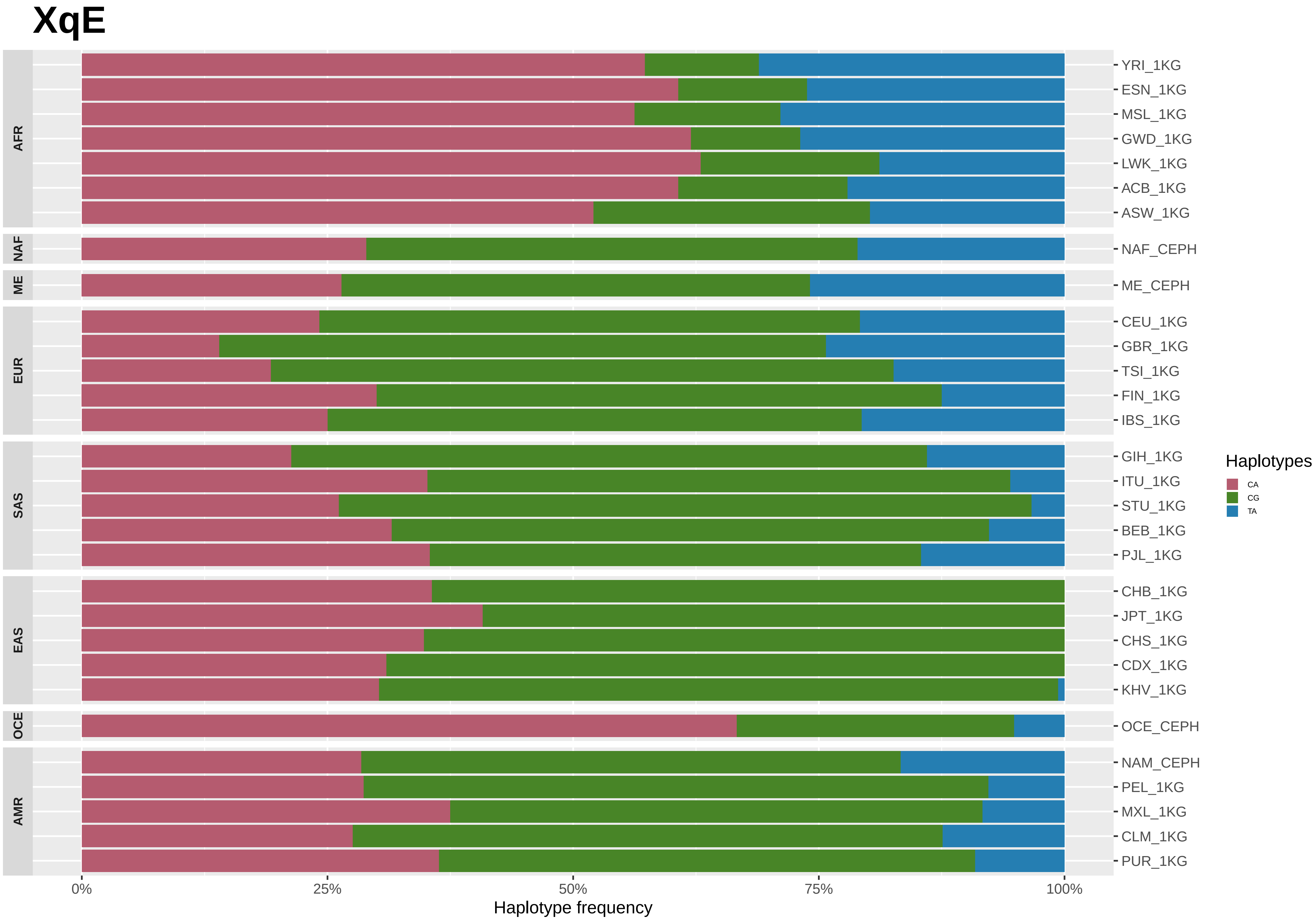

XqE
AFR
NAF
ME
EUR
SAS
EAS
OCE
AMR
YRI_1KG
ESN_1KG
MSL_1KG
GWD_1KG
LWK_1KG
ACB_1KG
ASW_1KG
NAF_CEPH
ME_CEPH
CEU_1KG
GBR_1KG
TSI_1KG
FIN_1KG
IBS_1KG
GIH_1KG
ITU_1KG
STU_1KG
BEB_1KG
PJL_1KG
CHB_1KG
JPT_1KG
CHS_1KG
CDX_1KG
KHV_1KG
OCE_CEPH
NAM_CEPH
PEL_1KG
MXL_1KG
CLM_1KG
PUR_1KG
Haplotypes
CA
CG
TA
0%
25%
50%
75%
100%
Haplotype frequency

# XqF

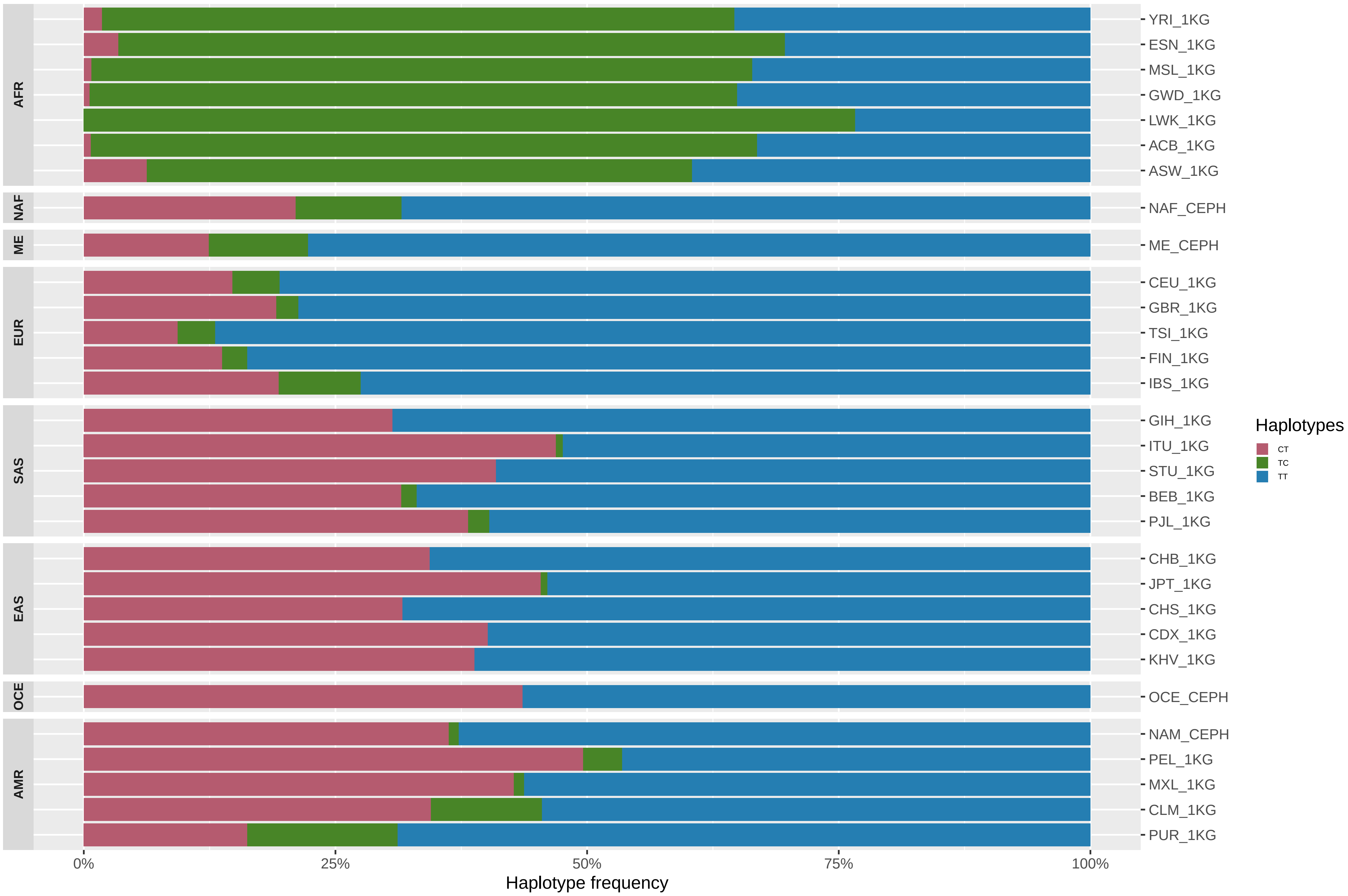

AFR
NAF
ME
EUR
SAS
EAS
OCE
AMR
YRI_1KG
ESN_1KG
MSL_1KG
GWD_1KG
LWK_1KG
ACB_1KG
ASW_1KG
NAF_CEPH
ME_CEPH
CEU_1KG
GBR_1KG
TSI_1KG
FIN_1KG
IBS_1KG
GIH_1KG
ITU_1KG
STU_1KG
BEB_1KG
PJL_1KG
CHB_1KG
JPT_1KG
CHS_1KG
CDX_1KG
KHV_1KG
OCE_CEPH
NAM_CEPH
PEL_1KG
MXL_1KG
CLM_1KG
PUR_1KG
0%
25%
50%
75%
100%
Haplotype frequency
Haplotypes
CT
TC
TT

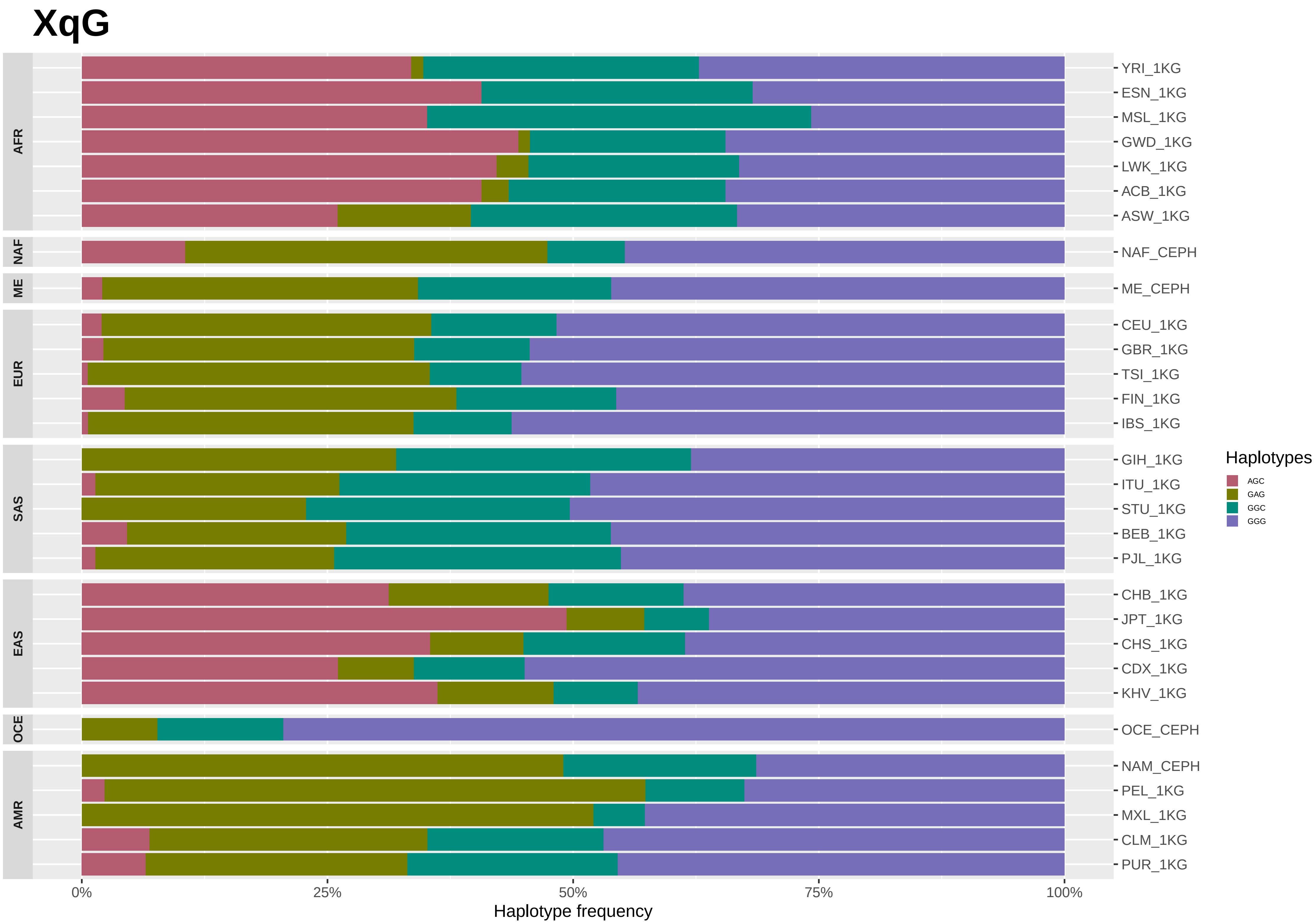
XqG
AFR
NAF
ME
EUR
SAS
EAS
OCE
AMR
YRI_1KG
ESN_1KG
MSL_1KG
GWD_1KG
LWK_1KG
ACB_1KG
ASW_1KG
NAF_CEPH
ME_CEPH
CEU_1KG
GBR_1KG
TSI_1KG
FIN_1KG
IBS_1KG
GIH_1KG
ITU_1KG
STU_1KG
BEB_1KG
PJL_1KG
CHB_1KG
JPT_1KG
CHS_1KG
CDX_1KG
KHV_1KG
OCE_CEPH
NAM_CEPH
PEL_1KG
MXL_1KG
CLM_1KG
PUR_1KG
0%
25%
50%
75%
100%
Haplotype frequency
Haplotypes
AGC
GAG
GGC
GGG

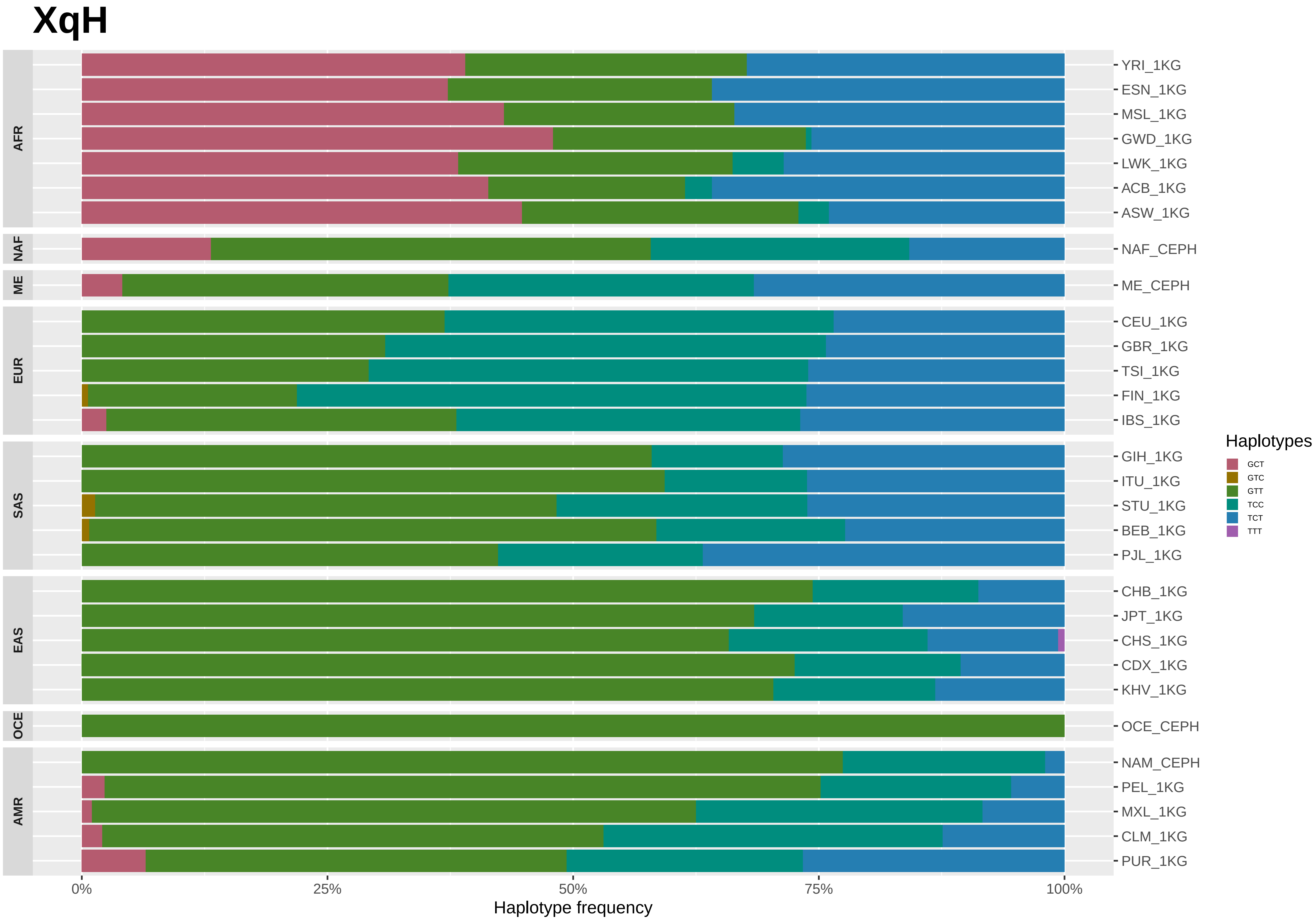
XqH
AFR
NAF
ME
EUR
SAS
EAS
OCE
AMR
YRI_1KG
ESN_1KG
MSL_1KG
GWD_1KG
LWK_1KG
ACB_1KG
ASW_1KG
NAF_CEPH
ME_CEPH
CEU_1KG
GBR_1KG
TSI_1KG
FIN_1KG
IBS_1KG
GIH_1KG
ITU_1KG
STU_1KG
BEB_1KG
PJL_1KG
CHB_1KG
JPT_1KG
CHS_1KG
CDX_1KG
KHV_1KG
OCE_CEPH
NAM_CEPH
PEL_1KG
MXL_1KG
CLM_1KG
PUR_1KG
0%
25%
50%
75%
100%
Haplotype frequency
Haplotypes
GCT
GTC
GTT
TCC
TCT
TTT
