# Supplementary material for: Broadening the Applicability of a Custom Multi-Platform Panel of Microhaplotypes: Bio-Geographical Ancestry Inference and Expanded Reference Data
Source: Front Genet. 2020 Oct 20;11:581041. doi: 10.3389/fgene.2020.581041 (PMC7606911; doi:10.3389/fgene.2020.581041)

Supplementary File S2-A: Mixture 1. Simulated profile of a 1:3 ratio mixture of HG02922 unadmixed ESN (AFR) and NA18939 unadmixed JPT (EAS).

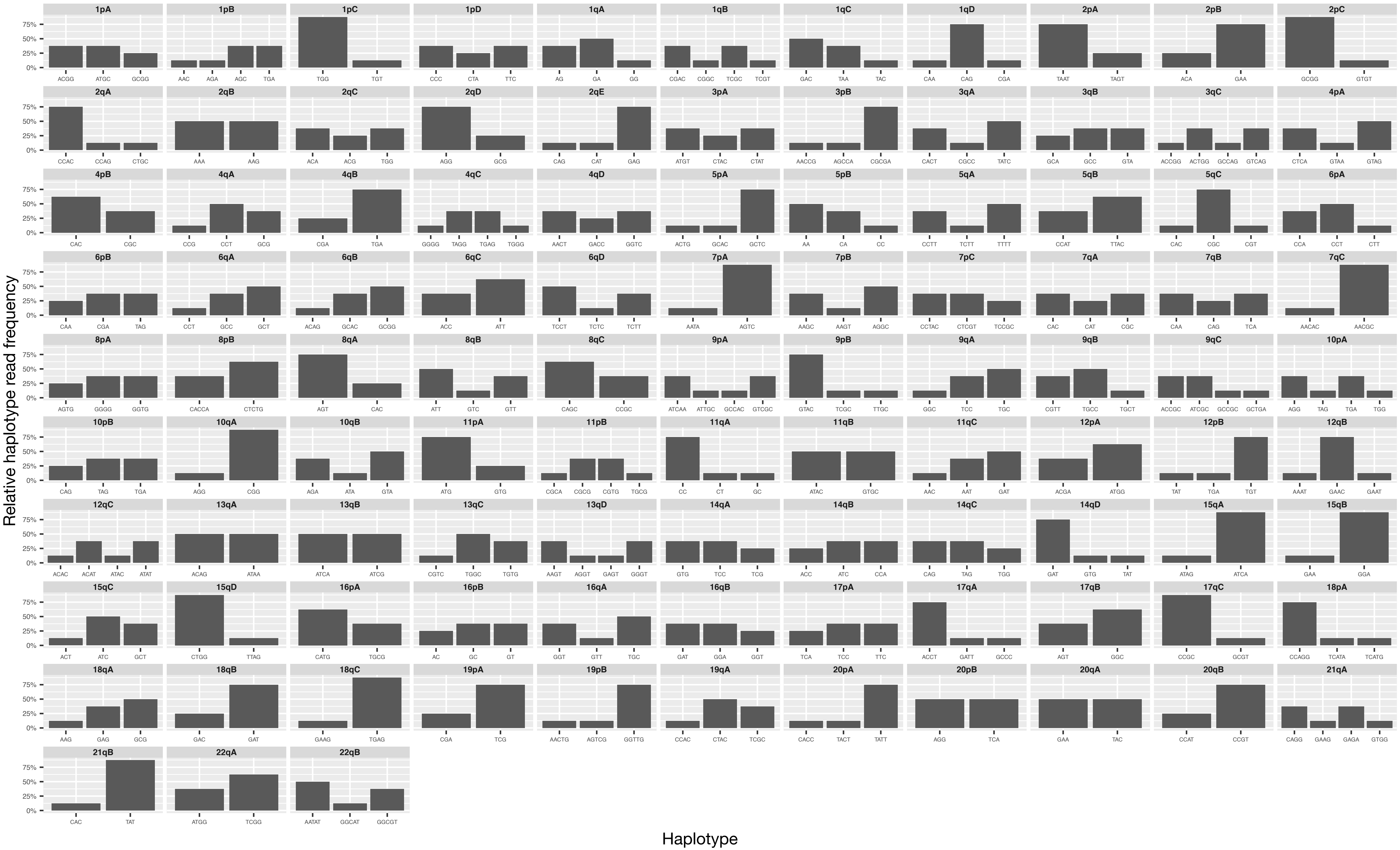

Supplementary File S2-B: Mixture 2. Simulated profile of a 1:5 ratio mixture of HG00097 unadmixed GBR (EUR) and HG00096 unadmixed GBR (EUR).

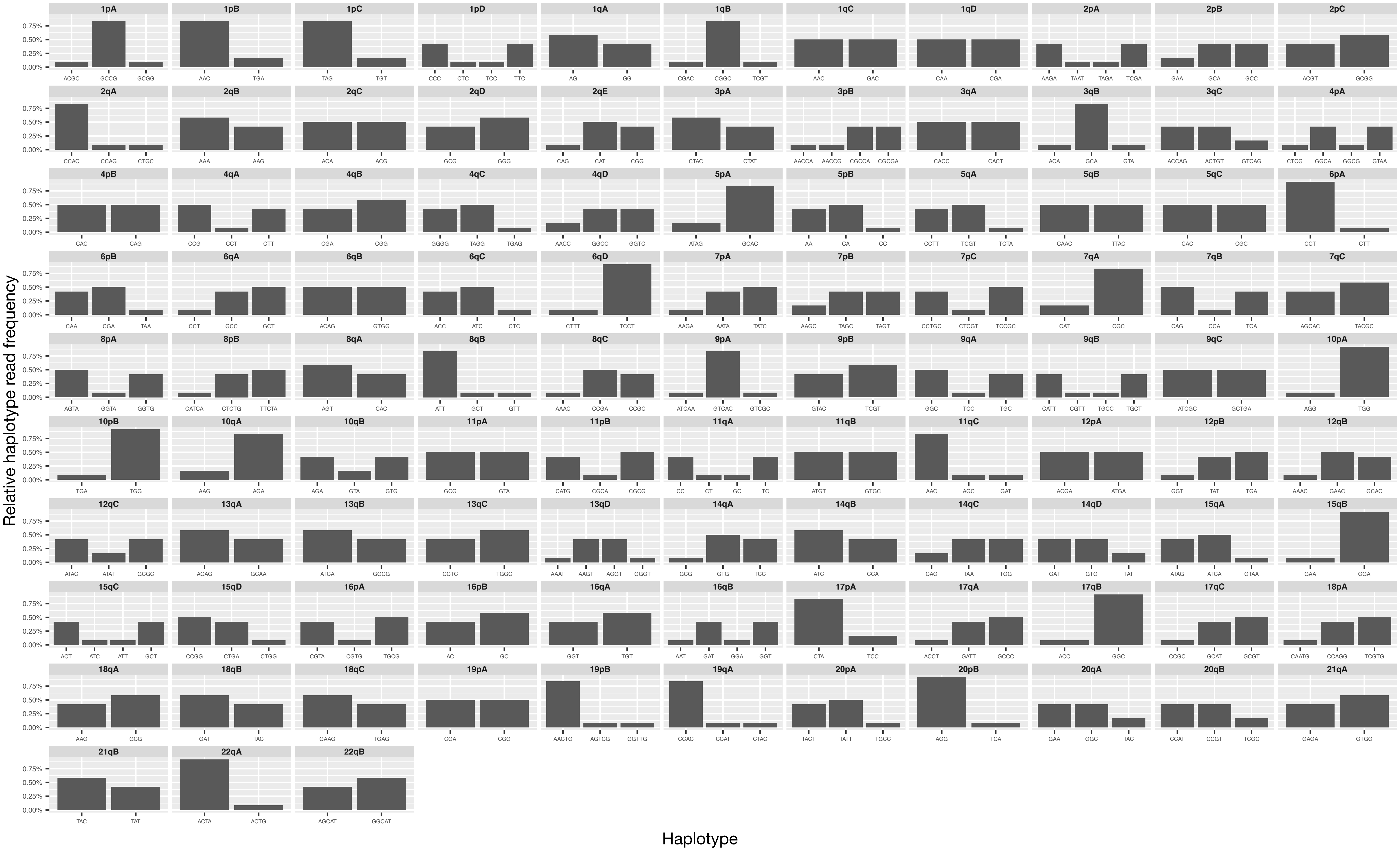

Supplementary File S2-C: Mixture 3. Simulated profile of a 1:7 ratio mixture of HG01565 admixed PEL (AMR) and HG00096 unadmixed GBR (EUR).

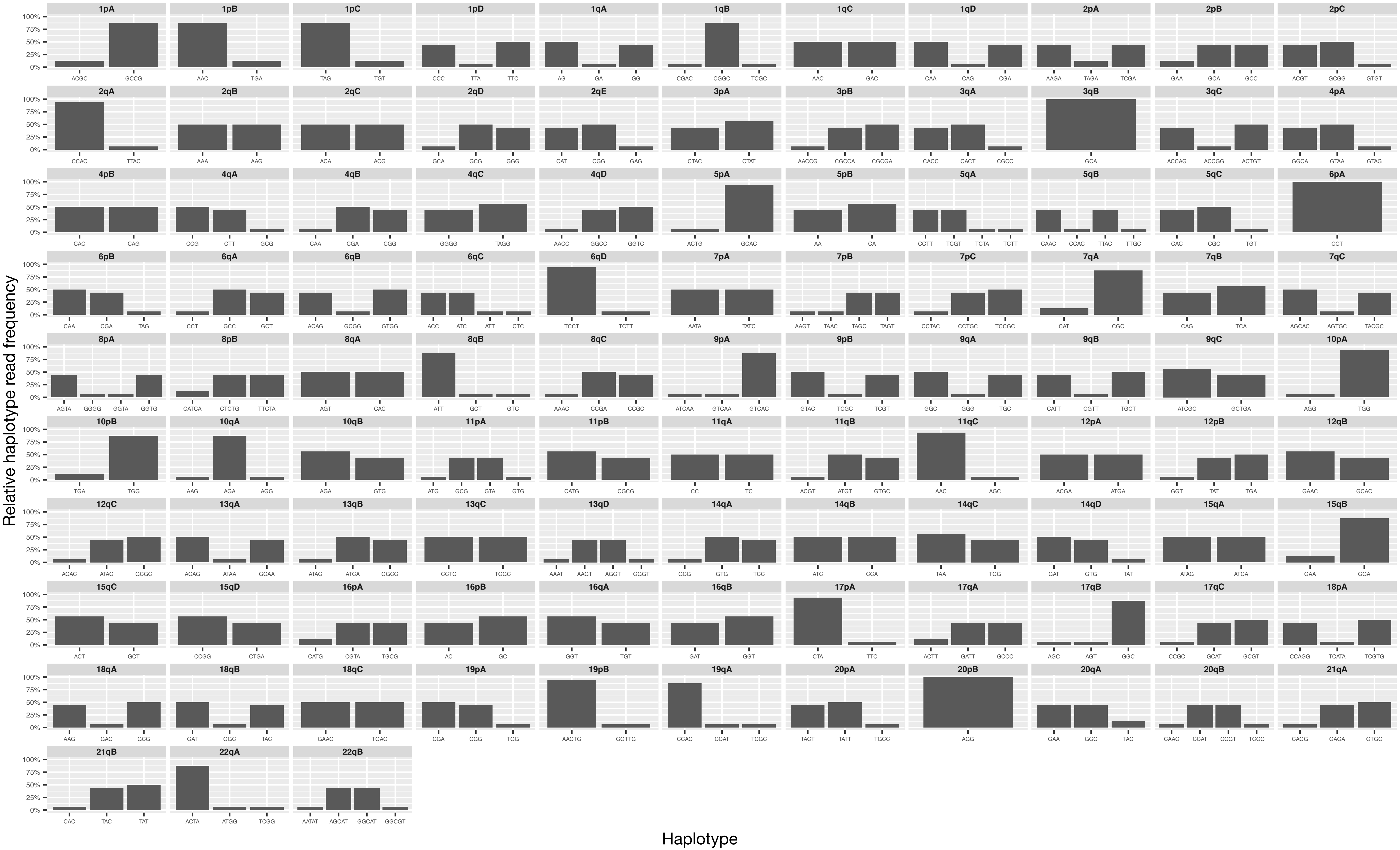

Supplement: Supplementary file 2 [file Data_Sheet_2.PDF]
