# Supplementary figures and images for: Broadening the Applicability of a Custom Multi-Platform Panel of Microhaplotypes: Bio-Geographical Ancestry Inference and Expanded Reference Data
Source: Front Genet. 2020 Oct 20;11:581041. doi: 10.3389/fgene.2020.581041 (PMC7606911; doi:10.3389/fgene.2020.581041)

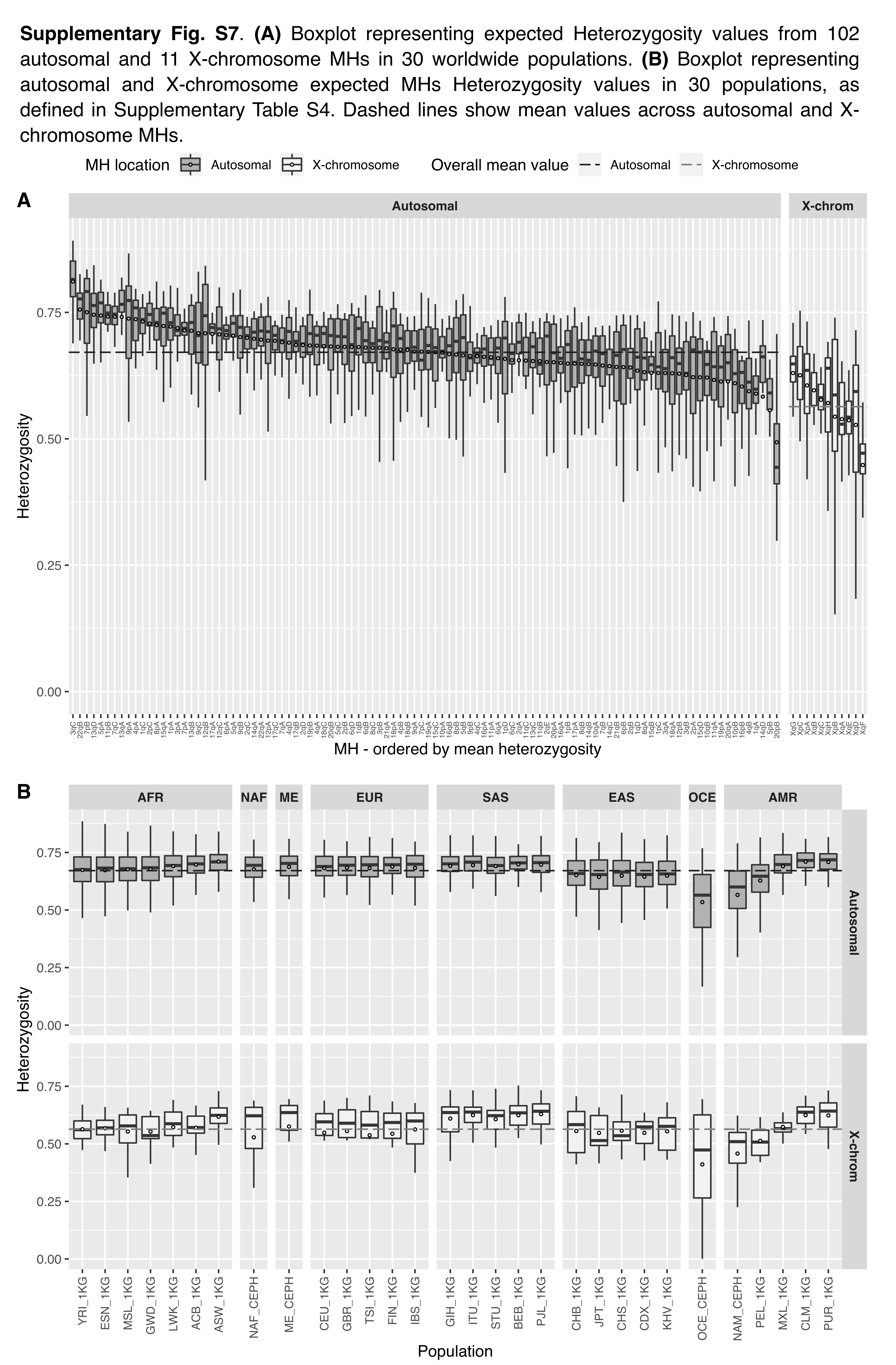

Supplement: Supplementary file 10 [file Image_7.JPEG]
